# Vinylic C–H Activation of Styrenes by an Iron–Aluminum Complex

**DOI:** 10.1021/jacs.3c14281

**Published:** 2024-02-02

**Authors:** Nikolaus Gorgas, Benedek Stadler, Andrew J. P. White, Mark R. Crimmin

**Affiliations:** † Department of Chemistry, Molecular Sciences Research Hub, Imperial College London, 82 Wood Lane, Shepherds Bush, London W12 0BZ, U.K.; ‡ Institute of Applied Synthetic Chemistry, Vienna University of Technology, Getreidemarkt 9, 1060 Vienna, Austria

## Abstract

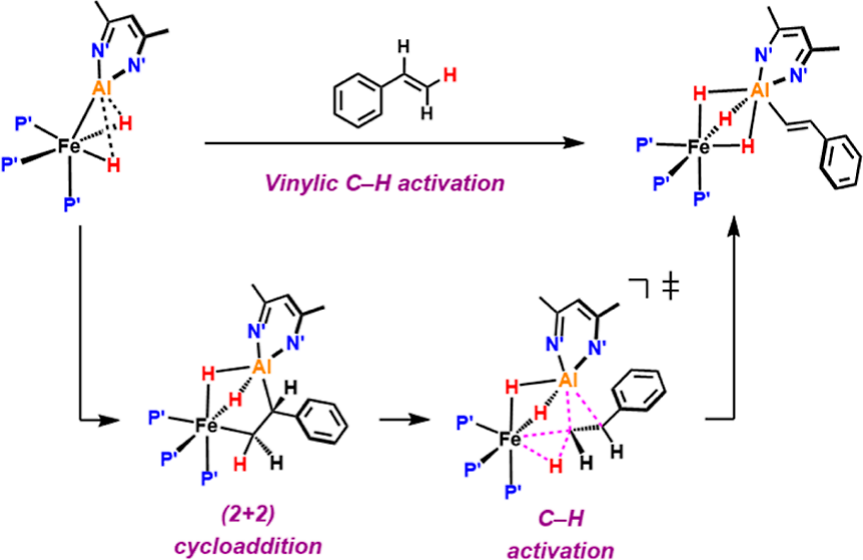

The oxidative addition of sp^2^ C–H bonds of alkenes to single-site transition-metal complexes is complicated by the competing π-coordination of the C=C double bond, limiting the examples of this type of reactivity and onward applications. Here, we report the C–H activation of styrenes by a well-defined bimetallic Fe–Al complex. These reactions are highly selective, resulting in the (*E*)-β-metalation of the alkene. For this bimetallic system, alkene binding appears to be essential for the reaction to occur. Experimental and computational insights suggest an unusual reaction pathway in which a (2 + 2) cycloaddition intermediate is directly converted into the hydrido vinyl product *via* an intramolecular sp^2^ C–H bond activation across the two metals. The key C–H cleavage step proceeds through a highly asynchronous transition state near the boundary between a concerted and a stepwise mechanism influenced by the resonance stabilization ability of the aryl substituent. The metalated alkenes can be further functionalized, which has been demonstrated by the (*E*)-selective phosphination of the employed styrenes.

## Introduction

**Figure 1:**
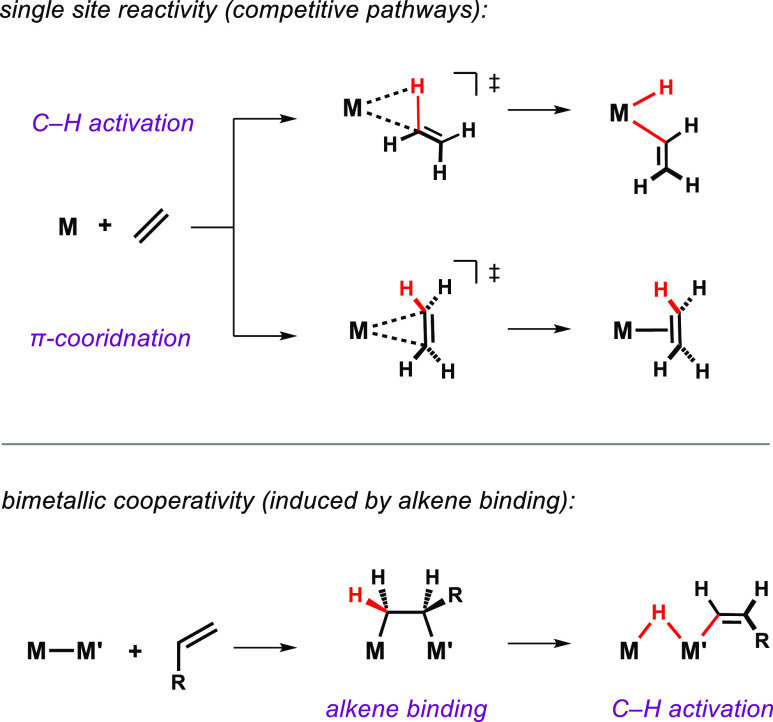
Single site vs bimetallic reactivity in vinylic C–H activations.

Our ability to selectively activate and functionalize C–H bonds in organic molecules is fundamental to countless chemical processes.^1^ Despite notable advances in this field,^2,3^ strategies for the selective functionalization of sp^2^ C–H bonds of alkenes are underdeveloped.^4−12^ This limitation can be traced back to fundamental selectivity issues that emerge in the reaction of alkenes with single-site transition-metal complexes. π-Coordination of the alkene to the metal is often kinetically accessible and nonreversible (Figure 1). The dominance of this pathway can effectively inhibit available mechanisms for vinylic C–H activation, including oxidative addition.^13^ This selectivity contrasts the rich chemistry of aromatic sp^2^ C–H or aliphatic sp^3^ C–H bonds where substrate binding (π-coordination or σ-complex formation) is typically reversible and a prerequisite for C–H bond breaking by oxidative addition.^14^

For example, Bergman and co-workers have studied the reaction of ethylene with the 16-electron reaction intermediate [IrCp*(PMe)_3_]. They found that π-complexation of the alkene is thermodynamically favored with respect to the oxidative addition of vinylic sp^2^ C–H bonds. Moreover, the π-complex was found to be not an intermediate in the lowest energy C–H activation process.^3,4^ Computational studies support the conclusions and suggest that π-coordination and C–H activation of the alkene are separate and competitive pathways.^15−18^

We recently reported a well-defined Fe–Al complex (**1**) that is capable of selectively breaking the sp^2^ and sp^3^ C–H bonds of pyridine substrates as well as acetonitrile.^19−21^ Herein, we present C–H activation in the vinylic position of styrenes using the same Fe–Al system. These reactions are highly selective, resulting in a rare (*E*)-β-metalation of the alkenes. In contrast to single-site systems, alkene binding appears to initiate C–H activation and is essential for the reaction to take place. An unusual reaction pathway in which a (2 + 2) cycloaddition intermediate is directly converted into the hydrido vinyl product is proposed. This new mechanism results in the net oxidative addition of an alkenyl sp^2^ C–H bond across the two metal centers and opens up new possibilities for selective alkene functionalization by C–H activation using a bimetallic approach.^22−26^

## Results and Discussion

### Nonreversible (2 + 2) Alkyne Binding

**Figure 2:**
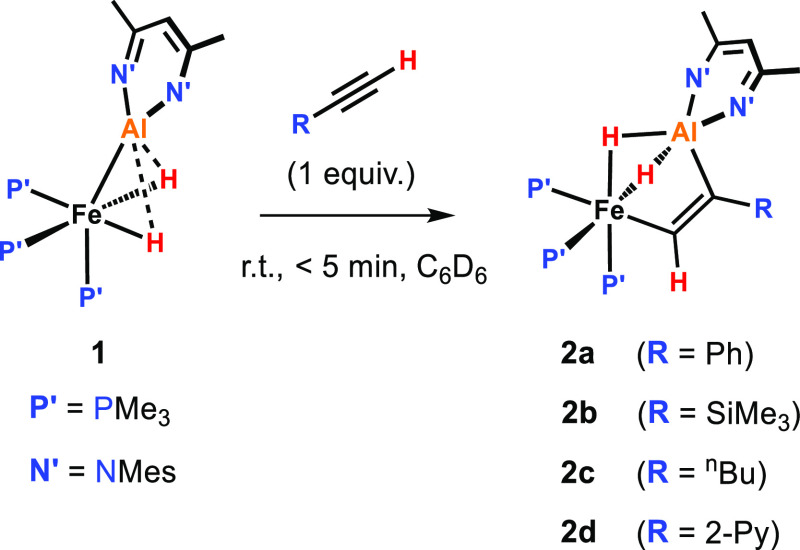
Nonreversible addition of terminal alkynes to **1**.

Addition of 1 equiv of a terminal alkyne RC≡CH (R = Ph, SiMe_3_ or *n*-Bu, 2-Py) to a solution of **1** in C_6_D_6_ at room temperature resulted in an immediate color change from dark to bright orange, in each case leading to the quantitative formation of the cycloaddition products **2a**–**d** (Figure 2). These reactions appear to be nonreversible. **2a**–**d** were all isolated in yields of >95% and are stable in both solution and in the solid state. **2b** was characterized by single-crystal X-ray diffraction (Figure 6a).

**2a**–**d** all give rise to very similar and characteristic NMR signals for the coordination of the alkyne. For example, the ^31^P{^1^H} NMR spectrum of **2a** exhibits a mutually coupled spin system comprising a doublet at δ_P_ = 29.0 ppm (2P) and a triplet resonance at 19.9 ppm (1P), consistent with the chemical nonequivalence of the axial and equatorial phosphine ligands. In the ^1^H NMR spectrum, the bridging hydrides appear as a broadened virtual triplet at δ_H_ = −14.71 ppm, and a doublet of triplets at δ_H_ = 10.02 ppm (^3^*J*_HP_ = 13.4 and 5.6 Hz) can be found for the ArC≡C*H* proton of the coordinated alkyne.^27^

### Reversible (2 + 2) Alkene Binding

**Figure 3:**
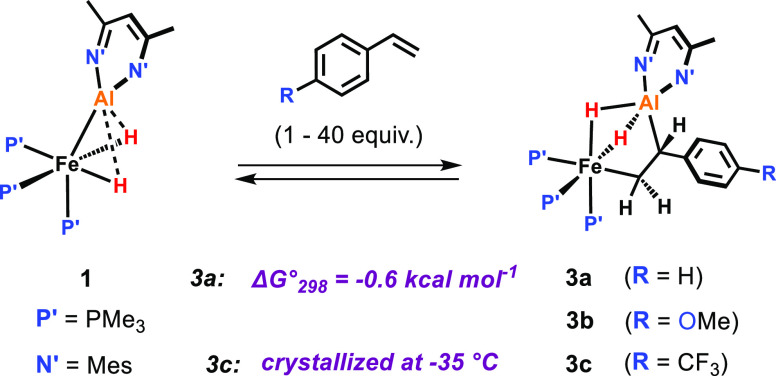
Reversible cycloaddition of styrenes to **1**.

Styrene substrates were found to bind reversibly to **1** (Figure 3). The stepwise addition of excess styrene (10–40 equiv) to **1** in C_6_D_6_ at room temperature led to the gradual appearance of a new species **3a**, suggesting an equilibrium established immediately after each addition. A Van’t Hoff analysis of the reaction of **1** with excess styrene (21.6 equiv) was conducted in toluene-*d*_8_ over a temperature range of 248–298 K. The formation of **3a** was found to be slightly exergonic: Δ*H*°_298_ = −12.5 kcal mol^–1^ and Δ*G*°_298_ = −0.4 kcal mol^–1^.

The formation of **3c** appeared to be more energetically favorable. Addition of 4-(trifluoromethyl)styrene (1.5 equiv) to a solution of **1** in toluene-*d*_8_ at −35 °C resulted in an immediate color change from dark red to bright orange, and the NMR spectra recorded at the same temperature revealed that the equilibrium between **1** and **3c** has been completely shifted to the product side. **3c** could be crystallized from *n*-pentane at −35 °C, and the solid-state structure was analyzed by single-crystal X-ray diffraction (Figure 6b).

In the ^1^H NMR spectrum of **3c** recorded at −35 °C, two broad apparent triplets at δ_H_ = −14.92 and −15.93 ppm can be found for the bridging Fe–(μ-*H*)_2_–Al hydrides as well as another three broad signals at δ_H_ = 2.74, 1.03, and 0.64 ppm for the ArC*H*=C*H*_2_ protons of the coordinated alkene group. In the ^31^P{^1^H} NMR spectrum, the three PMe_3_ ligands of **3c** appear as a well-resolved ABX spin system: the AB part centered at δ_P_ = 36.5 ppm (*J*_AB_ = 41.5 Hz) and the X part at 22.2 ppm.

### Vinylic C–H Activation

**Figure 4:**
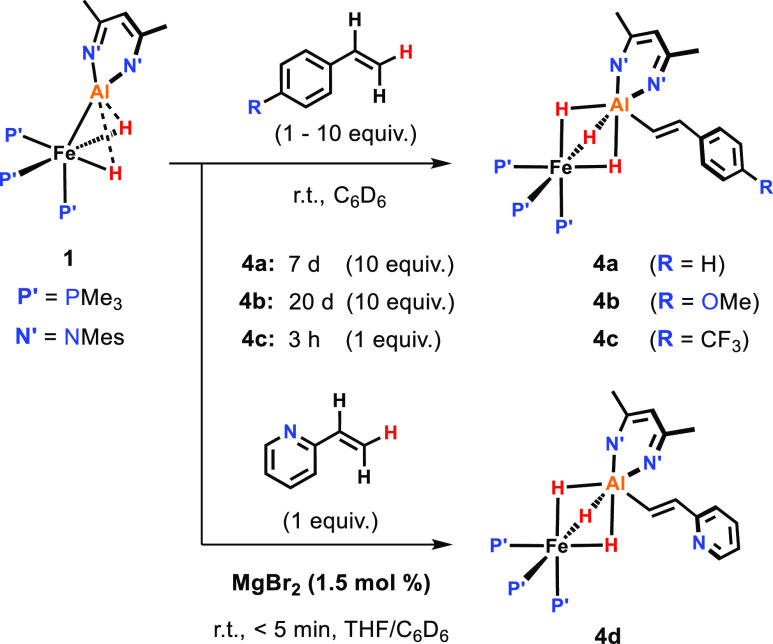
Vinylic C–H activation of styrene substrates.

Over the course of 14 days, the room-temperature reaction of **1** with styrene (1 equiv) in C_6_D_6_ afforded the vinylic C–H activation product **4a** in 78% NMR yield (Figure 4).^28^**4a** was isolated in a pure form and crystals suitable for X-ray diffraction were grown, confirming the (*E*)-β-alumination of styrene (Figure 6c). The new species exhibits a broadened hydride resonance at δ_H_ = −15.58 ppm in the ^1^H NMR spectrum integrating to 3H and a singlet at δ_p_ = 29.0 ppm in the ^31^P{^1^H} NMR spectrum characteristic for a (PMe_3_)_3_Fe–(μ-*H*)_3_–Al motif that resulted from the C–H activation reaction.^19−21^ The PhC*H*=C*H* protons resonate at δ_H_ = 7.86 and δ_H_ = 7.34 ppm, showing a large coupling constant of ^3^*J*_HH_ = 19.9 Hz diagnostic for an (*E*)-configuration of the C=C double bond.

Styrene derivatives containing electron-withdrawing substituents reacted much more quickly than those with electron-donating groups. For example, upon addition of 1 equiv of 4-(trifluoromethyl)styrene **4c** was formed nearly quantitatively within 3 h. In the case of **4a** and **4b**, an excess of the respective styrenes (10 equiv) was necessary to obtain reasonable reaction rates and full conversion of **1** at room temperature. The formation of the products in these cases was found to follow first-order kinetics, with *t*_1/2_ ≈ 24 h (**4a**) and *t*_1/2_ ≈ 71 h (**4b**).^29^

The highest reaction rates at room temperature were observed in the reaction of **1** with 2-vinylpyridine which affords **4d** almost instantly when carried out in the presence of catalytic amounts (1–2 mol %) of MgBr_2_. MgBr_2_ appears to act as a Lewis acid catalyst preventing coordination of the pyridine nitrogen to Al and activating the substrate for the C–H activation reaction (vide infra).

### Nonreversible (2 + 4) Addition

**Figure 5:**
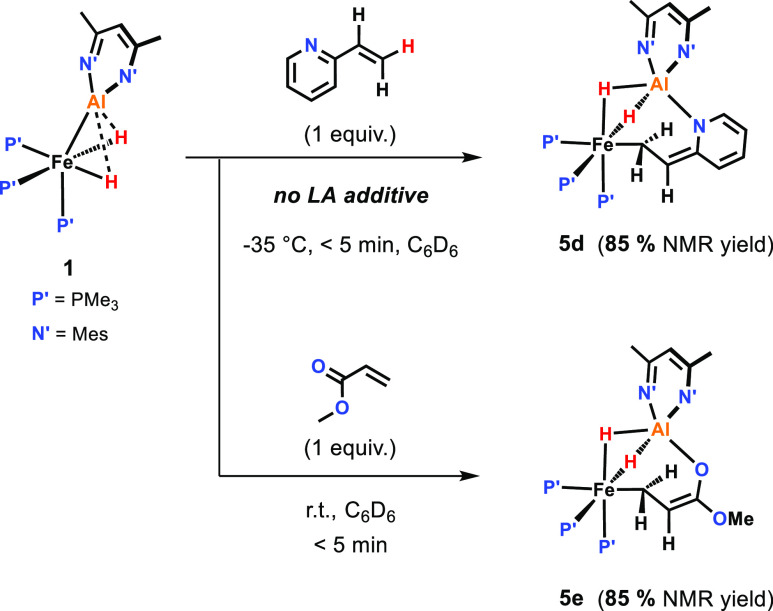
(2 + 4) additions to **1**.

In the absence of the Lewis acid additive, 2-vinylpyridine forms a (2 + 4) cycloaddition product with **1** (Figure 5). Addition of 2-vinylpyridine to a solution of **1** in toluene-*d*_8_ at −35 °C resulted in the formation of **5d** in ca. 85% NMR yield alongside **4d** as a minor side product. The ^1^H NMR spectrum of **5d** recorded at −40 °C shows a sharp triplet resonance at δ_H_ = 3.81 ppm, diagnostic for the α-C*H* group of the (2 + 4) bound substrate. Warming the reaction solution to room temperature resulted in the slow decomposition of **5d** and did not convert it into **4d** (see Supporting Information). These results suggest that the (2 + 4) addition represents a competitive pathway preventing the C–H activation reaction. The analogue product **5e** was obtained from the reaction of **1** with methyl acrylate at room temperature, with the (*E*)-C–H activation product **4e** also being formed in a 15% NMR yield.

**Figure 6:**
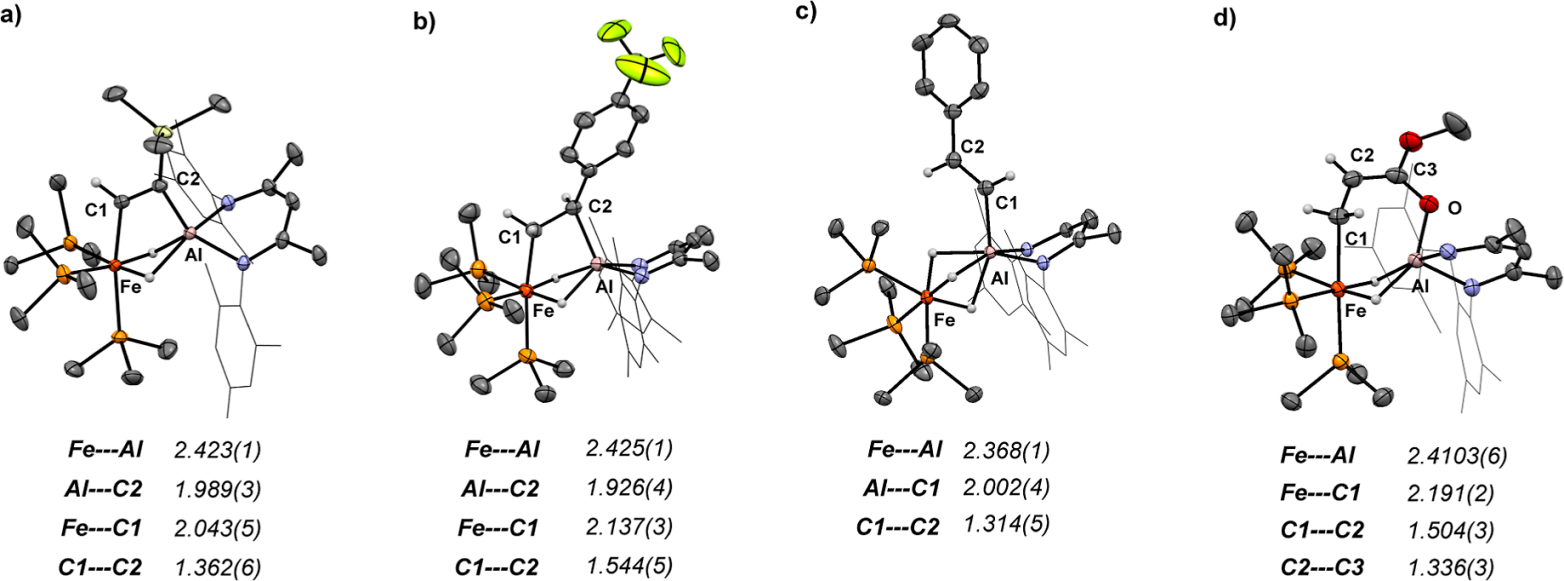
X-ray structures of **2b** (a), **3c** (b) **4a** (c), and **5e** (d). Bond lengths are given in Å.

In the case of **5e**, crystals suitable for X-ray diffraction could be grown, confirming the structure of the (2 + 4) cycloaddition product with the β-CH_2_ attached to Fe and the oxygen of the former carbonyl group bound to Al (Figure 6d).

### Structure and Bonding

**2b**, **3c, 4a**, and **5e** were characterized by single-crystal X-ray diffraction, and their solid-state structures are depicted in Figure 6. In **4a**, the Fe–Al (2.368(1) Å) as well as Al–C (2.002(4) Å) distances are very similar for the C–H activation products of **1** reported previously.^19−21^ The Fe–Al separation in **2b** (2.423(1) Å) and **3c** (2.425(1) Å) is longer than in **4a** and **1** (2.217(6) Å) but comparable to the parent dibromide complex [(PMe_3_)_3_(Br)Fe-(μ-H)_2_-Al(Br)(^Mes^BDI)] (2.453(1) Å, BDI = bis(β-diketiminate)).^19^ The C–C distances of the alkene/alkyne substrate get elongated upon binding (**3c**: 1.544(5) Å; **2b**: 1.362(6) Å) and are consistent with the formulation as a C–C single (ethane: 1.535 Å) or C=C double bond (ethylene: 1.339 Å).^30^ Similarly, the C–C bond lengths of the bound substrate in **5d** (C1–C2: 1.504(3) Å; C2–C3: 1.336(3) Å) are in accordance with an enolate tautomeric structure depicted in Figure 6.

DFT calculations were conducted to gain further insight into the alkene/alkyne binding to **1**.^31^ Calculations on the thermochemistry reveal that the cycloaddition products are significantly more stable for alkynes than for alkenes. For example, the formation **2a** is exergonic by Δ*G* = −31.2 kcal mol^–1^, whereas the binding of styrene in **3a** only results in a moderate stabilization of Δ*G* = −2.3 kcal mol^–1^.

Alkene binding to **1** was further investigated by ETS-NOCV (extended transition state-natural orbital for chemical valence) calculations (see Figure S14). Donation of electron density from the former Fe–Al bond into the π* orbital of the substrate (**3a**: Δρ_1_ = −420.7 kcal mol^–1^) accounts for >90% of the total orbital stabilization energy (**3a**: Δ*E*_orb_ = −421.0 kcal mol^–1^). Natural bond orbital (NBO) analysis identified a σ-bond between iron and the β-carbon of the substrate, whereas bonding of Al to the α-carbon is defined as the donor–acceptor interaction between a C-centered lone pair and empty s/p orbitals in Al possessing a partial ionic character. This is underpinned by the charges from NPA (natural population analysis), revealing that the Al–C bond is more polarized (**3a**: Al + 1.80, C −0.88) in comparison to the Fe–C bond (**3a**: Fe −0.52, C −0.80).

### Mechanism of the Vinylic C–H Activation

**Figure 7:**
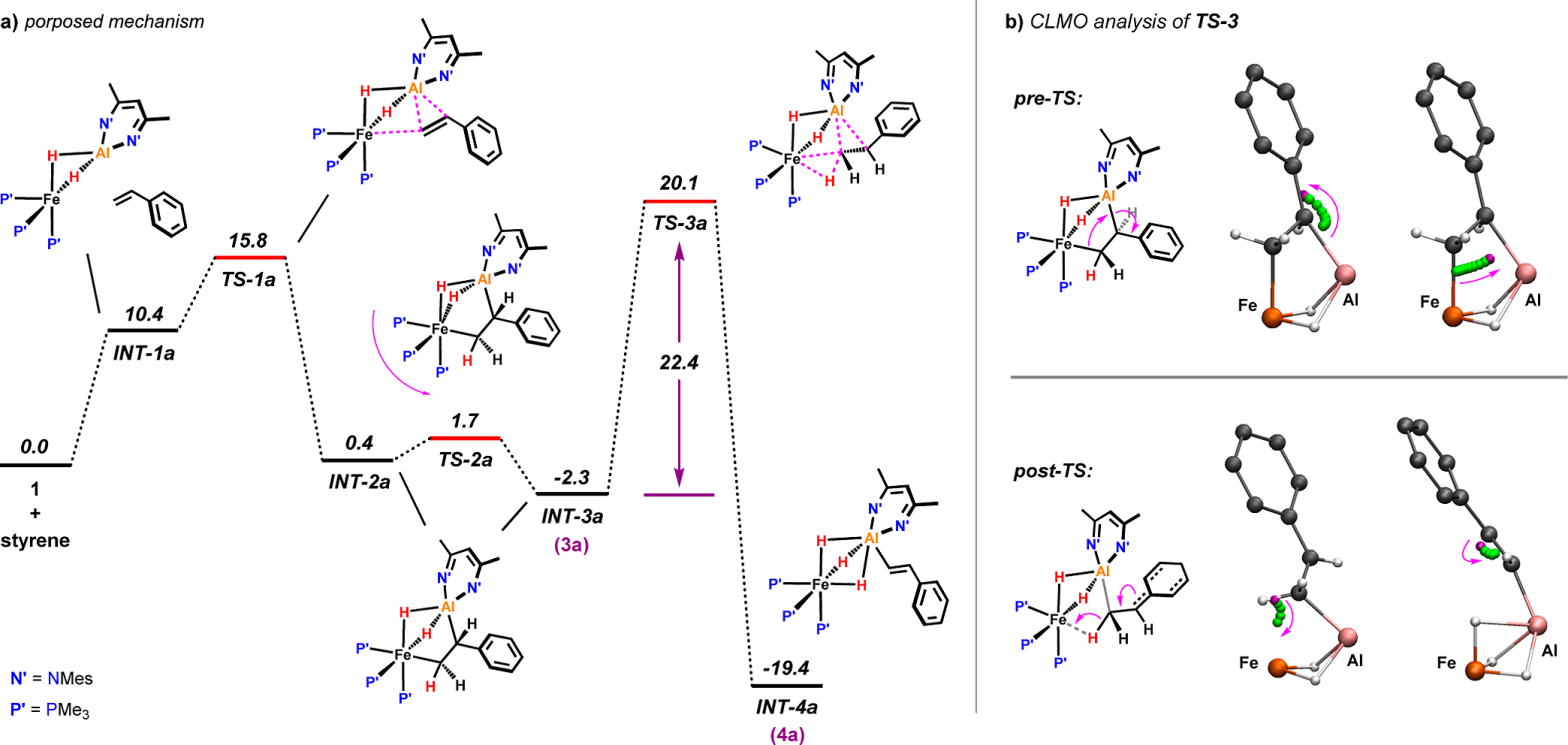
(a) Calculated free-energy profile for the vinylic C–H activation of styrene. Energies are in kcal mol^–1^. B3LYP-D3/Def2-TZVPP/SDDAll (Fe,Al)/PCM (benzene)//B3PW91-D3/6-31G**/SDDAll (Fe,Al)/PCM (benzene). (b) Visualization of bond rearrangements in **TS-3** using LMO centroids (CLMOs).

DFT calculations were also undertaken on the mechanism of vinylic C–H activation of styrenes.^32^ A low-energy pathway was identified involving direct intramolecular C–H activation of the bound styrene in **3a** (Figure 7a).

The mechanism is initiated through the concerted but asynchronous addition of the C=C double bond to **1**. This first step has a low activation barrier of Δ*G*^⧧^_298K_ = 15.8 kcal/mol (**TS-1a**) and is moderately exergonic by −2.3 kcal/mol (**INT-3a**), in accordance with the experimentally observed reversibility of the reaction. The resulting alkene complex was found to adopt two different conformations (**INT-2a** and **INT-3a**) with respect to their distortion along the Fe–Al vector. These conformers appear to be close in energy (ΔΔ*G*°_298K_ = 2.7 kcal/mol) and are separated by barriers of just 2.1 and 4.0 kcal/mol, respectively. From **INT-3a**, vinylic C–H activation proceeds intramolecularly through **TS-3a** to directly give the final product **INT-4a**. This step represents the highest barrier (22.4 kcal/mol) of the entire pathway. The formation of **INT-4a** is exergonic by −19.4 kcal/mol, consistent with a nonreversible process.

**Figure 8:**
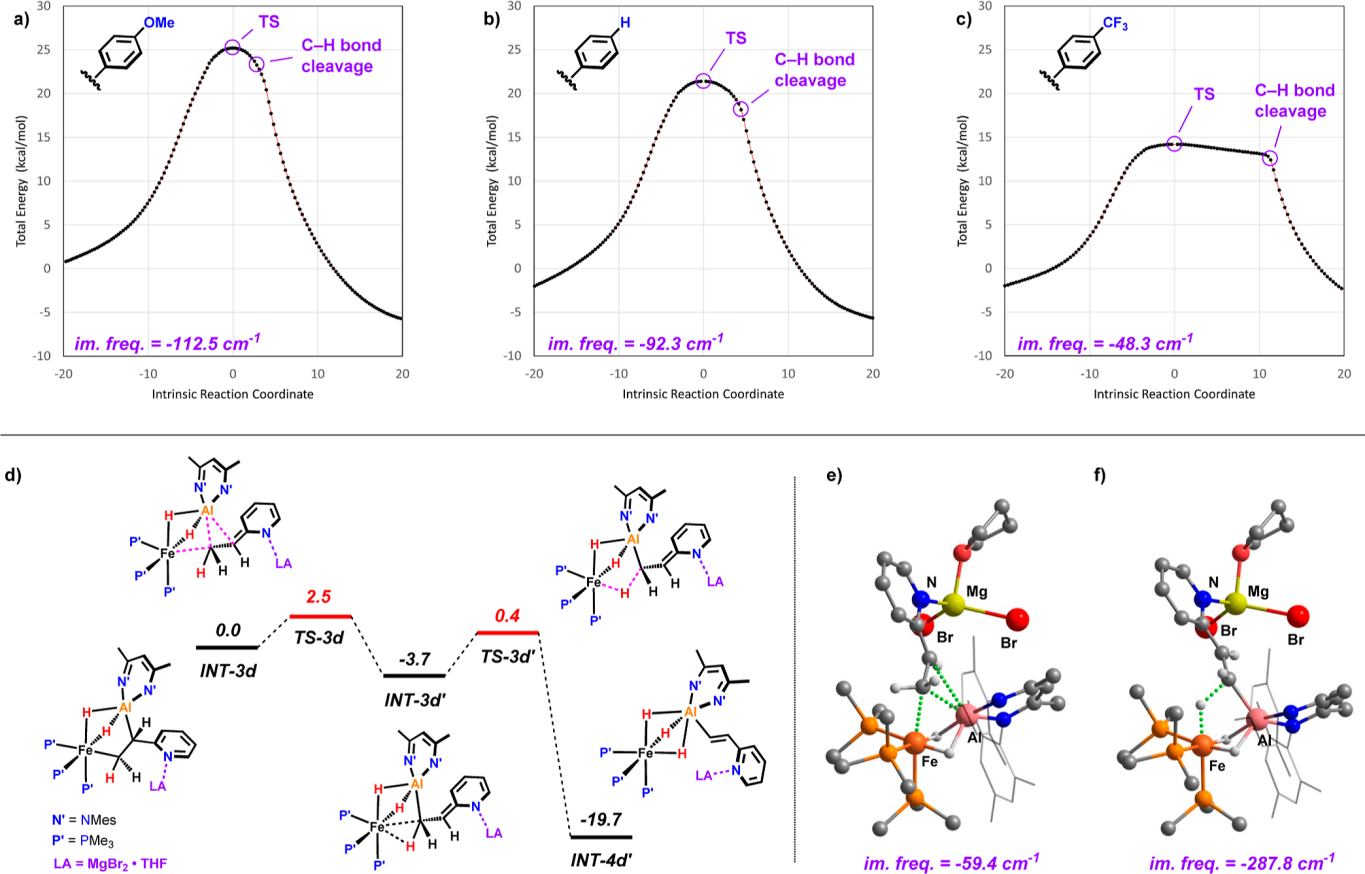
a–c) IRCs around **TS-3** employing different styrene substrates. (d) Gibbs free-energy profile for the stepwise C–H activation process with 2-vinylpyridine in presence of a Lewis acid catalyst (free energies in kcal mol^–1^). Optimized structures of the transition states **TS-3d** (e) and **TS-3d’** (f).

**TS-3a** appears to be a highly asynchronous transition state near the boundary between a concerted and a stepwise mechanism (*vide infra*). The low imaginary frequency (−92.3 cm^–1^) of **TS-3a** refers to the reorientation of the bound alkene in which the α-C is moving away from the Al center, while the β-C is transferred from Fe to Al. There is no stationary point for the actual cleavage of the C–H bond, which occurs along the intrinsic reaction coordinate en route to **INT-4a** (see the intrinsic reaction coordinate (IRC) in Figure 8b). The (*E*)-stereospecific nature of the C–H activation is a direct consequence of the orientation of the substituents in the transition state **TS-3a** (*vide infra*).

In order to get better insight into the nature of **TS-3a**, an analysis of the key localized molecular orbitals (LMOs) along the IRC was carried out. LMOs were calculated following the Pipek-Mezey criterion,^33^ and a procedure^34^ described by Vidossich and Lledóss was used to generate centroids of these LMOs (CLMOs). The CLMOs were used to follow the bond rearrangements around **TS-3a** (Figure 7b).^35^ For the sake of clarity, the depiction of the overall process was separated into pre- and post-TS stages. From **INT-3a** to **TS-3a** (pre-TS), migration of the β-C of the bound alkene goes along with an electron transfer from the Fe–C bond to Al, while the electrons from the Al–C are shifted toward the α-C and get delocalized through participation of the arene π-system (*vide infra*). In the post-TS stage, the most significant rearrangement of the electronic structure occurs during C–H bond cleavage. Electrons from the C–H σ-orbital are shifted toward Fe, forming the Fe–H bond. This process is consistent with a hydride transfer and distinct from the reaction of **1** with pyridines^19,20^ and CH_3_CN,^21^ which are found to proceed via a proton transfer (reductive deprotonation). Simultaneously, the delocalized lone pair at the α-C is shifted back toward the β-C to re-establish the π-system of the C=C double bond in the vinyl ligand.

The proposed mechanism is supported by deuterium labeling studies. A comparison of the reaction rates of **1** with styrene and styrene-*d*_8_ at 323 K from two separate experiments resulted in a kinetic isotope effect (KIE) of 2.64 ± 0.04. The overall KIE is likely affected by an additional equilibrium isotope effect (EIE) caused by the reversible alkene binding, which, however, appeared to be relatively small at room temperature (EIE = 1.06 at 298 K). This contrasts with the *ortho* C–H activation of pyridine for which an unusually large *k*_H_/*k*_D_ value of 14.0 ± 0.2 at 298 K was obtained, likely caused by a quantum tunneling and thus diagnostic for a proton-transfer reaction.^19^

The C–H activation step was calculated for the entire series of **2a–d** (Table 1). The obtained activation barriers correlate well with the Hammett parameters^36^ of the respective substituents and reflect the relative reactivities of these substrates observed experimentally. Across the series, the imaginary frequencies of **TS-3a–c** are getting lower with more positive Hammett parameters, indicating flattening of the potential energy surface around the transition state.

**Table 1:**
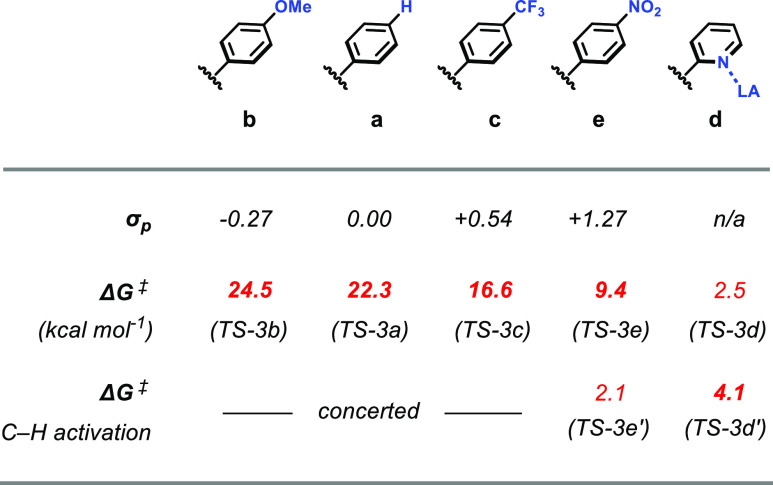
Substituent Effect on the Activation Energies for **INT-3** to **INT-4** in the Reaction of Styrenes with **1**

More insight was gained from the analysis of the IRCs (Figure 8a–c). While the IRC for 4-methoxystyrene is almost symmetrical around the transition state, it forms a plateau for 4-(trifluoromethyl)styrene flanked by a steep decay, marking the onset of the C–H bond cleavage. Computationally, the series was expanded to 4-nitrostyrene^37^ due to the large positive Hammett parameter of the NO_2_ substituent (σ^–^ = +1.27).^38−40^ In this extreme case, complete deconvolution of the rearrangement and C–H activation of the bound substrate into two separate transition states were obtained (**TS-3e**, Δ*G*^⧧^ = 9.4 kcal/mol, and **TS-3e**′, Δ*G*^⧧^ = 2.1 kcal/mol; see Supporting Information).^41^ This trend as well as the calculated activation barriers can be traced back to the resonance stabilizing effect of the para-substituent. A comparison of **TS-3** down the series shows that the C(α)–C(Ar1) bond distances are getting shorter, indicating an increasing double bond character (**TS-3b**: 1.412 Å and **TS-3e**: 1.384 Å). At the same time, NBO analysis of **TS-3** reveals the negative charge accumulation at the α-C decreases in the same order (**TS-3b**: −0.59 and **TS-3e**: −0.48). This ability to stabilize the negative charge at the α-C position appears to be the key factor for the reactivity. To some extent, these findings resemble recent studies on the transition from a stepwise to a concerted behavior of S_N_Ar reactions.^42,43^

The same effect was observed for the MgBr_2_-promoted reaction of 2-vinylpyridine with **1** (Figure 8d–f). Binding of the Lewis acid to the pyridine nitrogen allows for an extreme resonance structure, resulting in a stepwise process and an even lower barrier than for 4-nitrostyrene. The emergent intermediate (**INT-3d′**, Δ*G* = −3.7 kcal/mol) is just slightly lower in energy than the adjacent barriers (**TS-3d**: Δ*G* = 2.5 kcal/mol and **TS-3d′**: Δ*G* = 0.4 kcal/mol).

NBO analysis provided further insight into the nature of this intermediate. In **INT-3d′**, the former π-system of the alkene remains broken resulting in two separated p-orbitals. The β-carbon appears to be sp^3^-hybridized carrying a negative partial charge (−1.10) stabilized through coordination to aluminum. The α-C appears to be sp^2^-hybridized, forming a resonance structure with the adjacent pyridyl group and thus carries a much lower negative partial charge (−0.46).

Binding of the substrate in **INT-3d′** was further stabilized through an agostic interaction between one of the β-C–H bonds and the iron center. In **TS-3d′**, this C–H bond is cleaved (*vide supra*). With the lone pair on the α-C, the alkene π-system is re-established forming a C=C double bond which, as a consequence of the antiperiplanar conformation of the C–C bond in **INT-3d′**, adopts the final (*E*)-configuration.

### C–H Phosphination

**Figure 9:**
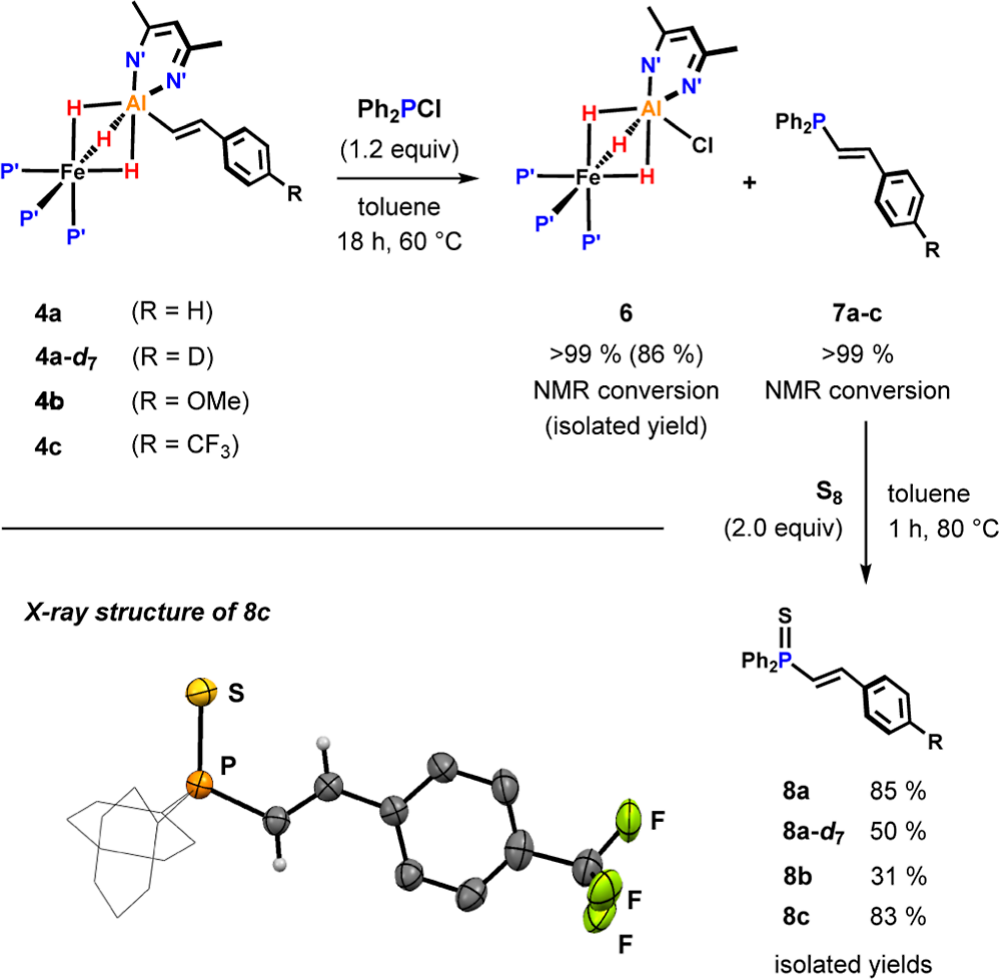
Stoichiometric C–H phosphination of styrenes.

The alkenyl group in the C–H aluminated products can be further functionalized. This is demonstrated with a small scope of styrene substrates that gave C–H activation products **4a–c**. Reacting these compounds with chlorodiphenylphosphine at 60 °C for 18 h in toluene leads to the quantitative conversion of **4a–c** to afford the chlorinated complex **6** and the (*E*)-β-vinylphosphines **7a–c** with excellent stereoselectivity (Figure 9). **7a–c** are highly sensitive to air and were isolated as air-stable thiophosphines **8a–c**. Even deuterated vinylphosphine **8a–*d*_7_** can be obtained as exemplified by the phosphination of **4a–*d*_7_**. This method represents a rare example of a direct vinylic C–H phosphination.^3,44^

## Conclusions

In summary, we report the vinylic C–H activation of styrenes with a bimetallic Fe–Al complex. These reactions were found to proceed via a novel mechanism involving binding of the alkene across the Fe–Al bond, followed by an intramolecular transition state for the C–H bond cleavage.

For monometallic systems, the direct oxidative addition of the sp^2^ C–H bond in an unactivated alkene is hard to achieve. This is well documented and can be rationalized by the competing π-coordination of the alkene over the formation of a weakly bound σ-C–H complex vital to the bond cleavage at the transition-metal center.^5^

By contrast, our findings suggest that alkene binding is essential for the C–H activation to take place in the present bimetallic system. The (2 + 2) cycloaddition of the styrene substrates is weak and appears to initiate a low-energy pathway.

We identified an unusual transition state connecting the alkene complex with the final hydrido vinyl product. This key step proceeds through a highly asynchronous transition state near the boundary between a concerted and a stepwise mechanism influenced by the resonance stabilization ability of the aryl substituent. Moreover, the geometry of the transition state results in the selective metalation of the (*E*)-β-C–H bond of the substrate. Our preliminary results on the C–H phosphination of styrenes demonstrate the potential of this concept for C–H functionalization reactions and might stimulate future developments toward catalytic processes.

## Data Availability

Raw NMR and computational data are available at the following repository: https://doi.org/10.14469/hpc/13502.

## Supporting Information Available

Synthetic procedures, kinetic experiments, NMR spectra of all compounds, crystal structures of **4d**, **S1**, and **S2**, crystallographic data, and computational methods (PDF)
Cartesian coordinates of the DFT-optimized structures (XYZ)

## Supplementary Material

ja3c14281_si_001.pdf

ja3c14281_si_002.xyz
