# Supplementary material for: Vinylic C–H Activation of Styrenes by an Iron–Aluminum Complex
Source: J Am Chem Soc. 2024 Feb 2;146(6):4252–9. doi: 10.1021/jacs.3c14281 (PMC10870711; doi:10.1021/jacs.3c14281)
Supplement: Supplementary file 1 — ja3c14281_si_001.pdf [file ja3c14281_si_001.pdf]

Supporting Information for:

Vinylic C–H activation of Styrenes by an Iron–Aluminium Complex

Nikolaus Gorgas*[,†,‡], Benedek Stadler[†], Andrew J. P. White[†], and Mark R. Crimmin*[,†]

[†]Department of Chemistry, Imperial College London, Shepherds Bush, London, W12 0BZ, UK

[†]Institute for Applied Synthetic Chemistry, TU Vienna, Getreidemarkt 9, 1060 Vienna, Austria.

1. General Experimental **2**

2. **Synthetic Procedures** **3**

3. Reversible Alkene Binding **24**

4. Kinetic Experiments **26**

6. X-Ray Data **41**

7. NMR Spectra of new Compounds **48**

8. References **94**

1. General Experimental

All manipulations were carried out using standard Schlenk-line and glovebox techniques under an inert atmosphere of argon or dinitrogen. A MBraun Labmaster glovebox was employed, operating at <0.1 ppm O_2 and <0.1 ppm H_2O. Toluene, hexane, pentane, THF, Et_2O solvents were dried over activated alumina from a SPS (solvent purification system) based upon the Grubbs design, degassed before use, and stored over 3 Å molecular sieves. C_6D_6, C_6H_6, HMDSO, TMS and toluene-d_8 were dried over 3 Å molecular sieves and freeze-pump-thaw degassed three times before use. Glassware was dried for 12 h at 120 °C prior to use. Chemicals were purchased from Sigma Aldrich, Fluorochem, Alfa Aesar, and VWR. Methyl acrylate and 2-vinylpyridine were distilled from CaH_2 under vacuum onto ca. 100 ppm *tert*-butyl catechol stabiliser, degassed via three freeze-pump-thaw cycles, filtered through a column of activated neutral alumina and stored at -35 °C under N_2, away from light. Styrene and styrene-d_8 were distilled from CaH_2 under vacuum onto ca. 100 ppm *tert*-butyl catechol stabiliser, degassed via three freeze-pump-thaw cycles, filtered through a column of activated neutral alumina and stored over activated 3 Å molecular sieves at -35 °C under N_2, away from light. 2-ethynylpyridine, 2-trimethylsilylacetylene and phenylacetylene were distilled from CaH_2, degassed using three freeze-pump-thaw cycles, and stored over 3 Å molecular sieves at -35 °C under N_2, away from light. Chlorodiphenylphosphine was distilled and degassed using three freeze-pump-thaw cycles. Sulphur was recrystallised from toluene and vacuum dried. Column chromatography purifications were carried out using silica gel (tech grades, 60 Å, 230-400 mesh, 40-63 µm particle size).

1 was prepared as reported previously by our group.[1]

NMR Spectra were recorded on Bruker 400 MHz or 500 MHz spectrometers at 298 K unless otherwise stated and values recorded in ppm. Data were processed in MestReNova software. Where needed, chemical shifts were assigned with the assistance of 2D NMR (HSQC, HMBC, COSY) spectra. Elemental analyses were performed by Elemental Labs (https://www.elementallab.co.uk/) or London Metropolitan University (https://www.londonmet.ac.uk/).

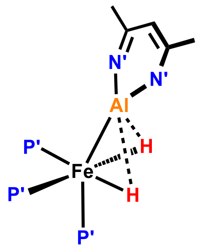

1

Figure S1. Line drawings of complex **1**. P' = PMe_3, N' = N(2,4,6-MeC_6H_2).

2. Synthetic Procedures

*Synthesis of **2a***

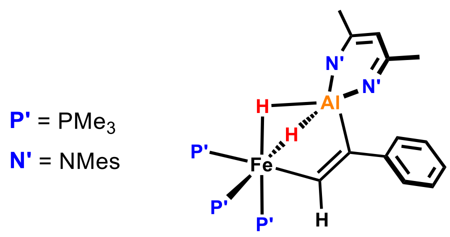

2a

In a glovebox, phenylacetylene (10 µL, 0.09 mmol, 2 equiv.) was added to a solution of **1** (30 mg, 0.046 mmol) in C_6D_6 (0.5 mL) and transferred to a J. Young NMR tube. The colour of the reaction solution immediately changed from dark red-orange to red. NMR analysis of the reaction mixture revealed complete consumption of **1** and formation of **2a** in >95 % yield (based on the relative integrals of in the ^{31}P NMR spectrum). The reaction solution was transferred back to a glovebox, filtered through glass wool and celite and all volatiles were removed under reduced pressure to afford **2a** as a foamy red-orange solid. Isolated yield: 34.5 mg (0.046 mmol, 99 %).

^{1}H NMR (400 MHz, C_6D_6, 298 K): δ 8.98 (td, $^{3}J_{HP}$ = 12.7, 5.3 Hz, 1H, Ph–C(Al)=C(Fe)–***H***), 7.69 (d, *J* = 6.9 Hz, 2H, Ph^{2,6}-C***H***), 7.51 (apparent t, *J* = 7.6 Hz, 2H, Ph^{3,5}-C***H***), 7.09 (t, *J* = 7.3 Hz, 1H, Ph^{4}-C***H***), 6.76 (s, 2H, Mes-C***H***), 6.72 (s, 2H, Mes-C***H***), 5.33 (s, 1H, BDI-C***H***), 2.38 (s, 6H, Mes-C***H***$_3$), 2.27 (s, 6H, Mes-C***H***$_3$), 2.15 (s, 6H, Mes-C***H***$_3$), 1.61 (s, 6H, BDI-C***H***$_3$), 0.99 (m, 18H, P-C***H***$_3$), 0.80 (d, *J* = 5.8 Hz, 9H, P-C***H***$_3$), -14.66 (br, 2H, Fe–***H***–Al).

^{31}P{^{1}H} NMR (162 MHz, C_6D_6, 298 K): δ 29.0 (d, *J* = 31.7 Hz, 2P), 20.5 (t, *J* = 31.7 Hz, 1P).

^{13}C{^{1}H} NMR (101 MHz, C_6D_6, 298 K): δ 183.6 (located from the ^{1}H/^{13}C HMBC spectrum, 1C, Ph–***C***(Al)=C(Fe)–H), 173.7 (br, 1C, Ph–C(Al)=***C***(Fe)–H), 168.6 (2C, BDI-***C***N), 145.6 (2C, Mes-***C*N**), 144.6 (1C, Ph^{1}-***C***) 136.5 (2C, Mes-***C***CH$_3$), 134.1 (2C, Mes-***C***CH$_3$), 132.1 (2C, Mes-***C***CH$_3$), 130.1 (2C, Mes-***C***H), 128.7 (2C, Mes-***C***H), 128.4 (Ph^{3,5}-***C***H, obscured by solvent, located from the ^{1}H/^{13}C HSQC spectrum), 126.1 (2C, Ph^{2,6}-***C***H), 122.3 (2C, Ph^{4}-***C***H), 98.0 (1C, BDI-***C***H), 26.6 (d, *J* = 17.0 Hz, 3C, P-***C***H$_3$), 23.7 (2C, BDI-***C***H$_3$), 23.0 (t, *J* = 10.7, 6C, P-***C***H$_3$), 20.9 (Mes-***C***H$_3$), 20.8 (Mes-***C***H$_3$), 19.6 (Mes-***C***H$_3$).

Anal. Calc ($C_{40}H_{64}AlFeN_2P_3$): C 64.17, H 8.62, N 3.74. Calc ($C_{40}H_{64}AlFeN_2P_3$·3(C_6H_6)): C 71.01, H 8.22, N 2.86. Calc ($C_{40}H_{64}AlFeN_2P_3$·4(C_6H_6)) C 72.65, H 8.10, N 2.65. Found: C 72.87, H 8.05, 3.28. Elemental analysis is most consistent with the isolation of **2a·4(C_6H_6)**.

*Synthesis of **2b***

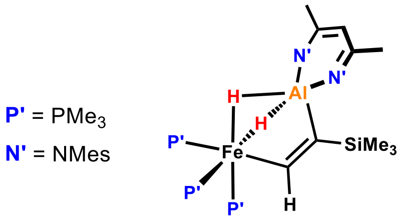

2b

In a glovebox, trimethylsilylacetylene (32 µL, 0.230 mmol) was added to a solution of **1** (15 mg, 0.023 mmol) in C_6D_6 (0.6 mL). The reaction solution immediately turned amber and was transferred to a J. Young NMR tube. NMR analysis of the reaction mixture revealed complete consumption of the **1** and formation of **2c** in >95 % yield (based on the relative integrals of in the ^{31}P NMR spectrum). The reaction solution was transferred back to a glovebox and all volatiles were removed under reduced pressure to afford **2c** as a foamy orange solid. Isolated yield: 17.0 mg (0.023 mmol, 99 %). Crystals suitable for X-ray diffraction were obtained by slow evaporation of a concentrated solution of **2c** in tetramethylsilane.

^{1}H NMR (400 MHz, C_6D_6, 298 K): δ 9.81 (td, $^3J_{HP}$ = 13.3, 8.6 Hz, 1H, Si–C(Al)=C(Fe)–***H***), 6.84 (s, 2H, Mes-C***H***), 6.77 (s, 2H, Mes-C***H***), 5.22 (s, 1H, BDI-C***H***), 2.56 (s, 6H, Mes-C***H***$_3$), 2.23 (s, 6H, Mes-C***H***$_3$), 2.19 (s, 6H, Mes-C***H***$_3$), 1.60 (s, 6H, BDI-C***H***$_3$), 1.00 (m, 18H, P-C***H***$_3$), 0.71 (d, J = 5.6 Hz, 9H, P-C***H***$_3$), 0.42 (s, 9H, Si-C***H***$_3$), -14.49 (bt, $^2J_{HP}$ = 23.3 Hz, 2H, Fe–***H***–Al).

^{31}P{^{1}H} NMR (162 MHz, C_6D_6, 298 K): δ 28.3 (d, J = 27.4 Hz, 2P), 18.9 (t, J = 27.4 Hz, 1P).

^{13}C{^{1}H} NMR (101 MHz, C_6D_6, 298 K): δ 195.7 (m, 1C, Si–C(Al)=***C***(Fe)–H), 193.5 (br, 1C, Si–***C***(Al)=C(Fe)–H), 167.7 (2C, BDI-***C***N), 145.6 (2C, Mes-***C***N), 136.2 (2C, Mes-***C***CH$_3$), 133.6 (2C, Mes-***C***CH$_3$), 131.9 (2C, Mes-***C***CH$_3$), 129.5 (2C, Mes-***C***H), 128.5 (2C, Mes-***C***H), 97.9 (1C, BDI-***C***H), 26.2 (d, J = 15.7 Hz, 3C, P-***C***H$_3$), 23.2 (2C, BDI-***C***H$_3$), 22.4 (t, J = 10.9 Hz, 6C, P-***C***H$_3$), 22.0 (2C, Mes-***C***H$_3$), 20.6 (2C, Mes-***C***H$_3$), 19.3 (2C, Mes-***C***H$_3$), 0.6 (3C, Si-***C***H$_3$).

Anal. Calc. ($C_{37}H_{68}AlFeN_2P_3Si$): C, 59.67; H, 9.20; N, 3.76. Found: C, 60.34; H, 8.63; N, 3.66.

*Synthesis of **2c***

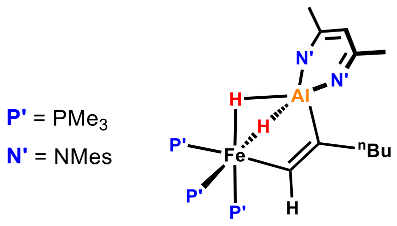

2c

In a glovebox, 1-hexyne (10 µL, 0.09 mmol, ca. 2 equiv.) was added to a solution of **1** (30 mg, 0.046 mmol) in C_6D_6 (0.5 mL) and transferred to a J. Young NMR tube. The colour of the reaction solution immediately changed from dark red-orange to bright orange. NMR analysis of the reaction mixture revealed complete consumption of **1** and formation of **2c** in >95 % yield (based on the relative integrals of in the ^{31}P NMR spectrum). The reaction solution was transferred back to a glovebox and all volatiles were removed under reduced pressure. The orange oily residue was taken up in a minimal amount of benzene, filtered through glass wool and celite, and the volatiles once again removed under reduced pressure to afford **2c·2(C_6H_6)** as a foamy orange solid. Isolated yield: 40.5 mg (0.046 mmol, 99 %).

^{1}H NMR (400 MHz, C_6D_6, 298 K): δ 7.58 (td, $^3J_{HP}$ = 11.9, 5.2 Hz, 1H, Fe–C***H***), 6.86 (s, 2H, Mes–C***H***), 6.79 (s, 2H, Mes–C***H***), 5.18 (s, 1H, BDI–C***H***), 2.74 (br t, *J* = 7.5 Hz, 2H, Al–C–C***H***$_2$), 2.64 (s, 6H, Mes–C***H***$_3$), 2.27 (s, 6H, Mes–C***H***$_3$), 2.20 (s, 6H, Mes–C***H***$_3$), 1.96-1.87 (m, 2H, Al–C–CH$_2$–C***H***$_2$), 1.70 (apparent sextet, *J* = 7.3 Hz, 2H, C***H***$_2$–CH$_3$), 1.62 (s, 6H, BDI–C***H***$_3$), 1.05 – 1.01 (apparent dd, *J* = 3.2 Hz, 18H, P-***C***H$_3$), 0.76 (d, *J* = 5.7 Hz, 9H, P-***C***H$_3$), 1.17 (t, *J* = 7.3 Hz, 3H, CH$_2$–C***H***$_3$), -14.69 (br m, 2H, Fe–***H***–Al).

^{31}P{^{1}H} NMR (162 MHz, C_6D_6, 298 K): δ 29.25 (d, *J* = 30.5 Hz, 2P), 21.86 (t, *J* = 30.5 Hz, 1P).

^{13}C{^{1}H} NMR (101 MHz, C_6D_6, 298 K): δ 187.1 (Al–***C***, found in the HMBC spectrum) 168.0 (2C. BDI–***C***N), 158.1 (Fe–***C***H, found in the HSQC and HMBC spectrum) 146.0 (2C, Mes–***C***N), 136.4 (2C, Mes–***C***N), 134.0 (2C, Mes–***C***N), 132.4 (2C, Mes–***C***N), 129.9 (2C, Mes–***C***H), 128.9 (2C, Mes–***C***H), 97.9 (BDI–***C***H), 47.0 – 46.3 (m, Al–C–***C***H$_2$), 34.0 (Al–C–CH$_2$–***C***H$_2$), 26.85 (d, J = 16.5 Hz, 3C, P-***C***H$_3$), 24.1 (***C***H$_2$–CH$_3$$^)$, 23.6 (2C, BDI–***C***H$_3$), 22.9 (apparent td, *J* = 10.2, 2.5 Hz, 6C, P-***C***H$_3$), 21.5 (2C, Mes–***C***H$_3$), 20.9 (2C, Mes–***C***H$_3$), 19.6 (2C, Mes–***C***H$_3$), 15.0 (CH$_2$–***C***H$_3$).

Anal. Calc. ($C_{38}H_{68}AlFeN_2P_3$): C 62.63, H 9.41, N 3.84. Calc ($C_{50}H_{80}AlFeN_2P_3$): C 68.02, H 8.90, N 3.17. Found: C 68.47, H 9.50, N 3.53. Elemental analysis is consistent with the isolation of **2c·2(C_6H_6)**.

*Synthesis of **2d***

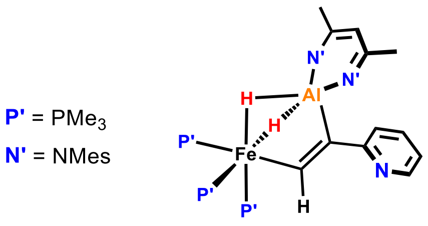

2d

In a glovebox, a stock solution of 2-ethynylpyridine in C_6D_6 (0.1 M, 230 µL, 0.023 mmol) was added to a solution of **1** (15 mg, 0.023 mmol) in C_6D_6 (0.4 mL) and transferred to a J. Young NMR tube. The colour of the reaction solution immediately changed from red-orange to amber. NMR analysis of the reaction mixture revealed complete consumption of **1** and formation of **2d** in >95 % yield (based on the relative integrals of in the ^{31}P NMR spectrum). The reaction solution was transferred back to a glovebox and all volatiles were removed under reduced pressure to afford **2d** as a foamy orange solid. Isolated yield: 16.6 mg (0.022 mmol, 96 %).

^{1}H NMR (400 MHz, C_6D_6, 298 K): δ 10.02 (td, $^{3}J_{HP}$ = 13.4, 5.6 Hz, 1H, Py–C(Al)=C(Fe)–***H***), 8.75 (d, *J* = 4.3, 1H, Py-C***H***), 7.49 (vt, *J* = 7.7, 1H, Py-C***H***), 7.38 (d, *J* = 7.7 Hz, 1H, Py-C***H***), 6.75 (s, 2H, Mes-C***H***), 6.71 (s, 2H, Mes-C***H***), 6.61 (dd, *J* = 7.7, 4.3 Hz, 1H, Py-C***H***), 5.30 (s, 1H, BDI-C***H***), 2.40 (s, 6H, Mes-C***H***$_3$), 2.27 (s, 6H, Mes-C***H***$_3$), 2.14 (s, 6H, Mes-C***H***$_3$), 1.61 (s, 6H, BDI-C***H***$_3$), 1.01 (dd, *J* = 7.6, 4.4 Hz, 18H, P-C***H***$_3$), 0.79 (d, *J* = 5.7 Hz, 9H, P-C***H***$_3$), -14.71 (bvt, *J* = 24.5 Hz, 2H, Fe–***H***–Al).

^{31}P{^{1}H} NMR (162 MHz, C_6D_6, 298 K): δ 29.0 (d, *J* = 33.3 Hz, 2P), 19.9 (t, *J* = 33.3 Hz, 1P).

^{13}C{^{1}H} NMR (101 MHz, C_6D_6, 298 K): δ 188.9 (m, 1C, Py–C(Al)=***C***(Fe)–H), 183.5 (located from the ^{1}H/^{13}C HMBC spectrum, 1C, Py–***C***(Al)=C(Fe)–H), 168.3 (2C, BDI-***C***N), 157.6 (1C, Py^{2}-***C***), 149.7 (1C, Py-***C***H), 145.2 (2C, Mes-***C***N), 136.1 (2C, Mes-***C***CH$_3$), 134.9 (1C, Py-***C***H), 133.8 (2C, Mes-***C***CH$_3$), 131.8 (2C, Mes-***C***CH$_3$), 129.8 (2C, Mes-***C***H), 128.5 (2C, Mes-***C***H), 123.4 (1C, Py-***C***H), 116.4 (1C, Py-***C***H), 97.5 (1C, BDI-***C***H), 26.1 (d, *J* = 17.0 Hz, 3C, P-***C***H$_3$), 23.5 (2C, BDI-***C***H$_3$), 22.7 (t, *J* = 10.9 Hz, 6C, P-***C***H$_3$), 20.5 (2C, Mes-***C***H$_3$), 20.3 (2C, BDI-***C***H$_3$), 19.3 (2C, Mes-***C***H$_3$).

Anal. Calc. ($C_{39}H_{63}AlFeN_3P_3$): C, 62.48; H, 8.47; N, 5.61. Found: C, 62.18; H, 8.35; N, 5.55.

Synthesis of **S1**

P' = PMe_3
N' = NMes

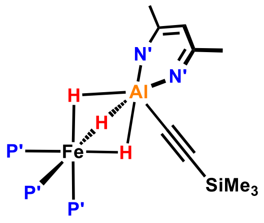

S1

In a glovebox, a solution of **2b** (32.4 mg, 0.0353 mmol) in benzene (ca. 0.5 mL) was transferred to a J. Youngs NMR tube. The mixture was heated to 80 °C overnight by when NMR analysis showed a complete consumption of **2b** and exclusive formation of **S1**. The NMR tube was cycled back into the glovebox, the solution decanted, and the volatiles evaporated under vacuum. The resulting yellow solid was washed with small amounts (ca. 3 x 0.2 mL) of cold (-35 °C) TMS, then dried under vacuum. Yield: 31.1 mg, 0.339 mmol, 96 %, yellow solid. Crystals suitable for X-ray diffraction could be grown from a concentrated solution of **S1** in pentane at -35 °C over a week.

^{1}H NMR (400 MHz, C_6D_6, 298 K): δ 6.8 (s, 2H, Mes-C***H***), 6.75 (s, 2H, Mes-C***H***), 5.23 (s, 1H, BDI-C***H***), 2.83 (s, 6H, Mes-C***H***$_3$), 2.33 (s, 6H, Mes-C***H***$_3$), 2.20 (s, 6H, Mes-C***H***$_3$), 1.50 (s, 6H, BDI-C***H***$_3$), 0.97 (m, 27 H, P-C***H***$_3$), -15.81 (br m, 3H, Fe–***H***–Al).

^{31}P{^{1}H} NMR (162 MHz, C_6D_6, 298 K): δ 29.17 (s, 3P).

^{13}C{^{1}H} NMR (101 MHz, C_6D_6, 298 K): δ 167.6 (2C, BDI-***C***N), 146.4 (2C, Mes-***C***N), 136.8 (2C, Mes-***C***CH$_3$), 134.2 (2C, Mes-***C***CH$_3$), 133.4 (2C, Mes-***C***CH$_3$), 130.1 (2C, Mes-***C***H), 129.1 (2C, Mes-***C***H), 108.2 (1C, AlC***C***Si), 100.0 (1C, BDI-***C***H), 26.4 (m, 9C, P-***C***H$_3$), 24.0 (2C, BDI-***C***H$_3$), 21.3 (2C, Mes-***C***H$_3$), 20.9 (2C, BDI-***C***H$_3$), 20.1 (2C, Mes-***C***H$_3$), 1.3 (3C, Si-***C***H$_3$). The Al–***C***–CSi resonance was not observed.

*Synthesis of **3c***

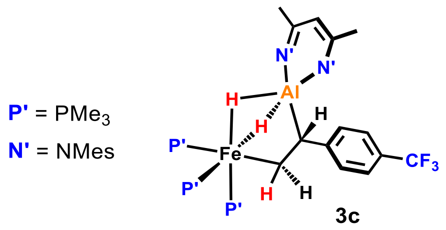

Crystallisation: In a glovebox, a solution of **1** (20 mg, 0.031 mmol) in *n*-pentane (1.0 mL) was cooled to -35 °C inside the glovebox freezer. 4-(trifluoromethyl)styrene (6.9 µL, 0.047 mmol) was added to the cold solution using a micro syringe. The solution immediately turned from dark to bright orange and kept at -35 °C. Orange crystals suitable for X-ray diffraction were obtained over 3 days at -35 °C.

Spectroscopic characterisation: In a glovebox, a solution of **1** (20 mg, 0.031 mmol) in toluene-d_8 (0.5 mL) was transferred to a J. Young NMR tube and cooled to -35 °C inside the glovebox freezer. 4-(trifluoromethyl)styrene (6.9 µL, 0.047 mmol) was added to the cold solution using a micro pipette. The NMR tube was quickly sealed, shaken, and removed from the glovebox. The sample was kept in liquid nitrogen till the NMR tube was inserted into the pre-cooled (-35 °C) spectrometer probe. 3c was formed in >99 % NMR yield.

^{1}H NMR (400 MHz, toluene-d_8, 238 K): δ 7.68 (d, *J* = 8.3 Hz, 2H, Ar-C***H***), 7.59 (d, *J* = 8.3 Hz, 2H, Ar-C***H***), 7.10 (s, 1H, Mes-C***H***), 6.87 (s, 1H, Mes-C***H***), 6.76 (s, 1H, Mes-C***H***), 6.75 (s, 1H, Mes-C***H***), 5.07 (s, 1H, BDI-C***H***), 2.84 (s, 3H, Mes-C***H***$_3$), 2.74 (br m, 1H, Ar–C***H***(Al)–CH$_2$(Fe)), 2.25 (s, 6H, Mes-C***H***$_3$), 2.21 (s, 3H, Mes-C***H***$_3$), 2.18 (s, 3H, Mes-C***H***$_3$), 1.90 (s, 3H, Mes-C***H***$_3$), 1.68 (s, 3H, BDI-C***H***$_3$), 1.24 (s, 3H, BDI-C***H***$_3$), 1.15 (d, *J* = 6.0 Hz, 9H, P-C***H***$_3$), 1.03 (br, 1H, Ar–CH(Al)–C***H***$_2$(Fe), located from the ^{1}H/^{13}C HSQC spectrum), 0.85 (d, *J* = 6.2 Hz, 9H, P-C***H***$_3$), 0.71 (d, *J* = 5.5 Hz, 9H, P-C***H***$_3$), 0.64 (br, 1H, Ar–CH(Al)–C***H***$_2$(Fe)), -14.92 (br t, *J* = 23.9 Hz, 1H, Fe–***H***–Al), -15.93 (br t, *J* = 27.8 Hz, 1H, Fe–***H***–Al).

^{31}P{^{1}H} NMR (162 MHz, toluene-d_8, 238 K): δ 36.5 (***AB***X m, J_{AB} = 41.5 Hz, 2P), 22.2 (AB***X*** m, 1P).

^{19}F{^{1}H} NMR (377 MHz, toluene-d_8, 238 K): δ -59.7 (s, 3F).

^{13}C{^{1}H} NMR (101 MHz toluene-d_8, 238 K): δ 169.1 (1C, BDI-**C**N), 168.1 (1C, BDI-**C**N), 161.3 (Ar-**C**), 144.7 (1C, Mes-**C**N), 144.7 (1C, Mes-**C**N), 135.5 (1C, Mes-**C**CH$_3$), 135.0 (1C, Mes-**C**CH$_3$), 134.5 (1C, Mes-**C**CH$_3$), 134.1 (1C, Mes-**C**CH$_3$), 134.1 (1C, Mes-**C**CH$_3$), 133.5 (1C, Mes-**C**CH$_3$), 129.7 (1C, Mes-**C**H), 129.1 (1C, Mes-**C**H), 129.0 (1C, Mes-**C**H), 128.6 (1C, Mes-**C**H), 126.2 (2C, Ar-**C**H), 123.6 (q, $^3J_{CF}$ = 3.5 Hz, 2C, Ar-**C**H), 120.7 (q, $^2J_{CF}$ = 30.8 Hz, 1C, Ar-**C**CF$_3$), 100.0 (1C, BDI-**C**H), 52.0 (br, 1C, Ar–**C**H(Al)–CH$_2$(Fe)), 26.2 (d, $^1J_{CP}$ = 17.3 Hz, 3C, P-**C**H$_3$), 24.2 (3C, BDI-**C**H$_3$), 23.0 (3C, BDI-**C**H$_3$), 22.6 (d, $^1J_{CP}$ = 18.2 Hz, 3C, P-**C**H$_3$), 22.2 (3C, Mes-**C**H$_3$), 21.7 (d, $^1J_{CP}$ = 17.7 Hz, 3C, P-**C**H$_3$), 21.2 (3C, Mes-**C**H$_3$), 20.7 (3C, Mes-**C**H$_3$), 20.7 (3C, Mes-**C**H$_3$), 19.8 (3C, Mes-**C**H$_3$), 18.9 (3C, Mes-**C**H$_3$), -0.7 (br, 1C, Ar–CH(Al)–**C**H$_2$(Fe)). The Ar-C**C**F$_3$ resonance could not be observed.

The instability of **3c** at room temperature prevented its characterisation by elemental analysis.

*Synthesis of **4a***

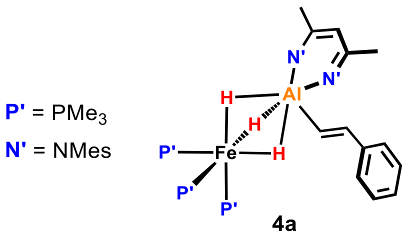

4a

In a glovebox, a stock solution of styrene in C_6D_6 (0.1 M, 230 µL, 0.023 mmol) was added to a solution of **1** (15 mg, 0.023 mmol) in C_6D_6 (0.4 mL). The solution was transferred to a J. Youngs NMR tube and left at room temperature. Continuous monitoring of the reaction by NMR spectroscopy showed slow conversion of the starting materials into **4a**. After 14 d, **4a** had been formed in 78 % NMR yield (based on the relative integrals of in the ^{31}P NMR spectrum). The orange reaction solution was transferred back to a glovebox and the solvent was removed under reduced pressure. The residue was washed with cold tetramethylsilane (ca. 3 x 0.5 mL) and cold *n*-pentane (ca. 2 x 0.3 mL) to afford an off-white solid which was dried under vacuum. Isolated yield: 9.5 mg (0.013 mmol, 55 %). Pale yellow needle crystals suitable for X-ray diffraction could be grown by vapour diffusion of HMDSO into a concentrated solution of **4a** in toluene.

Alternatively, a solution of **1** (20.0 mg, 0.031 mmol) and styrene (7.1 µL, 0.062 mmol) in C_6D_6 (0.5 mL) was heated to 40 °C for 5 d. All volatiles were removed under reduced pressure, the solid residue washed with cold *n*-pentane (2 x 0.5 mL) and dried under high vacuum. Isolated yield: 18.0 mg (0.024 mmol, 77 %).

^{1}H NMR (400 MHz, C_6D_6, 298 K): δ 7.86 (d, *J* = 19.9 Hz, 1H, Ph–CH=C***H***–Al), 7.77 (d, *J* = 7.6 Hz, 2H, Ph-C***H***), 7.34 (d, *J* = 19.9 Hz, 1H, Ph–C***H***=CH–Al), 7.29 (dd, *J* = 7.6, 7.4 Hz, 2H, Ph-C***H***), 7.07 (t, *J* = 7.4 Hz, 1H, Ph-C***H***), 6.78 (s, 2H, Mes-C***H***), 6.77 (s, 2H, Mes-C***H***), 5.25 (s, 1H, BDI-C***H***), 2.49 (s, 6H, Mes-C***H***$_3$), 2.41 (s, 6H, Mes-C***H***$_3$), 2.18 (s, 6H, Mes-C***H***$_3$), 1.54 (s, 6H, BDI-C***H***$_3$), 0.94 (s, 27H, P-C***H***$_3$), -15.58 (bq, $^{2}J_{HP}$ = 19.5 Hz, 3H, Fe–***H***–Al).

^{31}P{^{1}H} NMR (162 MHz, C_6D_6, 298 K): δ 29.0 (s, 3P).

^{13}C{^{1}H} NMR (101 MHz, C_6D_6, 298 K): δ 167.3 (2C, BDI-***C***N), 155.0 (located from the ^{1}H/^{13}C HSQC and ^{1}H/^{13}C HMBC spectra, 1C, Ph–CH=***C***H–Al) 146.2 (2C, Mes-***C***N), 142.9 (1C, Ph^{1}-***C***), 141.0 (1C, Ph–***C***H=CH–Al), 135.6 (2C, Mes-***C***CH$_3$), 133.7 (2C, Mes-***C***CH$_3$), 133.0 (2C, Mes-***C***CH$_3$), 129.8 (2C, Mes-***C***H), 128.9 (2C, Mes-***C***H), 128.3 (2C, Ph-***C***H), 125.8 (2C, Ph-***C***H), 125.4 (1C, Ph-***C***H), 99.7 (1C, BDI-***C***H), 26.0 (m, 9C, P-***C***H$_3$), 23.8 (2C, BDI-***C***H$_3$), 20.9 (2C, Mes-***C***H$_3$), 20.5 (2C, Mes-***C***H$_3$), 19.9 (2C, Mes-***C***H$_3$).

Anal. Calc. ($C_{40}H_{66}AlFeN_2P_3$): C,64.00; H, 8.86; N, 3.73. Found: C, 64.30; H, 8.68; N, 3.55.

*Synthesis of **4b***

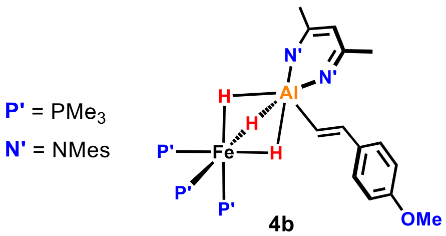

4b

In a glovebox, styrene (8.3 µL, 0.062 mmol) was added to a solution of **1** (20 mg, 0.031 mmol) in C_6D_6 (0.5 mL). The solution was transferred to a J. Youngs NMR tube and heated to 40 °C for 5 d. The reaction solution was transferred back to a glovebox and the solvent was removed under reduced pressure. The residue was washed with cold *n*-pentane (4 x 0.5 mL) to afford a pale orange solid which was dried under vacuum. Isolated yield: 18.0 mg (0.023 mmol, 74 %).

^{1}H NMR (400 MHz, C_6D_6, 298 K): δ 7.70 (d, *J* = 8.7 Hz, 2H, Ar-C***H***), 7.69 (d, *J* = 19.9 Hz, 1H, Ar–CH=C***H***–Al), 7.31 (d, *J* = 19.9 Hz, 1H, Ar–C***H***=CH–Al), 6.91 (d, *J* = 8.7 Hz, 2H, Ar-C***H***), 6.79 (s, 2H, Mes-C***H***), 6.78 (s, 2H, Mes-C***H***), 5.26 (s, 1H, BDI-C***H***), 3.33 (s, 3H, Ar-OC***H***$_3$), 2.52 (s, 6H, Mes-C***H***$_3$), 2.42 (s, 6H, Mes-C***H***$_3$), 2.19 (s, 6H, Mes-C***H***$_3$), 1.55 (s, 6H, BDI-C***H***$_3$), 0.96 (s, 27H, P-C***H***$_3$), -15.56 (bq, $^2J_{HP}$ = 20.9 Hz, 3H, Fe–***H***–Al).

^{31}P{^{1}H} NMR (162 MHz, C_6D_6, 298 K): δ 29.0 (s, 3P).

^{13}C{^{1}H} NMR (101 MHz, C_6D_6, 298 K): δ 167.3 (2C, BDI-***C***N), 158.3 (1C, Ar-***C***OCH$_3$), 152.1 (br, 1C, Ar–CH=***C***H–Al), 146.3 (2C, Mes-***C***N), 140.4 (1C, Ar–***C***H=CH–Al), 136.1 (1C, Ar-***C***), 135.6 (2C, Mes-***C***CH$_3$), 133.7 (2C, Mes-***C***CH$_3$), 133.0 (2C, Mes-***C***CH$_3$), 129.8 (2C, Mes-***C***H), 128.9 (2C, Mes-***C***H), 126.7 (Ar-***C***H), 113.9 (Ar-***C***H), 99.7 (BDI-***C***H), 54.5 (1C, Ar-CO***C***H$_3$), 26.1 (m, 9C, P-***C***H$_3$), 23.8 (2C, BDI-***C***H$_3$), 21.0 (2C, Mes-***C***H$_3$), 20.5 (2C, Mes-***C***H$_3$), 19.9 (2C, Mes-***C***H$_3$).

Anal. Calc. ($C_{41}H_{68}AlFeN_2OP_3$): C, 63.07; H, 8.78; N, 3.59. Found: C, 63.88; H, 7.63; N, 2.83.

*Synthesis of **4c***

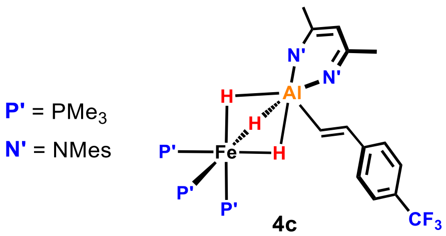

4c

In a glovebox, a stock solution of 4-(trifluoromethyl)styrene in C_6D_6 (0.1 M, 310 µL, 0.031 mmol) was added to a solution of **1** (20 mg, 0.031 mmol) in C_6D_6 (0.4 mL) and transferred to a J. Youngs NMR tube. The reaction solution immediately became brighter, and the colour changed from dark orange to yellow within 1 h. NMR analysis of the reaction mixture after 3 h revealed complete consumption of **1** and formation of **4c** in >95 % yield (based on the relative integrals of in the ^{31}P NMR spectrum). The reaction solution was transferred back to a glovebox and the solvent was removed under reduced pressure. The resulting solid was washed with small amounts (3 x 0.5 ml) of cold *n*-pentane and dried under vacuum to afford a pale orange solid. Isolated yield: 19.8 mg (0.024 mmol, 77 %).

^{1}H NMR (400 MHz, C_6D_6, 298 K): δ ^{1}H NMR (400 MHz, C_6D_6) δ 7.93 (d, *J* = 19.9 Hz, 1H, Ph–CH=C***H***–Al), 7.56 (d, *J* = 8.1 Hz, 2H, Ar-C***H***), 7.45 (d, *J* = 8.1 Hz, 2H, Ar-C***H***), 7.25 (d, *J* = 19.9 Hz, 1H, Ar–C***H***=CH–Al), 6.80 (s, 2H, Mes-C***H***), 6.78 (s, 2H, Mes-C***H***), 5.23 (s, 1H, BDI-C***H***), 2.45 (s, 6H, Mes-C***H***$_3$), 2.39 (s, 6H, Mes-C***H***$_3$), 2.19 (s, 6H, Mes-C***H***$_3$), 1.53 (s, 6H, BDI-C***H***$_3$), 0.93 (s, 27H, P-C***H***$_3$), -15.64 (bq, *J* = 21.0 Hz, 3H, Fe–***H***–Al).

^{31}P{^{1}H} NMR (162 MHz, C_6D_6, 298 K): δ 28.8 (s, 3P).

^{19}F{^{1}H} NMR (377 MHz, C_6D_6, 298 K) δ -61.6 (s, 3F).

^{13}C{^{1}H} NMR (101 MHz, C_6D_6, 298 K): δ 167.5 (2C, BDI-***C***N), 159.9 (br, 1C, Ar–CH=***C***H–Al), 146.0 (2C, Mes-***C***N), 145.9 (Ar-***C***), 139.3 (1C, Ar–***C***H=CH–Al), 135.4 (2C, Mes-***C***CH$_3$), 133.9 (2C, Mes-***C***CH$_3$), 133.0 (2C, Mes-***C***CH$_3$), 129.8 (2C, Mes-***C***H), 129.0 (2C, Mes-***C***H), 127.0 (q, $^{2}J_{CF}$ = 32.0 Hz, 1C, Ar-***C***CF$_3$), 125.7 (2C, Ar-***C***H), 125.3 (q, $^{1}J_{CF}$ = 271.4 Hz, 1C, Ar-C***C***F$_3$), 125.3 (q, $^{3}J_{CF}$ = 3.9 Hz, 2C, Ar-***C***H), 99.6 (1C, BDI-***C***H), 26.0 (m, 9C, P-***C***H$_3$), 23.7 (2C, BDI-***C***H$_3$), 20.8 (2C, Mes-***C***H$_3$), 20.5 (2C, Mes-***C***H$_3$), 19.9 (2C, Mes-***C***H$_3$).

Anal. Calc. ($C_{41}H_{65}AlF_3FeN_2P_3$): C, 60.15; H, 8.00; N, 3.42. Found: C, 60.57; H, 8.26; N, 3.12.

*Lewis-acid assisted reaction of **1** with 2-vinylpyridine: synthesis of **4d***

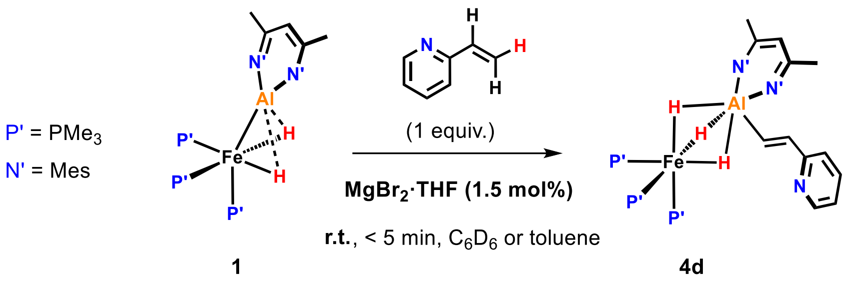

In a glovebox, a stock solution of $MgBr_2{\cdot}OEt_2$ in THF (2.32 M, 15 µL, 0.00035 mmol, 1.5 mol%) was added to **1** (15 mg, 0.023 mmol) and the mixture was placed under vacuum for 5 minutes. C_6D_6 (ca. 0.5 mL) was added, and the solution was lightly shaken while a solution of 2-vinylpyridine (5.0 µL, 0.046 mmol, 2equiv.) in toluene (ca. 1.0 mL) was added dropwise over the course of ca. 30 s resulting in a colour change from dark red to brownish yellow. The reaction mixture was transferred to a J. Youngs NMR tube, where NMR analysis of the mixture showed complete consumption of **1** and the formation of **4d** in >95 % yield. The NMR tube was cycled back into the glovebox, the volatiles were removed under vacuum, then the dark yellow residue was repeatedly suspended in pentane (ca. 3x1 mL) and the volatiles were removed under vacuum. The mixture was taken up in toluene (ca. 1.0 mL), filtered through glass wool and celite, then the volatiles removed under vacuum once more to yield crude **4d** as a dark yellow powder (13.4 mg, 0.018 mmol, 77 %), judged to be >95 % pure by NMR. Crystals suitable for X-ray diffraction and samples of analytical purity could be obtained by slow evaporation of a concentrated solution of **4d** in diethyl ether.

Analogous reactivity was observed when toluene (ca. 1.0 mL) was used as a reaction solvent instead of C_6D_6.

^{1}H NMR (400 MHz, C_6D_6, 298 K): δ 8.61 (d, *J* = 4.7 Hz, 1H, Py-C***H***), 8.27 (d, *J* = 20.2 Hz, 1H, Py–CH=C***H***–Al), 7.71 (d, *J* = 8.1 Hz, 1H, Py-C***H***), 7.66 (d, *J* = 20.2 Hz, 1H, Py–C***H***=CH_2–Al), 7.18 (d, *J* = 7.8 Hz, 1H, Py-C***H***), 6.77 (s, 4H, Mes-C***H***), 6.58 (dd, *J* = 7.8, 4.7 Hz, 1H, Py-C***H***), 5.25 (s, 1H, BDI-C***H***), 2.51 (s, 6H, Mes-C$\boldsymbol{H}_3$), 2.40 (s, 6H, Mes-C$\boldsymbol{H}_3$), 2.17 (s, 6H, Mes-C$\boldsymbol{H}_3$), 1.53 (s, 6H, BDI-C$\boldsymbol{H}_3$), 0.95 (s, 27H, P-C$\boldsymbol{H}_3$), -15.56 (bq, *J* = 20.5 Hz, 3H, Fe–***H***–Al).

^{31}P{^{1}H} NMR (162 MHz, C_6D_6, 298 K): δ 28.9 (s, 3P).

^{13}C{^{1}H} NMR (101 MHz, C_6D_6, 298 K): δ 167.4 (2C, BDI-***C***N), 160.7 (1C, Py^2-***C***), 160.5 (br, 1C, Py–CH=***C***H–Al), 149.3 (1C, Py-***C***H), 146.2 (2C, Mes-***C***N), 142.7 (1C, Py–***C***H=CH_2–Al), 135.7 (2C, Mes-***C***CH_3), 135.0 (1C, Py-***C***H), 133.7 (2C, Mes-***C***CH_3), 133.0 (2C, Mes-***C***CH_3), 129.8 (2C, Mes-***C***H), 128.9 (2C, Mes-***C***H), 119.8 (1C, Py-***C***H), 118.5 (1C, Py-***C***H), 99.8 (1C, BDI-***C***H), 26.0 (m, 9C, P-***C***H_3), 23.8 (2C, BDI-***C***H_3), 21.0 (2C, Mes-***C***H_3), 20.5 (2C, Mes-***C***H_3), 19.9 (2C, Mes-***C***H_3).

Anal. Calc. ($C_{39}H_{65}AlFeN_3P_3$): C, 62.31; H, 8.72; N, 5.59. Found: C, 62.18; H, 8.35; N, 5.55.

*Low temperature reaction of 1 with 2-vinylpyridine: synthesis of **5d***

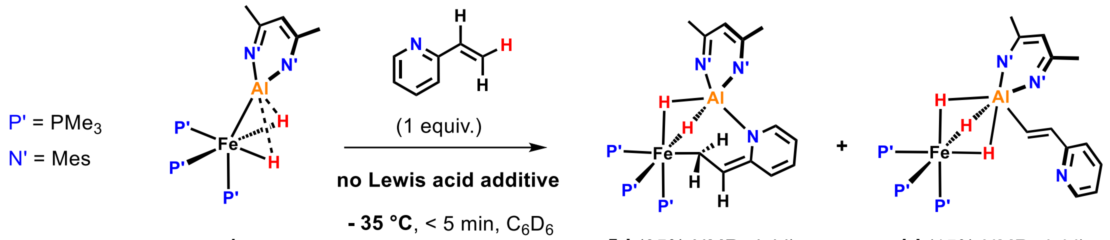

1 **5d** (85% NMR yield) **4d** (15% NMR yield)

Spectroscopic characterisation: In a glovebox, a solution of **1** (5 mg, 0.0077 mmol) in toluene-d_8 (0.5 mL) was transferred to a J. Youngs NMR tube and cooled to -35 °C inside the glovebox freezer. 2-vinylpyridine (2.75 µL, 0.025 mmol, 3.3 equiv.) was added to the cold solution using a micro pipette, resulting in a colour change from dark red orange to greenish brown. The NMR tube was quickly sealed, shaken, and removed from the glovebox. The sample was kept in liquid nitrogen till the NMR tube was inserted into the pre-cooled (-40 °C) spectrometer probe. NMR characterisation of the sample showed a complete consumption of **1**, with the product mixture consisting of **5d** (85 %) and **4d** (15 %).

Spectroscopic Data for 5d:

^{1}H NMR (400 MHz, toluene-d_8, 233 K): δ 7.62 (d, J = 7.1 Hz, 1H, Py-C***H***), 6f.76 (s, Mes-***C***H), 6.66 (overlapped s, Mes-***C***H), 6.62 (overlapped s, Mes-***C***H), 6.55 (s, Mes-***C***H), 6.25 (d, J = 9.2 Hz, 1H, Py-C***H***), 6.02 (dd, J = 9.2, 5.4 Hz, Py-C***H***), 5.24 (d, J = 1.8 Hz, 1H, Py-C***H***), 5.07 (s, 1H, BDI–C***H***), 4.55 (t, J = 8.2 Hz, 1H, Py–C***H***=CH_2–Fe), 3.06 (s, 3H, C***H***$_3$), 2.34 (s, 3H, C***H***$_3$), 2.29 (s, 3H, C***H***$_3$), 2.16 (s, 3H, C***H***$_3$), 2.12 (s, 6H, C***H***$_3$), 1.66 (br m, J = 8.2 Hz, 1H, Py–CH=C***H***^{A}H^B–Fe), 1.55(s, 3H, C***H***$_3$), 1.32 (s, 3H, C***H***$_3$), 1.08 (br s, 9H, P-C***H***$_3$), 1.01 (d, J = 6.6 Hz, 3H, P-C***H***$_3$), 0.77 (d, J = 5.0 Hz, 3H, P-C***H***$_3$), 0.60 (br d, J = 4.7 Hz, 9H, P-C***H***$_3$), 0.04 (d, J = 6.1 Hz, 3H, P-C***H***$_3$), -0.02 (br m, 1H, Py–CH=CH^A***H***^{B}–Fe), -15.75 (br m, 1H, , Fe–***H***–Al), -17.16 (br m, 1H, , Fe–***H***–Al).

^{31}P{^{1}H} NMR (162 MHz, toluene-d_8, 233 K): δ 27.90 (m, 2P), 23.45 (t, $^2J_{PP}$ = 24.8 Hz).

^{13}C{^{1}H} NMR (101 MHz, toluene-d_8, 233 K): δ 169.8 (BDI–***C***N), 169.2 (BDI–***C***N), 147.0 (Mes–***C***N), 145.3 (Mes–***C***N), 145.2 (Py–***C***H), 143.61 (Py–***C***H), 136.7 (Mes–***C***CH$_3$), 135.1 (Mes–***C***CH$_3$), 135.0 (Mes–***C***CH$_3$), 134.9 (Mes–***C***CH$_3$), 132.3 (Mes–***C***H$_3$), 131.4 (Mes–***C***H$_3$), 130.2 (Mes–***C***H$_3$), 129.8 (Mes–***C***H$_3$), 125.0 (Py^2–***C***), 122.9 (Py–***C***H), 102.27 (Py–***C***H–CH_2–Fe), 101.44 BDI–***C***H), 98.04 (Py–***C***H), 30.8 (m, P-***C***H$_3$), 25.7 (m, P-***C***H$_3$), 25.4 (***C***H$_3$), 24.6 (***C***H$_3$), 24.5 (***C***H$_3$), 23.9 (m, P-***C***H$_3$), 22.2 (m, P-***C***H$_3$), 21.7 (m, P-***C***H$_3$), 21.4 (overlapped ***C***H$_3$), 21.1 (overlapped ***C***H$_3$), 20.7 (overlapped ***C***H$_3$), 20.6 (overlapped ***C***H$_3$), 19.2 (***C***H$_3$), -11.8 (Py–CH–***C***H$_2$–Fe).

Due to the instability of **5d** elemental analysis could not be performed.

*Additional notes on the reactions of **1** with 2-vinylpyridine*

- Warming the solution of **5d** (prepared at -35 °C as described above) to room temperature results in the loss of the NMR signals associated with **5d** within 1 h. Analogously to **5e** (*vide infra*), **5d** is assumed to decompose to an aluminacycle and range of presumed unidentified Fe(II) dihydride complexes, as well as other minor decomposition products.

- Attempts to crystallise **5d** or any of the decomposition products from cold solutions of various solvent mixtures (*n*-pentane, Et_2O, HMDSO, toluene) proved unsuccessful.

- Reactions of **1** with 2-vinylpyridine at room temperature (without the addition of Lewis acid catalyst) resulted in an immediate colour change to greenish brown which slowly turned red over the course of several hours. NMR monitoring revealed the formation of a mixture of products, mainly consisting of varying ratios of **5d** that rapidly decomposes, minor amounts of **4d**, as well unidentified products. The solvent system used (benzene, toluene, pentane, Et_2O), the concentrations of the reactants, rates and order of addition, did not significantly alter the ratio of the products or the reproducibility of the reaction.

- Various other Lewis acids (eg. LiCl, $[Et_3NH]Cl$, $[NH_4][PF_6]$) were also tested but proved ineffective as catalysts. Sufficiently strong Brønsted acids (eg. benzoic acid) also catalyse the reaction, but do not lead to clean product formation due to competitive O–H activation and protolysis.

- Apart from the temperature and the use of Lewis acid catalysis the purity of the 2-vinylpyridine substrate was found also be important for these reactions. Old samples of 2-vinylpyridine stored over 4 Å molecular sieves developed a faint yellow colour even when stored in the freezer. Though no impurities could be detected by NMR or GC, these samples generally gave less reliable reactions, with the product mixture containing more unidentifiable side-products.

*The reaction of **1** with methyl acrylate*

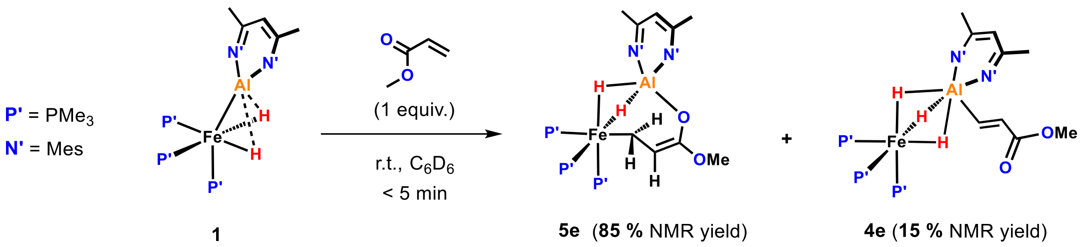

To a solution of **1** (30 mg, 0.0464 mmol) in C_6D_6 (0.5 mL) in a J. Youngs NMR tube was added methyl acrylate (8.4 µL, 0.87 mmol, 2 equiv.) and an immediate colour change to bright orange was observed. NMR monitoring revealed complete consumption of **1** and exclusive formation of **5e** in 85 %, and **4e** in 15 % NMR yield. NB: Attempts at a $MgBr_2$-catalysed reaction *via* an analogous procedure to **4d** (*vide supra*) resulted in no apparent change to the ratio of the products.

*Isolation of **5e***

The NMR tube was cycled back into the glovebox and the volatiles were removed under vacuum. The orange solid was extracted with pentane (ca. 3x3 mL), the combined extracts were filtered through glass wool and celite and concentrated to a ca. 2 mL volume. Storage of the orange solution at -35 °C overnight gave crystals of **5e** suitable for X-ray diffraction. The crystals were washed with small amounts of cold (-35 °C) pentane (ca. 3x0.5 mL) and dried under vacuum to yield **5e** as orange crystals (20.4 mg, 0.0278 mmol, 60 %).

*Isolation of **4e**:* Repeatedly (2x) concentrating the mother liqueur and crystallising at -35 °C, then discarding the resulting yellow-orange solid – a co-crystallised mixture of **5e** and **4e** – gave <0.2 mg of yellow and orange crystals. This mixture was found to be sufficiently enriched in **4e**, that manual removal of the orange crystals from among the yellow microcrystals then drying under vacuum yielded **4e** as a yellow microcrystalline solid (< 0.1 mg, < 1 %).

*Spectroscopic data for **5e***

^{1}H NMR (500 MHz, C_6D_6, 298 K): δ 6.86 (s, 2H, Mes–C***H***), 6.73 (s, 2H, Mes–C***H***), 5.30 (s, 1H, BDI–C***H***), 3.82 (t, J = 6.7 Hz, 1H, C***H***–CH_2–Fe), 3.64 (s, 3H, OC***H***$_3$), 2.64 (br s, 6H, Mes–C***H***$_3$, FWHM = 192.5 Hz), 2.32 (br s, 6H, Mes–C***H***$_3$, FWHM = 54 Hz), 2.19 (s, 6H, Mes–C***H***$_3$), 1.58 (s, 6H, BDI–C***H***$_3$), 0.84 (br s, 18H, P-***C***H_3, FWHM = 260 Hz), 0.72 (br s, 9H, P-***C***H_3, FWHM = 34.5 Hz), -16.06 (br s, 1H, Fe–***H***–Al, FWHM = 203 Hz), -16.51 (br s, 1H, Fe–***H***–Al, FWHM = 212 Hz). The CH–C***H***$_2$–Fe was not observed likely due overlapping with the broadened P–C***H***$_3$ resonances.

^{31}P{^{1}H} NMR (202 MHz, C_6D_6, 298 K): δ 29.54 (br s, 1P, FWHM = 195.5 Hz), 26.57 (br s, 1P, FWHM = 209.5 Hz), 23.09 (br t, J = 28.0 Hz, 1P, FWHM = 10.5 Hz).

^{13}C{^{1}H} NMR (126 MHz, C_6D_6, 298 K): δ 168.3 (2C, BDI–***C***N), 161.3 (***C***–CH–CH_2–Fe), 145.5 (br s, 2C, Mes–***C***N), 137.0 (2C, Mes–***C***CH_3), 134.4 (2C, Mes–***C***CH_3), 130.2 (br s, 2C, Mes–***C***H), 129.1 (2C, Mes–***C***H), 100.1 (BDI–***C***H),

74.4 (C–**C**H–CH_2–Fe), 53.2 (O**C**H_3), 26.4 (br s, 3C, P-**C**H_3), 24.16 (br s, 2C, Mes–**C**H_3), 22.5 (br s, 6C, P-**C**H_3), 20.9 (2C, BDI–**C**H_3), 20.1 (br s, 2C, Mes–**C**H_3) -13.27 (td, J = 12.4, 4.5 Hz, CH–**C**H_2–Fe).

Elemental analysis could not be performed due to **5e**'s instability at room temperature.

*Spectroscopic data for **4e***

^{1}H NMR (400 MHz, C_6D_6, 298 K): δ 8.75 (d, *J* = 20.0 Hz, 1H, CH–C***H***–Al), 6.90 (d, *J* = 20.2 Hz, 1H, C***H***–CH–Al), 6.75 (s, 4H, Mes–C***H***), 5.17 (s, 1H, BDI–C***H***), 3.56 (s, 3H, OC***H***$_3$), 2.46 (s, 6H, C***H***$_3$), 2.35 (s, 6H, C***H***$_3$), 2.16 (s, 6H, C***H***$_3$), 1.46 (s, 6H, C***H***$_3$), 0.90 (br m, 27H, P–C***H***$_3$), -15.68 (br m, 3H, Fe–***H***–Al).

^{31}P{^{1}H} NMR (162 MHz, C_6D_6, 298 K): δ 28.66 (s, 3P).

^{13}C{^{1}H} NMR and elemental analysis could not be performed due to **4e**'s low yield and its instability at room temperature.

*Isolation of **S2**:*

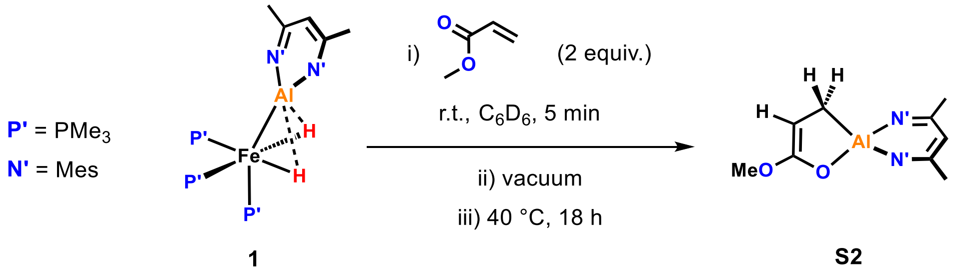

To a solution of **1** (30 mg, 0.0464 mmol) in C_6H_6 (0.5 mL) was added methyl acrylate (8.4 µL, 0.87 mmol, 2 equiv.) and an immediate colour change to bright orange was observed. After 5 min, the volatiles were evaporated under vacuum, and the bright orange residue was dissolved in C_6D_6 and transferred to a J. Youngs NMR tube. The mixture was heated to 40 °C for 18 h resulting in a colour change to brown. The NMR tube was cycled back into the glovebox and the volatiles were removed under vacuum. The brown residue was extracted with *n*-pentane (ca. 3x2 mL), the combined extracts were filtered through glass wool and celite, and the brown filtrate concentrated to ca. 0.75 mL volume under vacuum. Pale brown single crystals **S2** suitable for X-ray diffraction could then be grown at -35 °C over a week. The crystals were washed with small amounts of cold -35 °C *n*-pentane (ca. 3x0.2 mL) and dried under vacuum. Yield: 8.2 mg, 0.0183 mmol, 40 %.[a]

^{1}H NMR (500 MHz, C_6D_6, 298 K): δ δ 6.74 (s, 2H, Mes–C***H***), 6.70 (s, 2H, Mes–C***H***), 4.94 (s, 1H, BDI–C***H***), 3.69 (t, J = 3.2 Hz, 1H, Al–CH_2–C***H***), 3.27 (s, 3H, O–C***H***$_3$), 2.45 (s, 6H, Mes–C***H***$_3$), 2.13 (s, 6H, Mes–C***H***$_3$), 2.03 (s, 6H, Mes–C***H***$_3$), 1.47 (s, 6H, BDI–C***H***$_3$), 0.74 (d, J = 3.2 Hz, 2H, Al–C***H***$_2$).

---

[a] Though the crystalline material is **S2**, the sample appeared to be contaminated with a significant amount of amorphous brown material. Sample purity could not be improved *via* repeated crystallisation.

$^{13}C\{^1H\}$ NMR (126 MHz, C_6D_6, 298 K): 170.8 (2C, BDI–***C***N), 165.8 (O–***C***–OCH_3), 139.6 (2C, Mes–***C***N), 136.1 (2C, Mes–***C***CH_3), 134.7 (2C, Mes–***C***CH_3), 132.3 (2C, Mes–***C***CH_3), 130.4 (2C, Mes–C***H***), 129.4 (2C, Mes–C***H***), 97.7 (BDI–***C***H), 66.8 (Al–CH_2–***C***H), 53.4 (O–***C***H_3), 22.3 (2C, Mes–***C***H_3), 20.9 (2C, Mes–***C***H_3), 18.7 (2C, Mes–***C***H_3), 18.6 (2C, BDI–***C***H_3), 2.5 (br s, Al–***C***H_2–CH, FWHM = 52.5 Hz).

*General procedure for the synthesis of **6**, **7**, and **8***

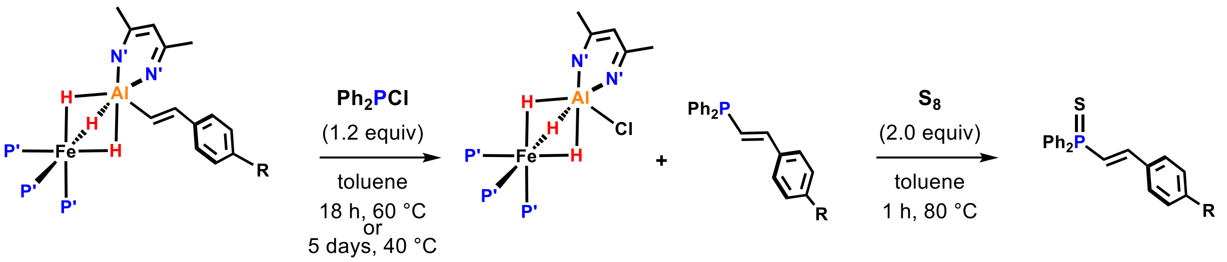

In a glovebox, a solution of **4** (0.05-0.1 mmol, 1 equiv.) in toluene (ca. 4.0 mL) was transferred to a microwave vial, then Ph_2PCl (0.06-0.12 mmol, 1.2 equiv.) was added via micropipette. The mixture was heated to 60 °C overnight,[b] then vial was cycled back into the glovebox. NMR analysis of the reaction mixture showed complete consumption of **4** and >99 % conversion to **6** and **7**. The opaque yellow mixture was filtered through glass wool and celite and the solids further extracted with toluene (ca. 2.0 mL). The volatiles were evaporated under vacuum, then the yellow crystalline residue was extracted with HMDSO (ca. 4 x 1.0 mL), then the volatiles once again removed under vacuum. The colourless to pale-yellow residue was taken up in toluene (ca. 2.0 mL), transferred to a microwave vial, then S_8 (0.1-0.2 mmol, 2.0 equiv.) was added. The vial was sealed, then the mixture was heated to 80 °C for 1 h. The pale-yellow to murky brown-black reaction mixture[c] was filtered through glass wool and celite, and the residue was extracted with $CHCl_3$ (ca. 3 x 1.0 mL). NMR analysis of the crude mixture showed complete consumption of **7** and >99 % conversion to **8**. The product was purified by column chromatography (10 % EtOAc in hexanes), followed by recrystallisation from hot hexanes (ca. 3.0 mL) at -35 °C. The spectroscopic data matched those reported in the literature.[2,3]

---

[b] Alternatively, the reaction can also be performed over 5 days at 40 °C. This minimizes the formation of the Ph_4P_2 side-product, with the trade-off of the longer reaction time.

[c] The presence of black solid (Fe_2S_3) is indicative of **6** carried through the HMDSO extraction due to incomplete removal of the toluene solvent in the step before. This does not affect the outcome of the reaction or that of the purification.

*Synthesis of **7a** and **8a***

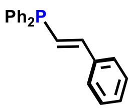

7a

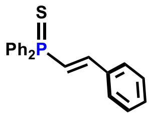

8a

7a and **8a** were prepared via the general procedure for the synthesis of **6**, **7**, and **8** using **4a** (75.1 mg, 0.1 mmol, 1 equiv.), Ph_2PCl (21.6 µL, 0.12 mmol, 1.2 equiv.) and S_8 (51.3 mg, 0.2 mmol, 2.0 equiv.). **8a** was purified by column chromatography (10 % EtOAc in hexanes, R_f = 0.40), followed by recrystallisation from hot hexanes at -35 °C. Yield: 27.2 mg, 0.0849 mmol, 85 %, colourless needle crystals. The spectroscopic data matched those reported in the literature.[2,3]

*Spectroscopic data for **7a***

$^{31}P\{^1H\}$ NMR (162 MHz, C_6D_6, 298 K): δ -11.35 (s, 1P).

*Spectroscopic data for **8a***

^1H NMR (400 MHz, $CDCl_3$, 298 K): δ ^1H NMR (400 MHz, C_6D_6) δ 7.87 – 7.77 (m, 4H), 7.68 – 7.43 (m, 9H), 7.43 – 7.33 (m, 3H), 6.96 (dd, J = 21.1, 16.7 Hz, 1H).

$^{31}P\{^1H\}$ NMR (162 MHz, $CDCl_3$, 298 K): δ 37.20 (s, 1P, P=S).

*Synthesis of **7a-d$_7$** and **8a-d$_7$***

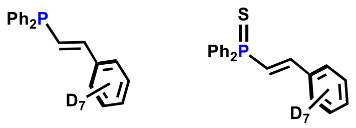

7a-d$_7$ **8a-d$_7$**

7a-d$_7$ and **8a-d$_7$** were prepared via the general procedure for the synthesis of **6**, **7**, and **8** using **4a-d$_8$** (50.0 mg, 0.066 mmol, 1 equiv.), Ph_2PCl (14.3 µL, 0.0792 mmol, 1.2 equiv.) and S_8 (33.8 mg, 0.132 mmol, 2.0 equiv.). **8a-d$_7$** was purified by column chromatography (10 % EtOAc in hexanes, R_f = 0.40), followed by recrystallisation from hot hexanes at -35 °C. Yield: 10.9 mg, 0.031 mmol, 50 %, colourless needle crystals.

*Spectroscopic data for **7a-d$_7$***

$^{31}P\{^1H\}$ NMR (162 MHz, C_6D_6, 298 K): δ -11.35 (s, 1P).

*Spectroscopic data for **8a-d$_7$***

^1H NMR (400 MHz, $CDCl_3$, 298 K): δ 7.87 – 7.76 (m, 4H), 7.55 – 7.43 (m, 6H).

^2H NMR (400 MHz, $CHCl_3$, 298 K): δ 7.70-7.54 (br m, 3D), 7.49-7.48 (br m, 2D), 7.13-6.89 (br m, 1D).

$^{31}P\{^1H\}$ NMR (162 MHz, $CDCl_3$, 298 K): δ 37.05 (s, 3P).

*Synthesis of **7b** and **8b***

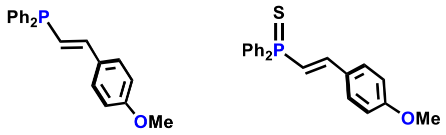

7b **8b**

7b and **8b** were prepared via the general procedure for the synthesis of **6**, **7**, and **8** using **4b** (78.1 mg, 0.1 mmol, 1 equiv.), Ph_2PCl (21.6 µL, 0.12 mmol, 1.2 equiv.) and S_8 (51.3 mg, 0.2 mmol, 2.0 equiv.). **8b** was purified by column chromatography (10 % EtOAc in hexanes, R_f = 0.28), followed by recrystallisation from hot hexanes at -35 °C. Yield: 10.9 mg, 0.031 mmol, 31 %, colourless needle crystals. The spectroscopic data matched those reported in the literature. [2,3]

*Spectroscopic data for **7b***

$^{31}P\{^1H\}$ NMR (162 MHz, $CDCl_3$, 298 K): δ -11.20 (s, 1P).

*Spectroscopic data for **8b***

^1H NMR (400 MHz, $CDCl_3$, 298 K): δ 7.87 – 7.76 (m, 4H), 7.61 – 7.41 (m, 9H), 6.94 – 6.86 (m, 2H), 6.78 (dd, J = 21.3, 16.6 Hz, 1H), 3.83 (s, 3H).

$^{31}P\{^1H\}$ NMR (162 MHz, $CDCl_3$, 298 K): δ 37.40 (s, 1P, P=S).

*Synthesis of **7c** and **8c***

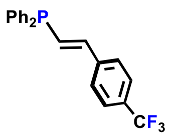

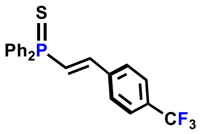

7c **8c**

7c and **8c** were prepared via the general procedure for the synthesis of **6**, **7**, and **8** using **4c** (41.0 mg, 0.05 mmol, 1 equiv.), Ph_2PCl (11 µL, 0.06 mmol, 1.2 equiv.) and S_8 (25.7 mg, 0.1 mmol, 2.0 equiv.). **8c** was purified by column chromatography (10 % EtOAc in hexanes, R_f = 0.42), followed by recrystallisation from hot hexanes at -35 °C. Yield: 16.1 mg, 0.0414 mmol, 83 %, colourless needle crystals. Colourless needle crystals suitable for X-ray diffraction were grown by slow evaporation of hexanes solvent over the course of 5 days. The spectroscopic data matched those reported in the literature. [2,3]

*Spectroscopic data for **7c***

$^{31}P\{^1H\}$ NMR (162 MHz, C_6D_6, 298 K): δ -10.92 (s, 1P).

$^{19}F\{^1H\}$ NMR (377 MHz, C_6D_6, 298 K) δ -62.24 (s, 3F, CF_3).

*Spectroscopic data for **8c***

$^{31}P\{^1H\}$ NMR (162 MHz, C_6D_6, 298 K): δ 36.22 (s, 1P, P=S).

$^{19}F\{^1H\}$ NMR (377 MHz, C_6D_6, 298 K) δ -62.42 (s, 3F, CF_3).

^1H NMR (400 MHz, $CDCl_3$, 298 K): δ 7.87 – 7.76 (m, 4H), 7.71 – 7.57 (m, 5H), 7.56 – 7.43 (m, 6H), 7.08 (dd, J = 20.3, 16.7 Hz, 1H).

$^{31}P\{^1H\}$ NMR (162 MHz, $CDCl_3$, 298 K): δ 36.94 (s, 1P, P=S).

$^{19}F\{^1H\}$ NMR (377 MHz, $CDCl_3$, 298 K) δ -62.78 (s, 3F, CF_3).

*Isolation of **6** and **6-d₁***

P' = PMe_3
N' = NMes

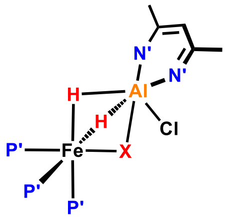

6 (X = H)
6-d$_1$ (X = D)

From the syntheses of **6**, **7**, and **8**, **6(-d$_1$)** could be isolated from the yellow residue left after the HMDSO extraction step. Crystallisation from concentrated 1:1 toluene:pentane solutions at -35 °C over a week gave **6(-d$_1$)** as yellow block crystals. Representative isolated yield (from 0.1 mmol **4a**): 35.7 mg, 0.0857 mmol, 86 %.

^{1}H NMR (400 MHz, C_6D_6, 298 K): δ 6.83 (s, 2H, Mes-C***H***), 6.75 (s, 2H, Mes-C***H***), 5.38 (s, 1H, BDI-C***H***), 2.83 (s, 6H, Mes-C***H***$_3$), 2.32 (s, 6H, Mes-C***H***$_3$), 2.19 (s, 6H, Mes-C***H***$_3$), 1.53 (s, 6H, BDI-C***H***$_3$), 0.93 (m, 27 H, P-C***H***$_3$), -15.73 (br s, 3H, Fe–***H***–Al).

^{31}P{^{1}H} NMR (162 MHz, C_6D_6, 298 K): δ 28.81 (s, 3P).

^{13}C{^{1}H} NMR (101 MHz, C_6D_6, 298 K): δ 168.0 (2C, BDI-***C***N), 145.8 (2C, Mes-***C***N), 136.8 (2C, Mes-***C***CH$_3$), 134.6 (2C, Mes-***C***CH$_3$), 133.6 (2C, Mes-***C***CH$_3$), 130.4 (2C, Mes-***C***H), 129.2 (2C, Mes-***C***H), 101.1 (1C, BDI-***C***H), 26.1 (m 9C, P-***C***H$_3$), 24.1 (2C, BDI-***C***H$_3$), 21.5 (2C, Mes-***C***H$_3$), 20.9 (2C, BDI-***C***H$_3$), 20.2 (2C, Mes-***C***H$_3$).

*Selected NMR resonances for **6-d$_1$***

^{1}H NMR (400 MHz, C_6D_6, 298 K): δ 5.38 (s, 1H, BDI-C***H***, ca. 40 % D incorporation), -15.73 (br s, 2H, Fe–***H***–Al, 100 % D incorporation).

^{2}H NMR (400 MHz, C_6H_6, 298 K): δ 5.38 (br s, BDI-C***H***), -15.73 (br s, 1D, Fe–***D***–Al).

3. Reversible Alkene Binding

*NMR spectra for the reaction of **1** with styrene (10 – 40 equiv.):*

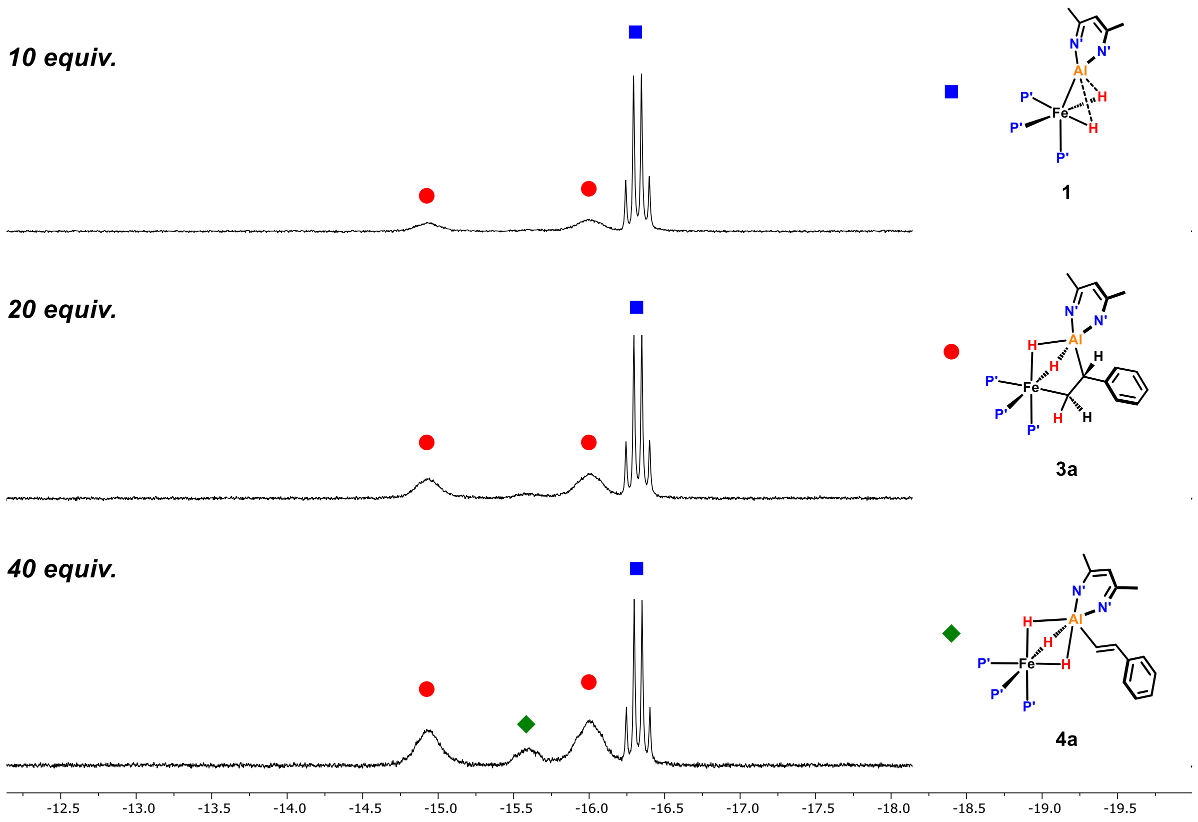

Figure S2. Stacked ^{1}H NMR spectra (hydride region, 400 MHz, C_6D_6, 298 K). **1** (■), **3a** (●), **4a** (◆).

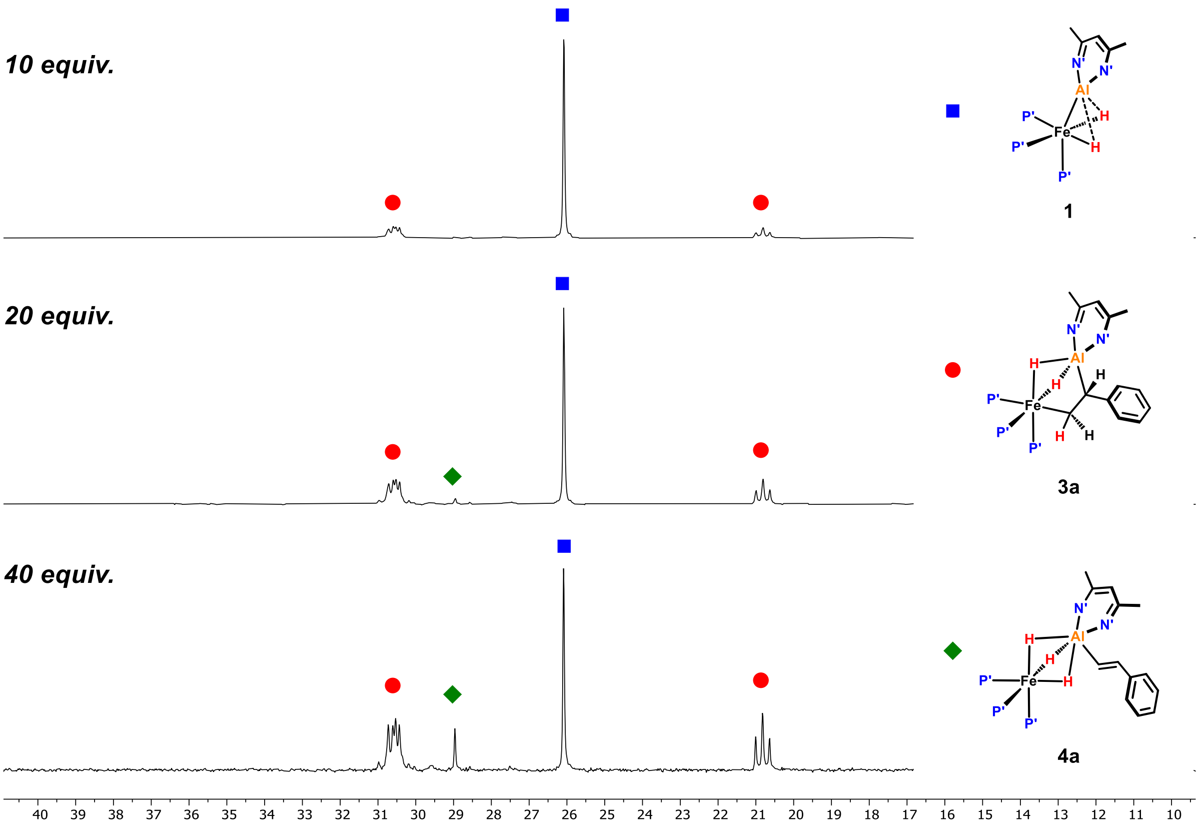

Figure S3. Stacked $^{31}P\{^{1}H\}$ NMR spectra (162 MHz, C_6D_6, 298 K). **1** (■), **3a** (●), **4a** (◆).

*Van’t Hoff analysis for the equilibrium reaction between **1** and styrene (21.5 equiv.):*

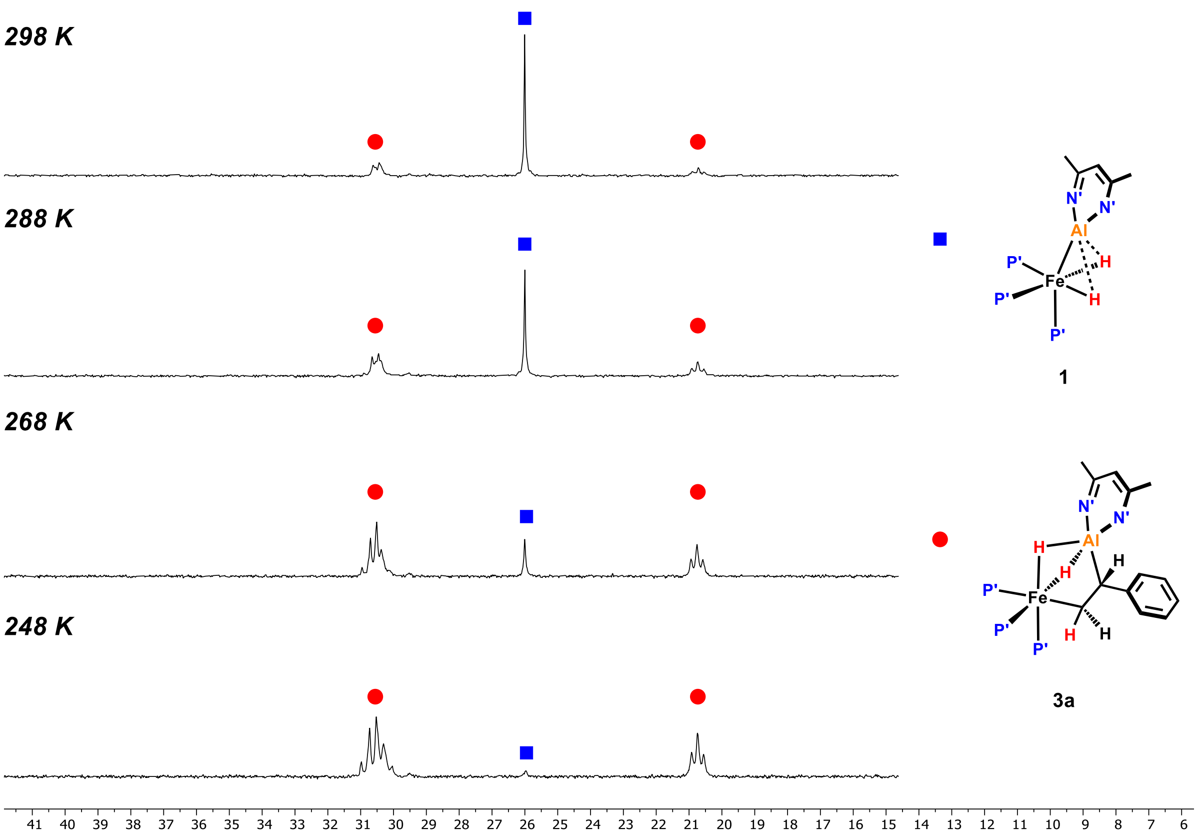

Figure S4. Stacked $^{31}P\{^{1}H\}$ NMR spectra (162 MHz, toluene-d_8, 21.6 equiv. of styrene, 248-298 K). **1** (■), **3a** (●).

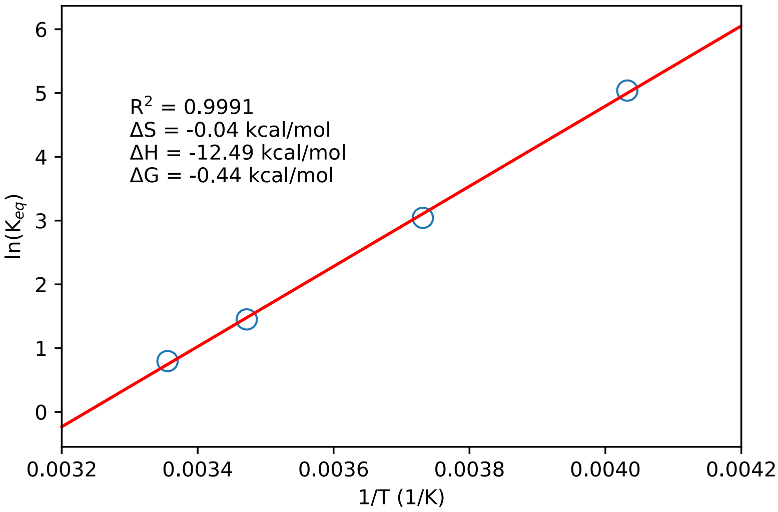

Figure S5. Van’t Hoff plot for the equilibrium between **1** + styrene and **3a** in toluene-d_8. Fitted using data obtained by $^{31}P\{^{1}H\}$ NMR for a temperature range of 248-298 K using 21.6 equiv. of styrene substrate.

4. Kinetic Experiments

*__KIE measurements for the reaction between **1** and styrene(-d_8)__*

KIE measurements we conducted by recording $^{31}P\{^{1}H\}$ time course for the reaction of **1** with styrene(-d_8) in two separate experiments under *pseudo*-first-order conditions. In a glovebox, a solution of **1** (5 mg, 0.0077 mmol) in C_6D_6 (0.5 mL) was transferred to a J. Youngs NMR tube containing a PPh_3 capillary (0.19 M in C_6D_6). To this styrene or styrene-d_8 (18.7 µL, 0.16 mmol, 21 equiv.) was added via micropipette, the NMR tube sealed, shaken and removed from the glovebox. The sample was kept in liquid nitrogen till the NMR tube was inserted into the pre-warmed (50 °C) spectrometer probe. Continuous monitoring of the reaction by NMR spectroscopy showed conversion of the starting materials into **4a** or **4a-*d*₈** overnight.

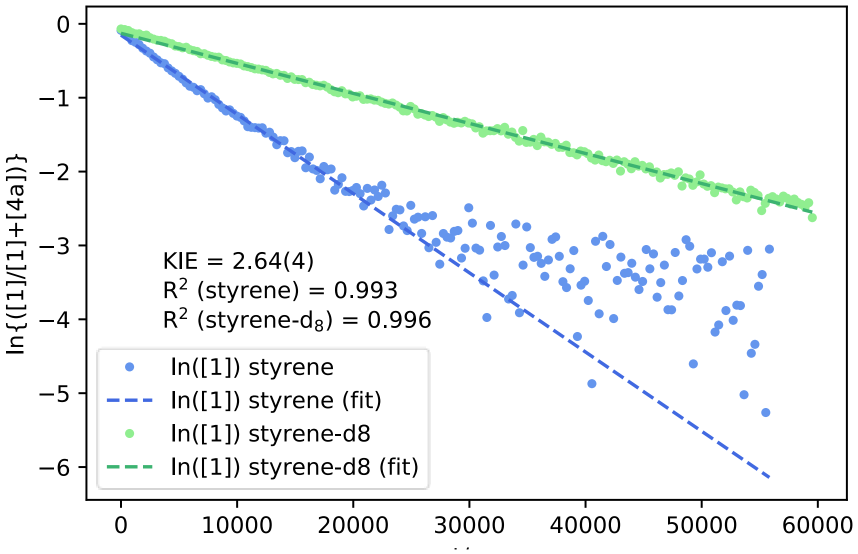

Figure S6. Data fitting for the KIE measurements for the reaction of **1** and styrene(-d_8) (50 °C, 21 equiv., C_6D_6, data points measured using $^{31}P\{^{1}H\}$ NMR). Only data measured in the first 20000 s (64 data points, 90 % conversion, 3.3 half-lives) were used for styrene, and the whole dataset (60000 s, 192 data points, 92 % conversion, 3.6 half-lives) was used for the fit for styrene-d_8.

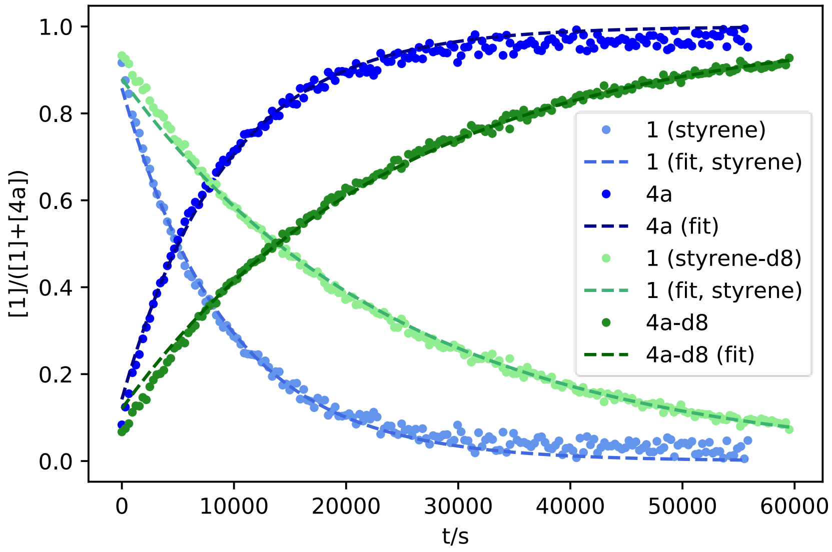

Figure S7. NMR time course for the KIE measurement for the reaction between **1** and styrene(-d_8) (50 °C, 21 equiv., C_6D_6, data points measured using $^{31}P\{^{1}H\}$ NMR).

__Room temperature reaction plots of 1 (10 mg, 0.015 mmol) + styrene substrate (0.150 mmol, 10 eq.) in C_6D_6 (0.5 mL) showing experimental (dots) and Copasi[4] fitted (solid lines) data__

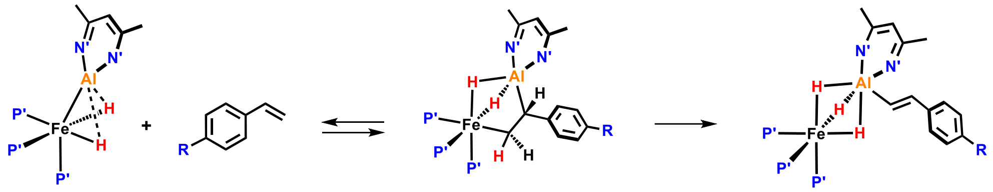

1 P' = PMe_3 (10 equiv.) **3a** R = H **4a** R = H

N' = Mes **3b** R = OMe **4b** R = OMe

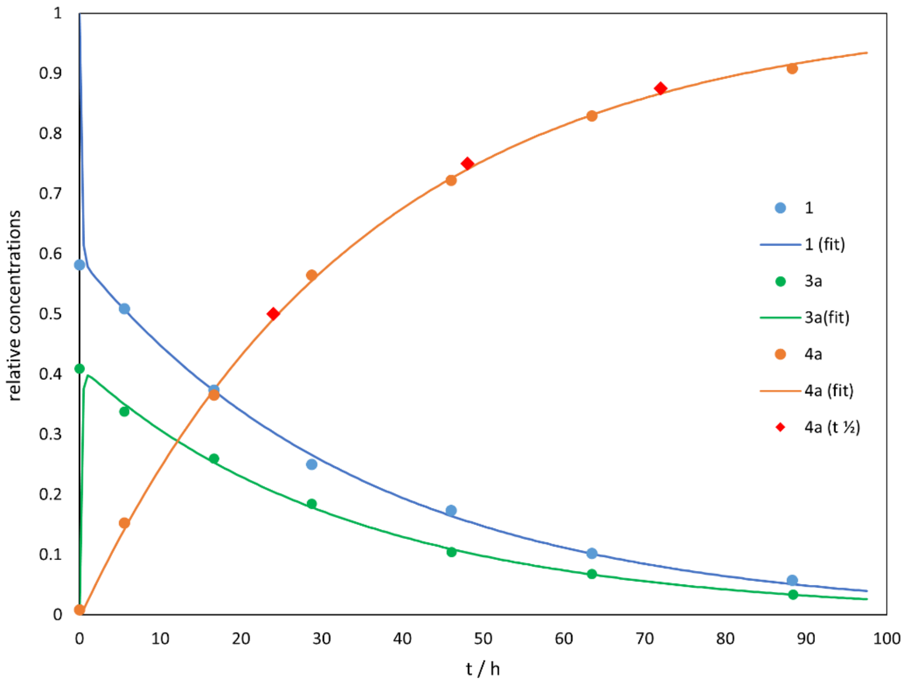

Figure S8. Plot of relative concentrations vs time for the reaction of **1** with styrene (298 K, C_6D_6).

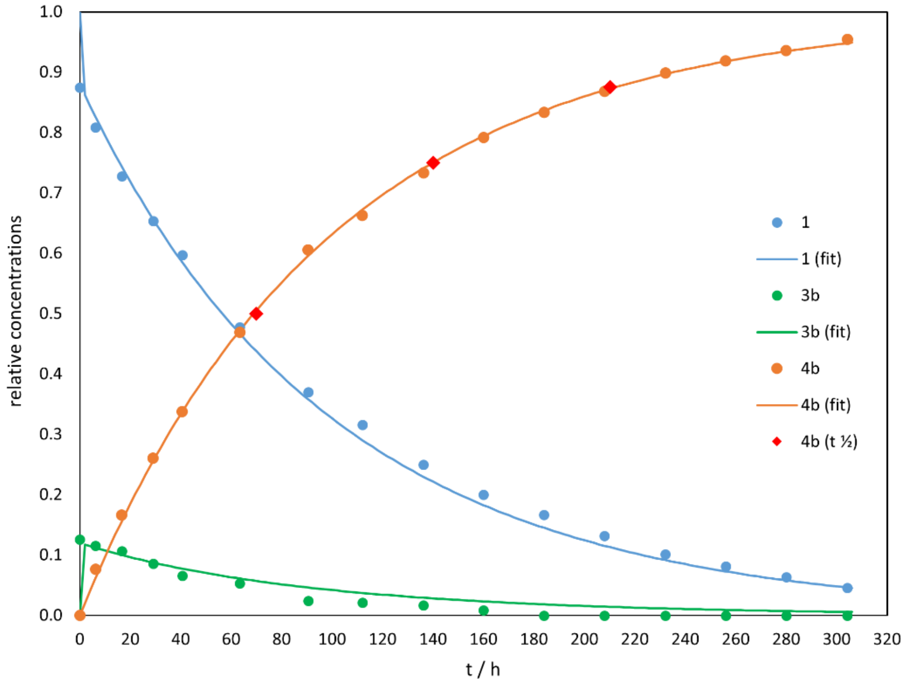

Figure S9. Plot of relative concentrations vs time for the reaction of **1** with 4-methoxy styrene (298 K, C_6D_6).

5. Computational Methods

DFT calculations were run using Gaussian 09 (Revision D.01).[5] NBO analysis was performed using the NBO 6.0 version program.[6] Geometry optimisation calculations were performed without symmetry constraints. Frequency analyses for all stationary points were performed using the enhanced criteria to confirm the nature of the structures as either minima (no imaginary frequency) or transition states (only one imaginary frequency). Intrinsic reaction coordinate (IRC) calculations followed by full geometry optimisations on final points were used to connect transition states and minima located on the potential energy surface allowing a full energy profile (calculated at 298.15 K, 1 atm) of the reaction to be constructed.[7,8] Solvent corrections were applied using the polarizable continuum model (PCM).[9] Dispersion corrections were applied using Grimme's D3 correction.[10] Geometry optimisations were carried out using the B3PW91[11] functional including solvent and dispersion corrections directly in the optimisations. Al and Fe centres were described with Stuttgart SDDAll ECP and associated basis sets, and the 6-31G** basis sets were used for all other atoms.[12,13,14] Single point energy calculations were performed on the geometries optimized on the B3PW91-D3/6-31G**/SDDAll /PCM level using the B3LYP[15,16] functional and the Def2-TZVPP[17] basis set including dispersion (D3) and solvent (PCM) corrections.

Although our previous computational model (B3PW91-D3/6-31G**/SDDAll /PCM) has been benchmarked against experimental data[1] and proved to predict transition state energies very well it tends to over-stabilize the cycloaddition intermediates. We thus employed additional single point corrections on the B3LYP-D3/Def2-TZVPP/SDDAll/PCM level of theory. The corrected free energies are in good agreement with experimentally obtained data for both, the thermochemistry of the cycloaddition of styrene to **1** (Van't Hoff analysis) as well as the reaction barrier intramolecular C–H activation of **1** (Eyring analysis) reported previously.[1] The calculated free energies are consistently ~ 2 kcal/mol lower than the experimental ΔG values (Table S1) and should therefore allow for a reasonable prediction of the reaction barriers. For comparison, calculations were also conducted using r^{2}SCAN-3c[18] implemented in ORCA 5.0.3[19] as state-of-the-art composite method. A summary of the extended functional / basis set testing is given in Table S2.

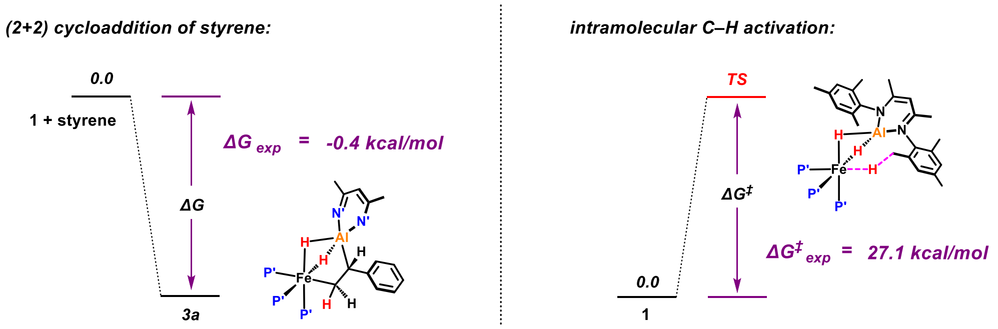

Optimisation	Single Point Correction	ΔG (kcal/mol)	ΔG‡ (kcal/mol)
B3PW91 / 6-31G** / SDDAll<br>PCM (toluene) / D3	------------<br>------------	-6.0	27.9
B3PW91 / 6-31G** / SDDAll<br>PCM (toluene) / D3	B3LYP / Def2-TZVPP / SDDAll<br>PCM (toluene) / D3	-2.3	25.8
r^{2}SCAN-3c<br>------------	B3LYP / Def2-TZVPP<br>D4 / SMD (toluene)	-2.5	24.3

Table S1. Comparison of calculated free Energies with experimentally obtained ΔG values.

Extended functional / basis set testing

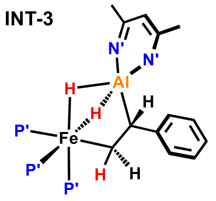

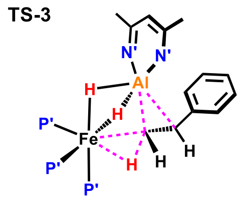

Optimisation	Single Point Correction	ΔG (INT-3)	ΔG (TS-3)	ΔG‡ (INT-3 → TS-3)
B3PW91 / 6-31G** / SDDAll<br>no correction	------------<br>------------	**17.9**	**47.1**	**29.2**
B3PW91 / 6-31G** / SDDAll<br>PCM (benzene)	------------<br>------------	**17.1**	**43.1**	**26.0**
B3PW91 / 6-31G** / SDDAll<br>D3	------------<br>------------	**-7.3**	**23.3**	**30.6**
B3PW91 / 6-31G** / SDDAll<br>D3 (BJ)	------------<br>------------	**-10.8**	**19.2**	**30.0**
B3PW91 / 6-31G** / SDDAll<br>PCM (benzene) / D3	------------<br>------------	**-6.0**	**21.4**	**27.4**
B3PW91 / 6-31G** / SDDAll<br>no correction	B3PW91 / 6-31G** / SDDAll<br>PCM (benzene) / D3	**-5.2**	**21.2**	**26.4**
B3PW91 / 6-31G** / SDDAll<br>PCM (benzene) / D3	B3PW91 / Def2-TZVPP / SDDAll<br>PCM (benzene) / D3	**-9.4**	**15.3**	**24.7**
B3PW91 / 6-31G** / SDDAll<br>PCM (benzene) / D3	B3LYP / Def2-TZVPP / SDDAll<br>PCM (benzene) / D3	**-2.3**	**20.1**	**22.4**
B3PW91 / 6-31G** / SDDAll<br>PCM (benzene) / D3	B3LYP / Def2-TZVPP / SDDAll<br>PCM (toluene) / D3	**-2.3**	**19.9**	**22.2**
B3PW91 / 6-31G** / SDDAll<br>PCM (benzene) / D3	B3LYP / Def2-TZVPP / SDDAll<br>D3	**-2.2**	**24.4**	**26.7**
r^2SCAN-3c<br>------------	------------<br>------------	**-2.5**	**24.6**	**27.1**
r^2SCAN-3c<br>------------	B3PW91 / Def2-TZVPP<br>no correction	**15.6**	**42.3**	**26.7**
r^2SCAN-3c<br>------------	B3PW91 / Def2-TZVPP<br>D3(BJ)	**-10.6**	**18.0**	**28.6**
r^2SCAN-3c<br>------------	B3PW91 / Def2-TZVPP<br>D4	**-8.2**	**20.5**	**28.7**
r^2SCAN-3c<br>------------	PBE0 / Def2-TZVPP<br>D4	**-7.5**	**21.5**	**29.0**
r^2SCAN-3c<br>------------	B3LYP / Def2-TZVPP<br>D4	**-2.1**	**25.1**	**27.2**
r^2SCAN-3c<br>------------	B3LYP / Def2-TZVPP<br>D4 / SMD (toluene)	**1.2**	**21.7**	**20.5**

Table S2. Summary of the functional / basis set testing. Free energies in kcal/mol.

*Calculated mechanism for the reaction of **1** with phenylacetylene*

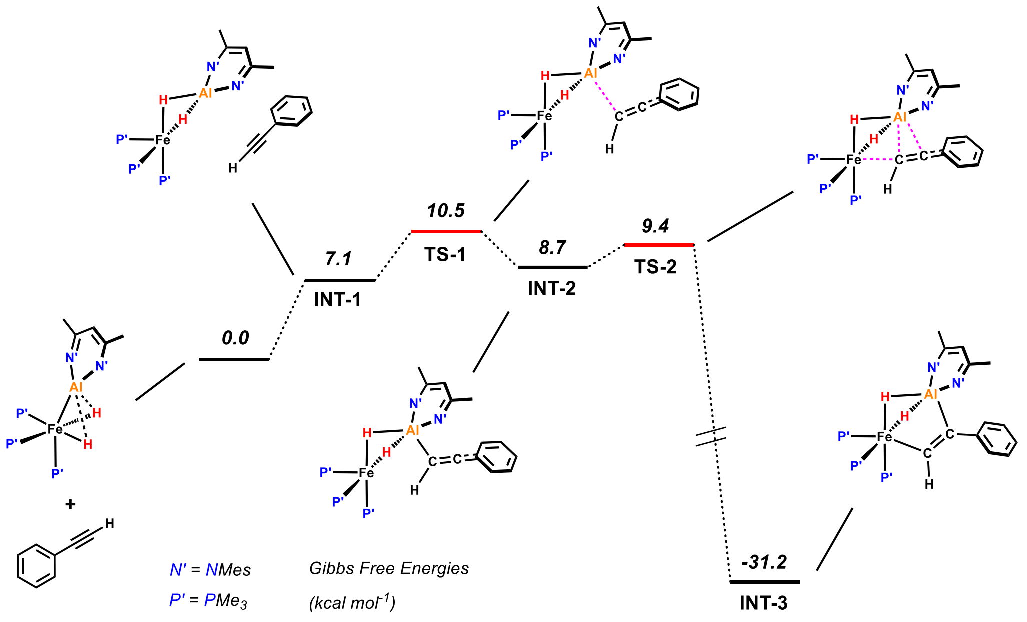

Figure S10. Calculated free energy profile for the reaction of **1** with phenylacetylene. ΔG in kcal/mol. B3LYP-D3 / Def2-TZVPP/ SDDAll / PCM (benzene) // B3PW91-D3 / 6-31G** / SDDAll / PCM (benzene).

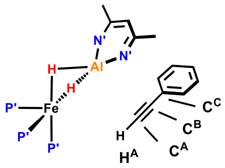

	INT-1	TS-1	INT-2	TS-2	INT-3
**Fe---Al (Å)**	*2.223*	*2.233*	*2.291*	*2.284*	*2.407*
**WBI_{Fe-Al}**	*0.51*	*0.47*	*0.26*	*0.18*	*0.12*
**Fe---C^A (Å)**	*4.640*	*4.105*	*3.457*	*2.969*	*2.021*
**WBI_{Fe-C}**	*0.00*	*0.02*	*0.16*	*0.21*	*0.49*
**Al---C^A (Å)**	*3.296*	*2.783*	*2.176*	*2.084*	*2.373*
**WBI_{Al-C}**	*0.04*	*0.10*	*0.35*	*0.41*	*0.11*
**Al---C^B (Å)**	*3.648*	*3.274*	*2.801*	*2.548*	*1.936*
**WBI_{Al-C}**	*0.01*	*0.03*	*0.08*	*0.09*	*0.44*
**C^A---C^B (Å)**	*1.215*	*1.225*	*1.274*	*1.286*	*1.363*
**WBI_{C-C}**	*2.80*	*2.73*	*2.31*	*2.11*	*1.85*
**C^B---C^C (Å)**	*1.423*	*1.418*	*1.395*	*1.388*	*1.463*
**WBI_{C-C}**	*1.07*	*1.09*	*1.20*	*1.26*	*1.05*
**C^A---H^A (Å)**	*1.066*	*1.066*	*1.079*	*1.082*	*1.105*
**WBI_{C-H}**	*0.90*	*0.89*	*0.88*	*0.87*	*0.90*
NPA charges:					
**Fe**	*-1.05*	*-1.04*	*-0.81*	*-0.71*	*-0.65*
**Al**	*1.33*	*1.37*	*1.54*	*1.69*	*1.79*
**H^A**	*0.27*	*0.28*	*0.23*	*0.21*	*0.16*
**C^A**	*-0.22*	*-0.28*	*-0.58*	*-0.66*	*-0.44*
**C^B**	*-0.03*	*-0.02*	*-0.06*	*-0.17*	*-0.75*
**C^C**	*-0.11*	*-0.11*	*-0.10*	*-0.08*	*0.16*

Table S3. NBO analysis of the stationary points in the reaction of **1** with phenylacetylene. G09/NBO6 ωB97X/G-31G**/SSDAll.

*Calculated mechanism for the reaction of **1** with styrene*

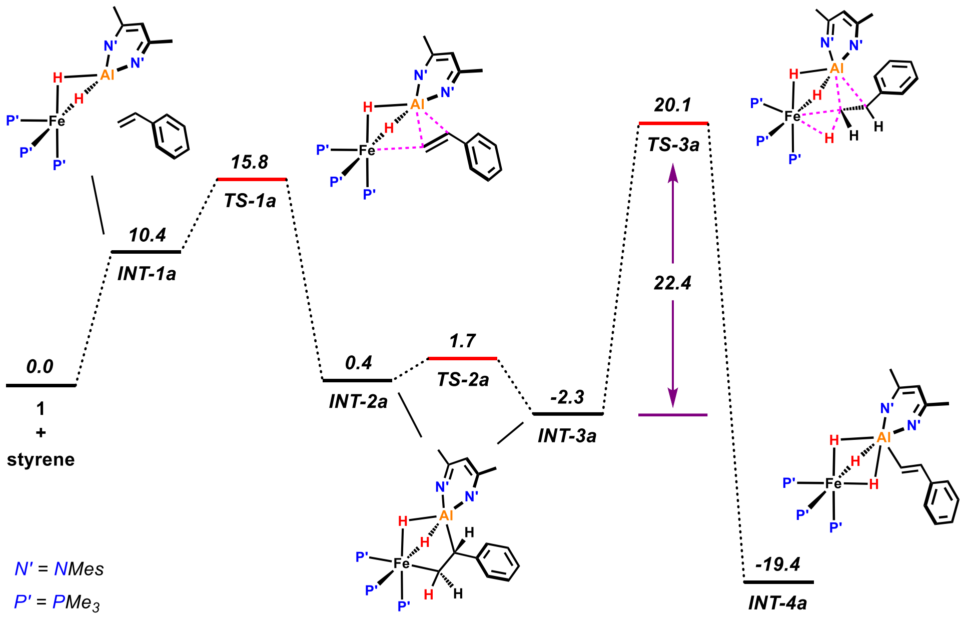

Figure S11. Calculated free energy profile for the reaction of **1** with styrene. ΔG in kcal/mol. B3LYP-D3 / Def2-TZVPP/ SDDAll / PCM (benzene) // B3PW91-D3 / 6-31G** / SDDAll / PCM (benzene).

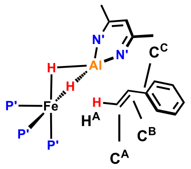

	INT-1	TS-1	INT-2	TS-2	INT-3	TS-3	INT-4
**Fe---Al (Å)**	2.224	2.289	2.391	2.402	2.372	2.315	2.336
**WBI_{Fe-Al}**	0.49	0.16	0.12	0.12	0.12	0.13	0.15
**Fe---C^A (Å)**	4.388	2.938	2.142	2.134	2.133	2.391	3.703
**WBI_{Fe-C}**	0.01	0.25	0.48	0.48	0.46	0.24	0.01
**Al---C^A (Å)**	3.026	2.138	2.498	2.518	2.305	1.998	2.014
**WBI_{Al-C}**	0.06	0.36	0.08	0.07	0.11	0.36	0.42
**Al---C^B (Å)**	3.520	2.495	1.988	1.982	1.983	2.834	3.057
**WBI_{Al-C}**	0.02	0.09	0.37	0.38	0.38	0.05	0.02
**C^A---C^B (Å)**	1.347	1.450	1.558	1.553	1.541	1.490	1.347
**WBI_{C-C}**	1.86	1.17	1.01	1.02	1.02	1.05	1.89
**C^B---C^C (Å)**	1.464	1.425	1.485	1.489	1.491	1.406	1.473
**WBI_{C-C}**	1.06	1.23	1.06	1.05	1.01	1.36	1.06
**C^A---H^A (Å)**	1.083	1.090	1.100	1.100	1.098	1.112	2.758
**WBI_{C-H}**	0.91	0.90	0.93	0.93	0.95	0.82	0.01
NPA charges:							
**Fe**	-1.05	-0.70	-0.63	-0.62	-0.62	-0.50	-0.86
**Al**	1.35	1.75	1.86	1.86	1.83	1.87	1.72
**H^A**	0.24	0.23	0.17	0.19	0.19	0.24	-0.18
**C^A**	-0.44	-0.81	-0.75	-0.75	-0.80	-1.08	-0.80
**C^B**	-0.23	-0.55	-0.90	-0.91	-0.88	-0.57	-0.25
**C^C**	-0.07	-0.03	0.02	0.01	-0.01	-0.02	-0.06

Table S4. NBO analysis of the stationary points in the reaction of **1** with phenylacetylene. G09/NBO6 ωB97X/G-31G**/SSDAll.

Calculated sp C–H activation of terminal alkynes

Experimentally, no C-H activation at higher temperatures (60-100 °C) was observed for the aryl substituted alkynes in **2a** and **2d**. The (2+2) addition product **2b** (from the reaction of **1** with trimethylsilylacetylene) slowly reacts at 80 °C give to the corresponding C–H activation product (18 h, >99 % NMR yield). DFT calculations give some insight into the divergent reactivity (Figure S1, Figure S12). Though we did not manage to find a transition state for the C–H activation step our calculation show that – presumably due to steric reasons – the alkyne complex of trimethylsilylacetylene **2b** is about 10 kcal/mol less stable than the respective phenylacetylene complex **2a** (**2a**: ΔG = -31.2 kcal/mol; **2b**: ΔG = --19.2 kcal/mol; free energies relative to the starting materials).

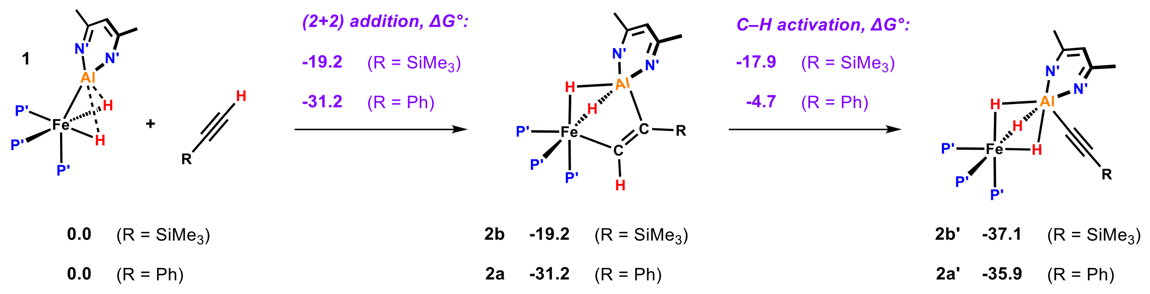

Figure S12. Calculated thermochemistry for the (2+2) addition and sp C–H activation of terminal alkynes with **1**. Gibbs free energies in kcal/mol relative to the starting materials (**1** + alkyne). B3LYP-D3 / Def2-TZVPP / SDDAll (Fe,Al) // B3PW91-D3 (PCM, benzene) / 6-31G** / SDDAll (Fe,Al).

Bonding analysis of the alkene complex **2b** and alkyne complex **4b**

QTAIM analysis

QTAIM analysis was conducted with Multiwfn.[20]

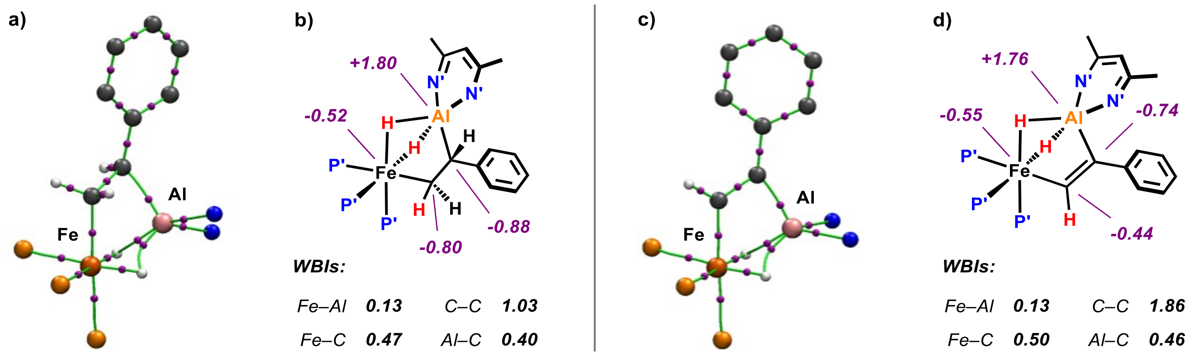

Figure S13. Right: QTAIM analysis of a) **3a** and c) **2a**. Bond critical points are shown in purple; bond critical paths in green. NPA charges and Wiberg Bond Indices (WBIs) for b) **3a** and d) **2a**.

ETS-NOCV calculations

ETS-NOCV calculations were performed in the Orca 4.2.1 quantum chemistry software package.[21,22] The calculations were run using the ωB97x functional with the def2-TZVPP basis set. Calculations were performed with the resolution of identity approximation for the Coulomb integrals, and chain of spheres approximation for the exchange integrals (RIJCOSX) with the def2/j auxiliary basis set.[23]

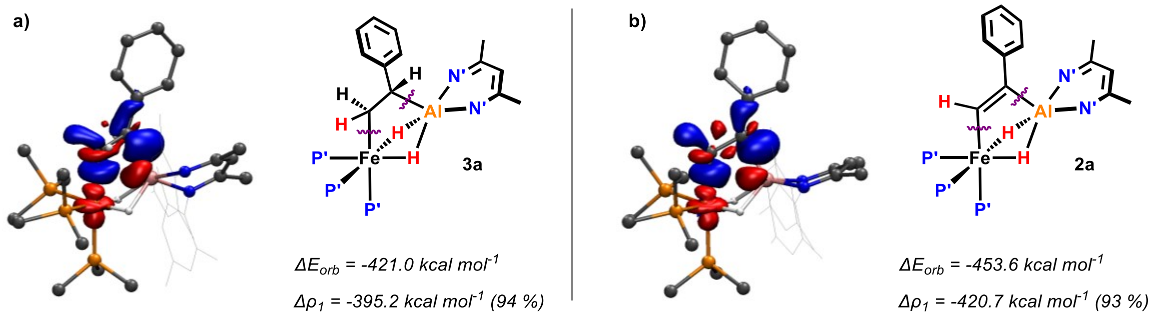

Figure S14. ETS-NOCV deformation density plots ($\Delta\rho_1$) for a) **3a** and b) **2a**. Charge flow from red to blue.

*CLMO analysis of **TS-3a***

In order to get better insight into the nature of **TS-3a** an analysis of the key localized molecular orbitals (LMOs) along the intrinsic reaction coordinate (IRC) was carried out. LMOs were calculated following the Pipek-Mezey criterion[24] and a procedure[25] described by Vidossich and Lledóss was used to generate centroids of these LMOs (CLMOs). The CLMOs were used to follow the bond rearrangements around **TS-3a**. For the sake of clarity, depiction of the overall process was separated in a pre- and post-TS stage.

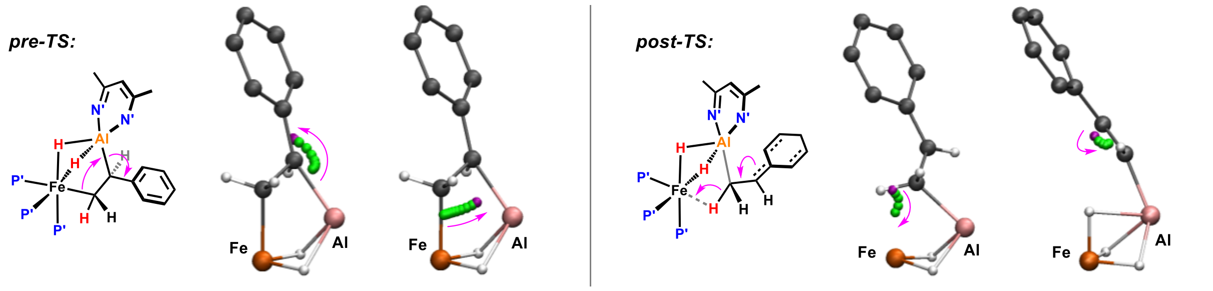

Figure S15. Visualization of bond rearrangements in **TS-3a** using LMO centroids (CLMOs).

*Substituent effect on the activation energy from **INT-3a** to **TS-3a***

The C–H activation step for the reaction of styrenes with **1** was calculated for the series **2a-c** (Figure S17). The free activation energies for these substrates appear to correlate with the respective Hammett parameters.

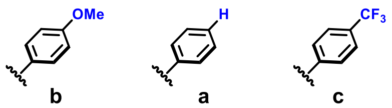

	b	a	c
σ_P [a]	-0.27	0	+0.54
**im. freq.** (cm^{-1})	-112.5	-92.3	-48.3
ΔG [b] (kcal mol^{-1})			
INT-3	**-0.6**	**-2.3**	**-6.2**
TS-3	**23.9**	**20.1**	**10.4**
INT-4	**-19.0**	**-19.4**	**-21.5**
ΔG‡	***24.5***	***22.3***	***16.6***

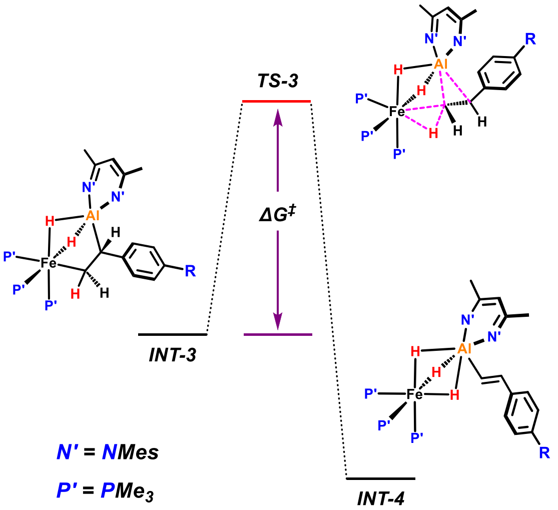

Figure S16. Calculated free activation energies (in kcal/mol relative to **1** + substrate) for the reactions of **1** with different styrene substrates.

Computationally, the series was expanded to 4-nitrostyrene as well due to the large positive Hammett parameter of the NO_2 substituent (σ^- = +1.27). In this extreme case, complete deconvolution of the rearrangement and C–H activation of the bound substrate into two separate transition states was with an overall barrier of only 9.3 kcal/mol for the reaction of **INT-3e** to **INT-4e** (Figure S18).

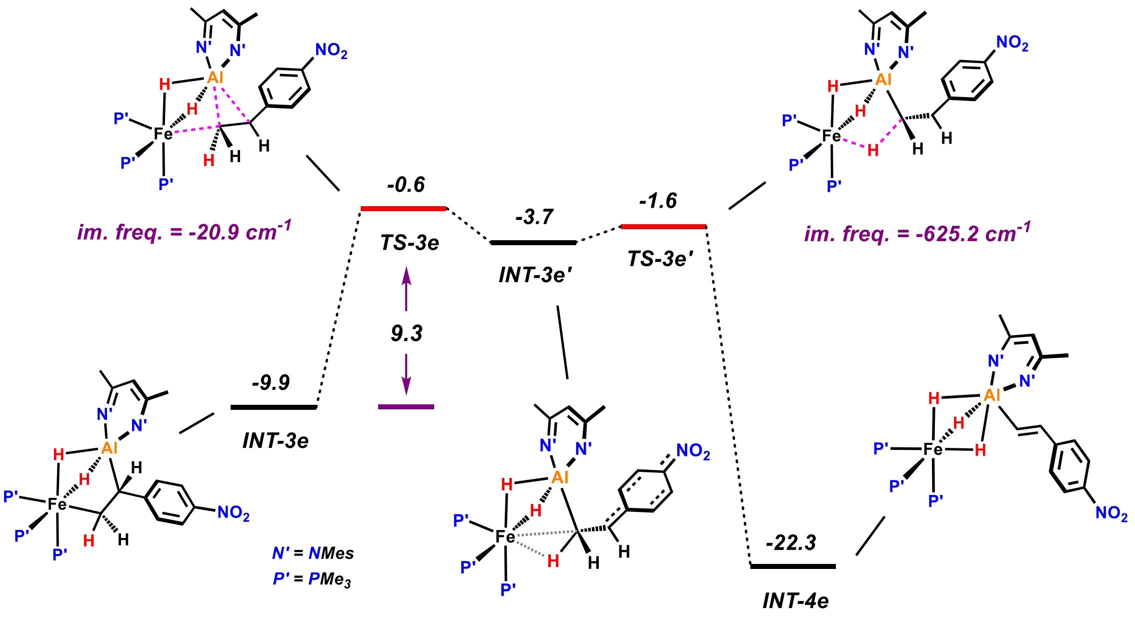

Figure S17. Calculated free energy profile for the vinylic C–H activation of 4-nitrostyrene (in kcal/mol relative to **1** + substrate).

Across the series, it appears that a linear relationship between the Hammett parameters and activation energies exists (Figure S19). For the nitro substituent, the σ⁻ (1.27) instead of the standard σ parameter (0.78) appears to be the better fit. The use of σ⁻ has for example been suggested for phenolates where the lone pair of the O⁻ can be delocalised into the p-NO_2 substituent.[26,27] This parallels the nature of the bound substrate in **TS-3e** in which the α-carbon carries a negative charge stabilised by the formation of a resonance structure with the adjacent 4-nitrophenyl group.

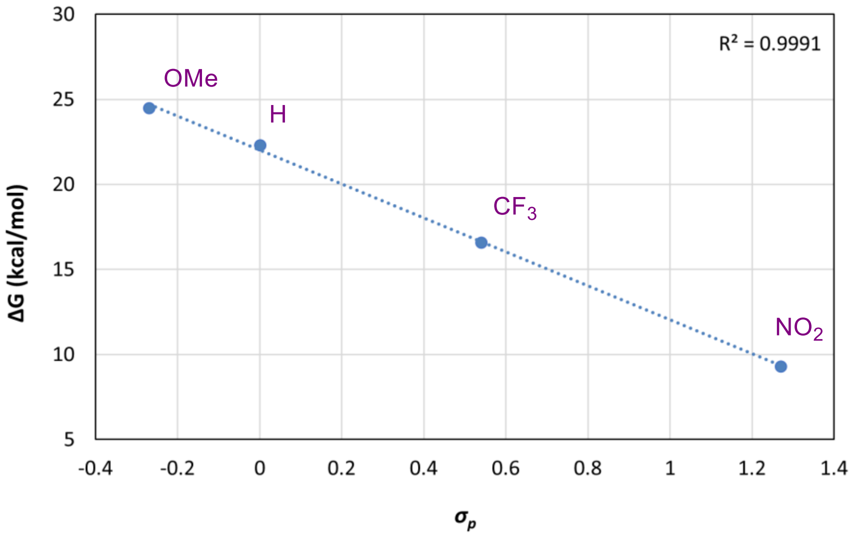

Figure S18. Plot of the activation energies (kcal/mol) vs. the respective Hammett parameters for the reaction of **1** with 4-substituted styrenes.

*Calculations on the reaction of **1** with 2-vinylpyridine*

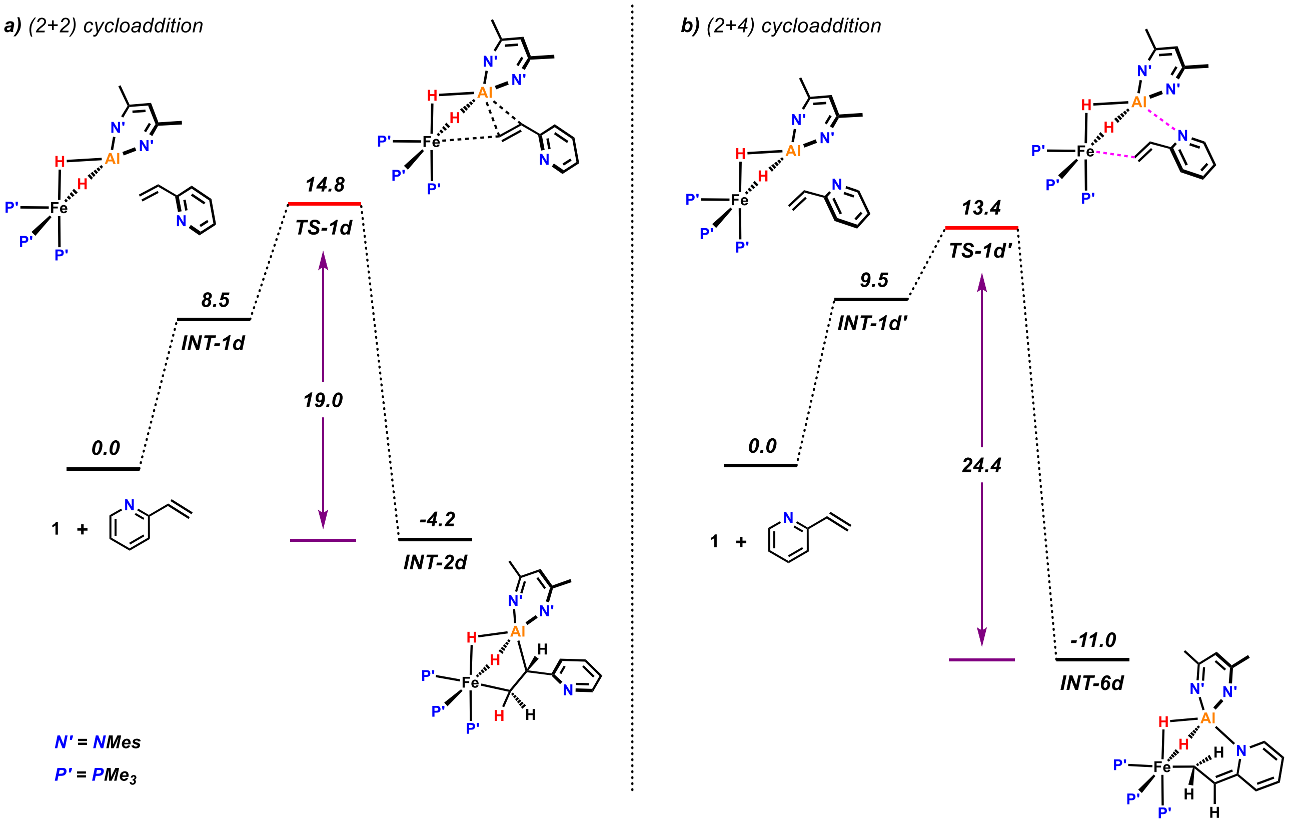

Figure S19. a) (2+2) and b) (2+4) cycloaddition of 2-vinylpyridine to **1** (ΔG in kcal/mol). B3LYP-D3 / Def2-TZVPP/ SDDAll / PCM (benzene) // B3PW91-D3 / 6-31G** / SDDAll / PCM (benzene).

Figure S20 shows the free energy profile for the reaction of **1** with 2-vinylpyridine catalysed by $MgBr_2 \cdot THF$ compared to pathways in absence of the catalyst (**TS-5d**, **TS-6d**, see Figure S21 as well). For the sake of comparability to the uncatalyzed pathways, $MgBr_2.THF$ was included as adduct to the final product (**4d---LA**). This way, $MgBr_2 \cdot THF$ is directly transferred from the end (**INT-4d**) to the start (**INT-2d**) of the reaction thereby avoiding "free" $MgBr_2 \cdot THF$ in the calculations.

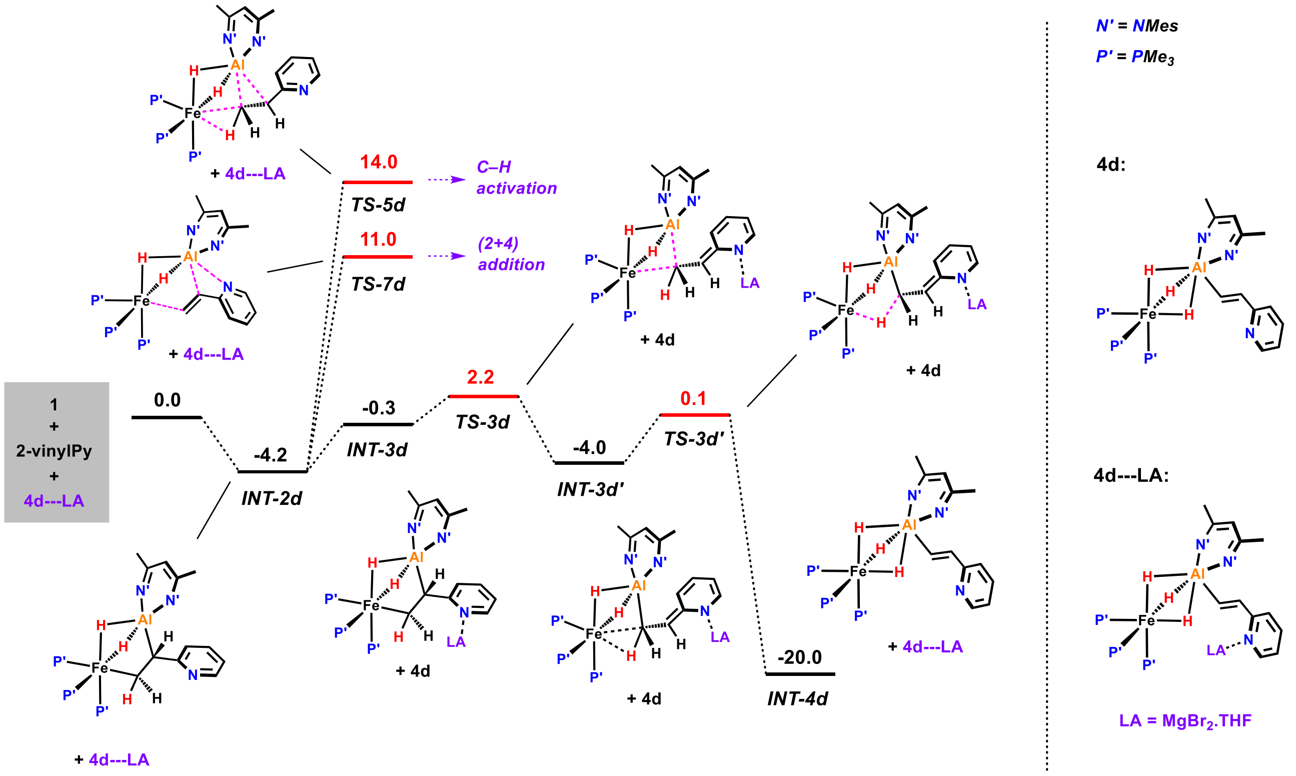

Figure S20. Calculated free energy profile for the vinylic C–H activation of 2-vinylpyridine catalysed by $MgBr_2$ (ΔG in kcal/mol). B3LYP-D3 / Def2-TZVPP/ SDDAll / PCM (benzene) // B3PW91-D3 / 6-31G** / SDDAll / PCM (benzene).

The calculated pathways in absence of $MgBr_2 \cdot THF$ are summarized in Figure S21. The obtained reaction barrier for the conversion of **INT-2d** to **INT-4d** is significantly higher in than for the catalysed pathway (18.2 vs. 6.4 kcal/mol).

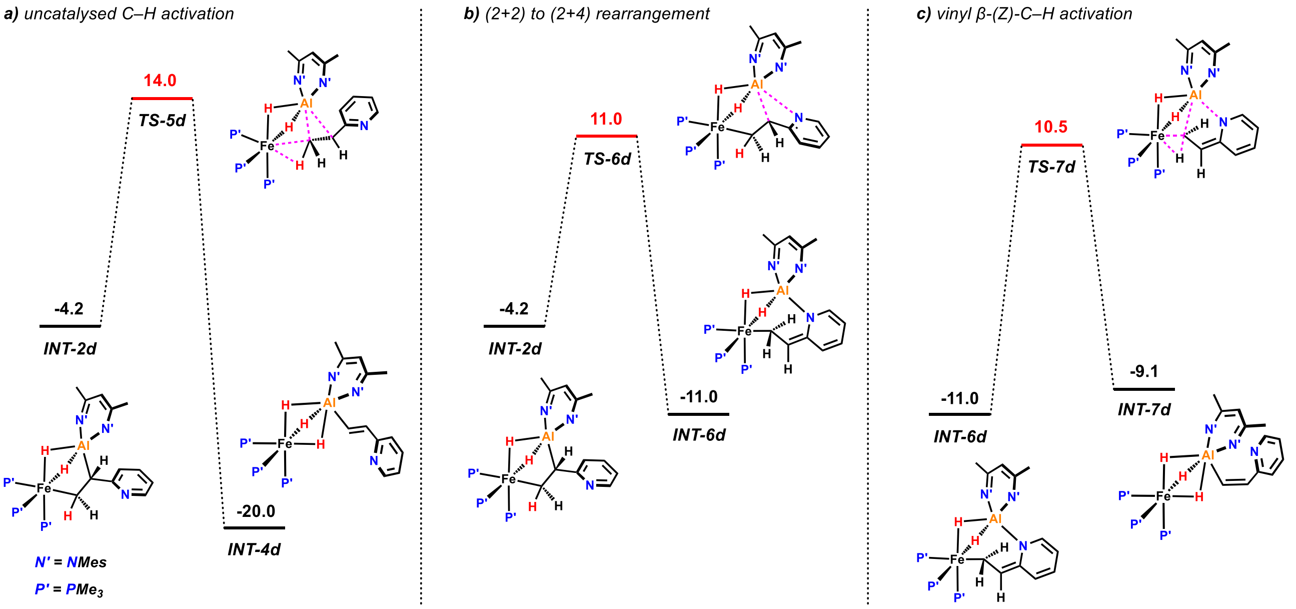

Figure S21. Pathways for the reaction of **1** with 2-vinylpyridine in absence of $MgBr_2$ (ΔG in kcal/mol relative to **1** + 2-vinylpyridine). B3LYP-D3 / Def2-TZVPP/ SDDAll / PCM (benzene) // B3PW91-D3 / 6-31G** / SDDAll / PCM (benzene).

In the uncatalyzed reaction, another transition state **TS-6d** connecting the (2+2) and (2+4) cycloaddition products **INT-2d** and **INT-6d** could be found (Figure S21b). Moreover, the bound substrate in **INT-6d** can potentially undergo a C–H activation through **TS-7d** resulting in the (*Z*)-alumination of the C=C double bond (**INT-7d**, Figure S21c). This intermediate appears to be less stable than **INT-6d** but likely represents the entry to the decomposition pathway observed experimentally.

Other C–H activation pathways for 2-vinylpyridine have been considered as well (Figure S22). In terms of selectivity, the directed activation reactions of the vinylic α-C–H (**TS-8d**) or the pyridine *ortho*-C–H bond (**TS-9d**) through N-coordination of the substrate to Al were found be too high in energy to compete with the cycloaddition pathways. Finally, an alternative transition state for the activation of the vinylic (*E*)-β-C–H bond (**TS-10d**) without pre-coordination of the substrate was found but appears to be inaccessible under thermal conditions.

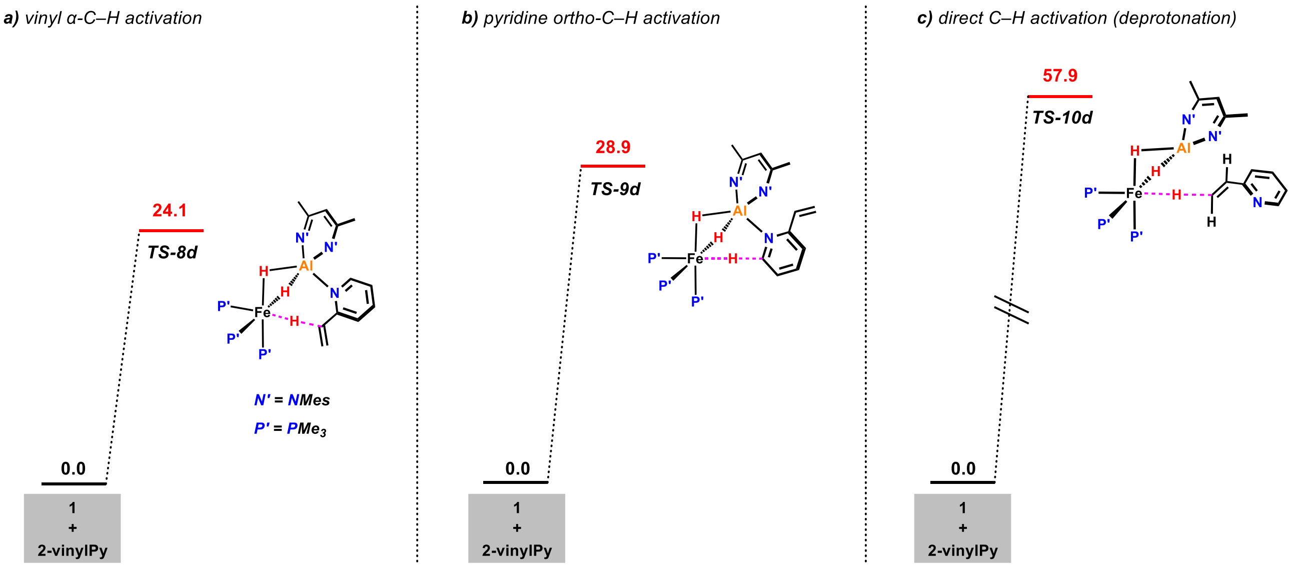

Figure S22. Alternative pathways for the reaction of **1** with 2-vinylpyridine (ΔG in kcal/mol). B3LYP-D3 / Def2-TZVPP/ SDDAll / PCM (benzene) // B3PW91-D3 / 6-31G** / SDDAll / PCM (benzene).

Functional dependence of the IRCs for ***TS-3a-e***

In order to get insight into the functional dependence of the IRCs around **TS-3**, additional calculations at a B3LYP-D3 / 6-31G** / SDDAll (Figure S23) as well as a ωB97X-D / 6-31G** / SDDAll (Figure S24) level of theory were conducted. In both cases, the shapes of the IRCs are different to those obtained with the B3PW91 functional but follow the same trend. With more positive Hammett parameters of the substrates the barriers for the C–H activation step decrease and the potential energy surface around the transition states flattens. As with the B3PW91 functional, transition from a concerted to a stepwise mechanism is obtained for 4-nitrostyrene using the B3LYP functional.

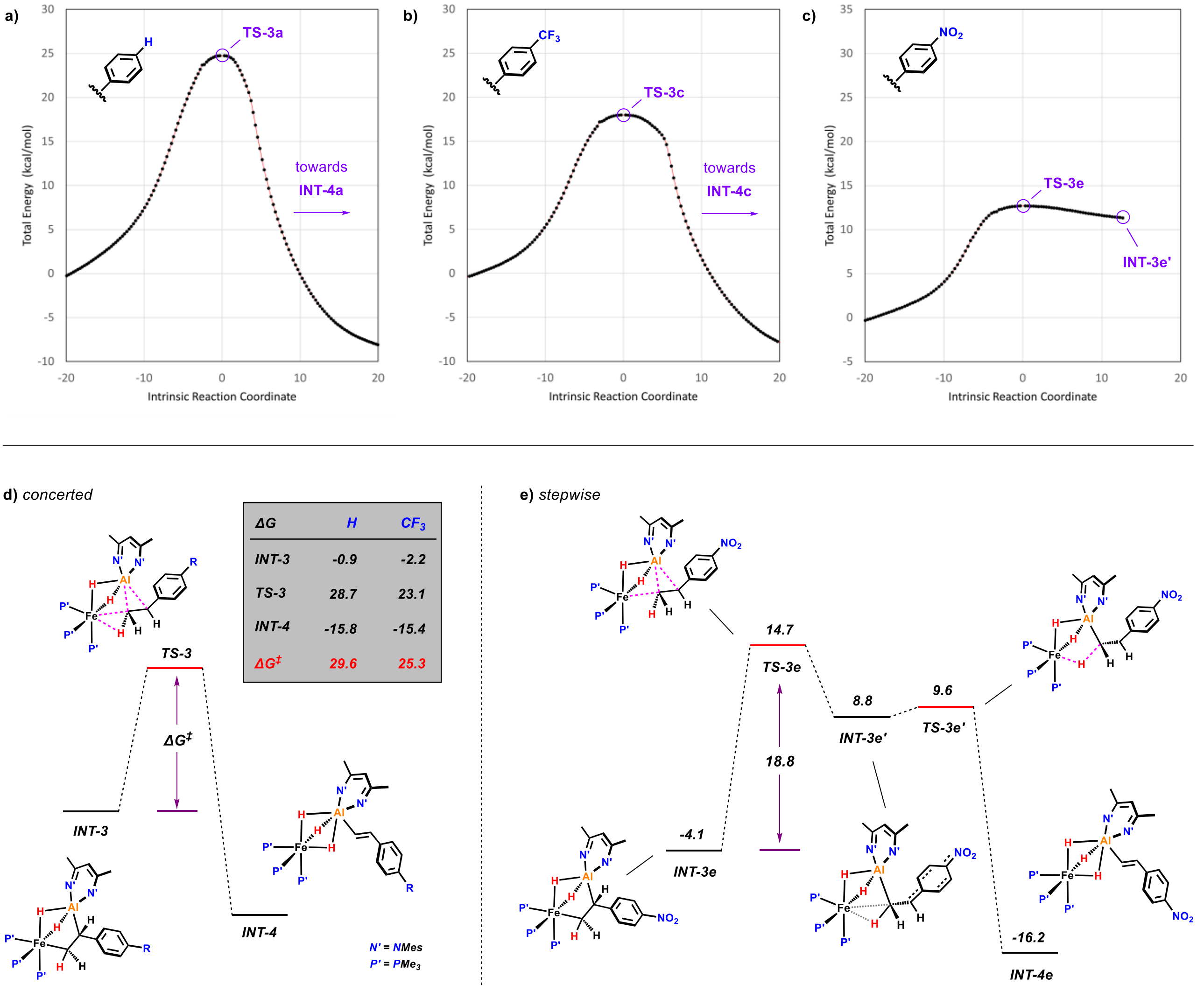

ΔG	H	CF_3
INT-3	-0.9	-2.2
TS-3	28.7	23.1
INT-4	-15.8	-15.4
ΔG‡	29.6	25.3

Figure S23. a-c) Intrinsic reaction coordinates around **TS-3** employing different styrene substrates. Gibbs free energy profile for the concerted (d) and stepwise (e) C–H activation processes (free energies in kcal mol^{-1}). B3LYP-D3 / 6-31G** / SDDAll (Fe,Al).

Even with ωB97X-D, which is not a particularly good functional for the calculation of transition state energies, the trend persists (Figure S24). In these cases, the IRCs are generally extremely flat around the transition states. However, we also observed the transition from a concerted to a stepwise mechanism with the ωB97X-D functional. Slightly different to B3PW91 or B3LYP this transition already happens with 4-trifluoro-methanestyrene.

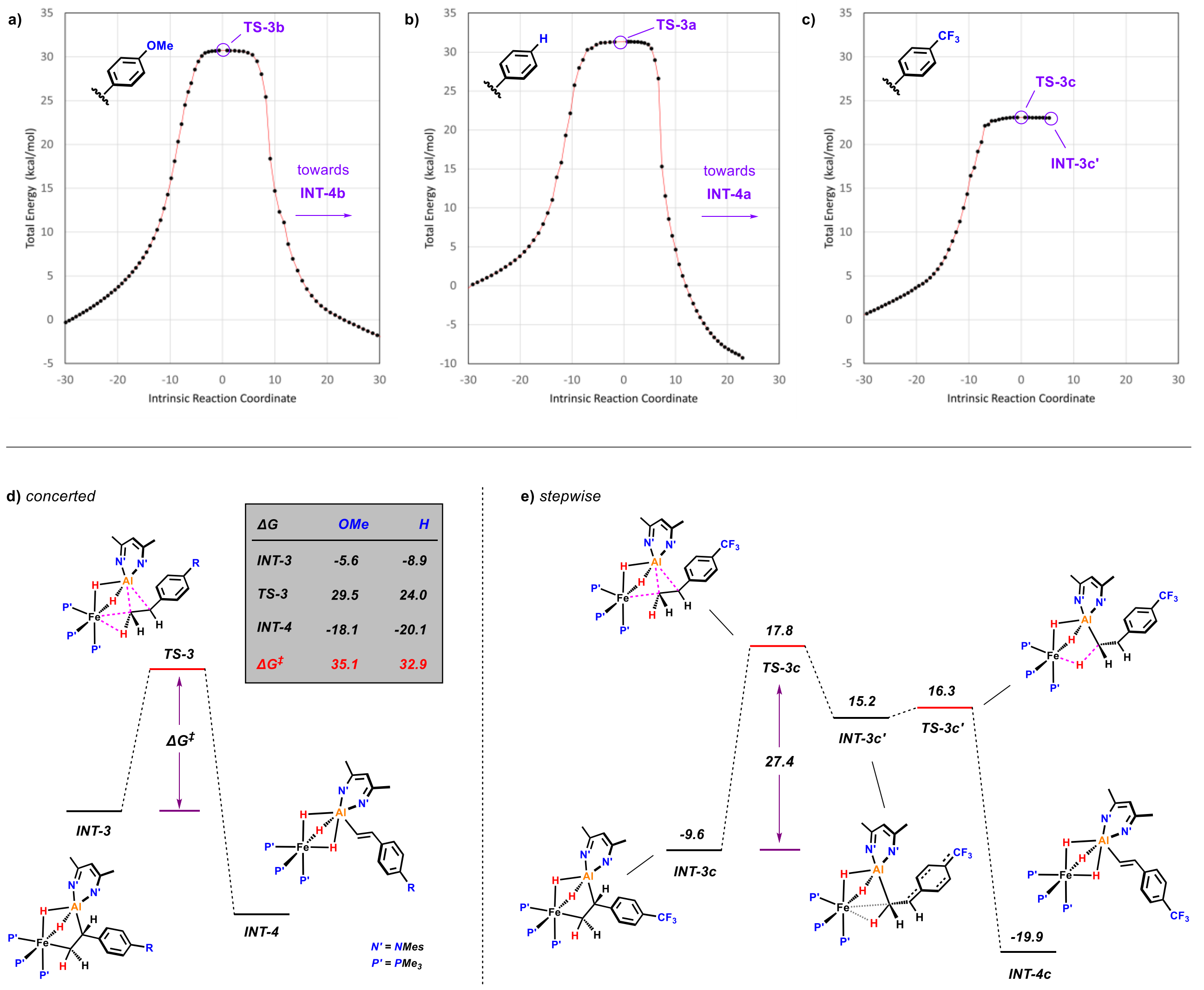

ΔG	OMe	H
INT-3	-5.6	-8.9
TS-3	29.5	24.0
INT-4	-18.1	-20.1
ΔG‡	35.1	32.9

Figure S24. a-c) Intrinsic reaction coordinates around **TS-3** employing different styrene substrates. Gibbs free energy profile for the concerted (d) and stepwise (e) C–H activation processes (free energies in kcal mol^{-1}). ωB97X-D / 6-31G** / SDDAll (Fe,Al).

6. X-Ray Data

Summary of crystal data[a]

compound	2b	3c	4a
CCDC No.	2281162	2281161	2281159
formula	$C_{37}H_{68}AlFeN_2P_3Si$	$C_{41}H_{65}AlF_3FeN_2P_3$	$C_{40}H_{66}AlFeN_2P_3$
solvent		$0.5(C_5H_{12})$	
formula weight	744.76	854.76	750.68
colour, habit	orange prism	orange block	pale yellow needle
temperature / K	173	173	173
crystal system	monoclinic	triclinic	monoclinic
space group	$P2_1/n$ (no. 14)	P-1 (no. 2)	$P2_1/c$ (no. 14)
a / Å	22.3086(5)	12.4178(7)	37.3212(4)
b / Å	18.7698(3)	13.4739(5)	10.93760(13)
c / Å	23.3308(5)	15.1265(9)	21.9575(3)
α / deg	90	91.557(4)	90
β / deg	116.420(3)	101.425(5)	106.7910(13)
γ / deg	90	107.380(4)	90
V / Å^3	8748.9(4)	2357.4(2)	8581.02(19)
Z	8[d]	2	8[d]
*D*c / g cm^{-3}	1.131	1.204	1.185
radiation used	Cu-Kα	Cu-Kα	Cu-Kα
μ / mm^{-1}	4.439	4.043	4.274
no. of unique reflections			
measured, R_{int}	16757, 0.0540	9000, 0.0349	17198, 0.0510
obs, $\|Fo\|>4\sigma(\|Fo\|)$	11278	7554	14877
completeness (%)[b]	98.6	98.1	100
no. of variables	920	564	1237
R_1 (obs), wR_2 (all)[c]	0.0528, 0.1389	0.0308, 0.1074	0.0473, 0.1276

Table S5 Part 1. A summary of the crystallographic data for the structures of **2b, 3c**, and **4a**. [a] Data were collected using a Xcalibur PX Ultra A diffractometer, and the structures were solved and refined using the OLEX2,[28] and SHELX-2019[29] program systems. [b] Completeness to 0.84 Å resolution. [c] $R_1 = \Sigma||F_o| - |F_c||/\Sigma|F_o|$; $wR_2 = \{\Sigma[w(F_o^2 - F_c^2)^2] / \Sigma[w(F_o^2)^2]\}^{1/2}$; $w^{-1} = \sigma_2(F_o^2) + (aP)^2 + bP$. [d] The asymmetric unit contains two independent molecules.

compound	4d	5d	8c
**CCDC No.**	2281160	2281163	2314166
**formula**	$C_{39}H_{65}AlFeN_3P_3$	$C_{36}H_{64}AlFeN_2O_2P_3$	$C_{21}H_{16}F_3P_1S_1$
**solvent**		$0.5(C_5H_{12})$	
**formula weight**	751.68	768.70	388.37
**colour, habit**	pale yellow tabloid	pale orange tablet	colourless needle
**temperature / K**	173	173	173
**crystal system**	monoclinic	monoclinic	monoclinic
**space group**	$P2_1/c$ (no. 14)	$P2_1/c$ (no. 14)	$P2_1/c$ (no. 14)
a **/ Å**	37.1111(8)	20.3189(3)	11.2905(2)
b **/ Å**	10.91765(18)	14.4405(2)	9.6234(2)
c **/ Å**	21.9409(4)	15.7233(2)	17.7428(4)
**α / deg**	90	90	90
**β / deg**	106.654(2)	112.393(2)	94.935(2)
**γ / deg**	90	90	90
V **/ Å^3**	8516.8(3)	4265.56(12)	1920.66(7)
Z	8[d]	4	4
Dc **/ g cm^{-3}**	1.172	1.197	1.343
**radiation used**	Cu-Kα	Cu-Kα	Cu-Kα
**μ / mm^{-1}**	4.314	4.344	2.550
**no. of unique reflections**			
**measured,** R_{int}	19260, 0.2206	8242, 0.0243	3691, 0.0443
**obs,** $\|Fo\|>4\sigma(\|Fo\|)$	13865	7259	3073
**completeness (%)**[b]	99.0	98.8	98.7
**no. of variables**	1212	479	271
R_1 **(obs),** wR_2 **(all)**[c]	0.0753, 0.2731	0.0361, 0.0964	0.0486, 0.1302

Table S5 Part 2. A summary of the crystallographic data for the structures of **4d**, **5d**, and **8c**.

compound	S1	S2
**CCDC No.**	2314167	2281164
**formula**	$C_{37}H_{68}Al_1Fe_1N_2P_3Si_1$	$C_{27}H_{35}AlN_2O_2$
**solvent**	0.5(C_5H_{12})	
**formula weight**	762.80	446.55
**colour, habit**	pale yellow column	pale brown block
**temperature / K**	173	173
**crystal system**	triclinic	trigonal
**space group**	*P*-1 (no. 2)	*R*-3 (no. 148)
a **/ Å**	13.5879(3)	25.5478(4)
b **/ Å**	17.5340(6)	25.5478(4)
c **/ Å**	19.4928(6)	20.6525(5)
**α / deg**	86.469(3)	90
**β / deg**	87.201(2)	90
**γ / deg**	82.429(2)	120
**V / Å^3**	4591.0(2)	11673.7(5)
Z	4[d]	18
**Dc / g cm^{-3}**	1.197	1.143
**radiation used**	Cu-Kα	Cu-Kα
**μ / mm^{-1}**	4.344	0.867
**no. of unique reflections**		
**measured, R_{int}**	8242, 0.0243	5038, 0.0243
**obs, $\|Fo\|>4\sigma(\|Fo\|)$**	7259	3949
**completeness (%)[b]**	98.8	99.2
**no. of variables**	479	298
**R_1 (obs), wR_2 (all)[c]**	0.0361, 0.0964	0.0461, 0.1359

Table S5 Part 3. A summary of the crystallographic data for the structures of **S1** and **S2**.

Refinement details

*The X-ray structure of **2b***

The crystal structure of **2b** has two independent molecules in the asymmetric unit. All three PMe_3 groups of the Fe1A-based molecule were found to be disordered across two orientations using the same free variable at a ratio of 0.62:0.38. The two geometries restrained to be similar and for all three, only the methyl groups were modelled as disordered (they are sharing the same P atoms). Only the C atoms of the major orientation were modelled anisotropically (the rest were modelled isotropically). The four Fe–μ-**H**–Al hydrides and the SiCC-**H** atoms were found in the Δ*F* maps and freely refined.

*The X-ray structure of **3c***

The crystal was found to contain 0.5 pentane solvent molecules per bimetallic complex, disordered across a centre of inversion located ca. 0.61 Å away from the central carbon of the solvent molecule. The best model was obtained by modelling the pentane molecule at 0.5 occupancy in one symmetry-unique orientation (plus its symmetry-related counterpart), with the C atoms being anisotropic. Modelling multiple symmetry unique orientations of the solvent resulted in an unstable model, and using SQUEEZE to mask it gave a worse model. The CF_3 group was found to be rotationally disordered, the best model was obtained by modelling the set of three F atoms at two orientations at a ratio of ca. 0.71:0.29, the two geometries restrained to be similar, and with both major and minor occupancies modelled anisotropically. The two Fe–μ-**H**–Al hydrides were found in the Δ*F* maps and freely refined.

*The X-ray structure of **4a***

The crystal of **4a** was found to be a two-component merohedral twin in a ca. 0.83:0.17 ratio with the two lattices related by the [1.00 0.00 1.00 0.00 -1.00 0.00 0.00 0.00 -1.00] twin law. The asymmetric unit contains two independent molecules, both of which are disordered across two orientations. As their independent refinement yielded very similar ratios for the two orientations and because both minor occupancy orientations clash with the other major occupancy orientation, they were both modelled using the same free variable at a ratio of ca. 0.93:0.07. The two geometries were restrained to be similar, and in both cases, only the non-hydrogen atoms of the major orientation were modelled anisotropically, the rest were modelled isotropically. All six symmetry independent Fe–μ-**H**–Al hydrides in the major orientations were found in the Δ*F* maps and freely refined. The hydrides of the minor orientations could not be modelled reliably, and so the atom list for the asymmetric unit is low by ca. 0.44 hydrogen atoms, and that for the unit cell as a whole is low by ca. 1.74 hydrogen atoms.

*The X-ray structure of **4d***

The crystal structure of **4d** is isomorphous with that of **4a.**

The crystal of **4d** was found to be two-component twin in a ca. 0.70:0.30 ratio with the two lattices related by the [1.00 0.00 0.00 0.00 -1.00 0.00 -0.97 0.00 -1.00] twin law. The asymmetric unit contains two independent molecules, both of which are disordered across two orientations. As their independent refinement yielded very similar ratios for the two orientations and because both minor occupancy orientations clash with the other major occupancy orientation, they were both modelled using the same free variable at a ratio of ca. 0.92:0.08. The two geometries were restrained to be similar, and in both cases, only the non-hydrogen atoms of the major orientation were modelled anisotropically, the rest were modelled isotropically. Though some of the six total Fe–μ-**H**–Al hydrides were found in the ΔF maps they could not be refined reliably, and so they were omitted making the atom list for the asymmetric unit low by 6 hydrogen atoms, and that for the unit cell as a whole low by 24.

*The X-ray structure of **5d***

The crystal structure of **5d** was found to contain 0.5 pentane solvent molecules per asymmetric unit, disordered across a centre of inversion located ca. 1.1 Å away from the central carbon of the molecule. Similarly to **4c**, the best results were obtained by modelling the included solvent at 0.5 occupancy at one symmetry-unique orientation (plus its symmetry-related counterpart), with the C atoms being anisotropic. The two Fe–μ-**H**–Al hydrides were found in the Δ*F* maps and freely refined.

*The X-ray structure of **8c***

The CF_3 group was found to be disordered across three separate orientations at a ratio of ca. 0.60:0.21:0.19. The three orientations were restrained to be similar, and only the major orientation was modelled anisotropically (the two minor orientations were modelled isotropically).

*The X-ray structure of **S1***

The crystal structure of **S1** contains 0.5 equivalent of pentane solvent per asymmetric unit (0.25 per main molecule), disordered across a centre of inversion located ca. 0.3 A away from the central carbon. Similarly to **4c**, the best results were obtained by modelling it in one symmetry unique orientation (and its symmetry related counterpart), with all C atoms being anisotropic. The Si1A-based trimethylsilyl group was found to be disordered. Only the methyl groups were modelled in two orientations (they are sharing the same Si atom) at a ratio of ca. 0.75:0.25. For both of the above disorders, the two orientations were restrained to be similar. All six Fe–μ-**H**–Al hydrides were found in the Δ*F* maps and freely refined.

Crystal structures

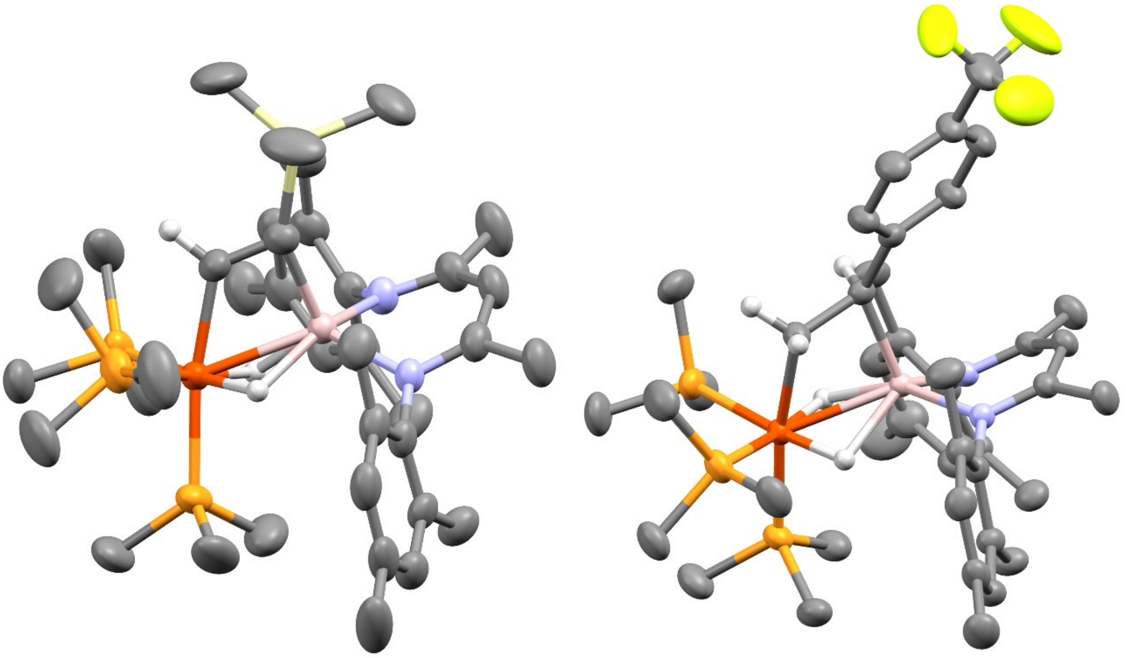

Figure S25. Left: Crystal structure of **2a**. Thermal ellipsoids are shown at a 50 % probability level. Most hydrogens and the second independent molecule in the asymmetric unit are hidden for clarity. Right: Crystal structure of **3c**. Thermal ellipsoids are shown at a 50 % probability level. Most hydrogens, atoms in the minor occupancy orientation, and the included pentane solvent molecule are hidden for clarity.

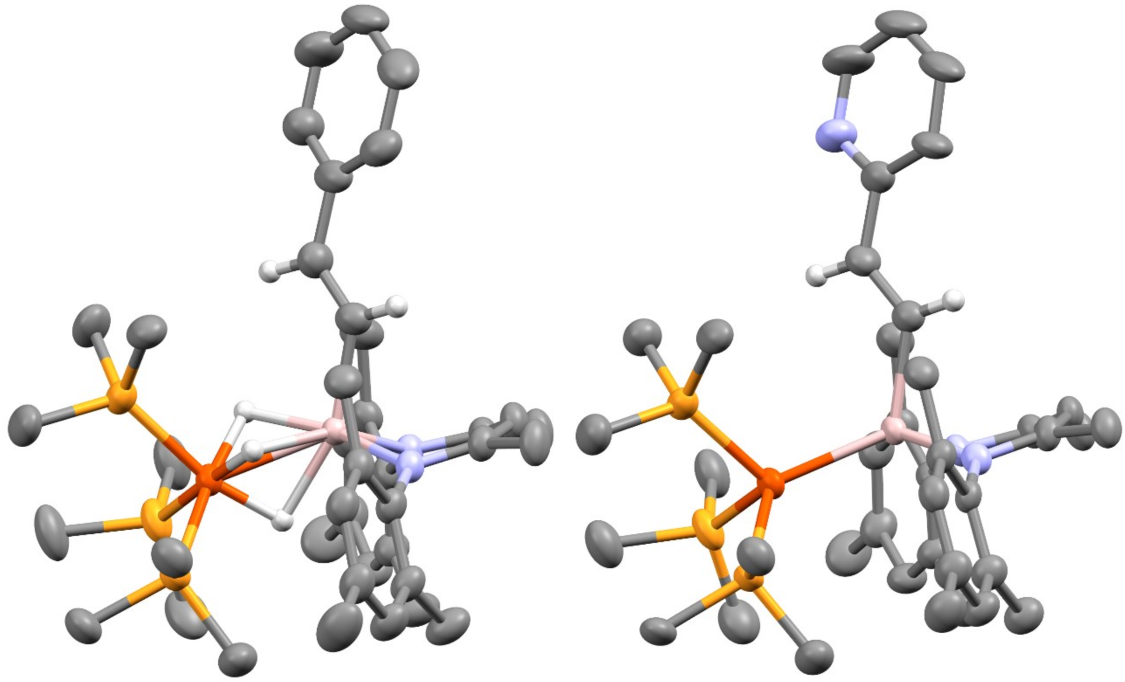

Figure S26. Left: Crystal structure of **4a**. Thermal ellipsoids are shown at a 50 % probability level. Most hydrogens, the second independent molecule in the asymmetric unit, and the minor occupancy orientation of the molecule are hidden for clarity. Right: Crystal structure of **4d**. Thermal ellipsoids are shown at a 50 % probability level. Most hydrogens, the second independent molecule in the asymmetric unit, and the minor occupancy orientation of the molecule are hidden for clarity. The Fe–μ-**H**–Al hydrides could not be refined reliably for this structure.

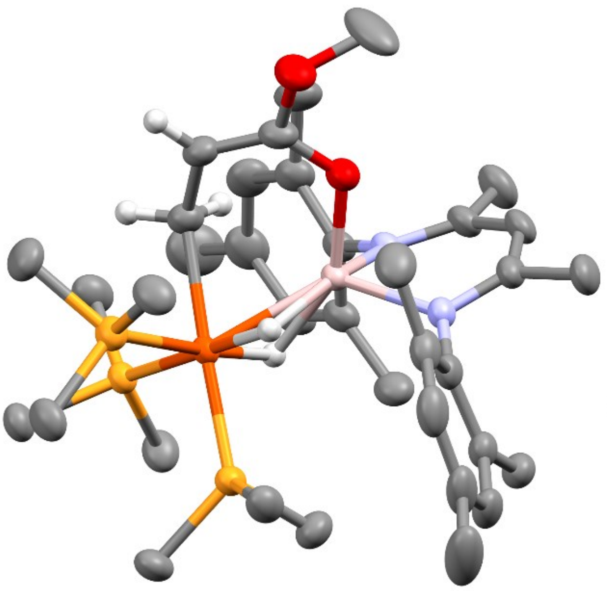

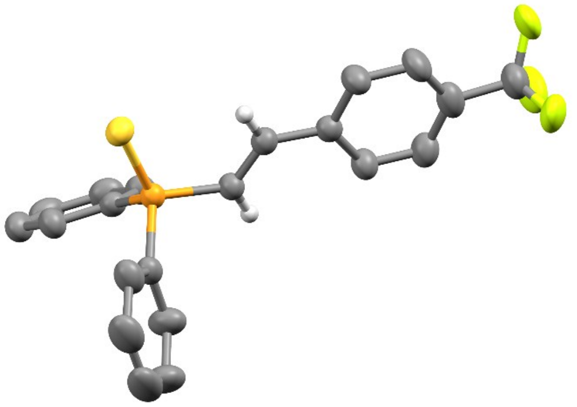

Figure S27. Left: Crystal structure of **5d**. Thermal ellipsoids are shown at a 50 % probability level. Most hydrogens and atoms and the included pentane solvent molecule are hidden for clarity. Right: Crystal structure of **8c**. Thermal ellipsoids are shown at a 50 % probability level. Hydrogens are hidden for clarity.

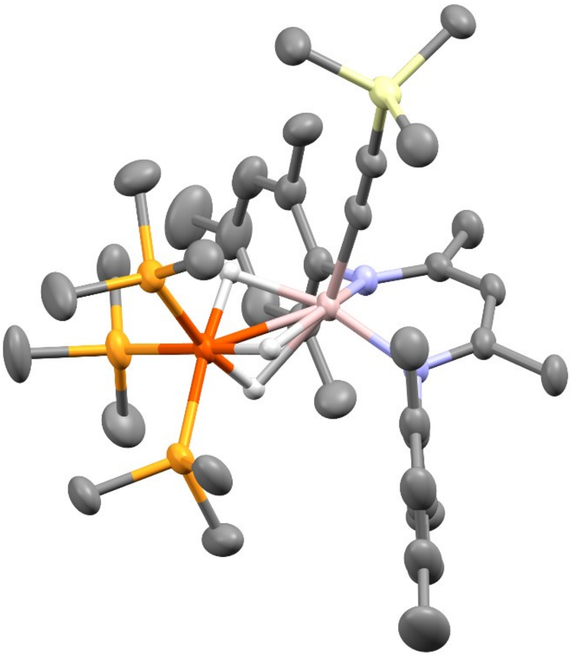

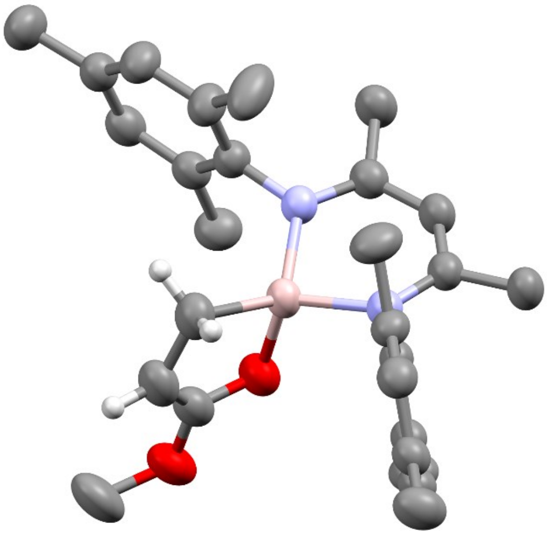

Figure S28. Left: Crystal structure of **S1**. Thermal ellipsoids are shown at a 50 % probability level. Most hydrogens and atoms, the second molecule in the asymmetric unit and he included pentane solvent molecule are hidden for clarity. Right: Crystal structure of **S2**. Thermal ellipsoids are shown at a 50 % probability level. Most hydrogens are hidden for clarity.

7. NMR Spectra of new Compounds

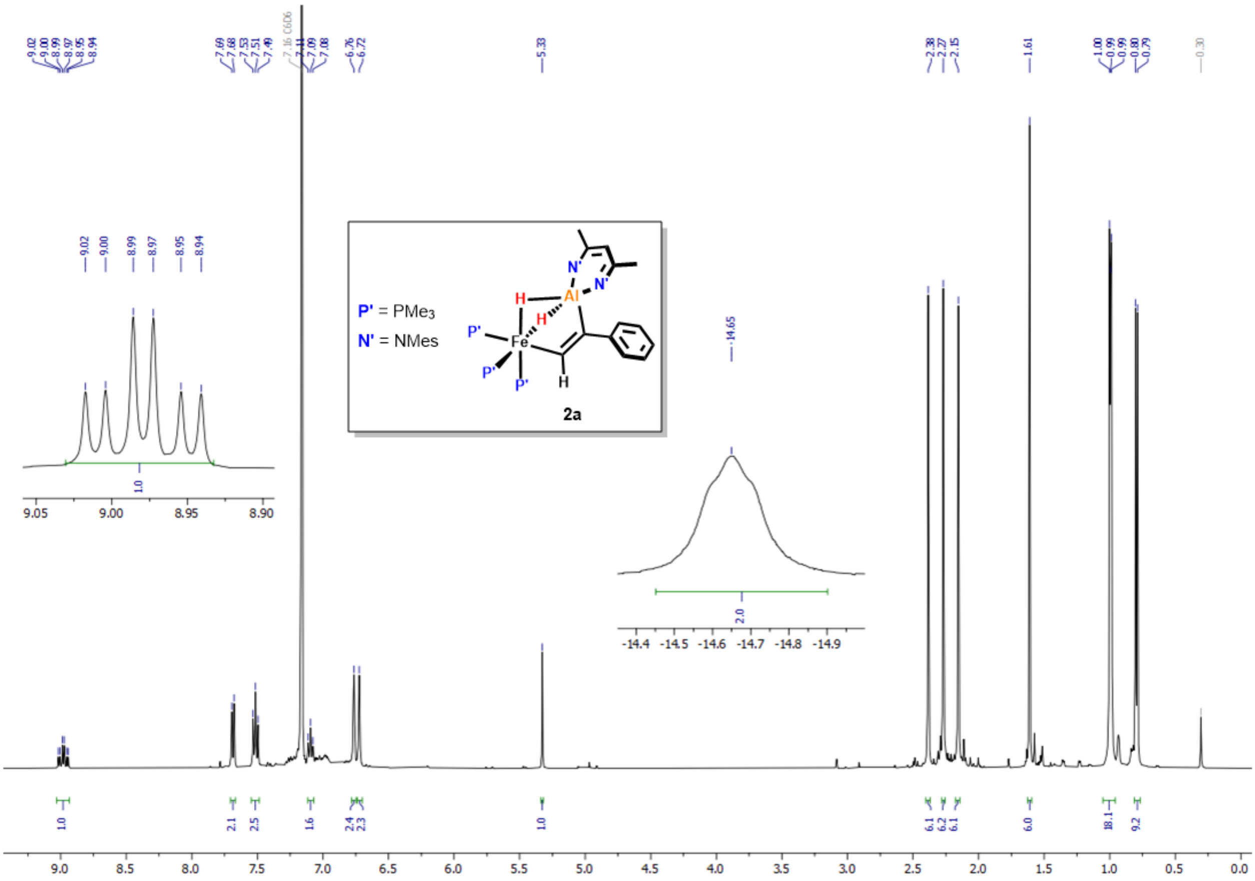

Figure S29. ^{1}H NMR of **2a** (400 MHz, C_6D_6, 298 K).

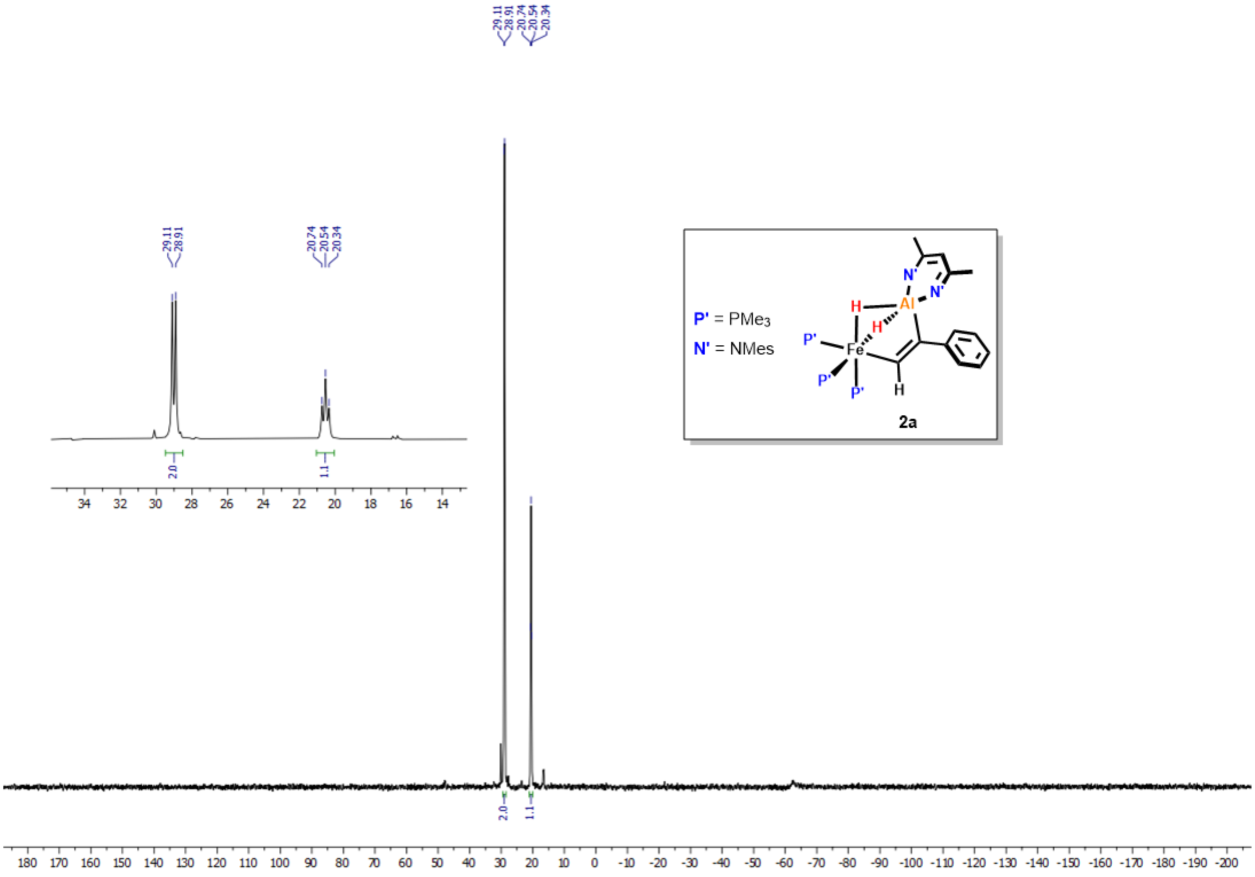

Figure S30. ^{31}P{^{1}H} NMR of **2a** (162 MHz, C_6D_6, 298 K).

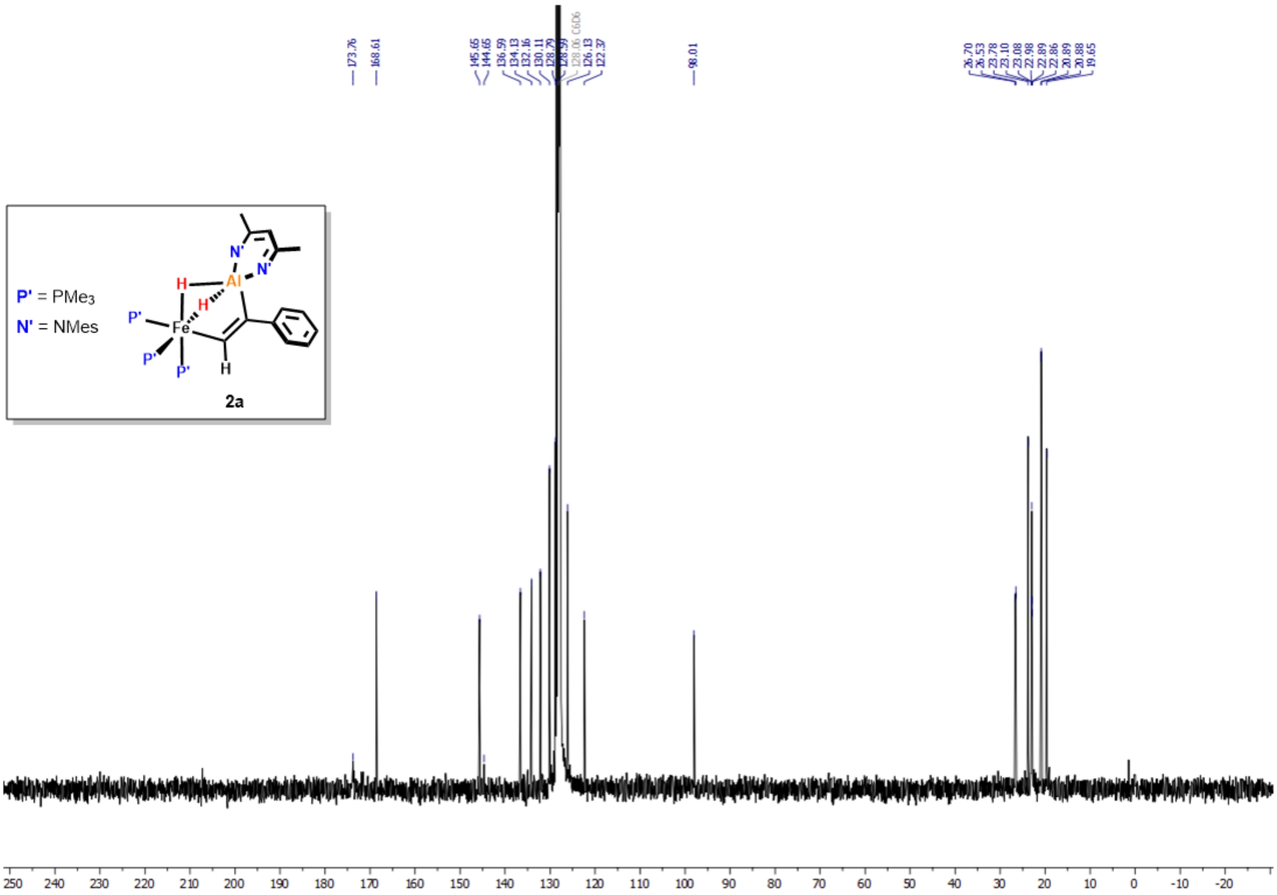

Figure S31. $^{13}C\{^{1}H\}$ NMR of **2a** (101 MHz, C_6D_6, 298 K).

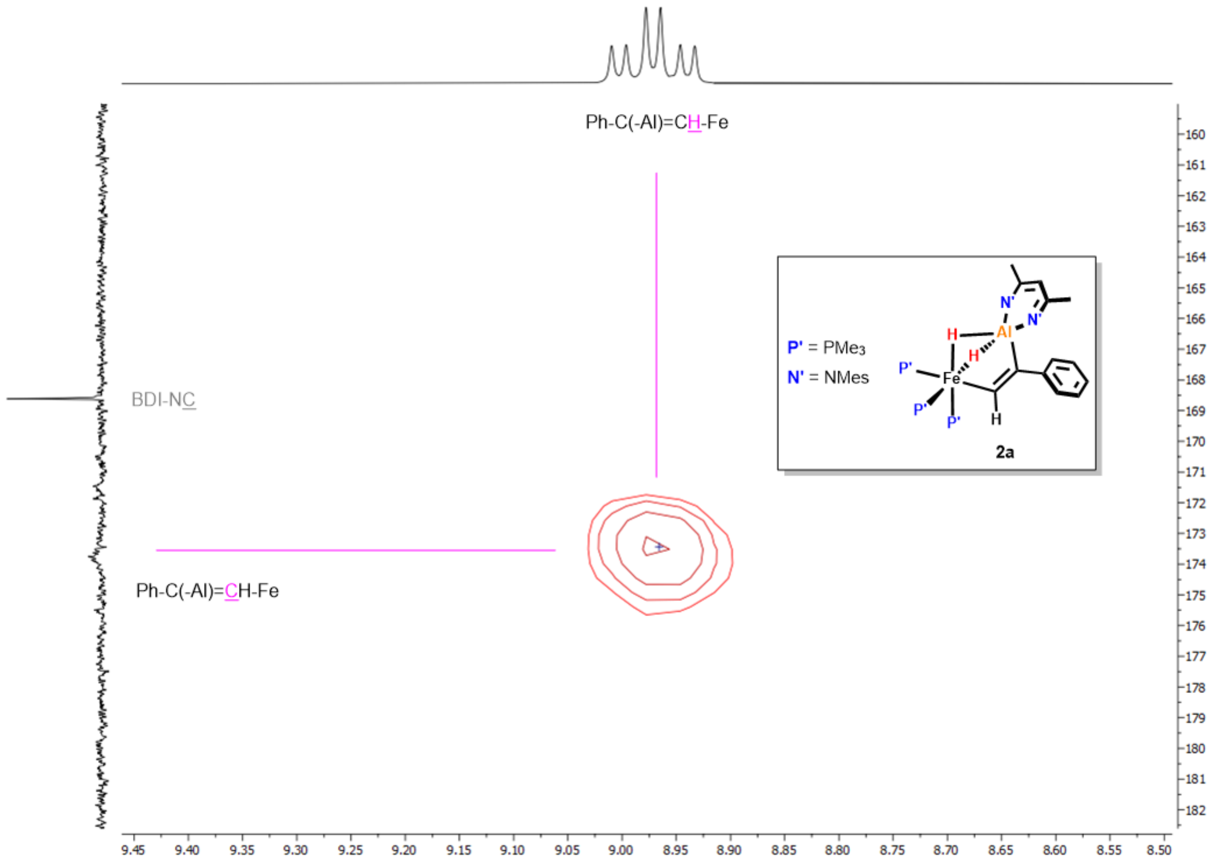

Figure S32. ^{1}H-^{13}C HSQC NMR of **2a** (400 MHz, C_6D_6, 298 K).

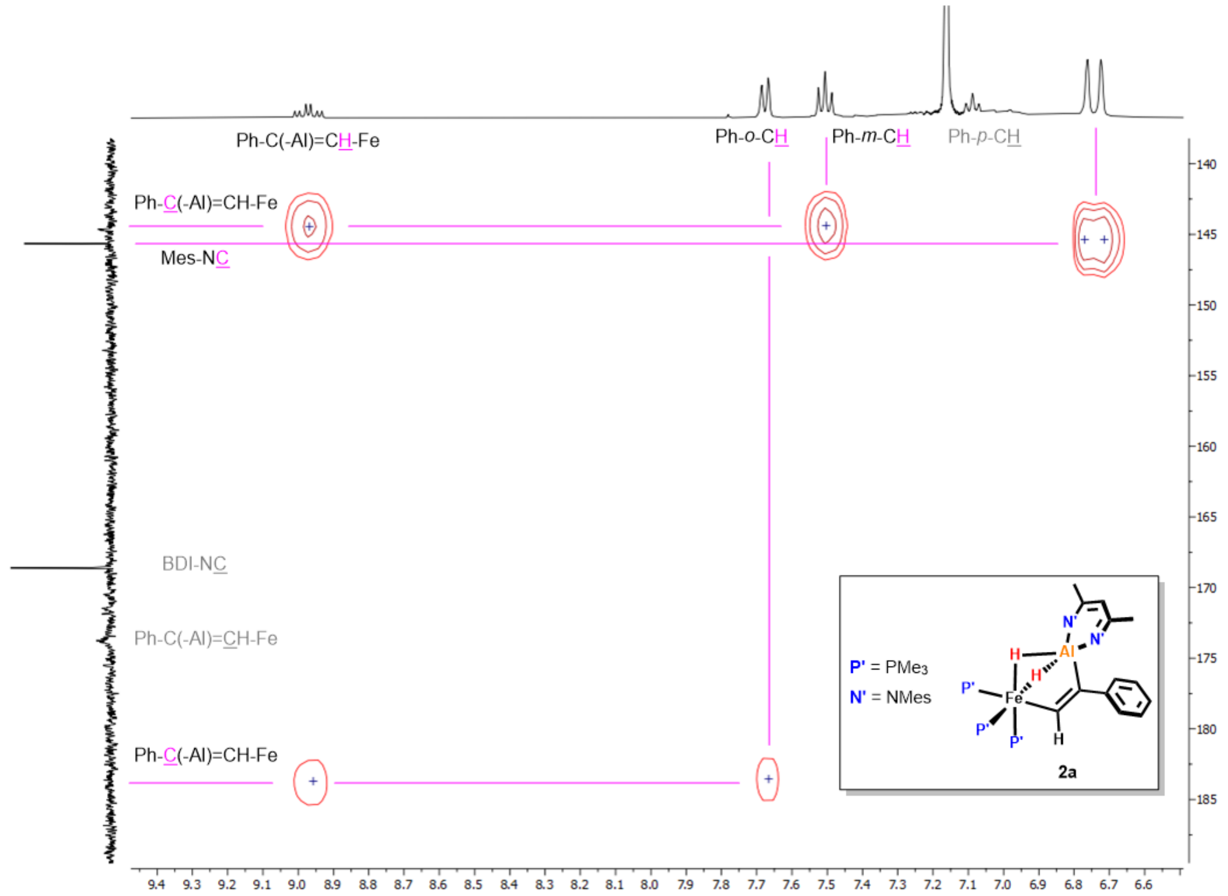

Figure S33. ^{1}H-^{13}C HMBC NMR of **2a** (400 MHz, C_6D_6, 298 K).

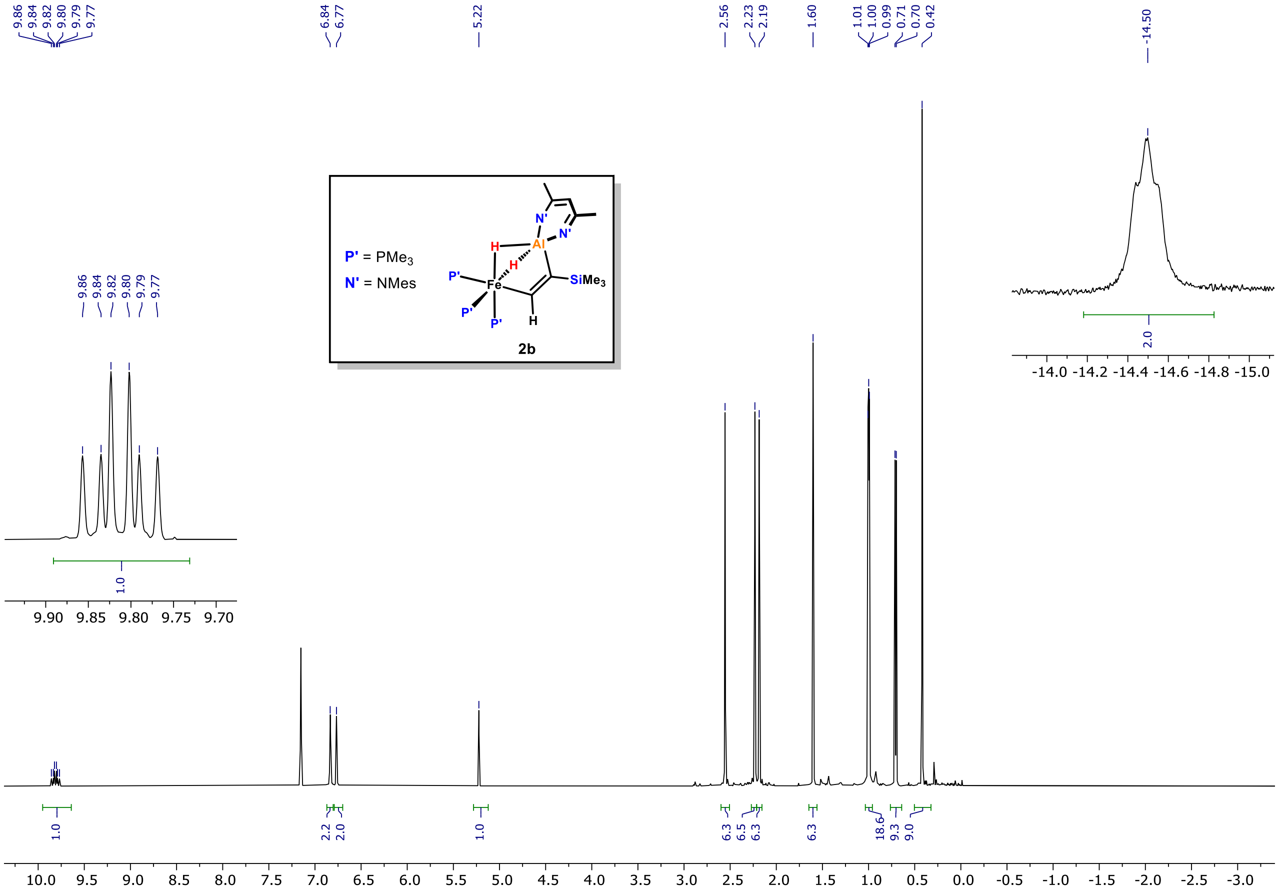

Figure S34. ^{1}H NMR of **2b** (400 MHz, C_6D_6, 298 K).

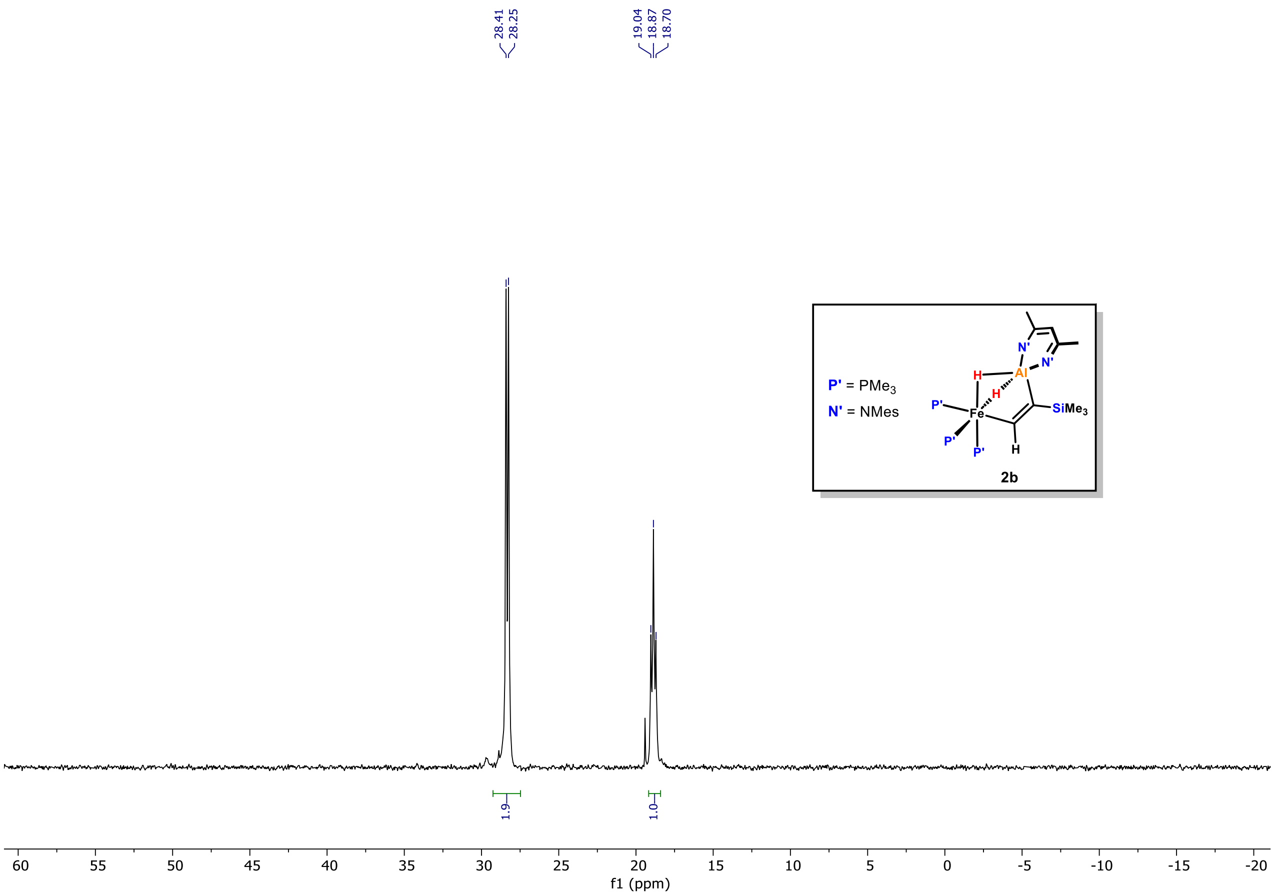

Figure S35. $^{31}P\{^1H\}$ NMR of **2b** (162 MHz, C_6D_6, 298 K).

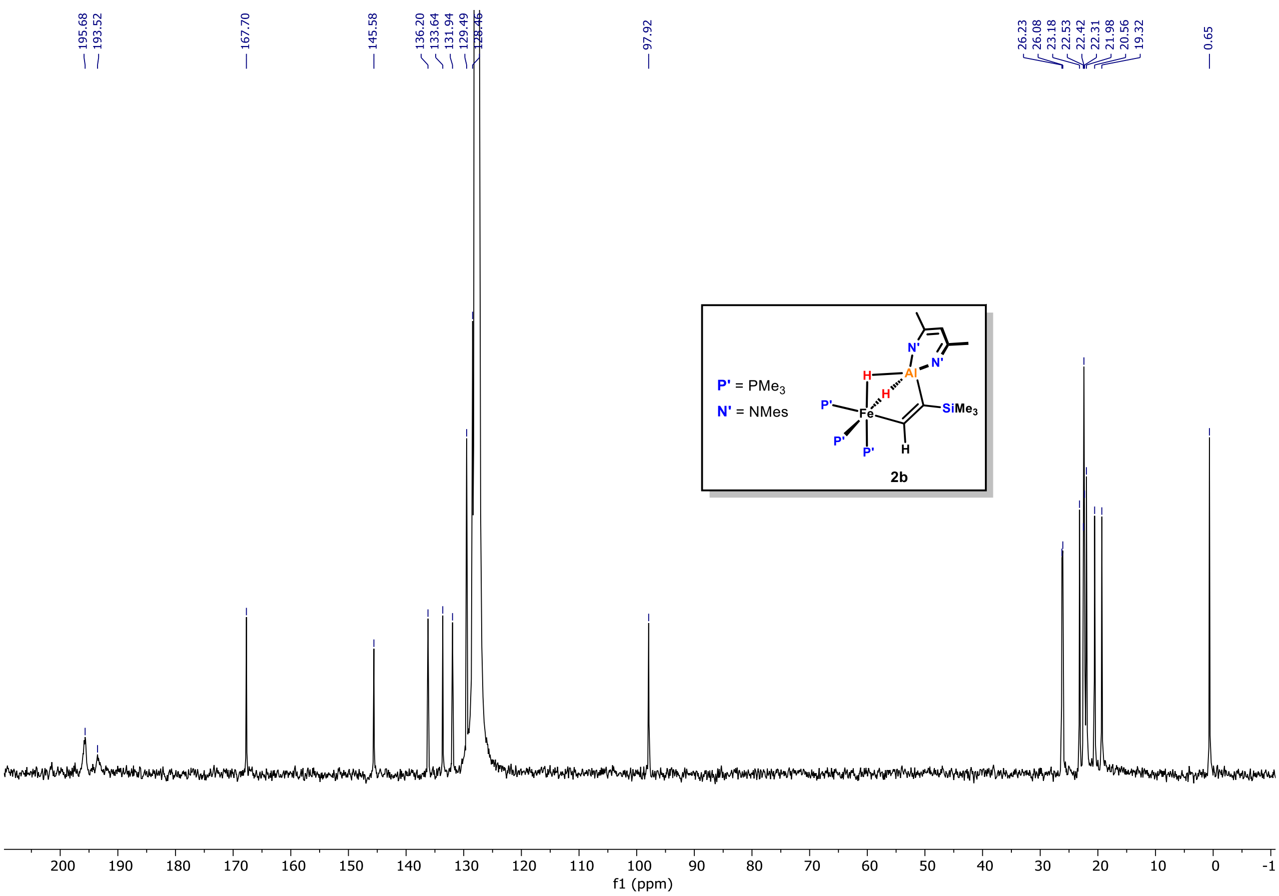

Figure S36. $^{13}C\{^1H\}$ NMR of **2b** (101 MHz, C_6D_6, 298 K).

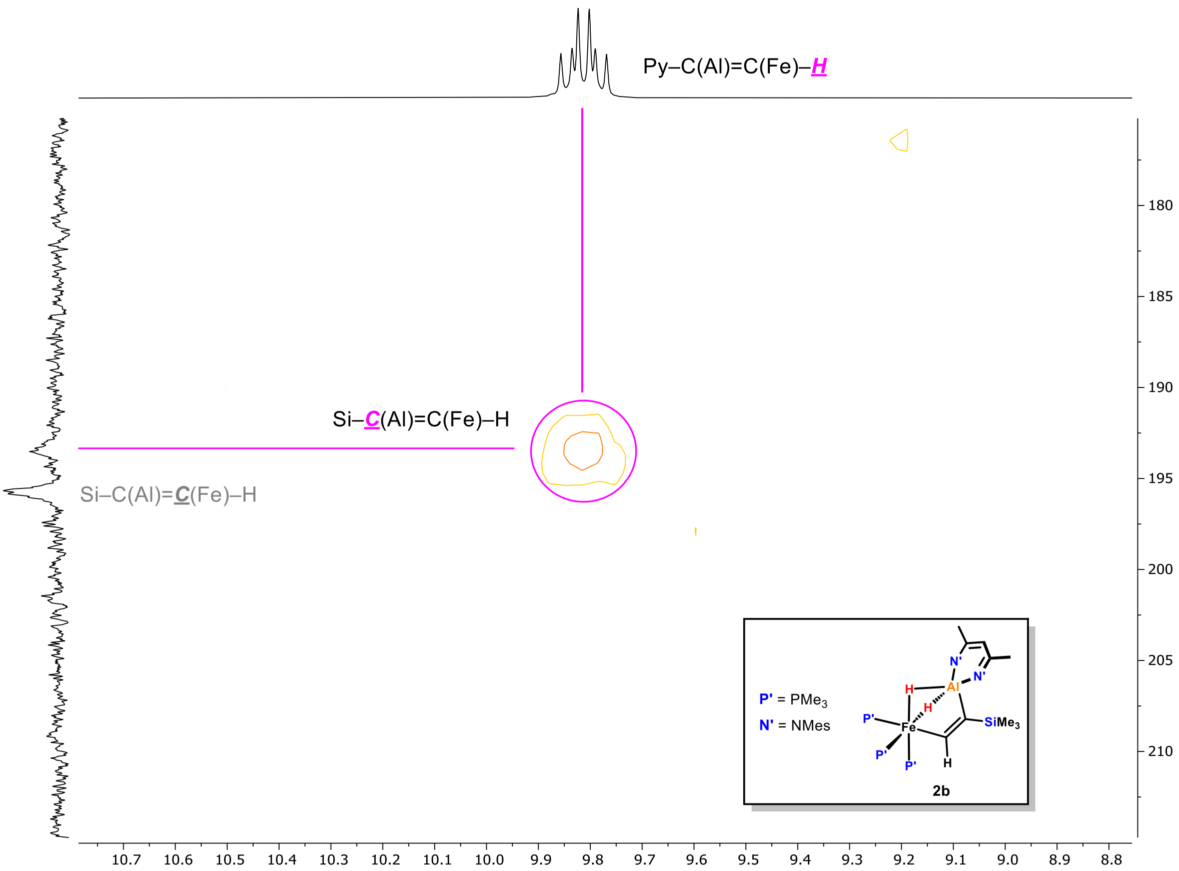

Figure S37. ^{1}H/^{13}C-HMBC NMR of **2b** (400 MHz, C_6D_6, 298 K).

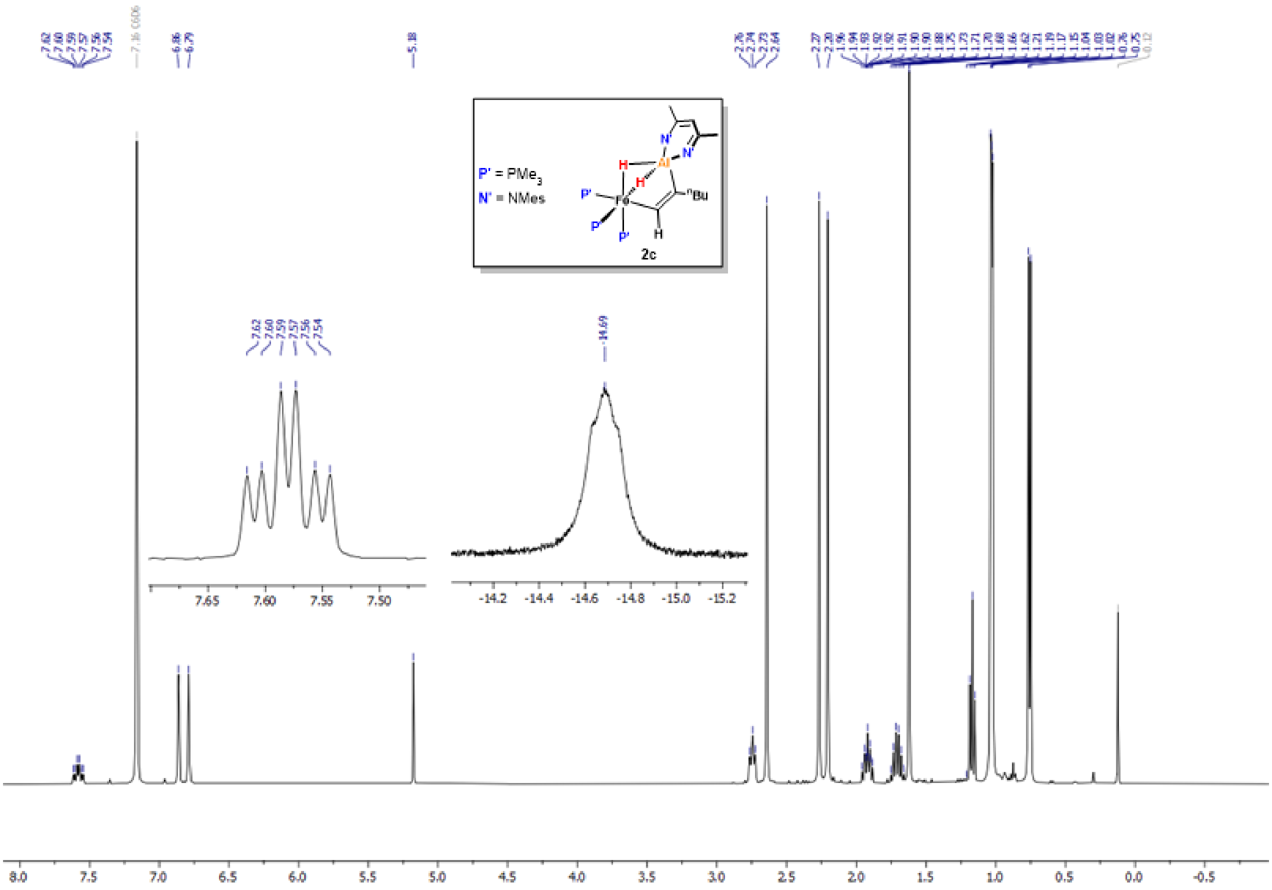

Figure S38. ^{1}H NMR of **2c** (400 MHz, C_6D_6, 298 K).

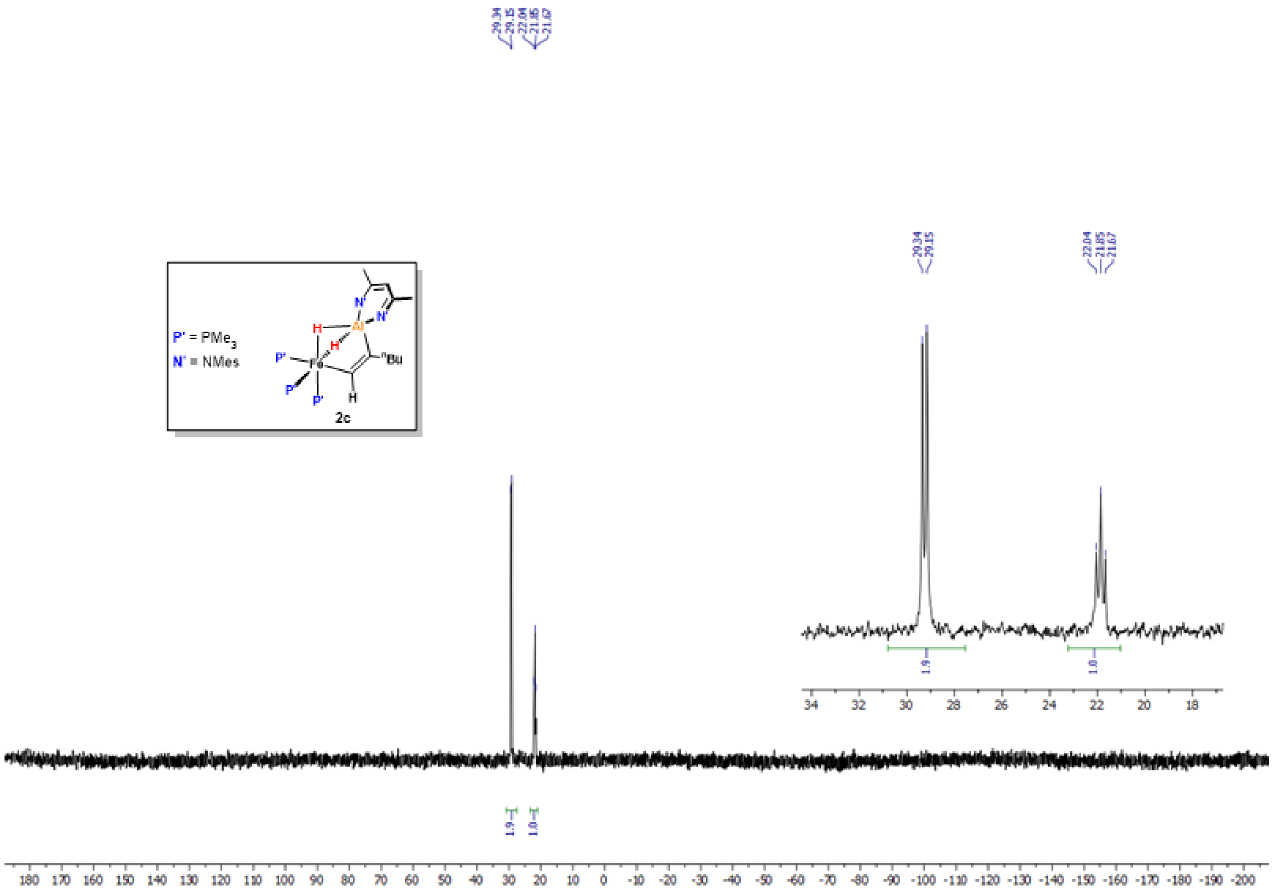

Figure S39. $^{31}P\{^{1}H\}$ NMR of **2c** (162 MHz, C_6D_6, 298 K).

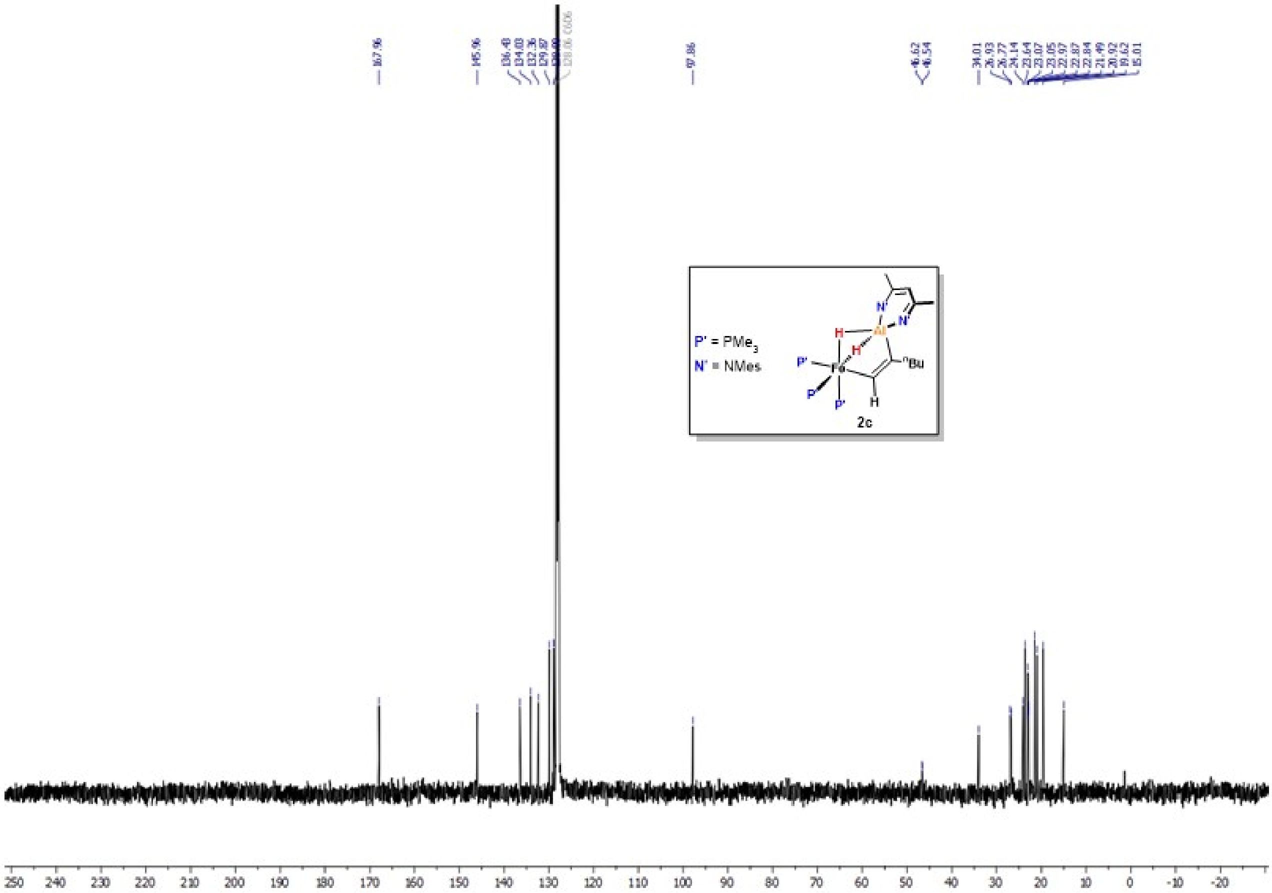

Figure S40. $^{13}C\{^{1}H\}$ NMR of **2c** (101 MHz, C_6D_6, 298 K).

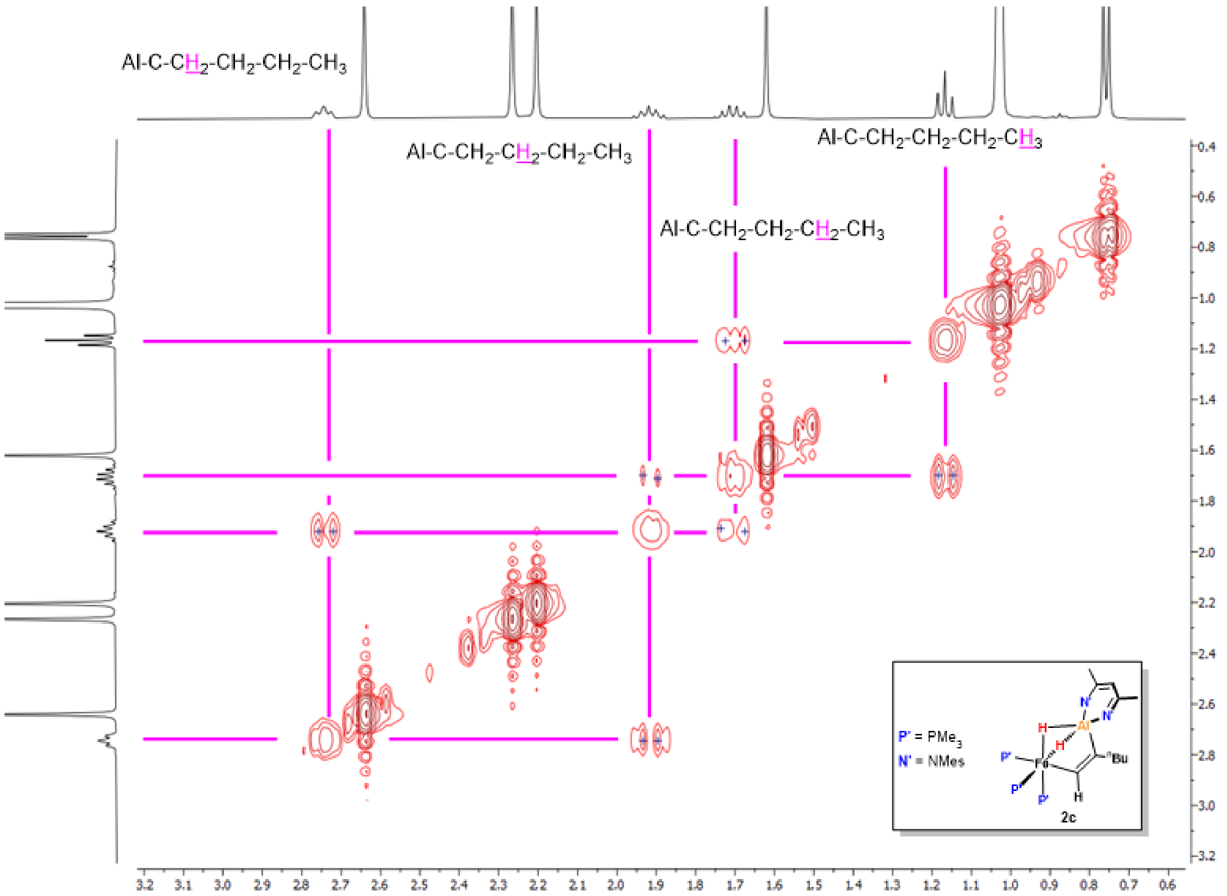

Figure S41. ^{1}H-^{1}H COSY NMR of **2c** (101 MHz, C_6D_6, 298 K).

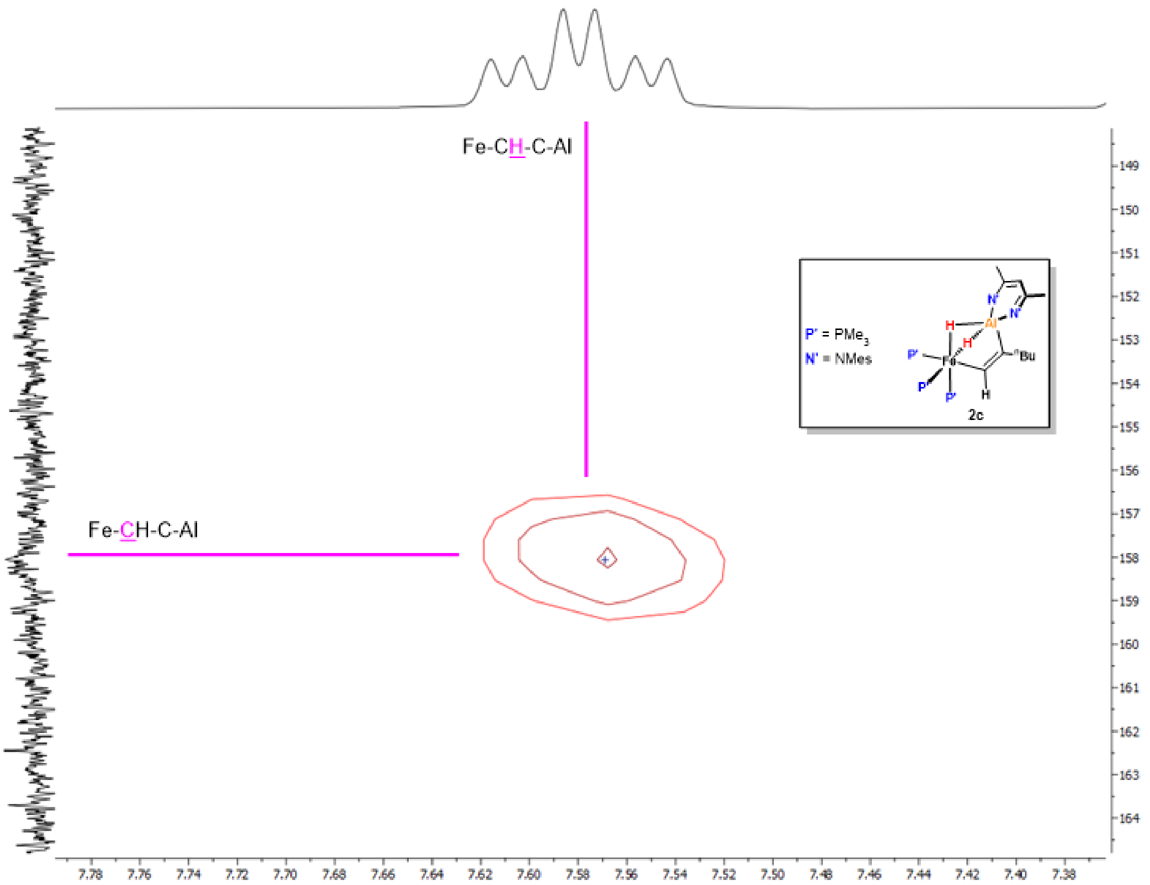

Figure S42. ^{13}C-^{1}H HSQC NMR of **2c** (C_6D_6, 298 K).

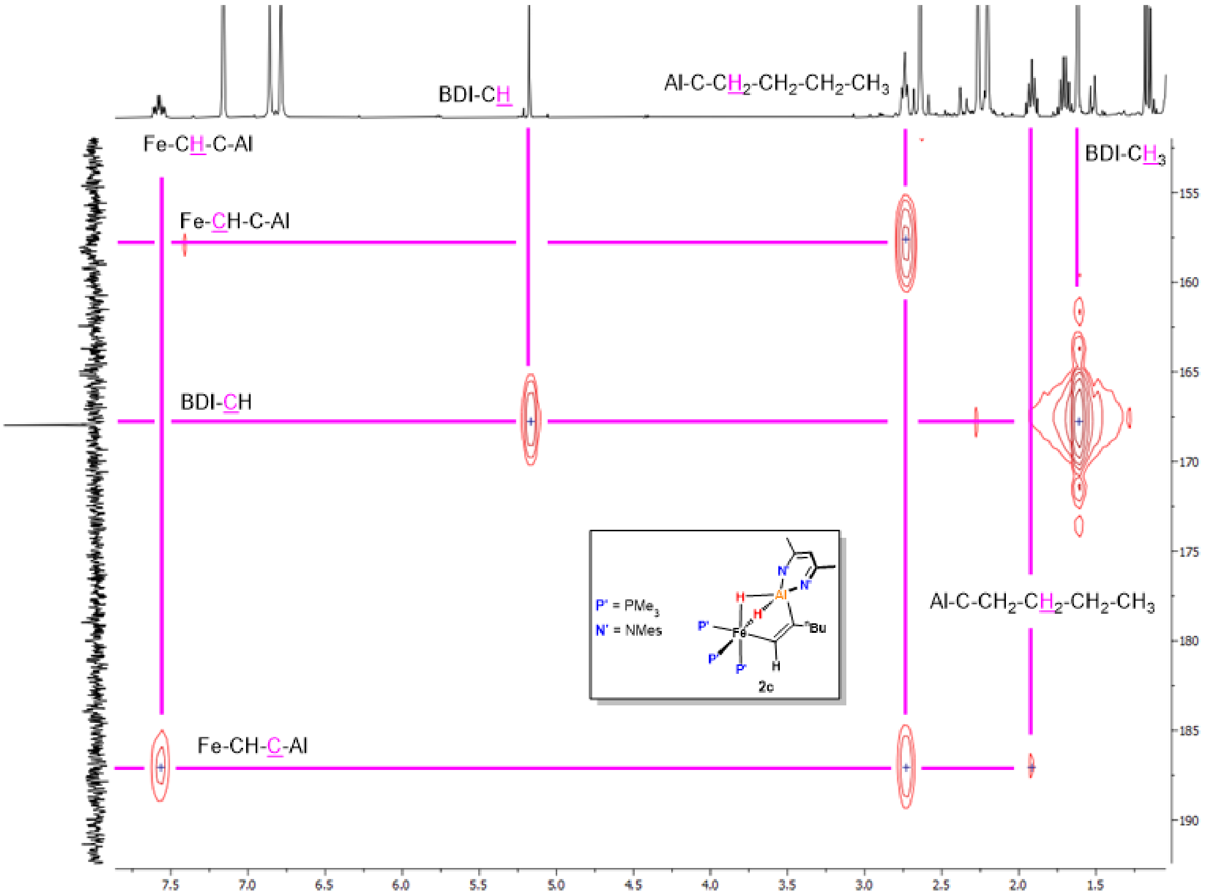

Figure S43. ^{13}C-^{1}H HMBC NMR of **2c** (C_6D_6, 298 K).

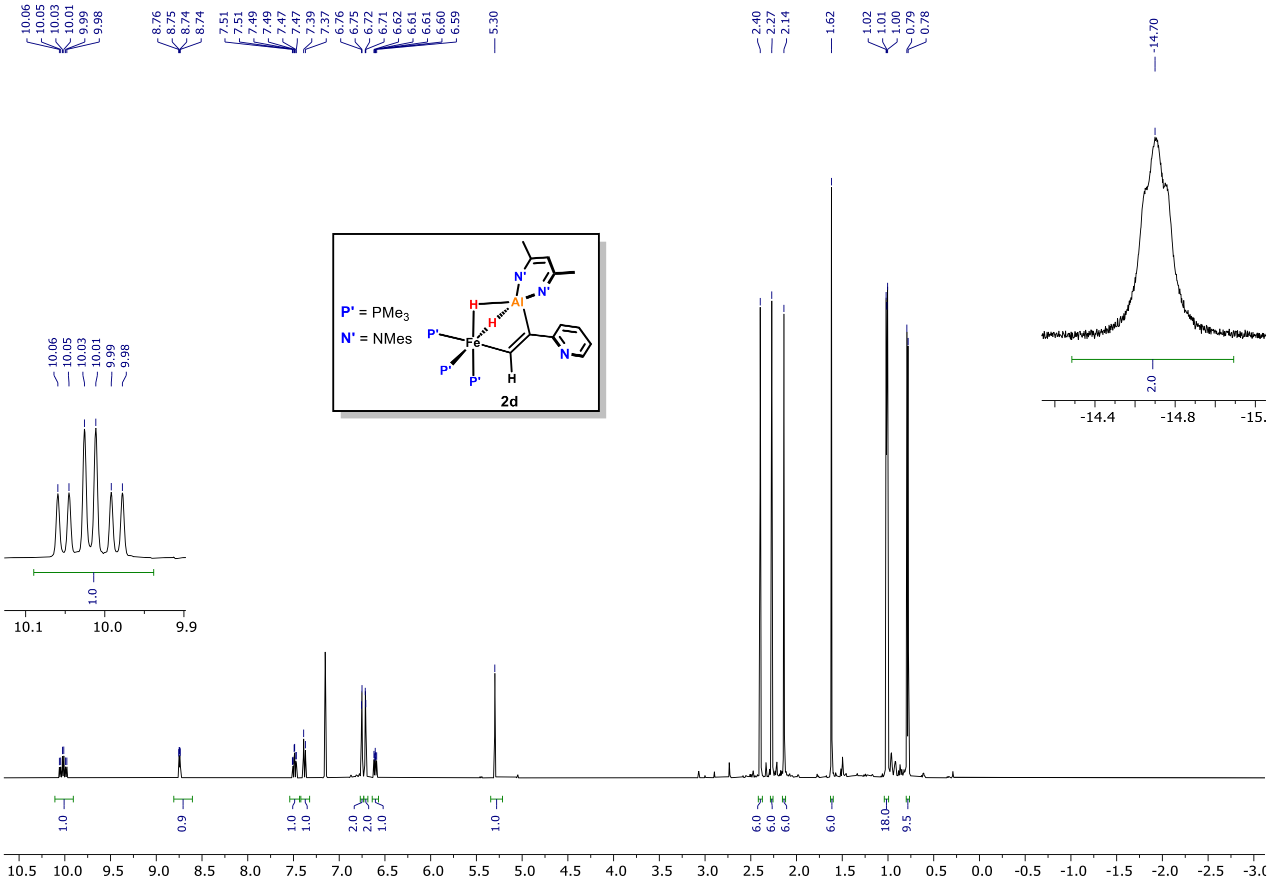

Figure S44. ^{1}H NMR of **2d** (400 MHz, C_6D_6, 298 K).

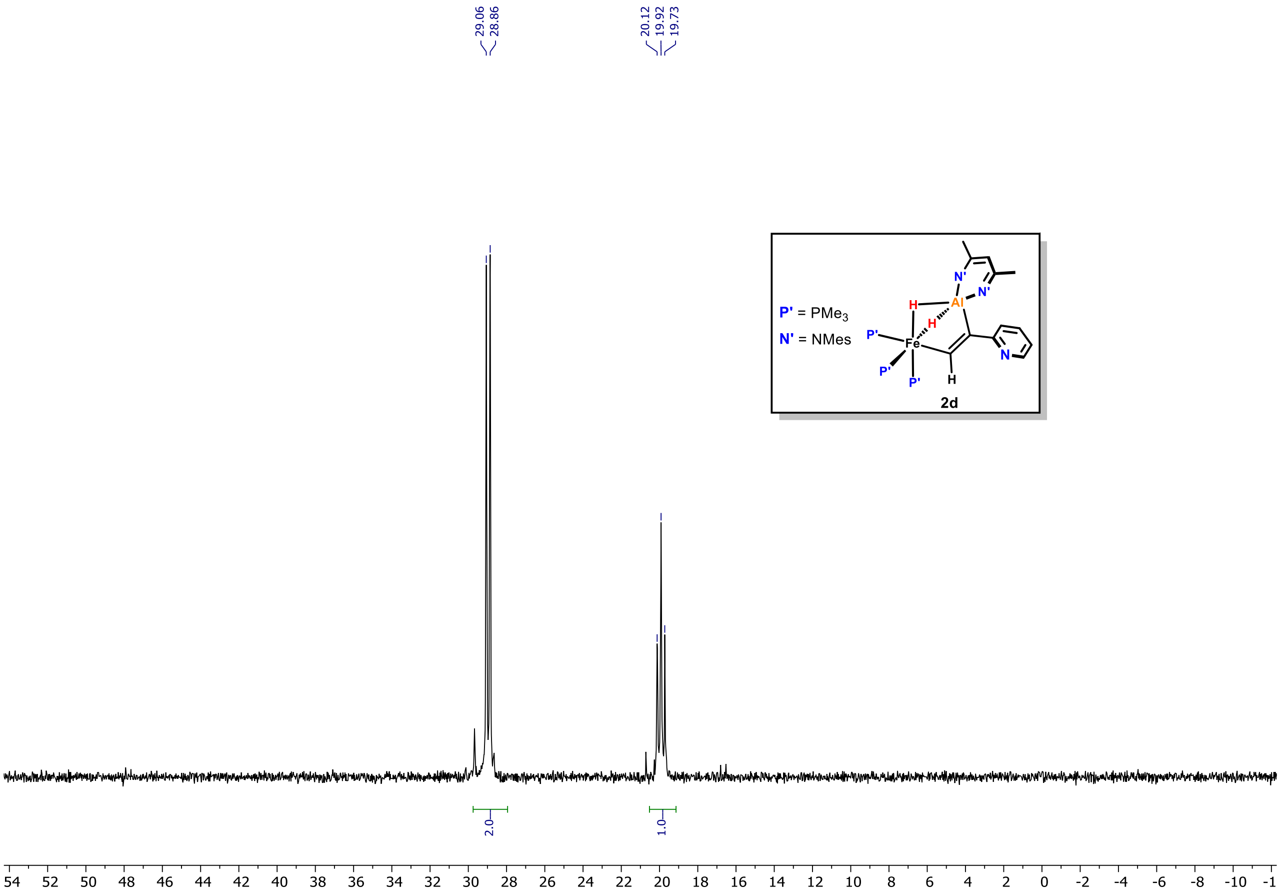

Figure S45. $^{31}P\{^{1}H\}$ NMR of **2d** (162 MHz, C_6D_6, 298 K).

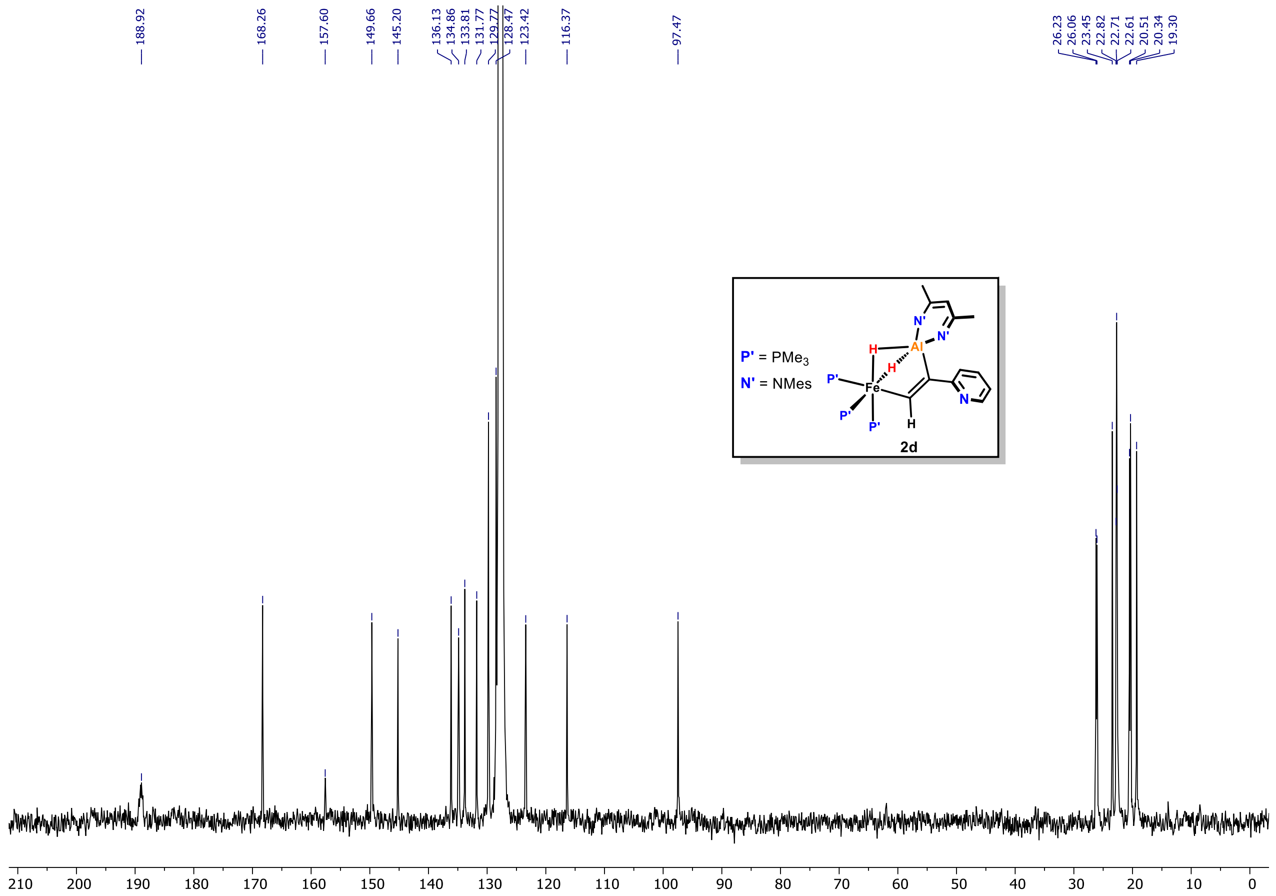

Figure S46. $^{13}C\{^{1}H\}$ NMR of **2d** (101 MHz, C_6D_6, 298 K).

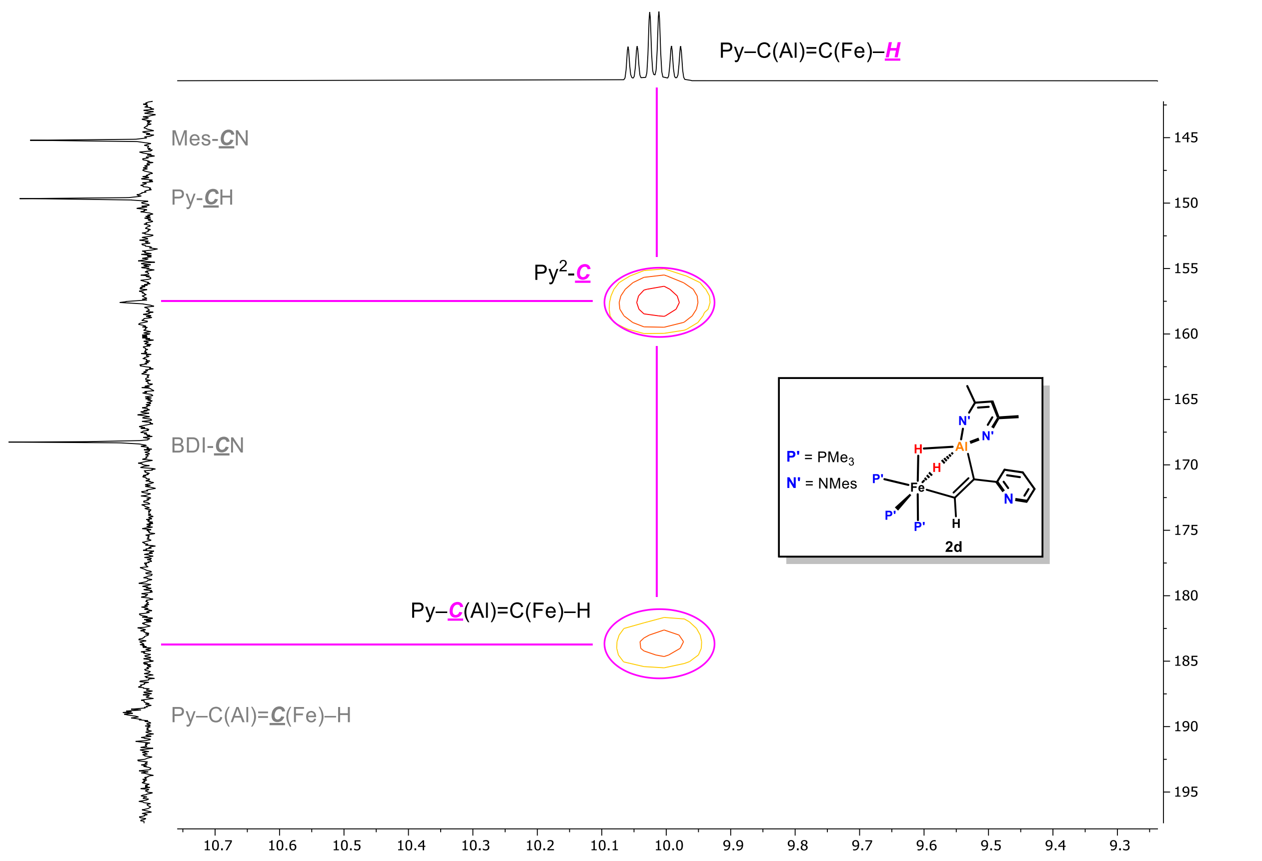

Figure S47. ^{1}H-^{13}C-HMBC NMR of **2d** (400 MHz, C_6D_6, 298 K).

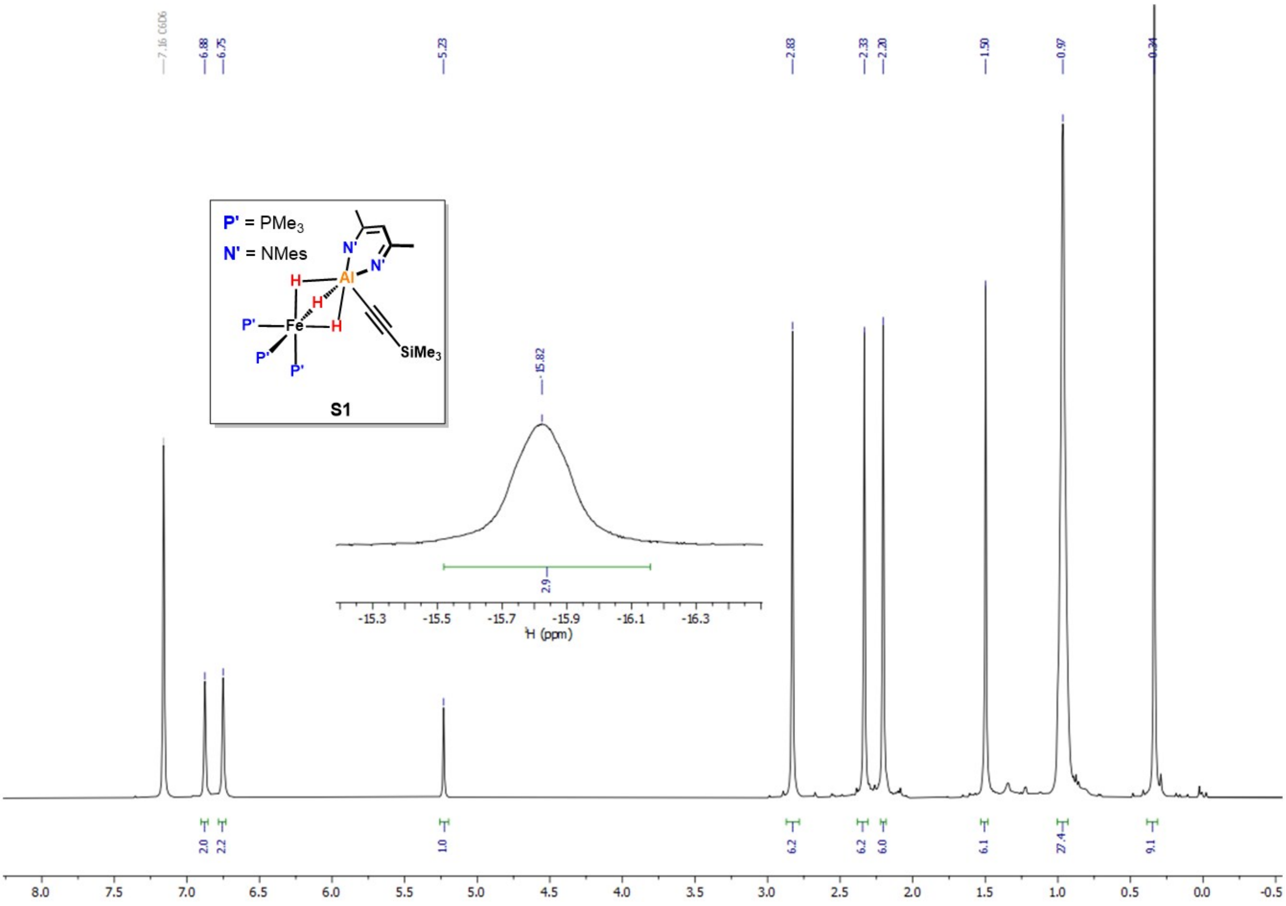

Figure S48. ^{1}H NMR of **S1** (400 MHz, C_6D_6, 298 K).

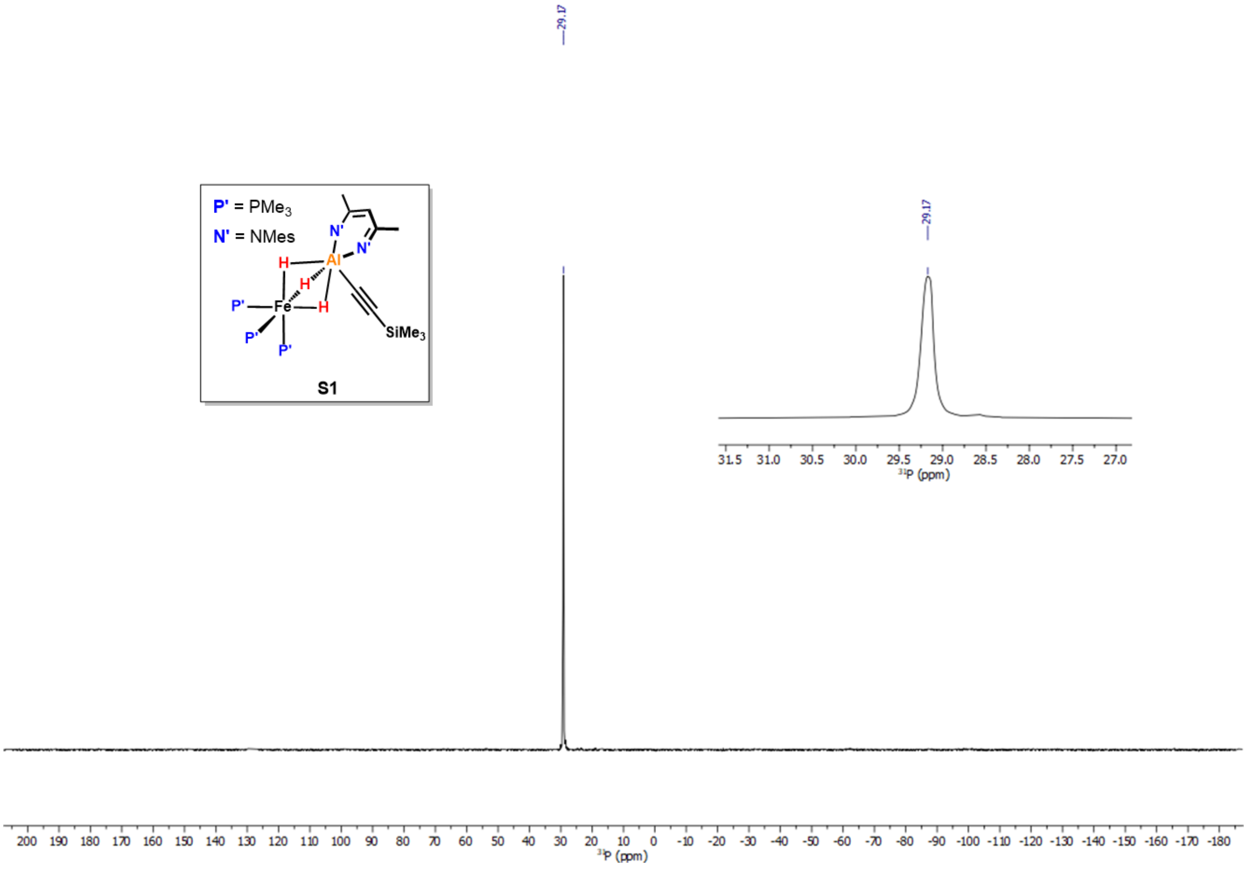

Figure S49. ^{31}P{^{1}H} NMR of **S1** (162 MHz, C_6D_6, 298 K).

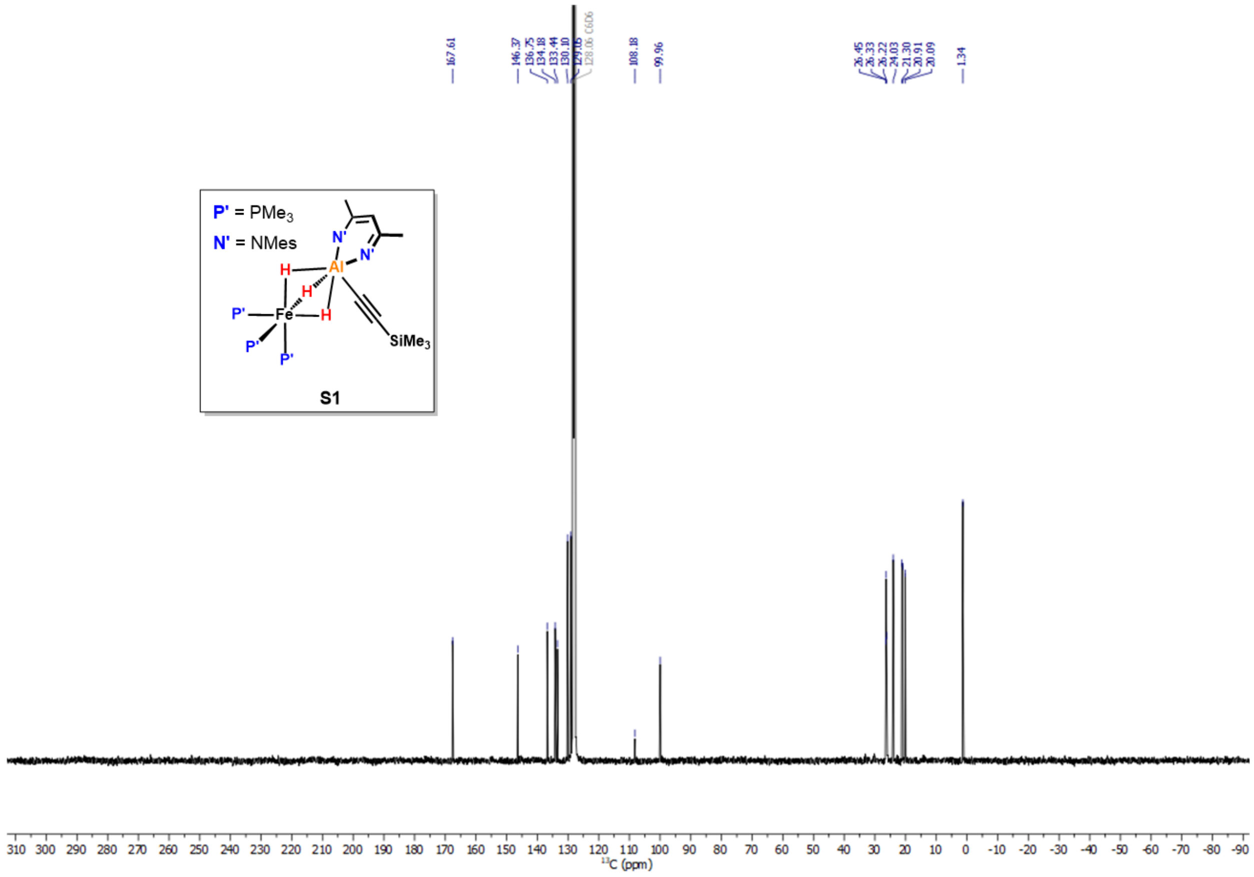

Figure S50. $^{13}C\{^{1}H\}$ NMR of **S1** (101 MHz, C_6D_6, 298 K).

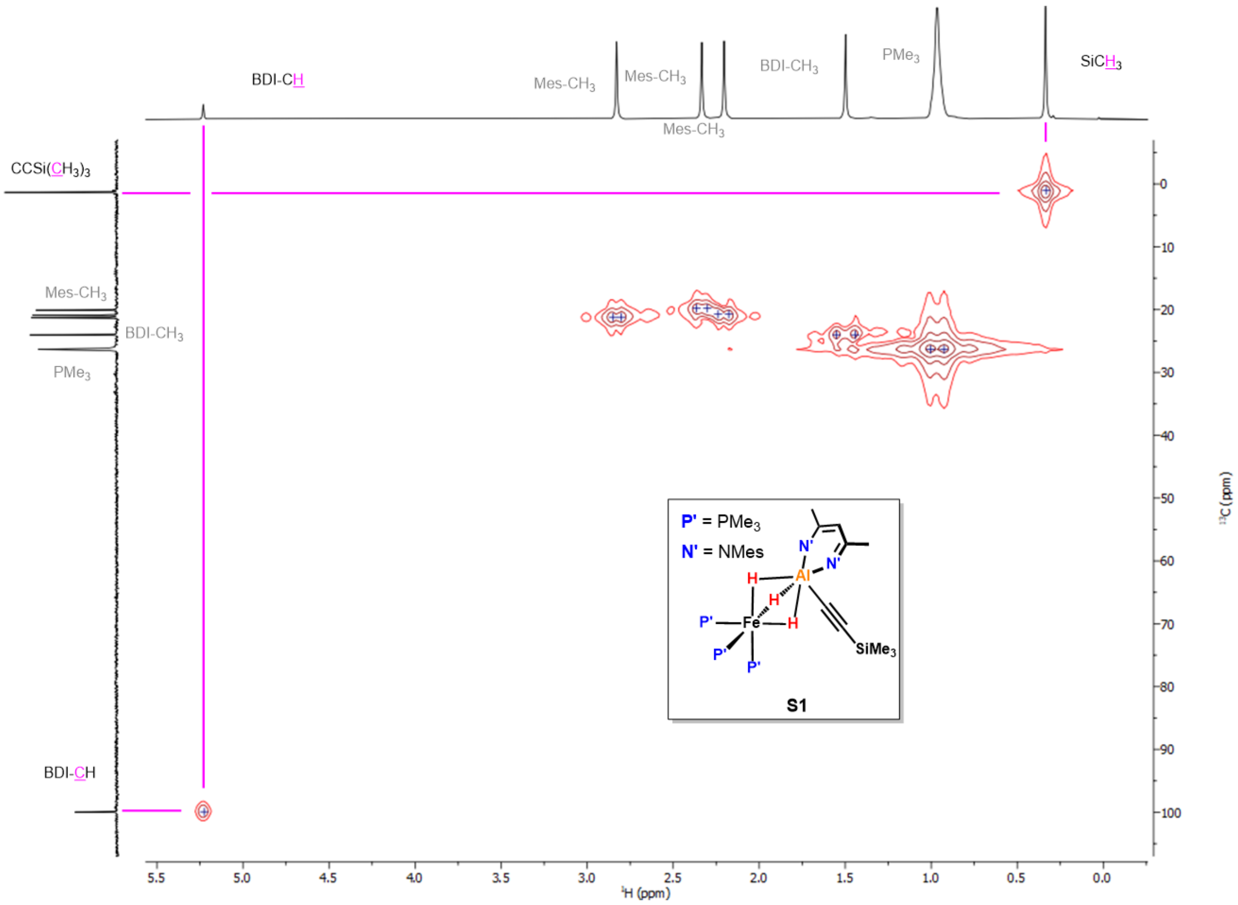

Figure S51. ^{1}H-^{13}C HSQC NMR of **S1** (400 MHz, C_6D_6, 298 K).

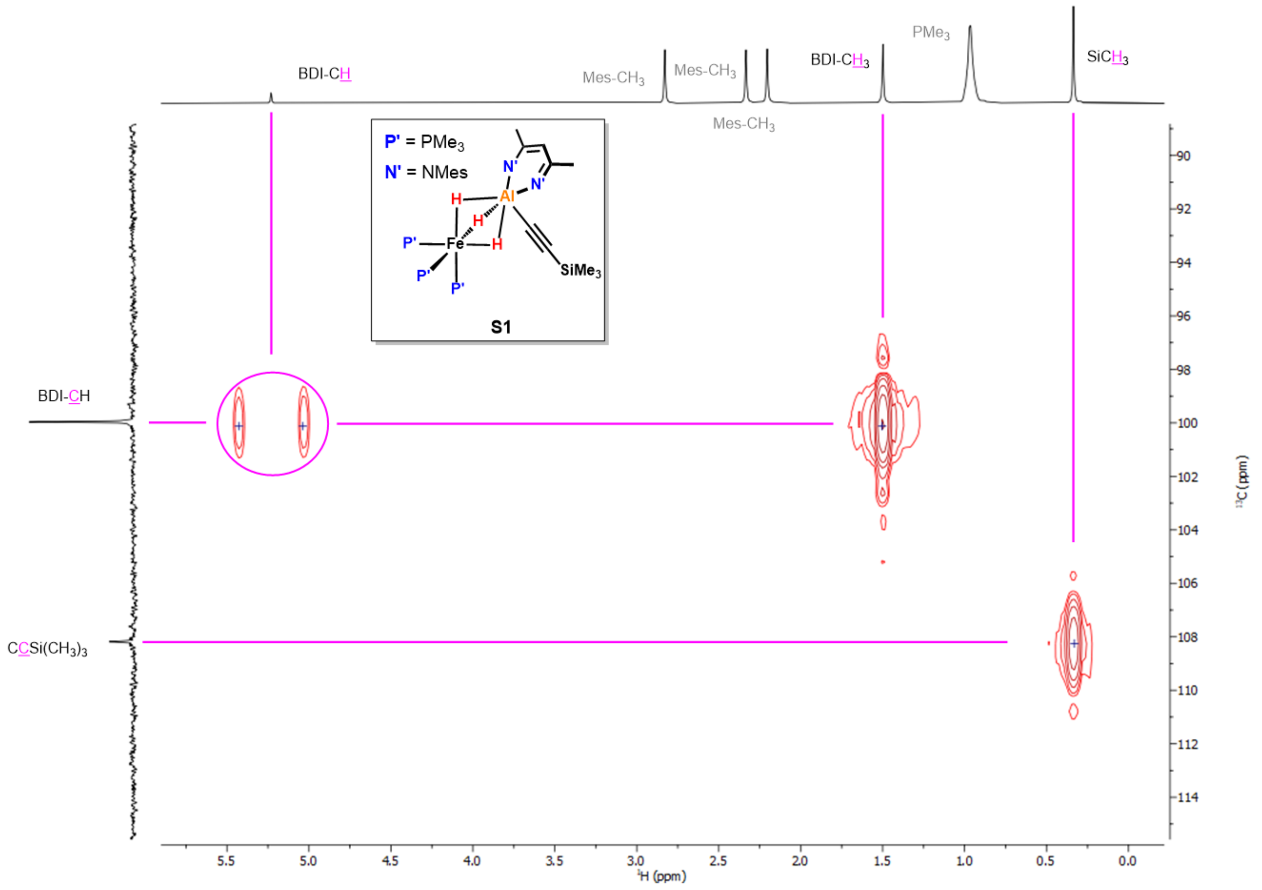

Figure S52. ^{1}H-^{13}C HMBC NMR of **S1** (400 MHz, C_6D_6, 298 K).

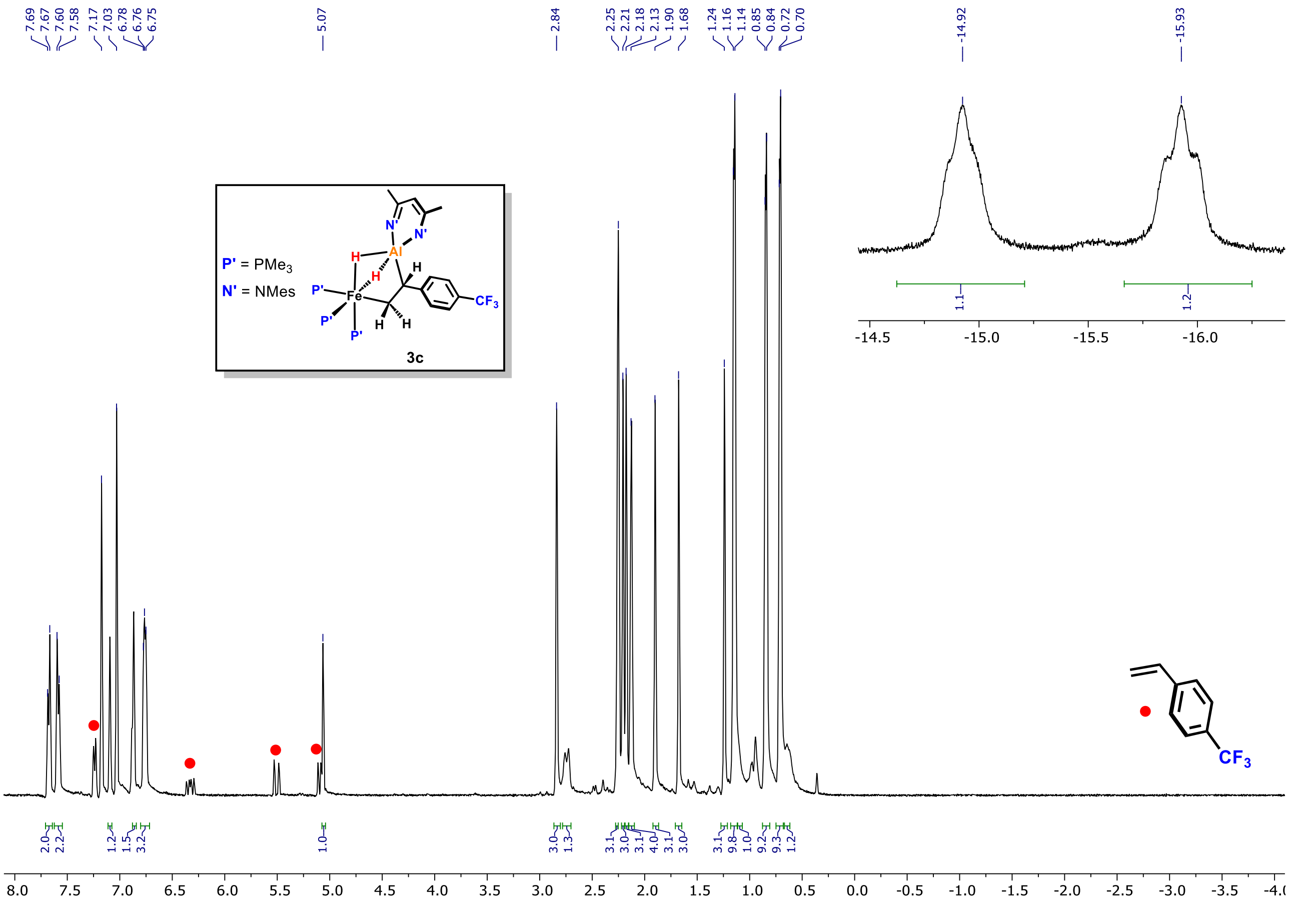

Figure S53. ^{1}H NMR of **3c** (400 MHz, toluene-d_8, 238 K).

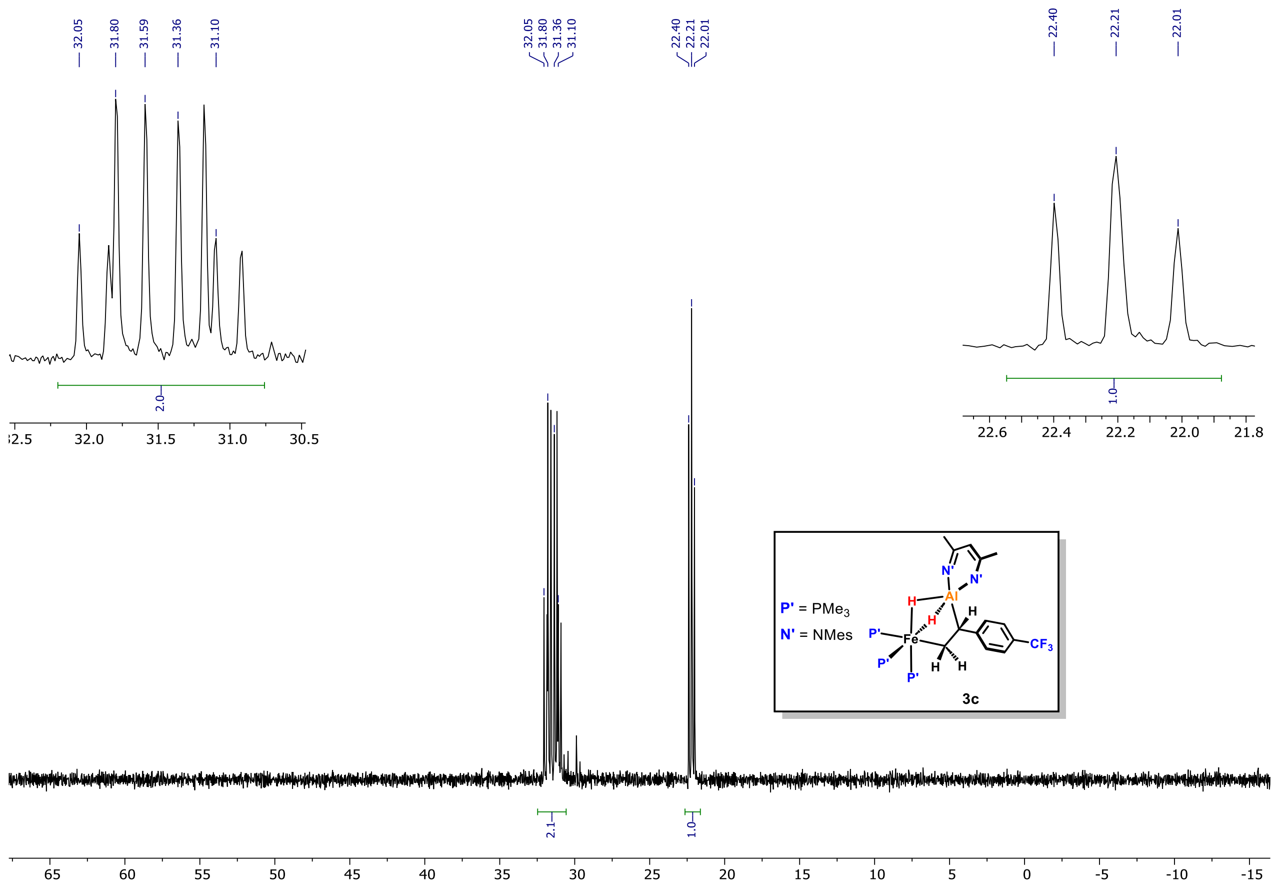

Figure S54. $^{31}P\{^{1}H\}$ NMR of **3c** (162 MHz, toluene-d_8, 238 K).

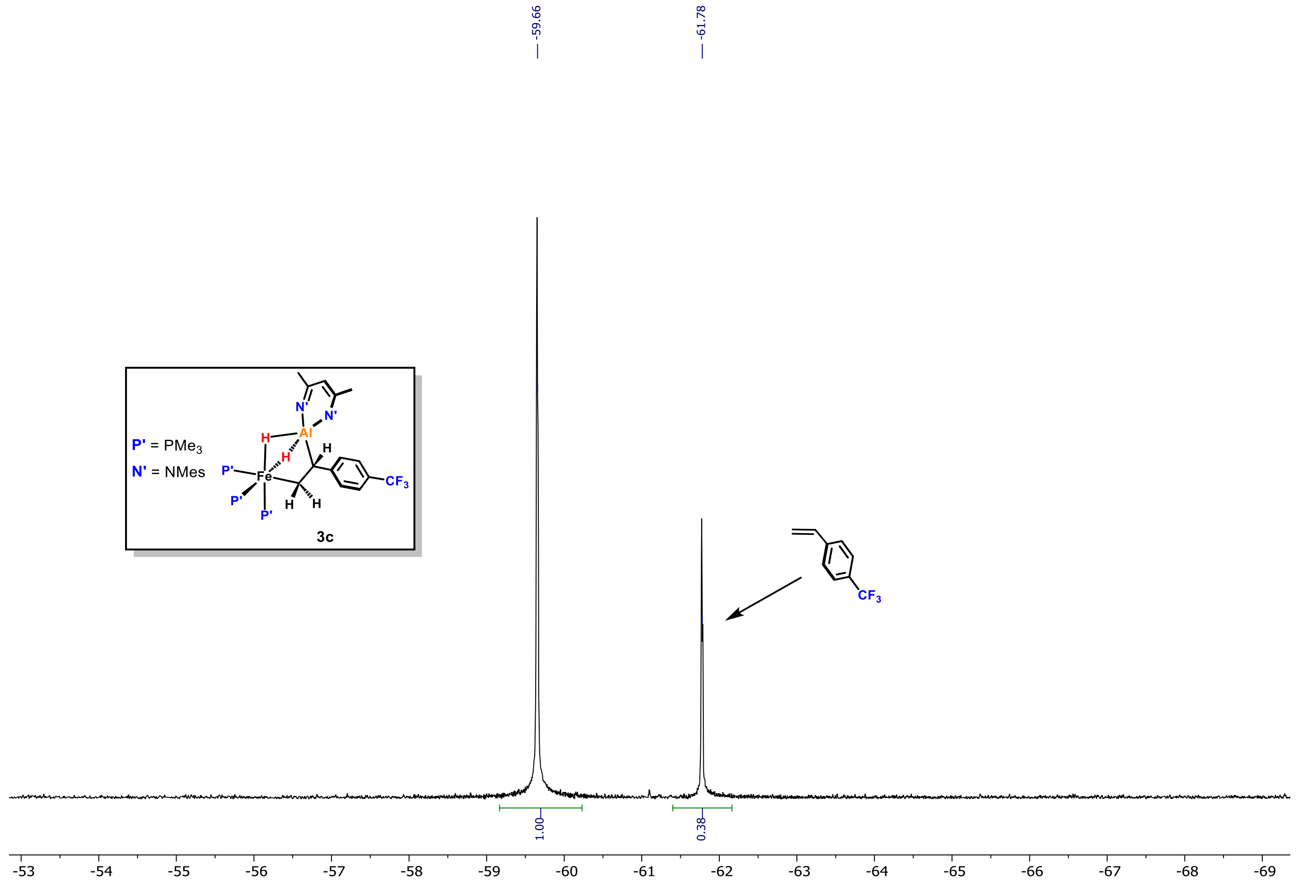

Figure S55. $^{19}F\{^{1}H\}$ NMR of **3c** (377 MHz, toluene-d_8, 238 K).

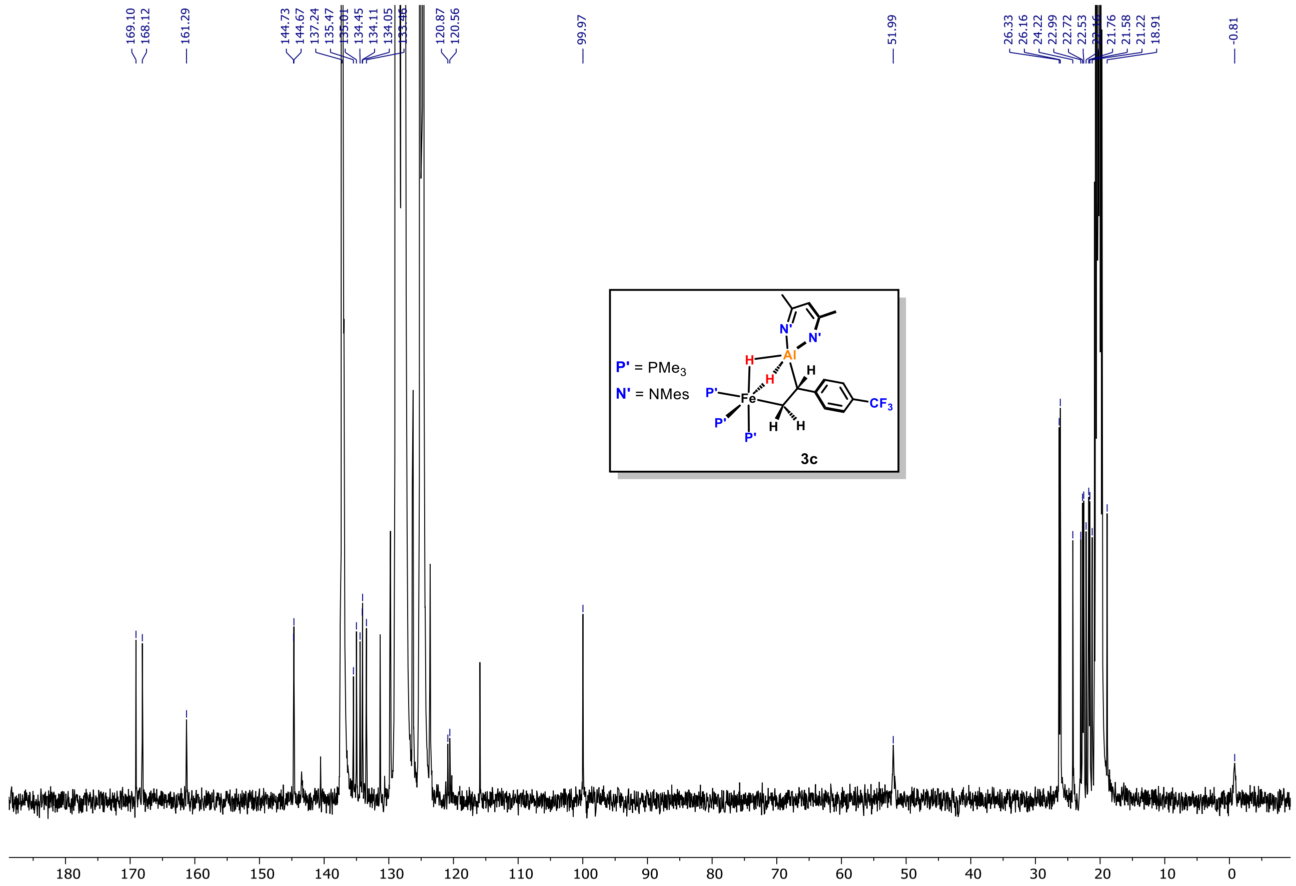

Figure S56. $^{13}C\{^{1}H\}$ NMR of **3c** (101 MHz, toluene-d_8, 238 K).

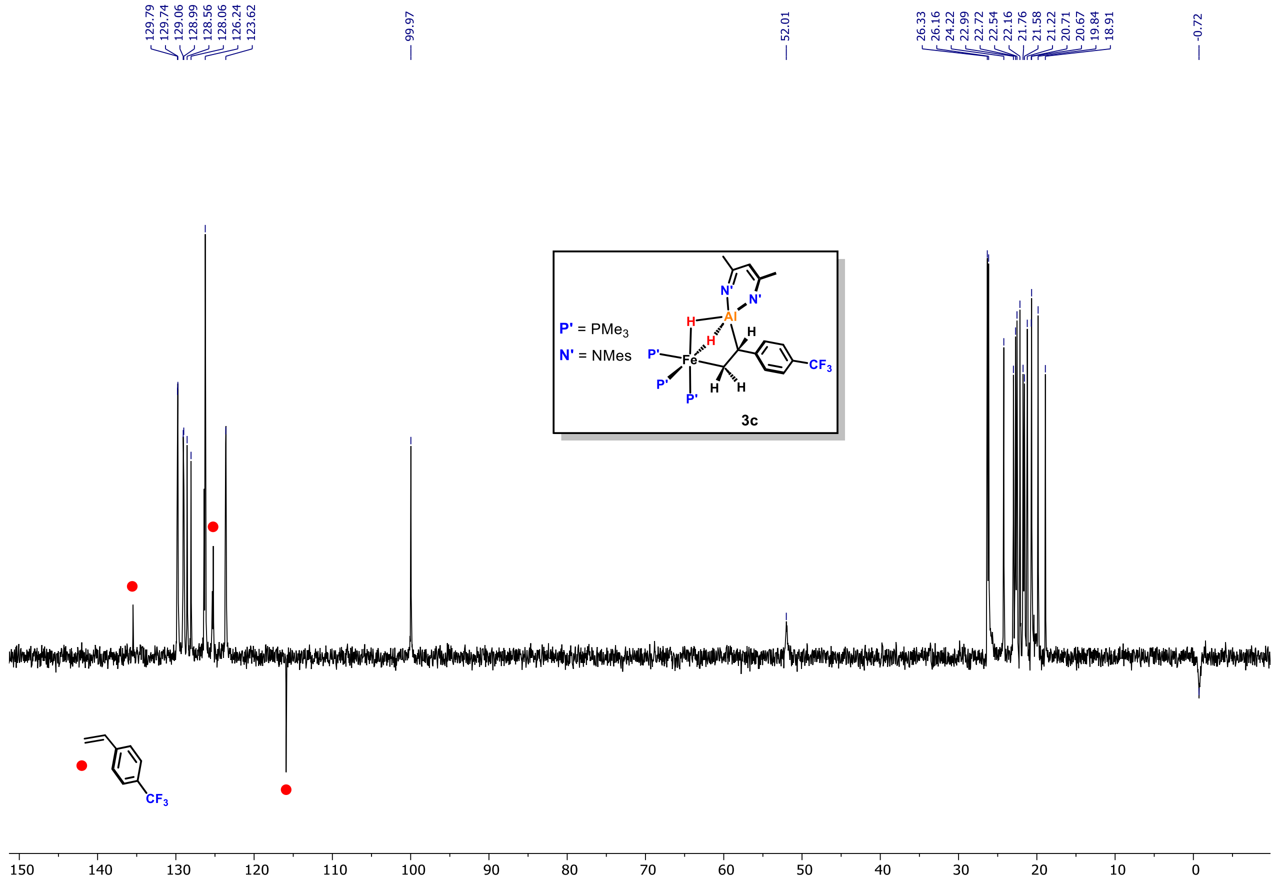

Figure S57. ^{1}H-^{13}C DEPT-135 NMR of **3c** (101 MHz, toluene-d_8, 238 K).

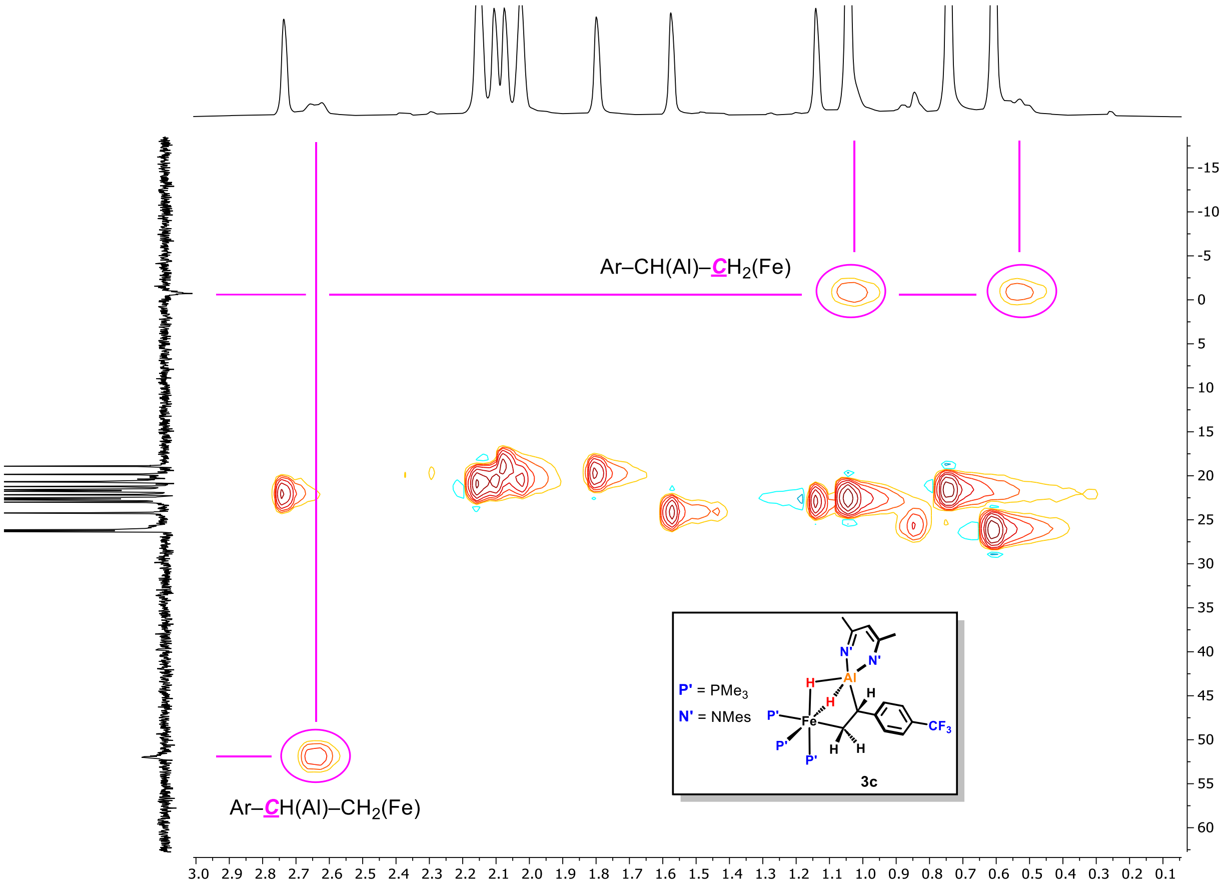

Figure S58. ^{1}H-^{13}C-HSQC NMR of **3c** (toluene-d_8, 238 K).

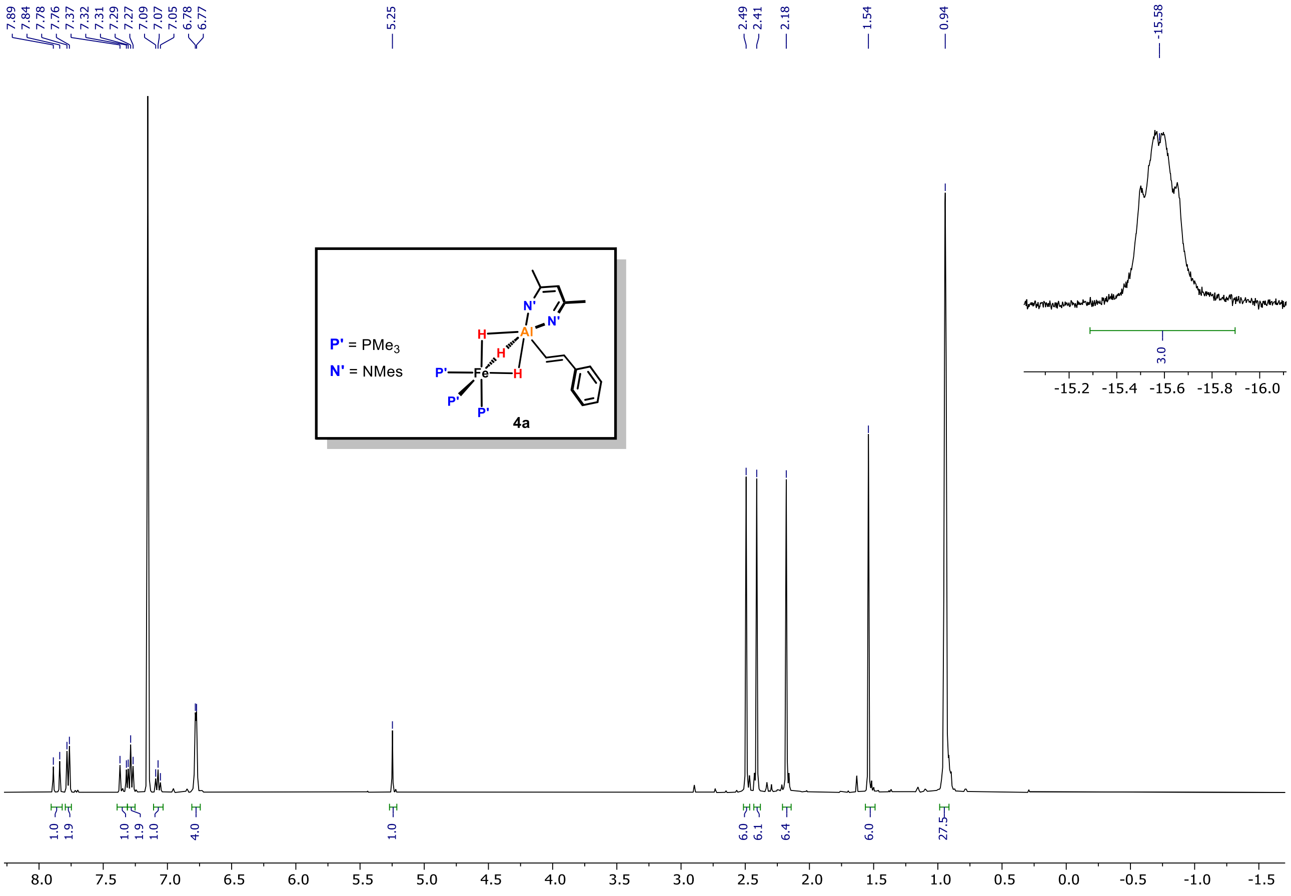

Figure S59. ^{1}H NMR of **4a** (400 MHz, C_6D_6, 298 K).

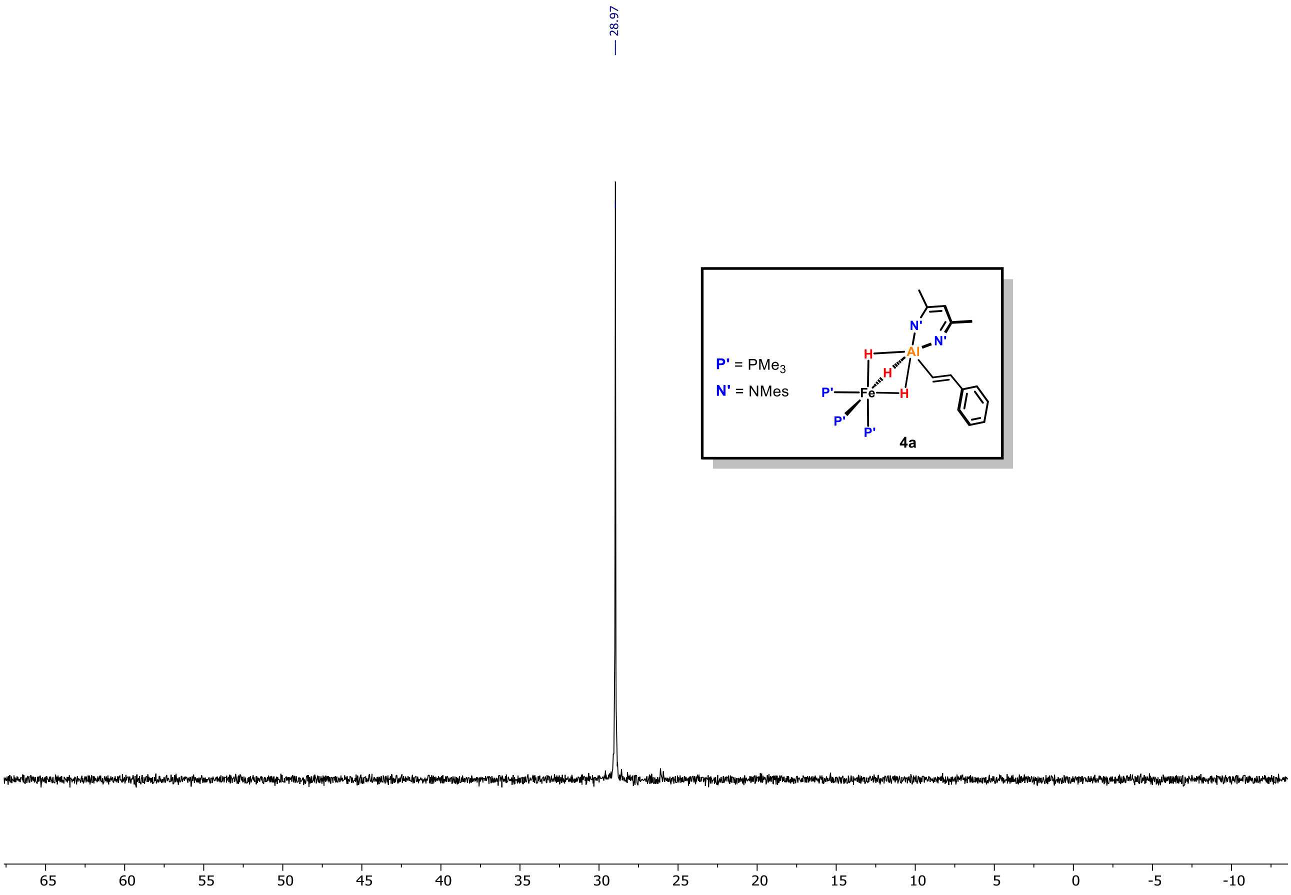

Figure S60. ^{31}P{^{1}H} NMR of **4a** (162 MHz, C_6D_6, 298 K).

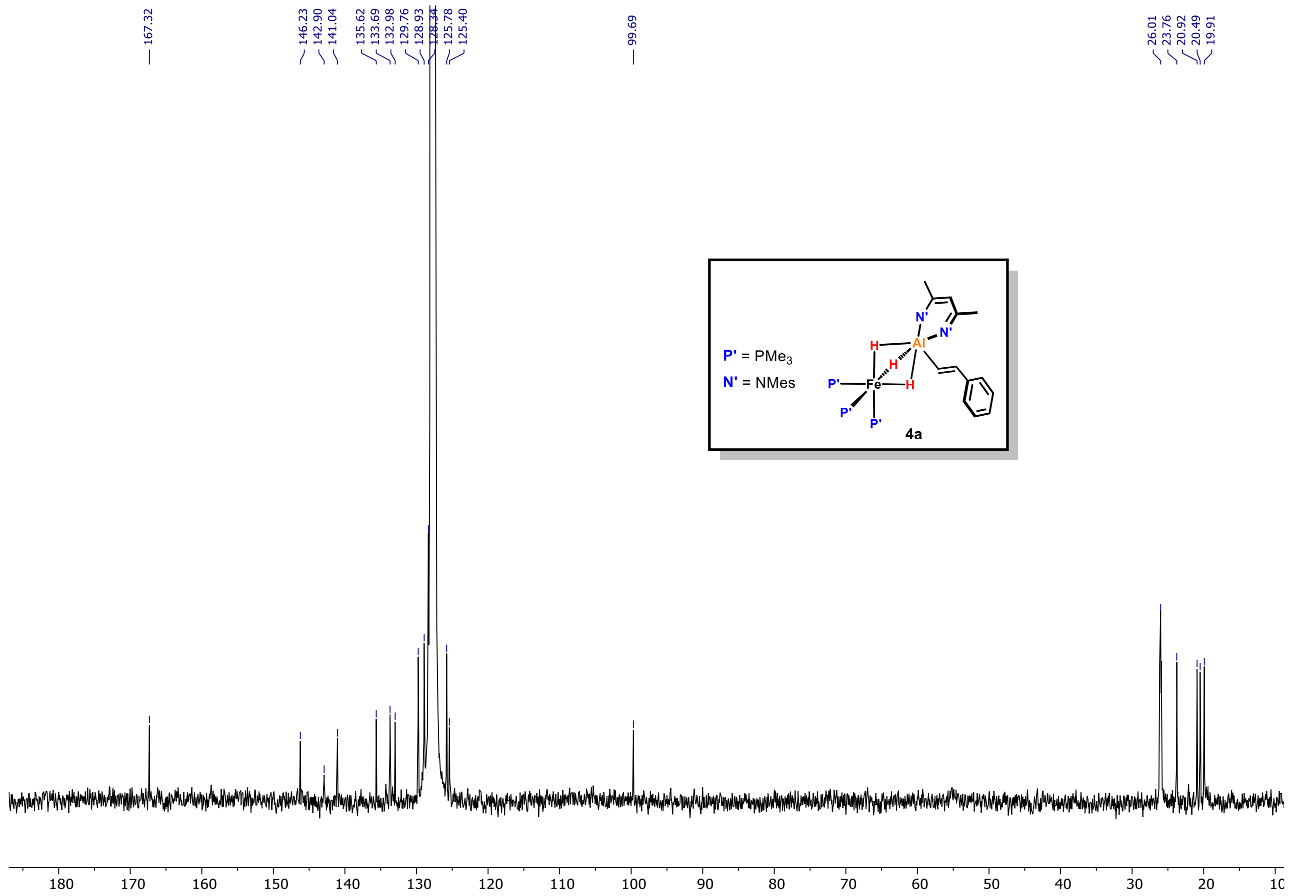

Figure S61. $^{13}C\{^{1}H\}$ NMR of **4a** (101 MHz, C_6D_6, 298 K).

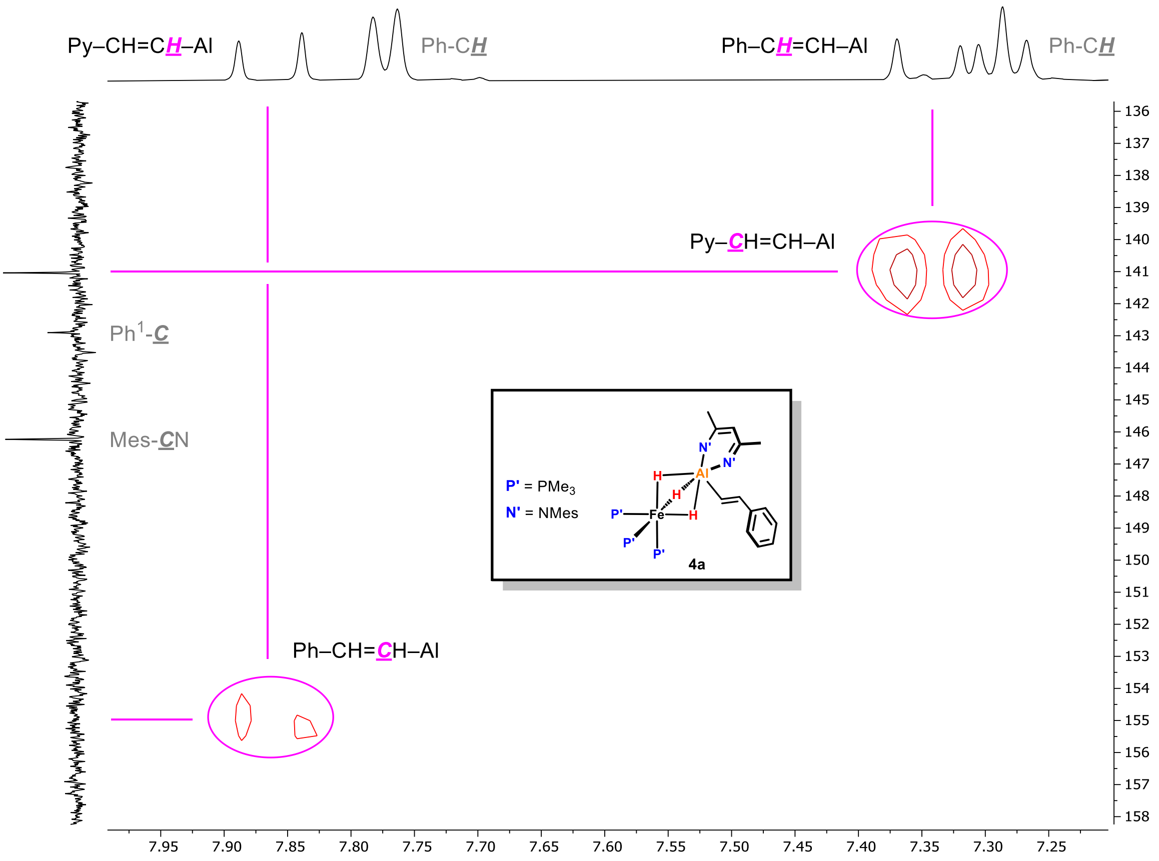

Figure S62. ^{1}H-^{13}C-HSQC NMR of **4a** (C_6D_6, 298 K).

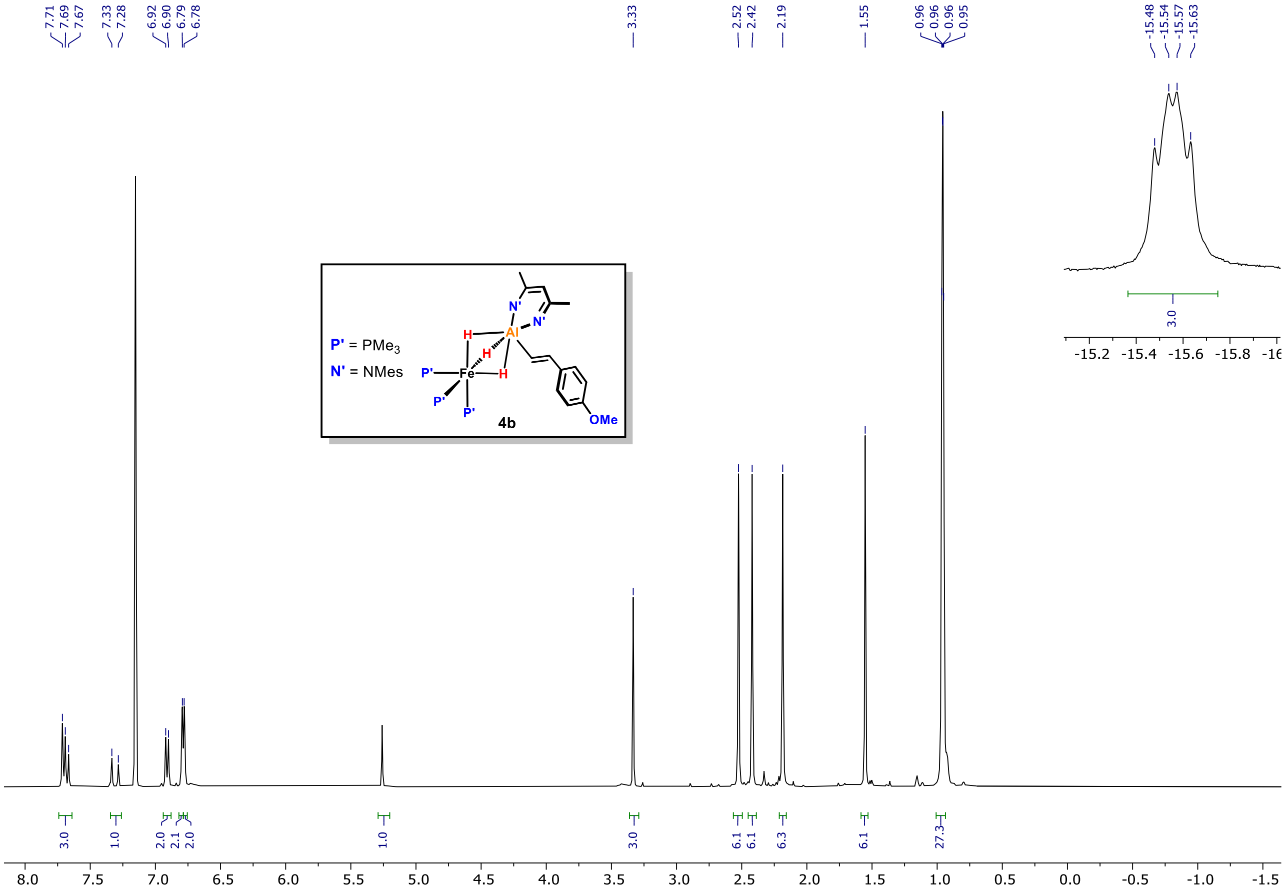

Figure S63. ^{1}H NMR of **4b** (400 MHz, C_6D_6, 298 K).

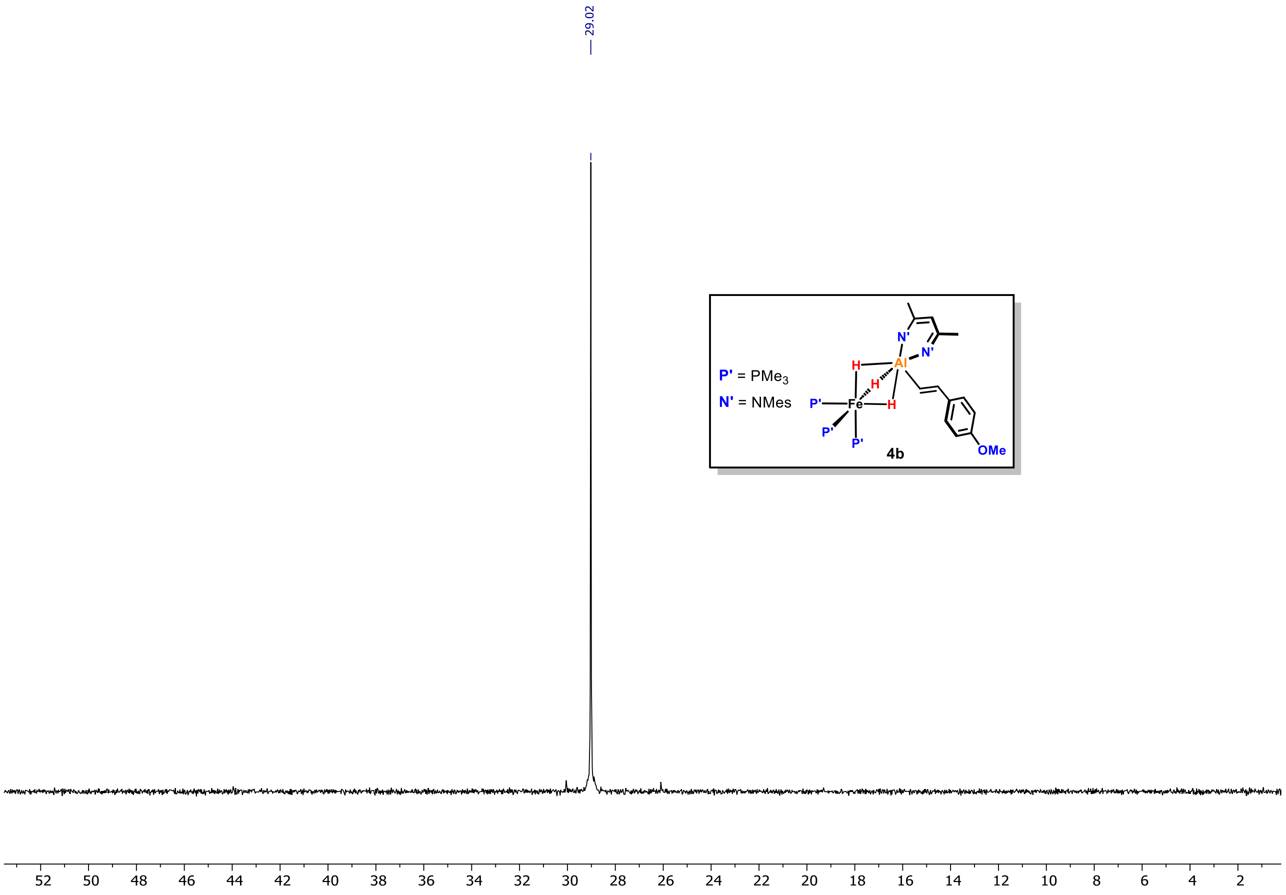

Figure S64. $^{31}P\{^{1}H\}$ NMR of **4b** (162 MHz, C_6D_6, 298 K).

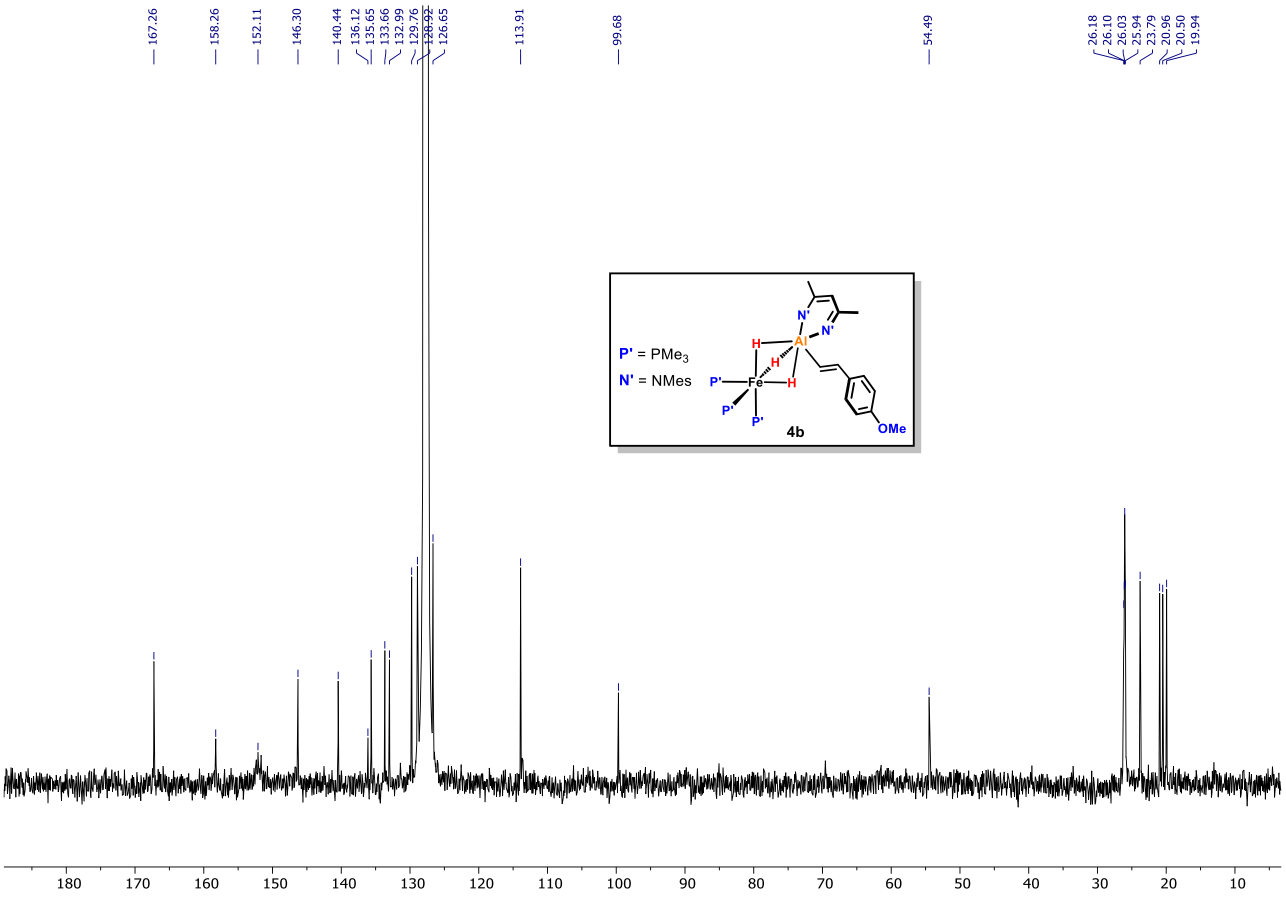

Figure S65. $^{13}C\{^{1}H\}$ NMR of **4b** (101 MHz, C_6D_6, 298 K).

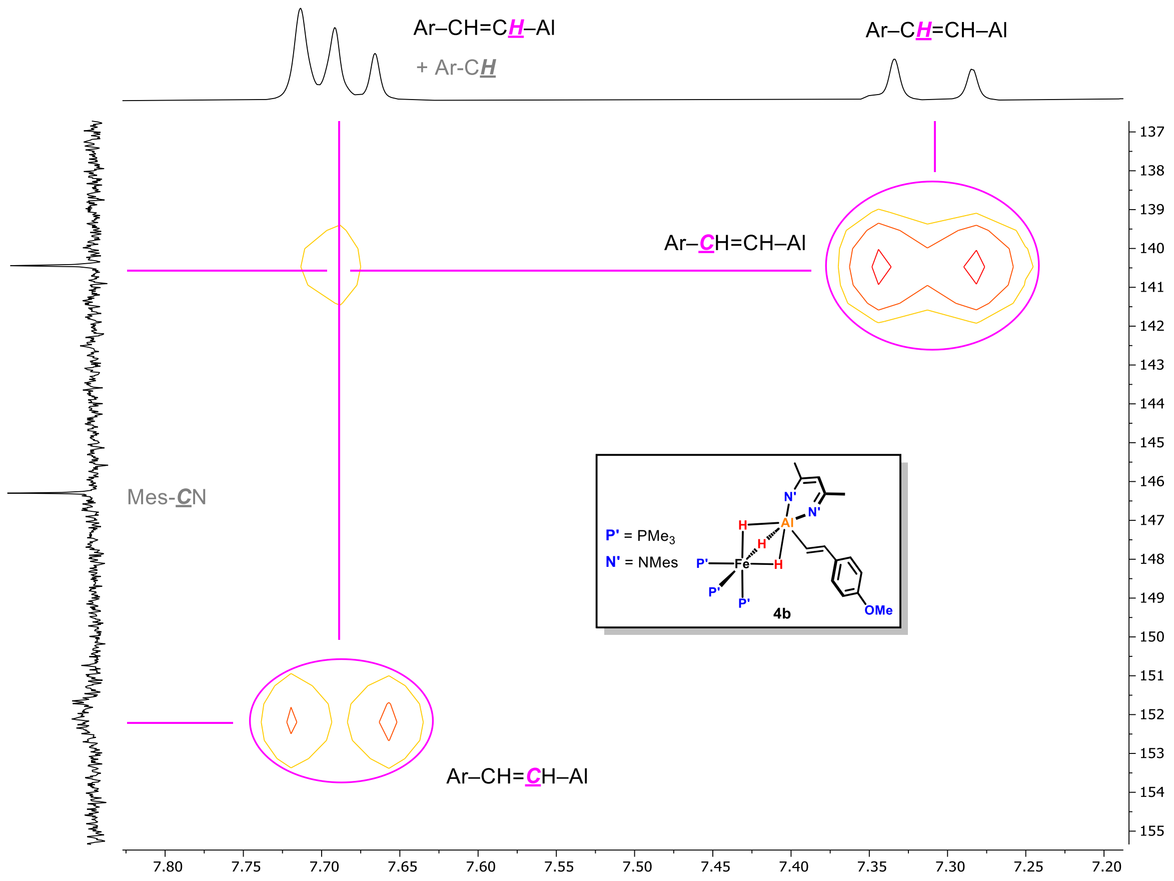

Figure S66. ^{1}H-^{13}C-HSQC NMR of **4b** (400 MHz, C_6D_6, 298 K).

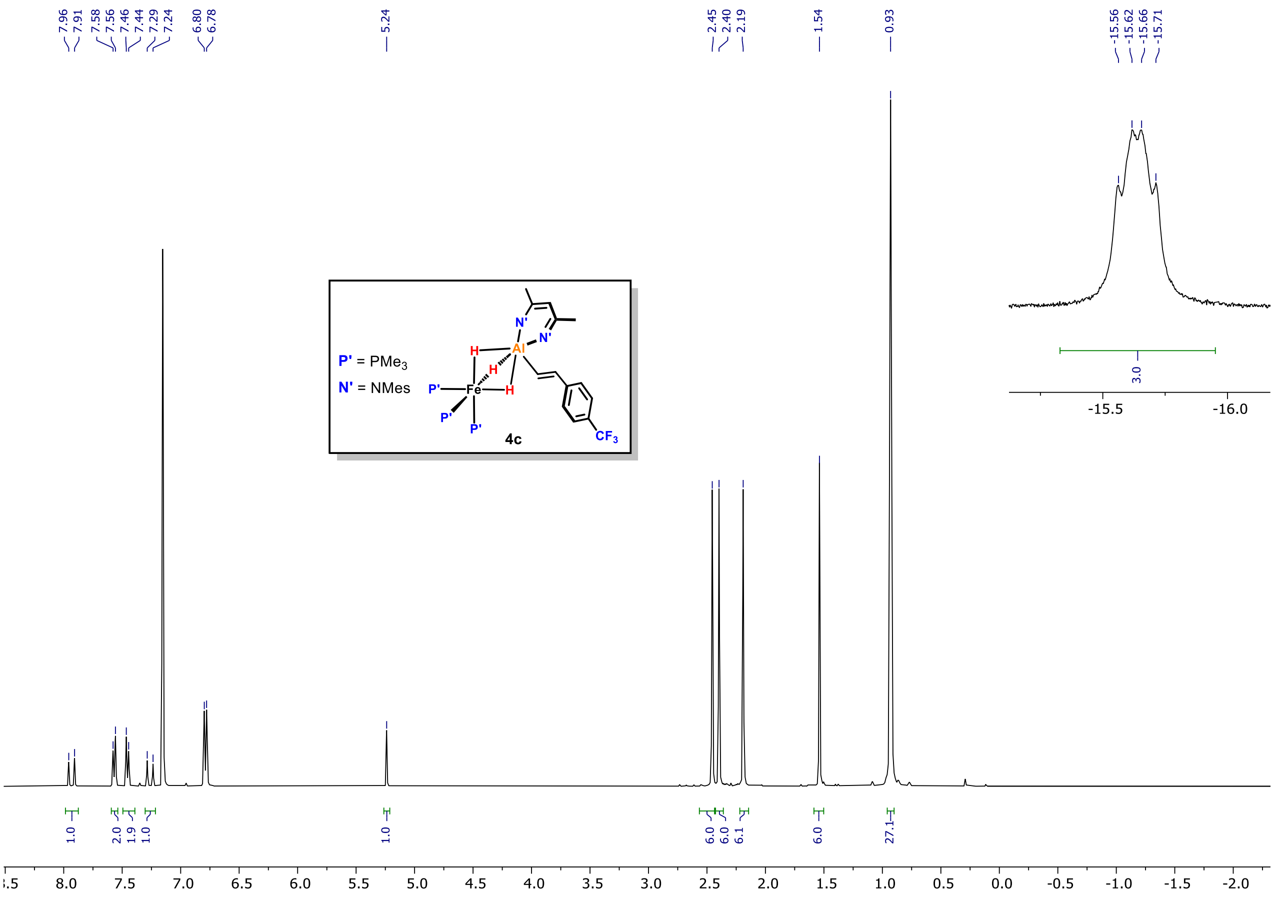

Figure S67. ^{1}H NMR of **4c** (400 MHz, toluene-d_8, 298 K).

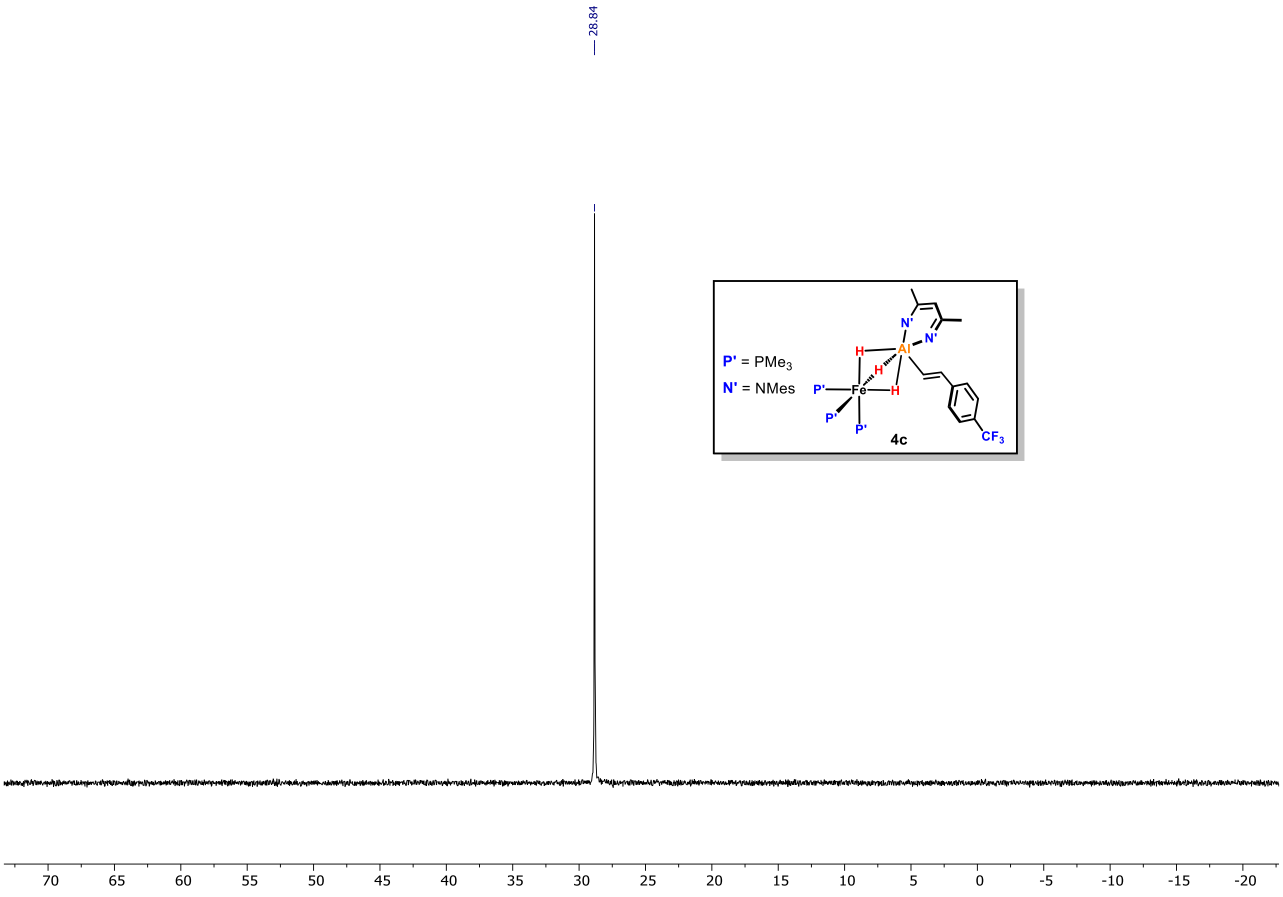

Figure S68. $^{31}P\{^{1}H\}$ NMR of **4c** (162 MHz, toluene-d_8, 298 K).

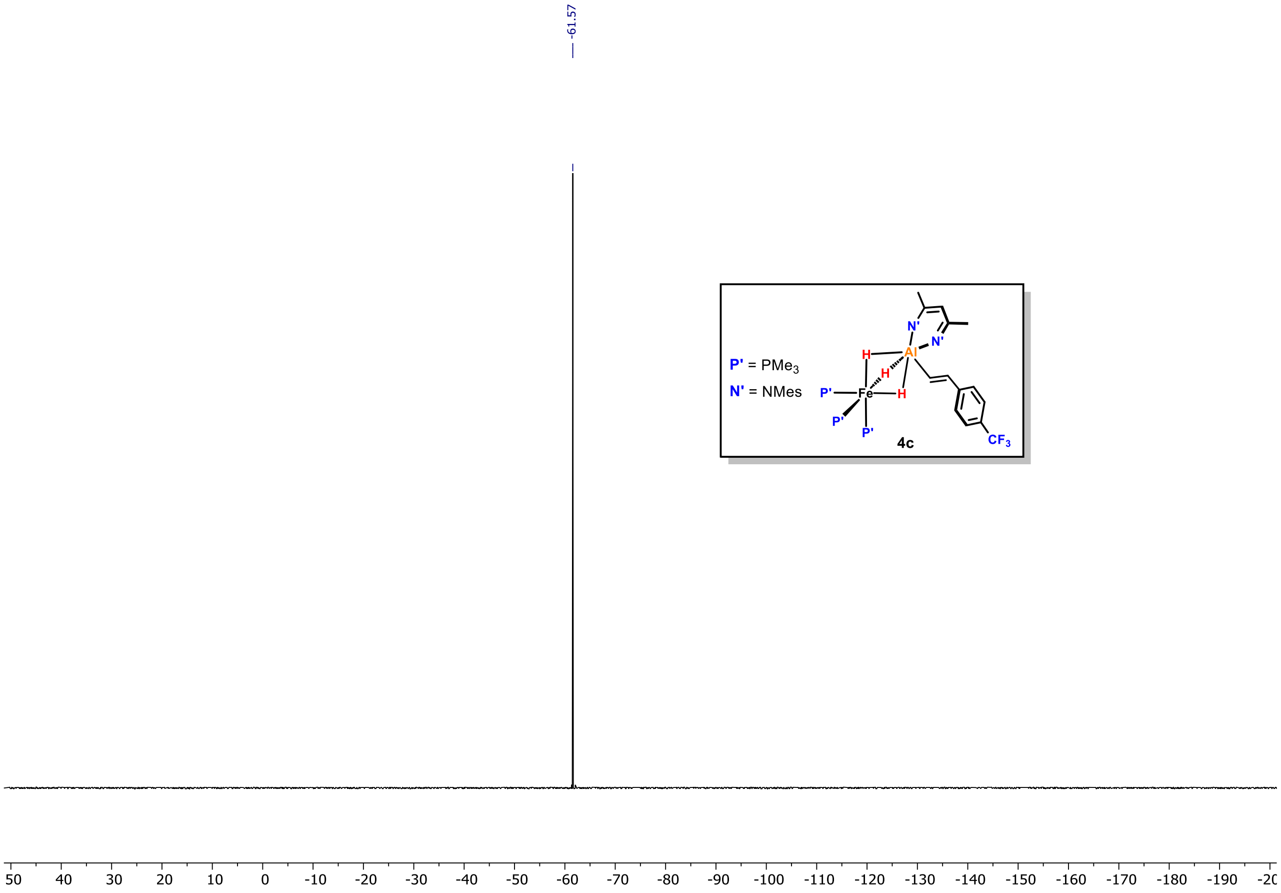

Figure S69. $^{19}F\{^{1}H\}$ NMR of **4c** (377 MHz, toluene-d_8, 298 K).

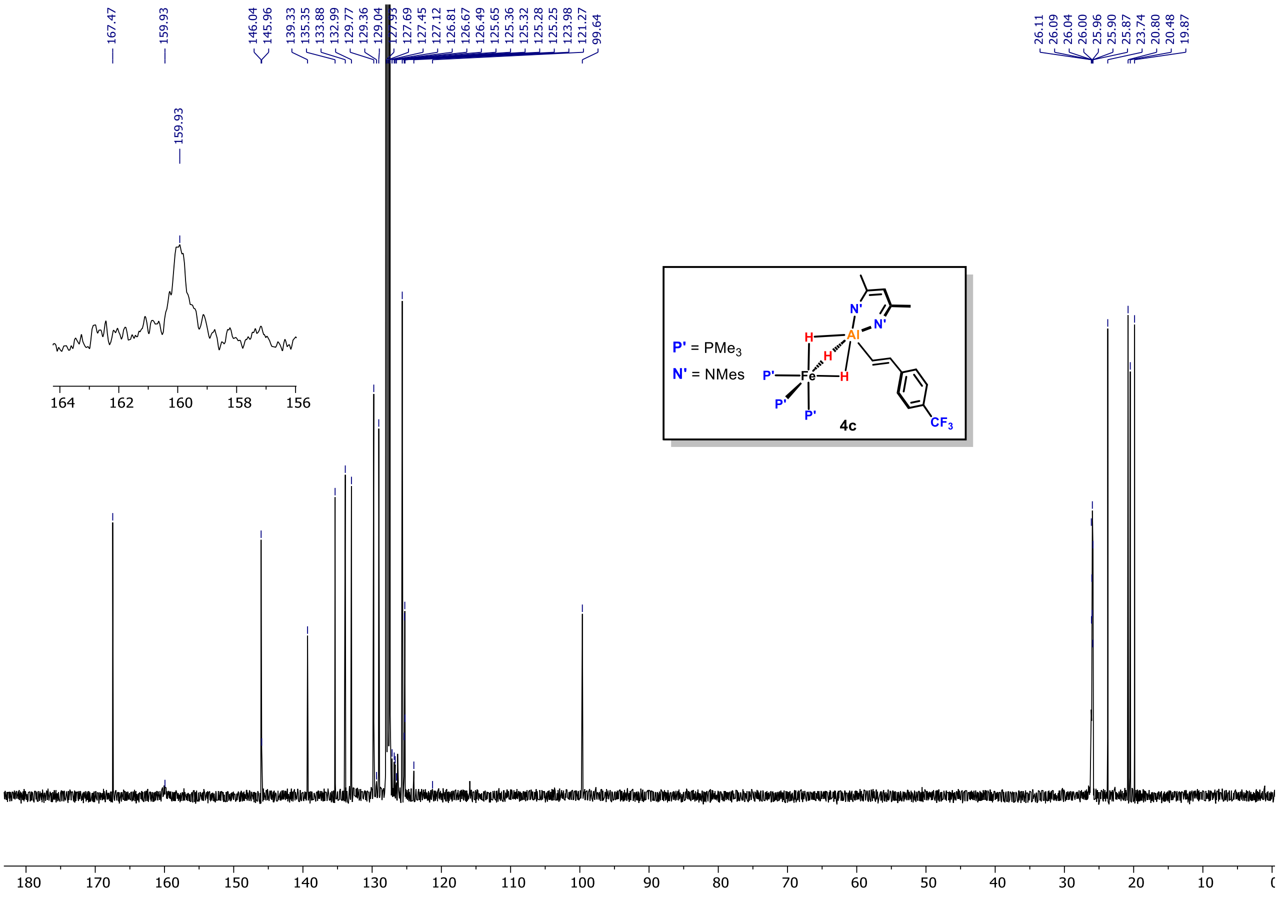

Figure S70. $^{13}C\{^{1}H\}$ NMR of **4c** (101 MHz, toluene-d_8, 298 K).

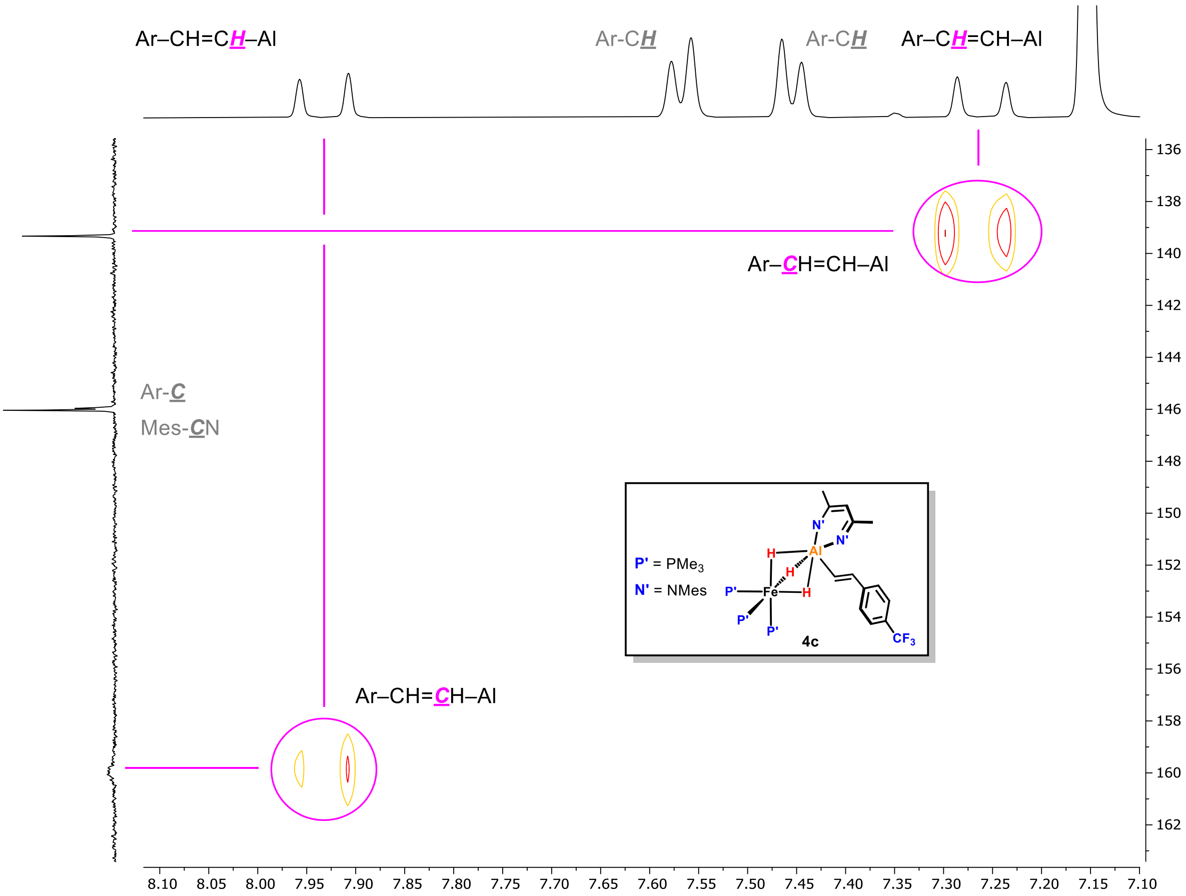

Figure S71. ^{1}H-^{13}C HSQC NMR of **4c** (C_6D_6, 298 K).

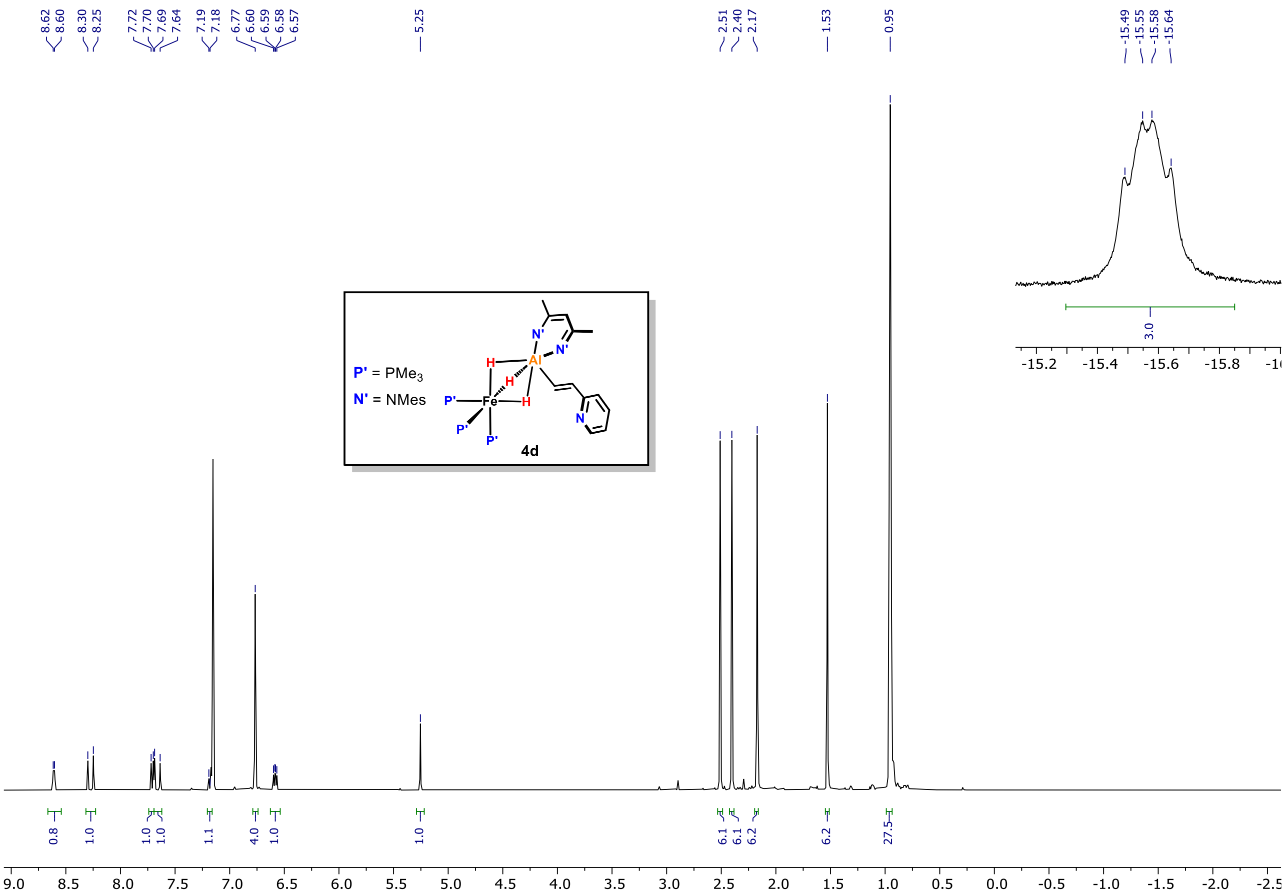

Figure S72. ^{1}H NMR of **4d** (400 MHz, C_6D_6, 298 K).

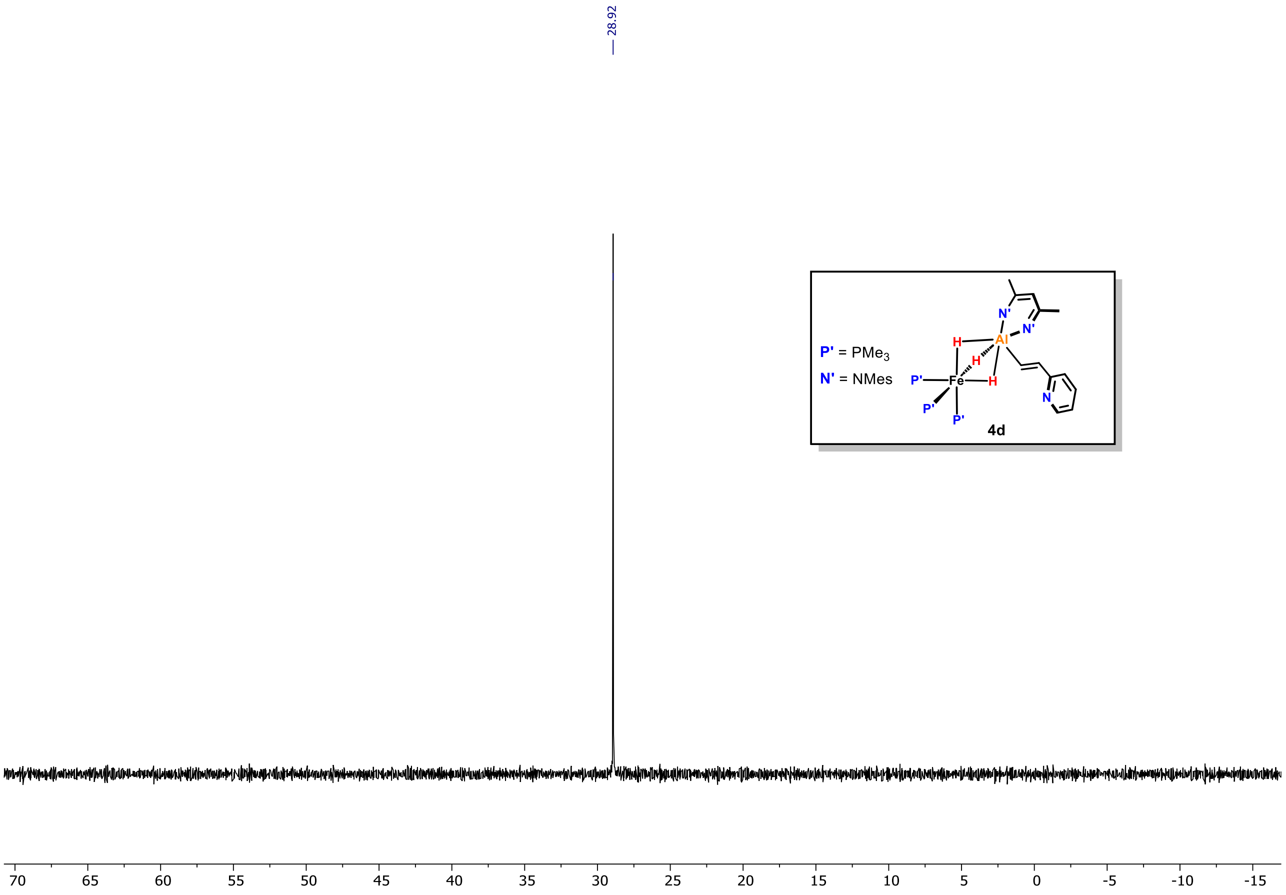

Figure S73. $^{31}P\{^{1}H\}$ NMR of **4d** (162 MHz, C_6D_6, 298 K).

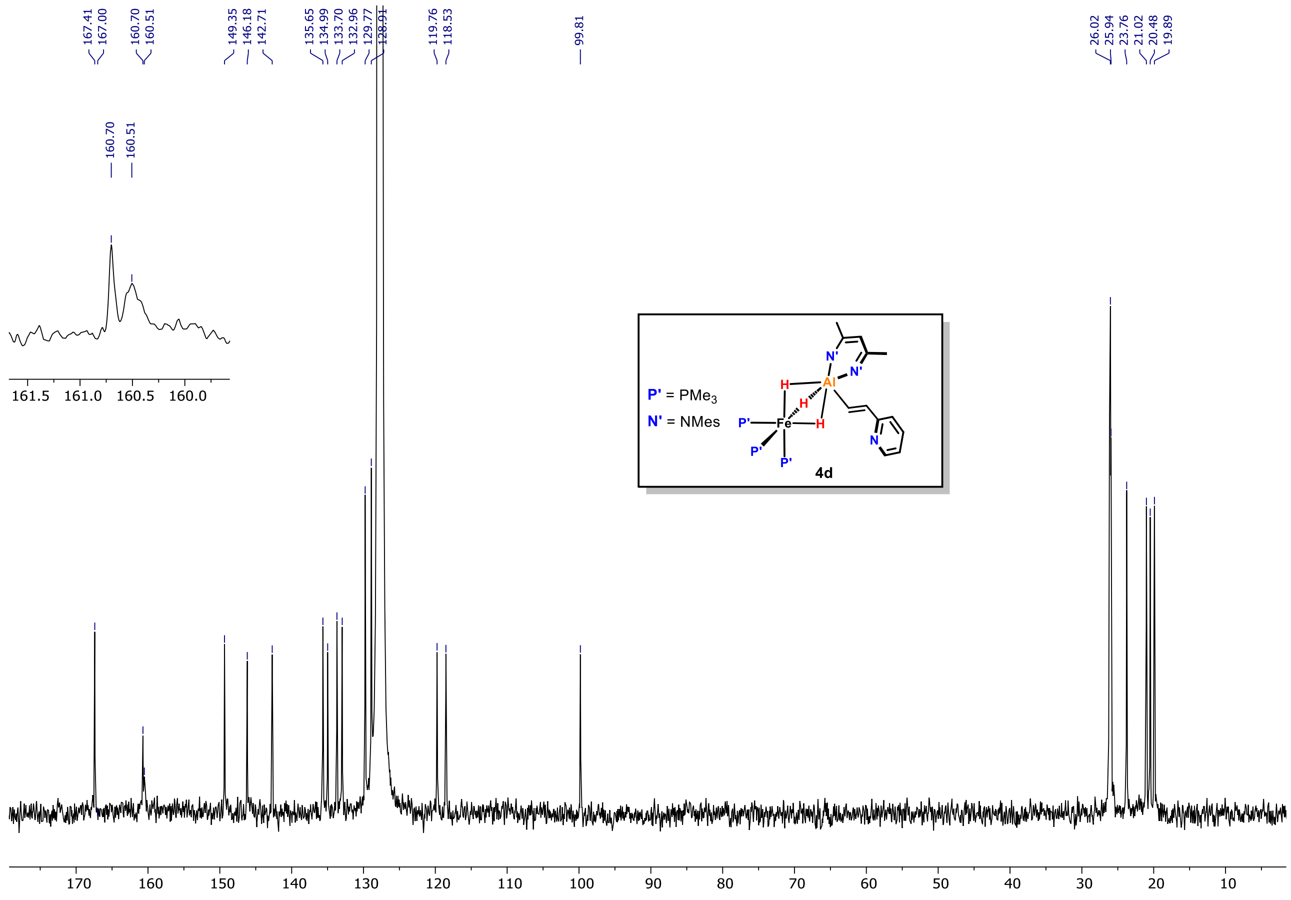

Figure S74. $^{13}C\{^{1}H\}$ NMR of **4d** (101 MHz, C_6D_6, 298 K).

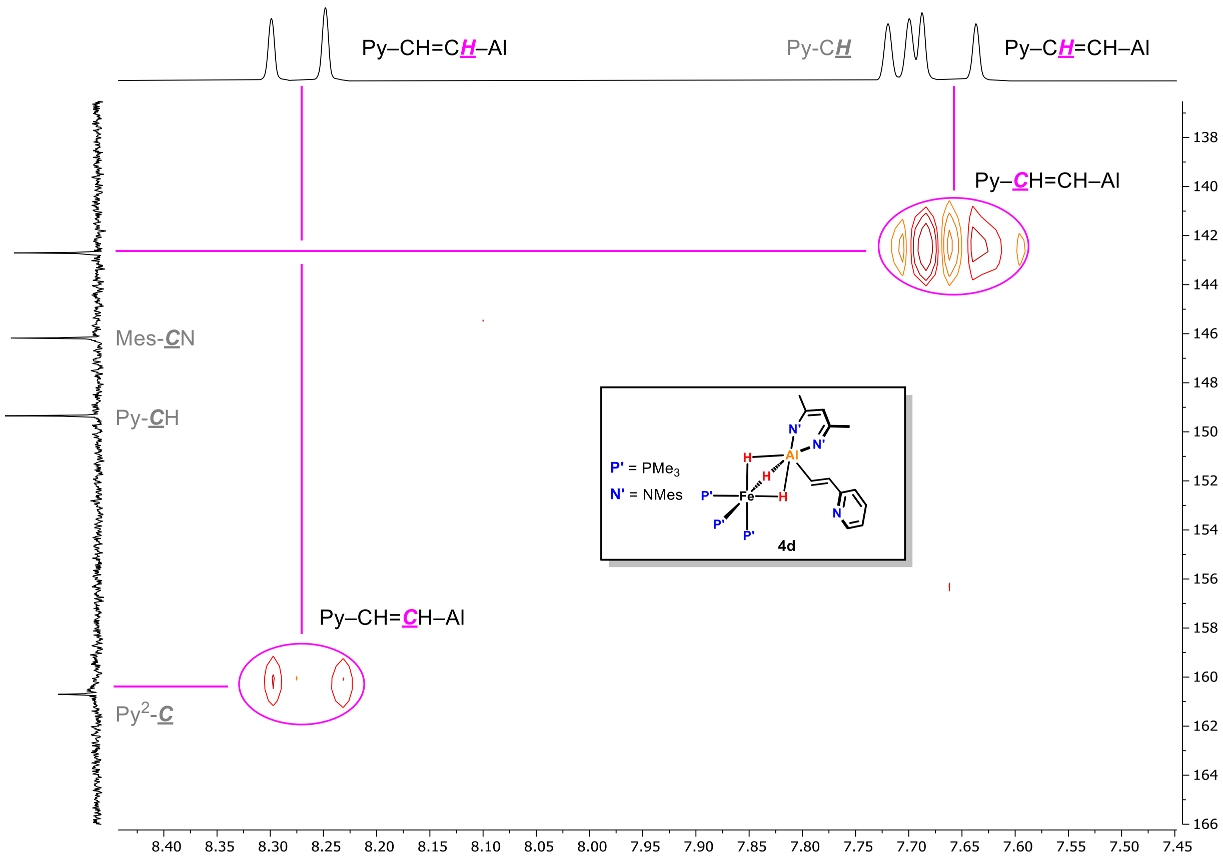

Figure S75. ^{1}H-^{13}C-HSQC NMR of **4d** (C_6D_6, 298 K).

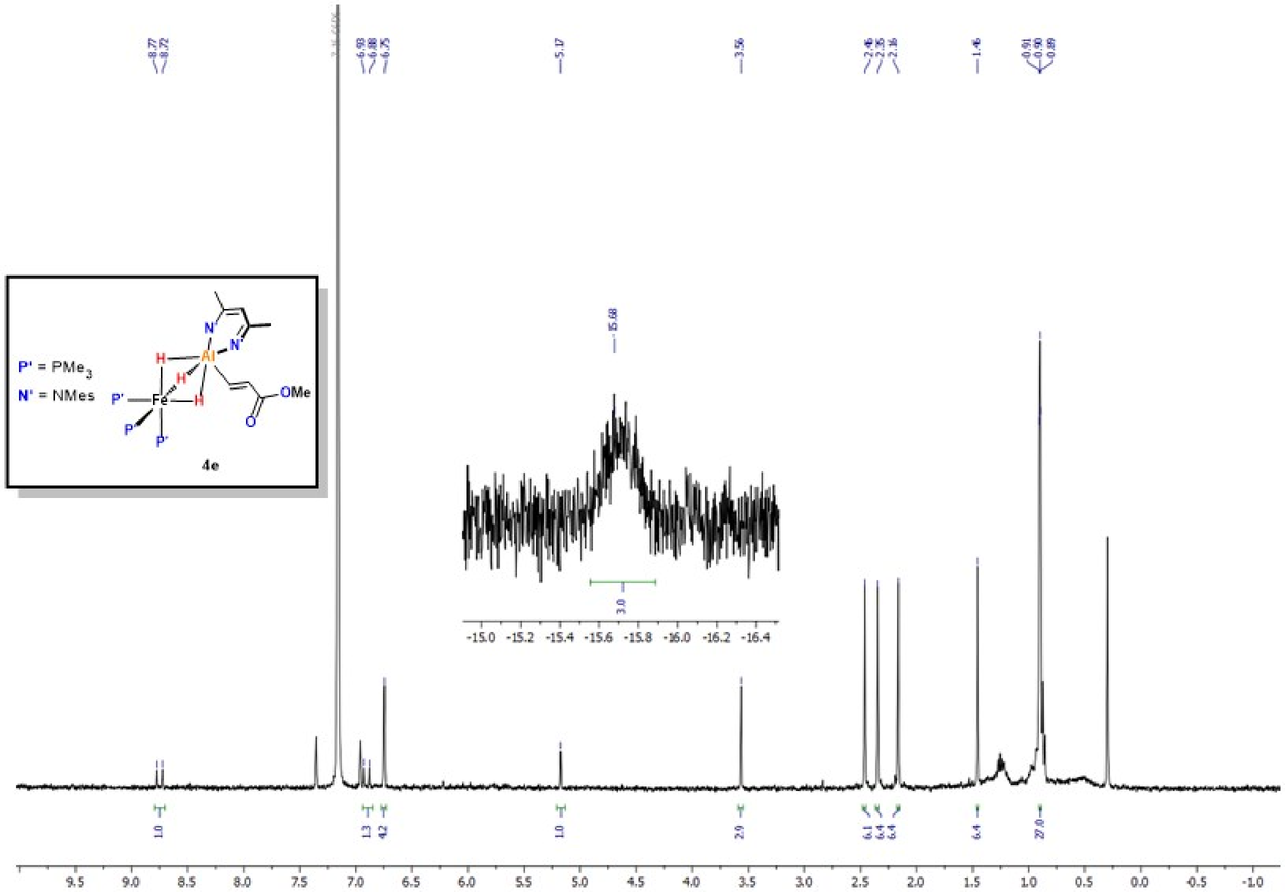

Figure S76. ^{1}H NMR of **4e** (400 MHz, C_6D_6, 298 K).

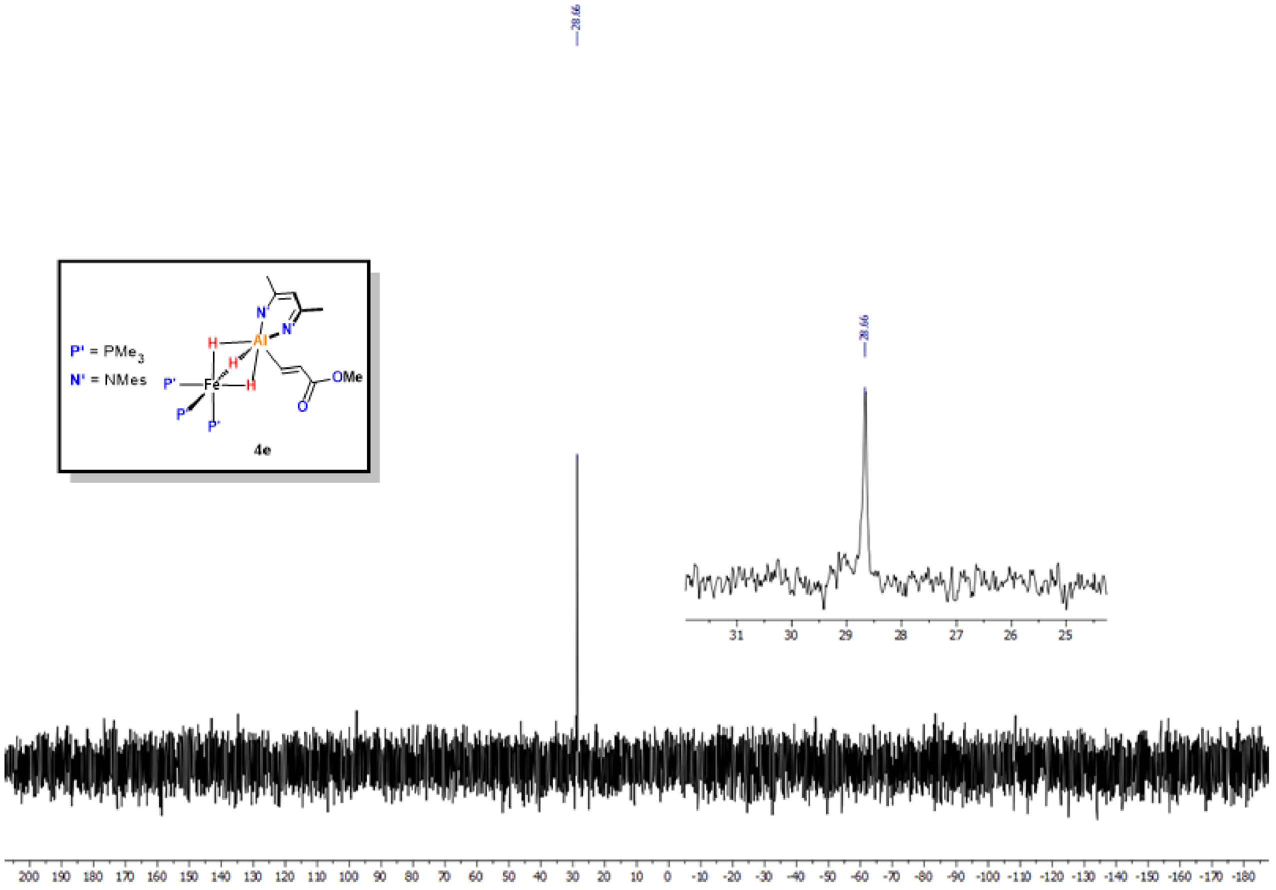

Figure S77. $^{31}P\{^{1}H\}$ NMR of **4e** (162 MHz, C_6D_6, 298 K).

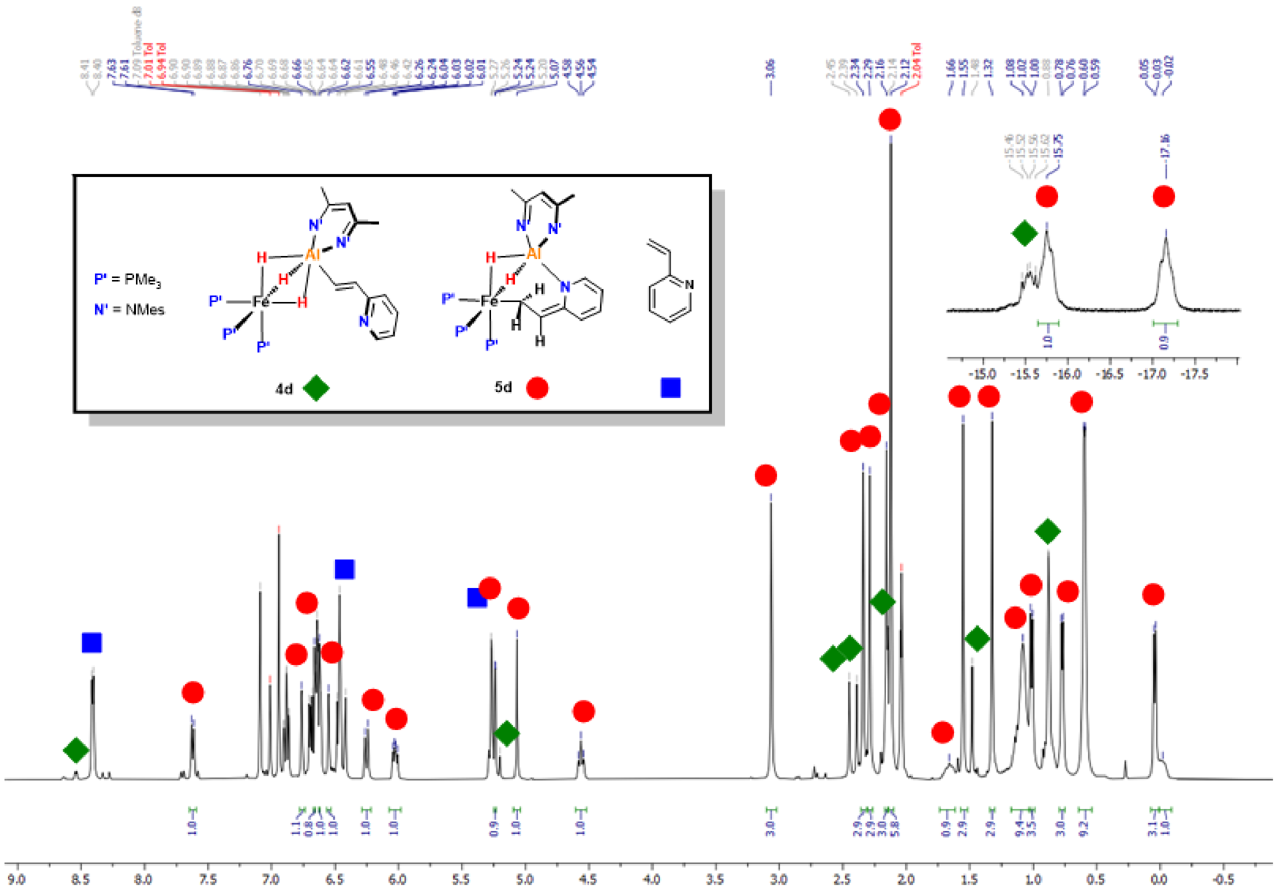

Figure S78. ^{1}H NMR of **5d** (400 MHz, toluene-d_8, 233 K).

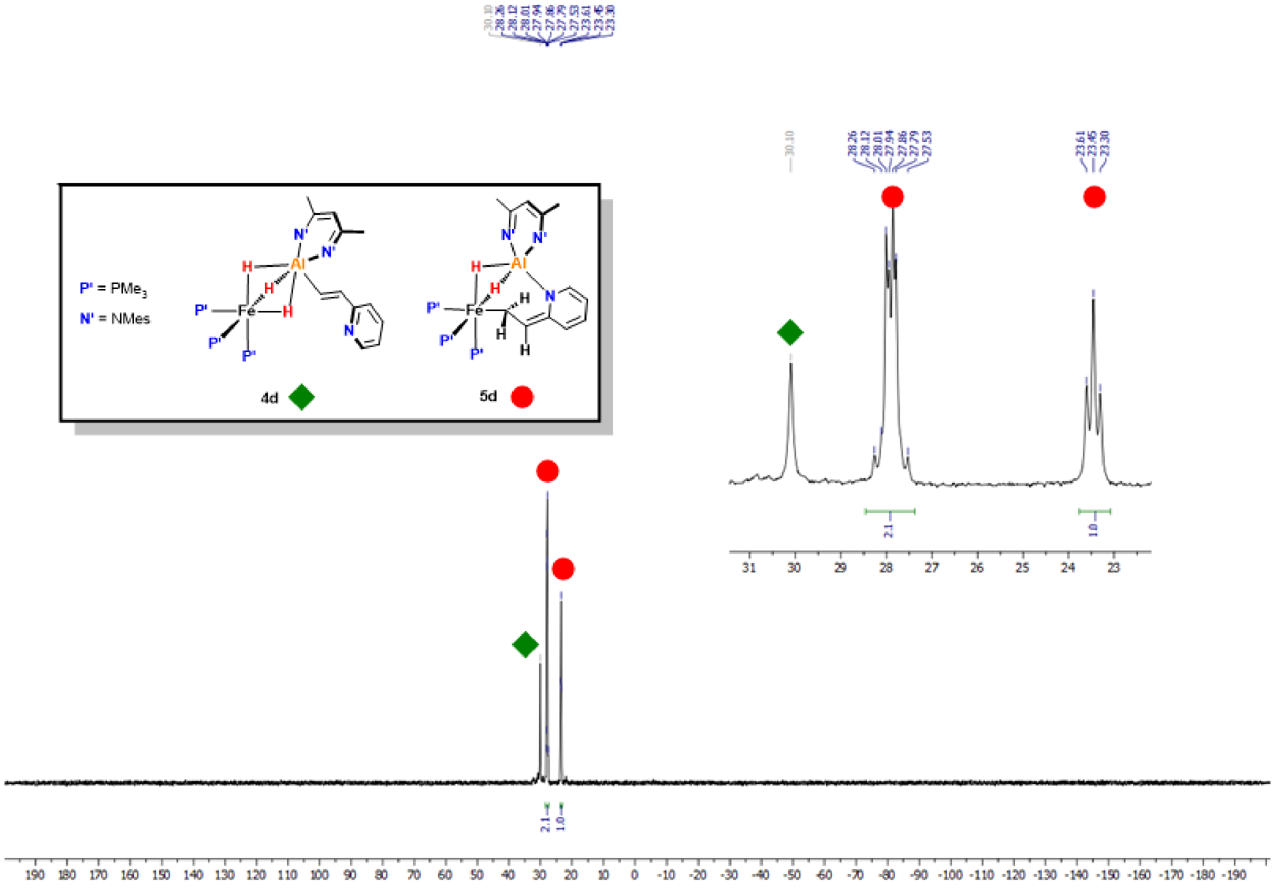

Figure S79. $^{31}P\{^{1}H\}$ NMR of **5d** (162 MHz, toluene-d_8, 233 K).

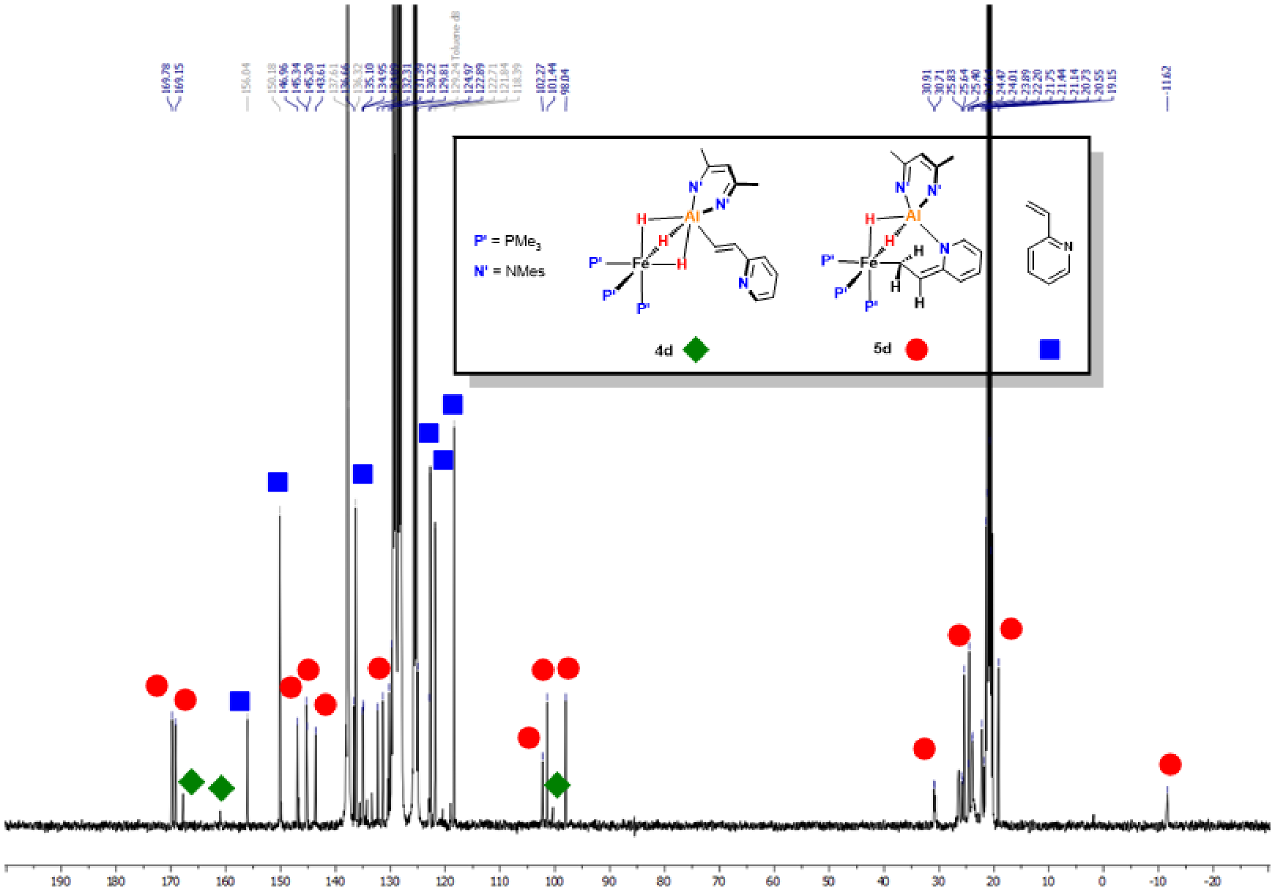

Figure S80. $^{13}C\{^1H\}$ NMR of **5d** (101 MHz, toluene-d_8, 233 K).

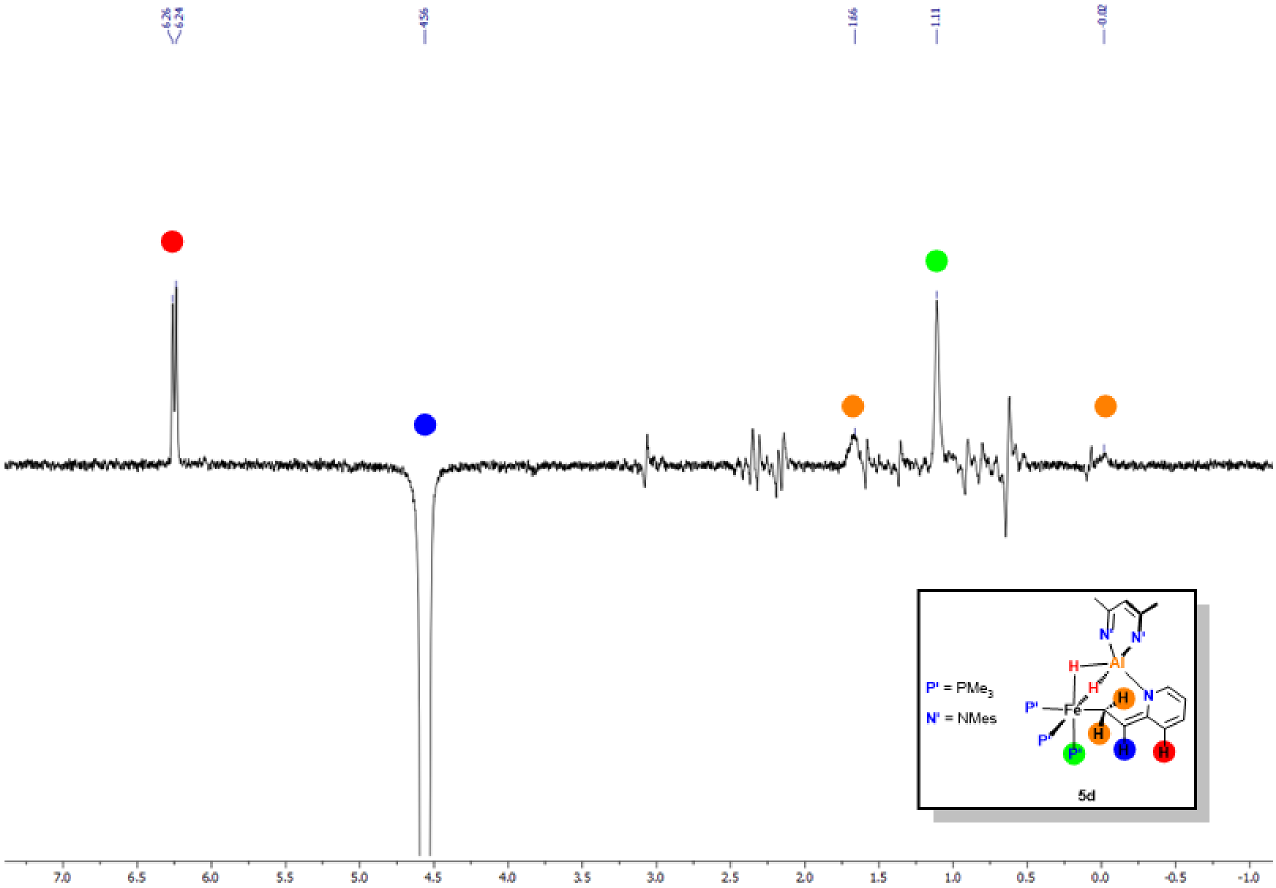

Figure S81. ^1H selective excitation NOE NMR (o2p = 4.56 ppm) of **5d** (400 MHz, toluene-d_8, 233 K).

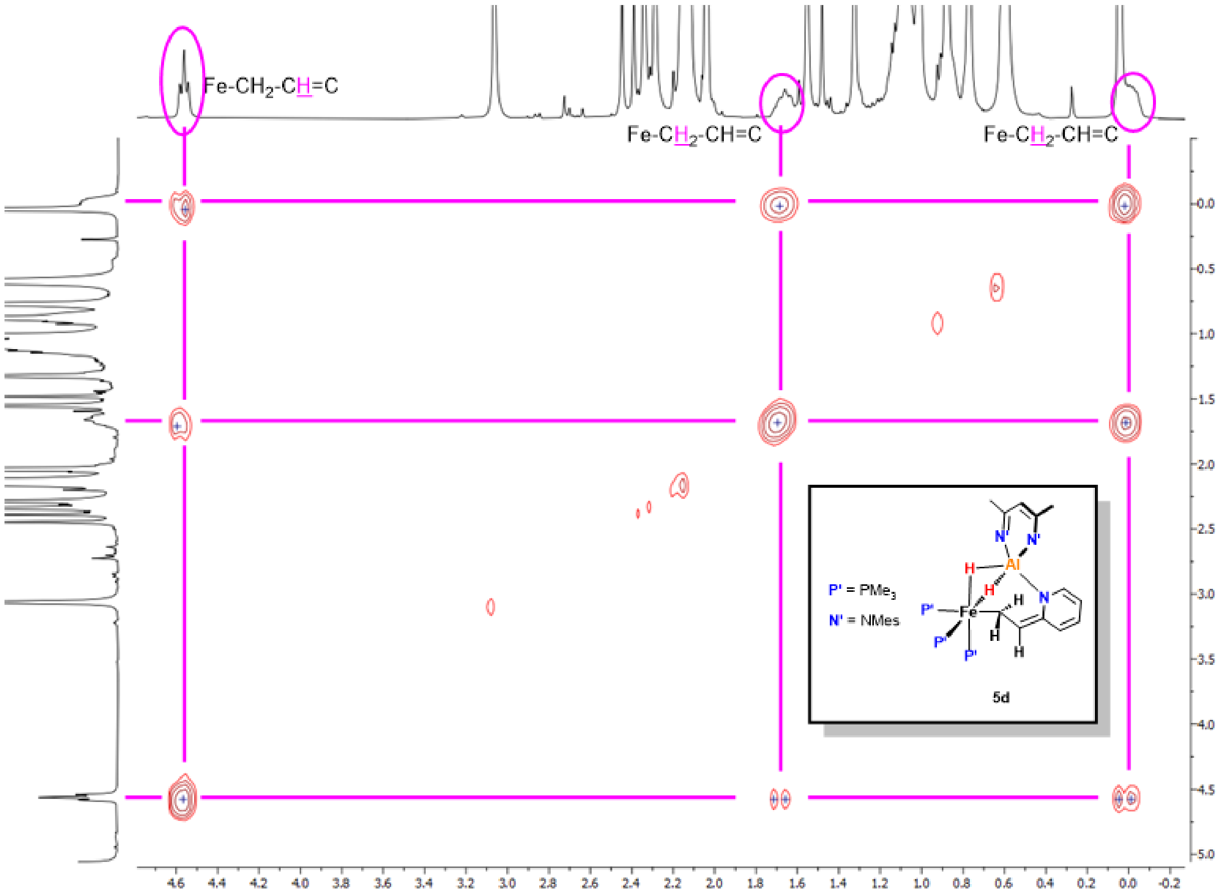

Figure S82. ^{1}H COSY NMR of **5d** (400 MHz, toluene-d_8, 233 K).

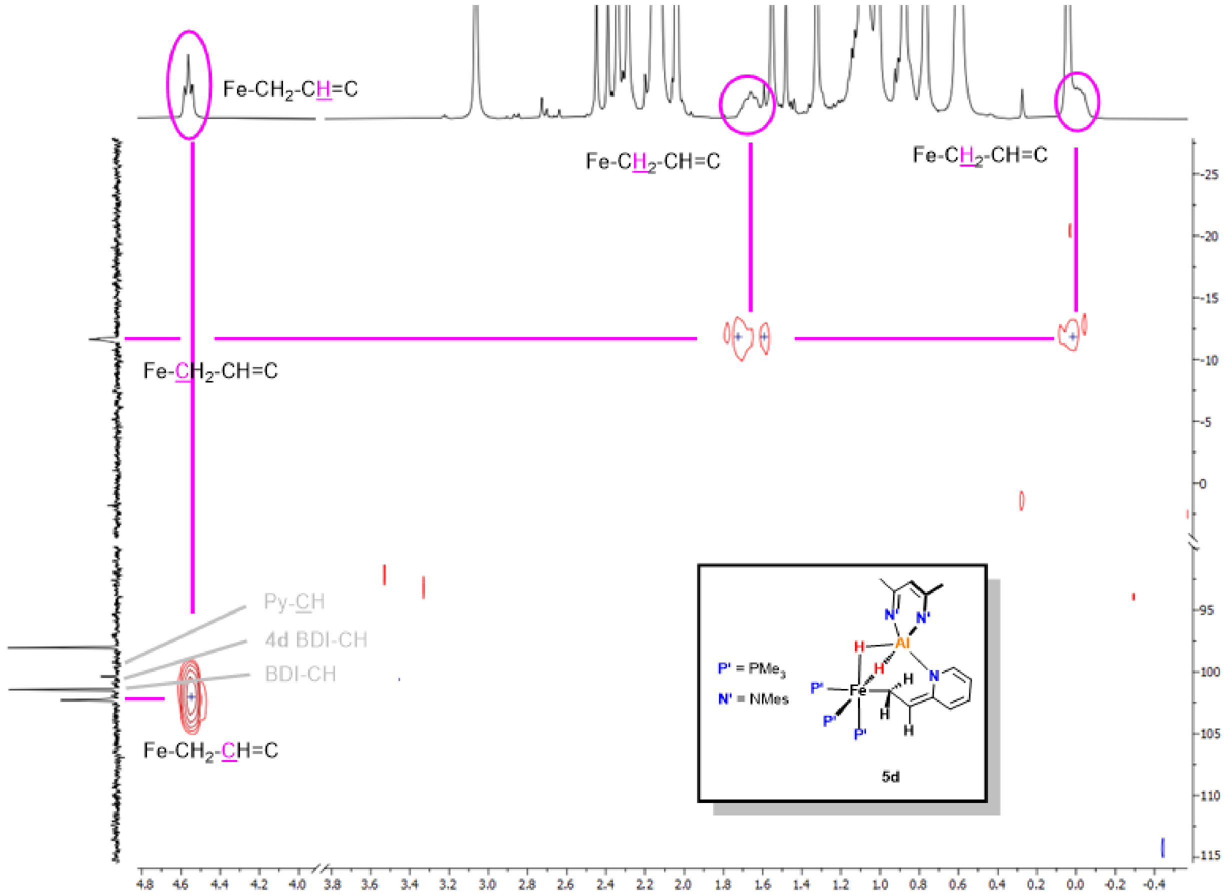

Figure S83. ^{1}H-^{13}C HSQC NMR of **5d** (toluene-d_8, 233 K).

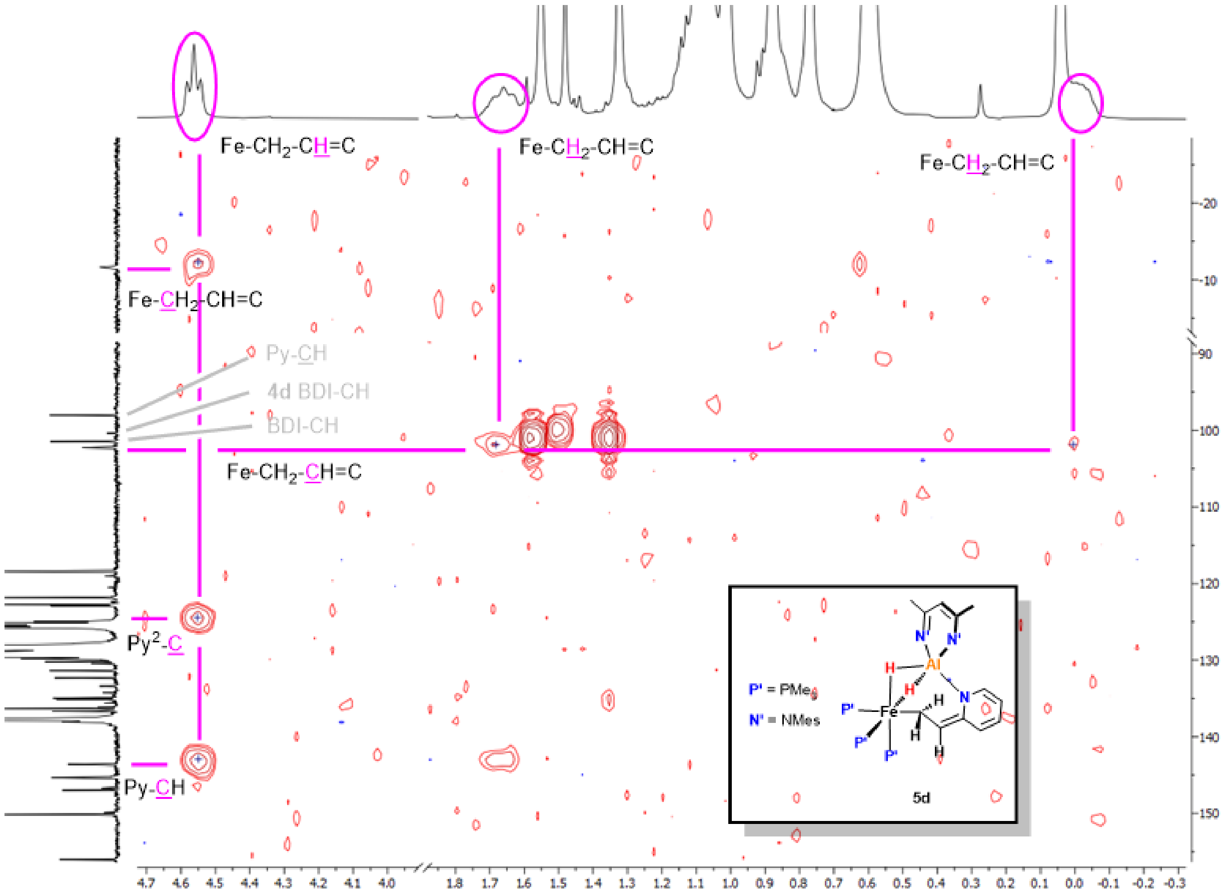

Figure S84. ^{1}H-^{13}C HMBC NMR of **5d** (toluene-d_8, 233 K).

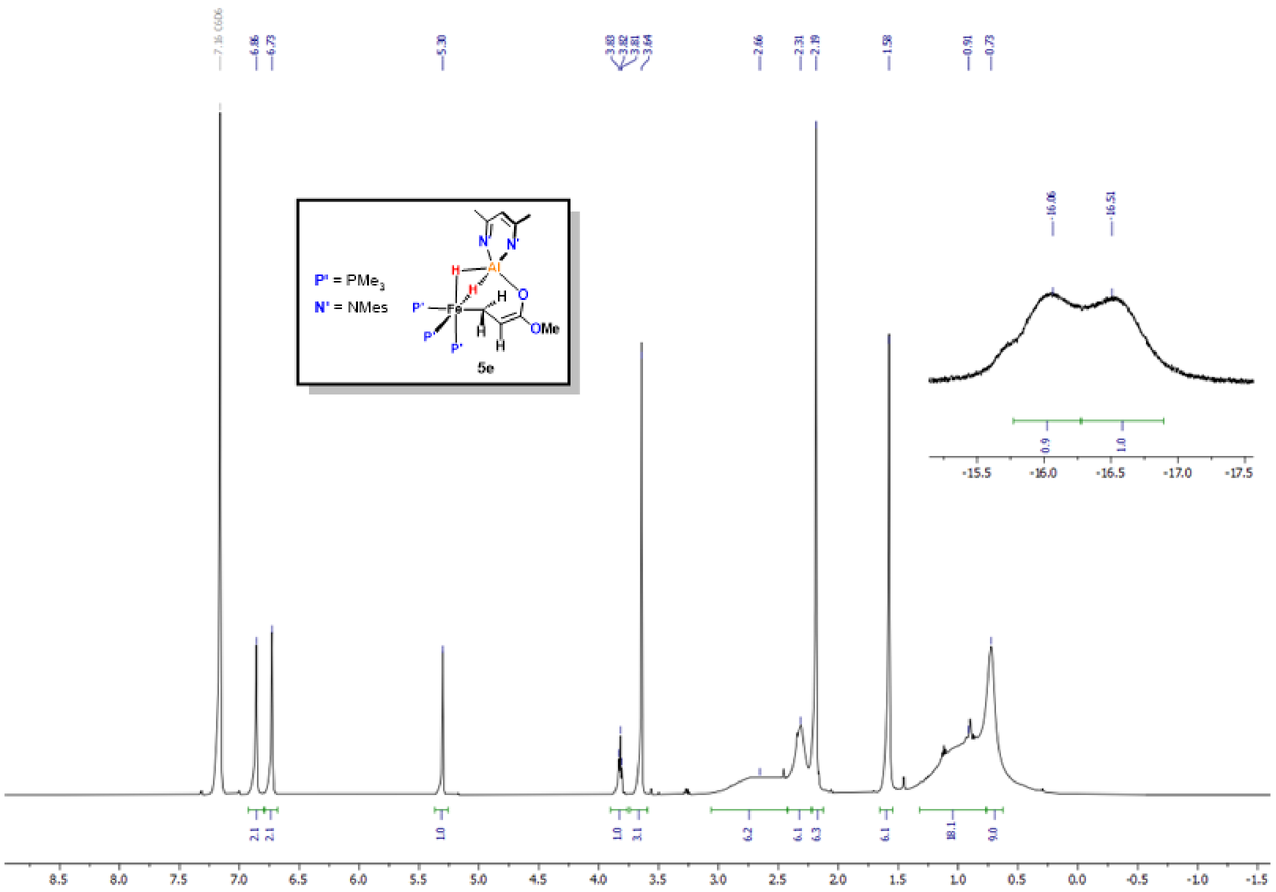

Figure S85. ^{1}H NMR of **5e** (500 MHz, C_6D_6, 298 K).

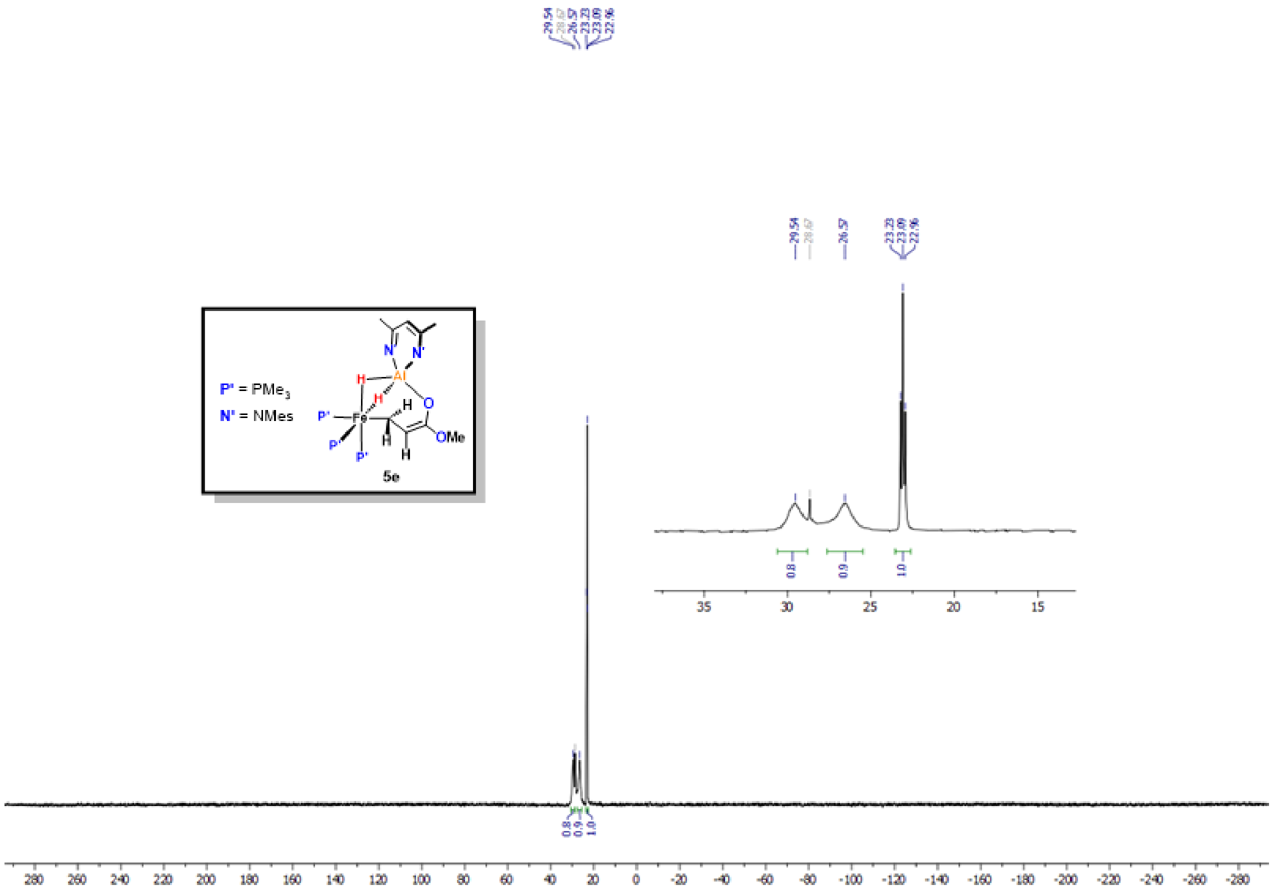

Figure S86. $^{31}P\{^{1}H\}$ NMR of **5e** (202 MHz, C_6D_6, 298 K).

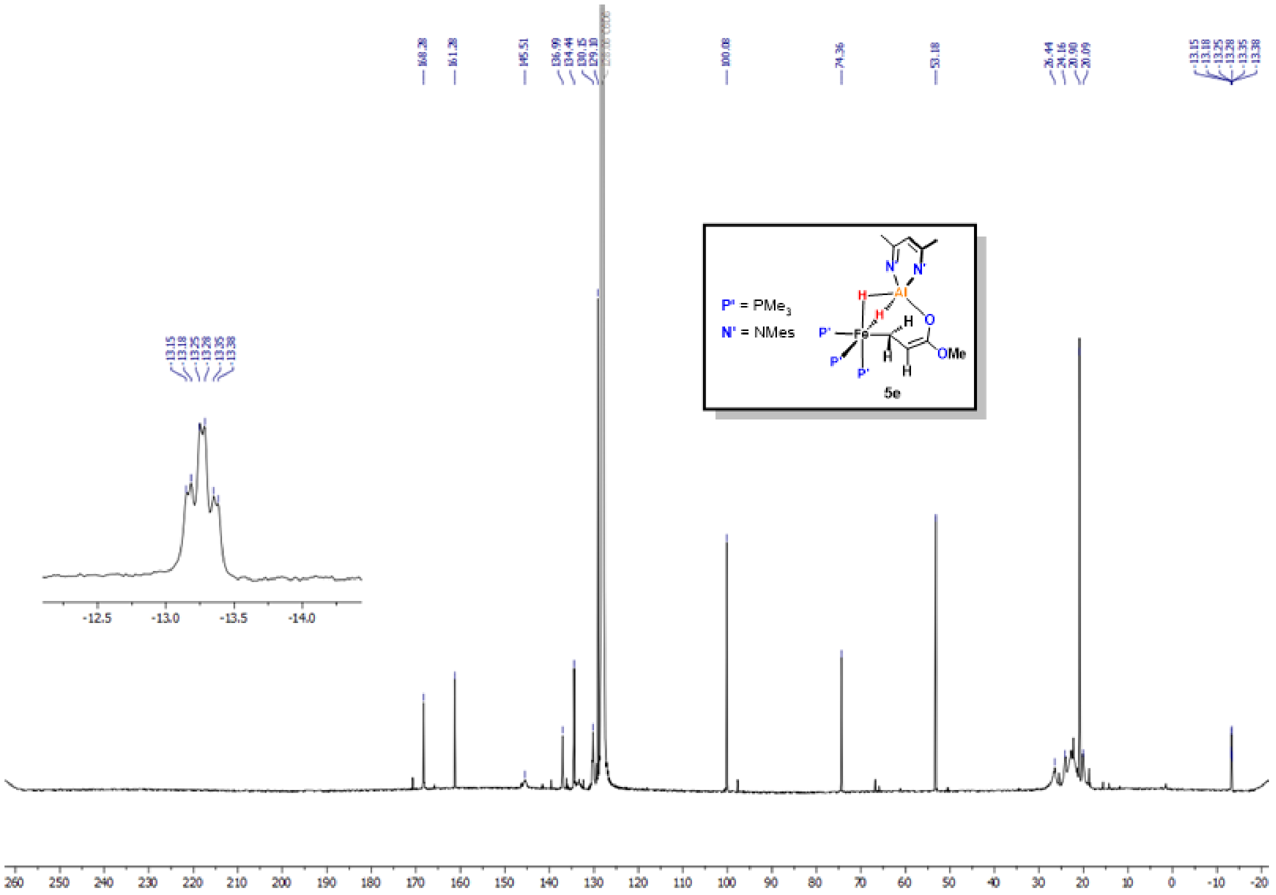

Figure S87. $^{13}C\{^{1}H\}$ NMR of **5e** (101 MHz, C_6D_6, 298 K).

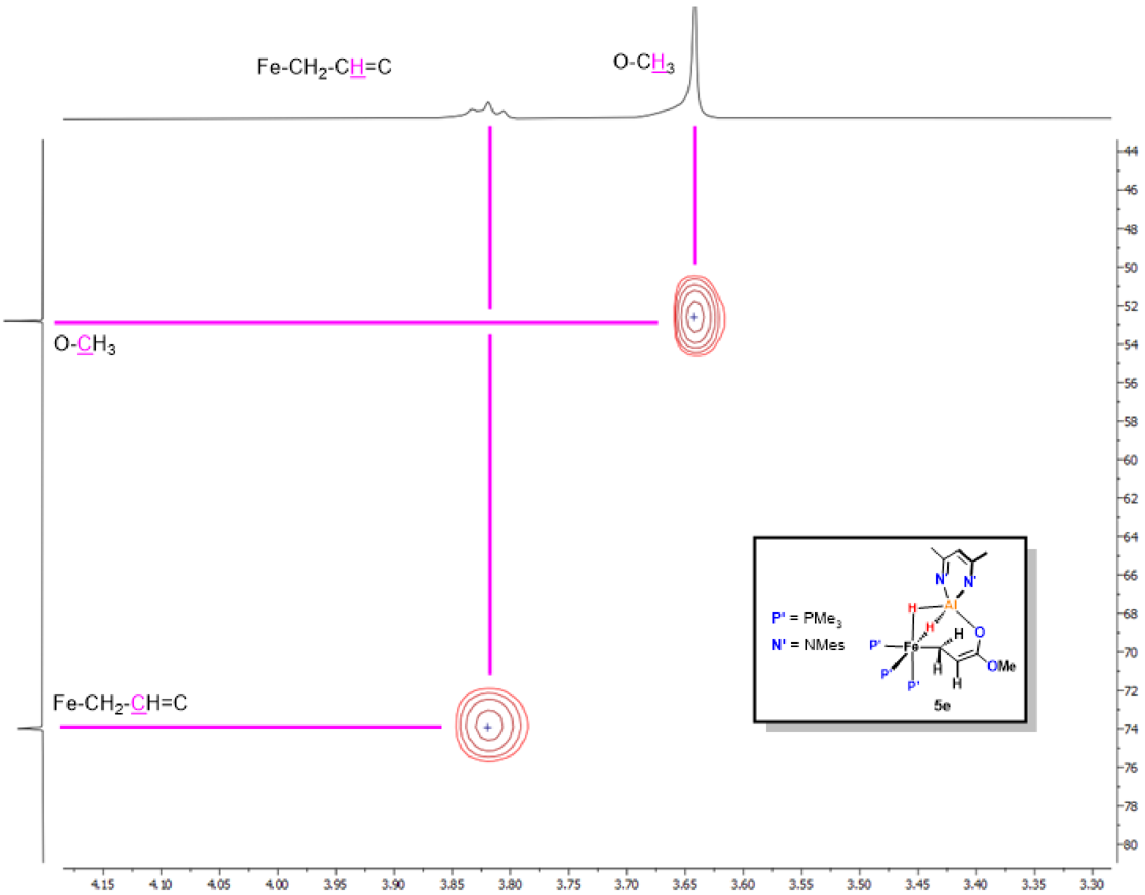

Figure S88. ^{1}H-^{13}C HSQC NMR of **5e** (C_6D_6, 298 K).

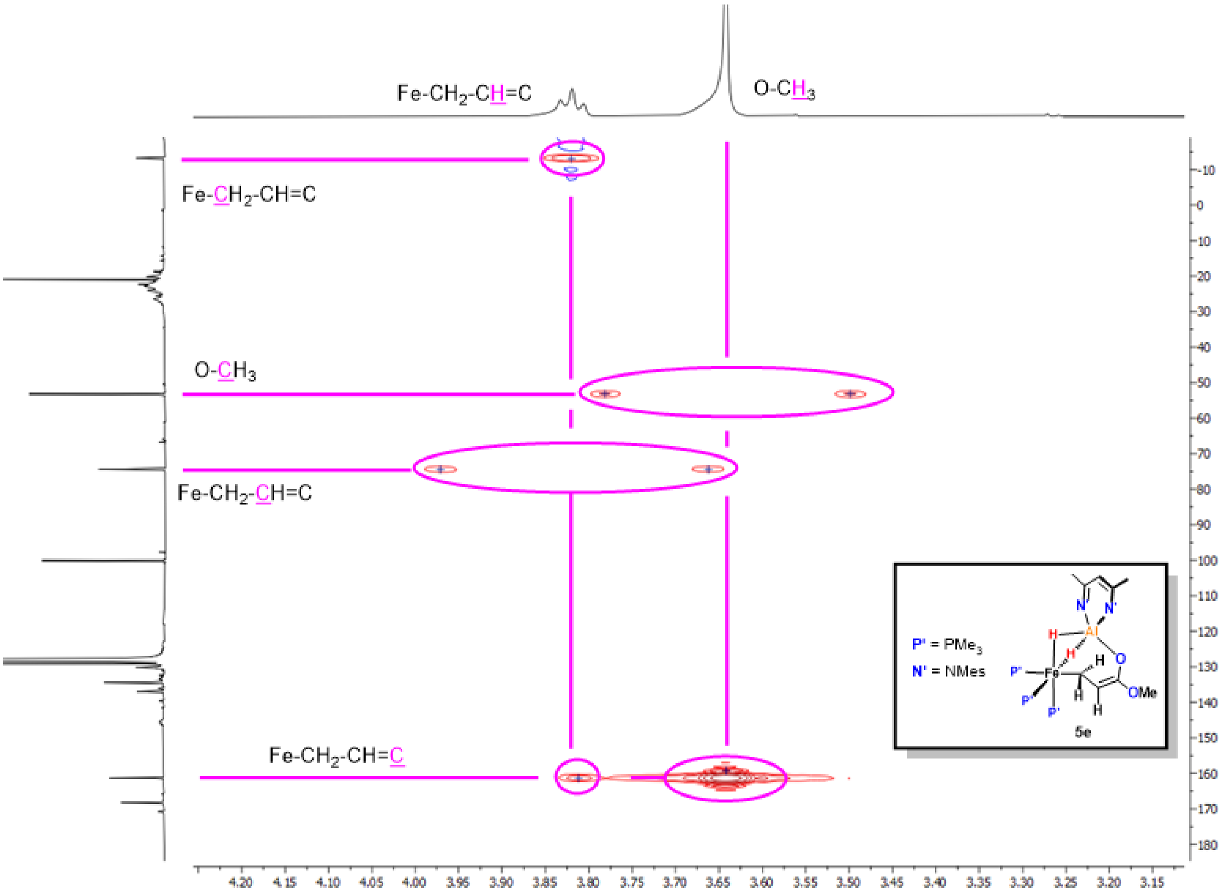

Figure S89. ^{1}H-^{13}C HMBC NMR of **5e** (C_6D_6, 298 K).

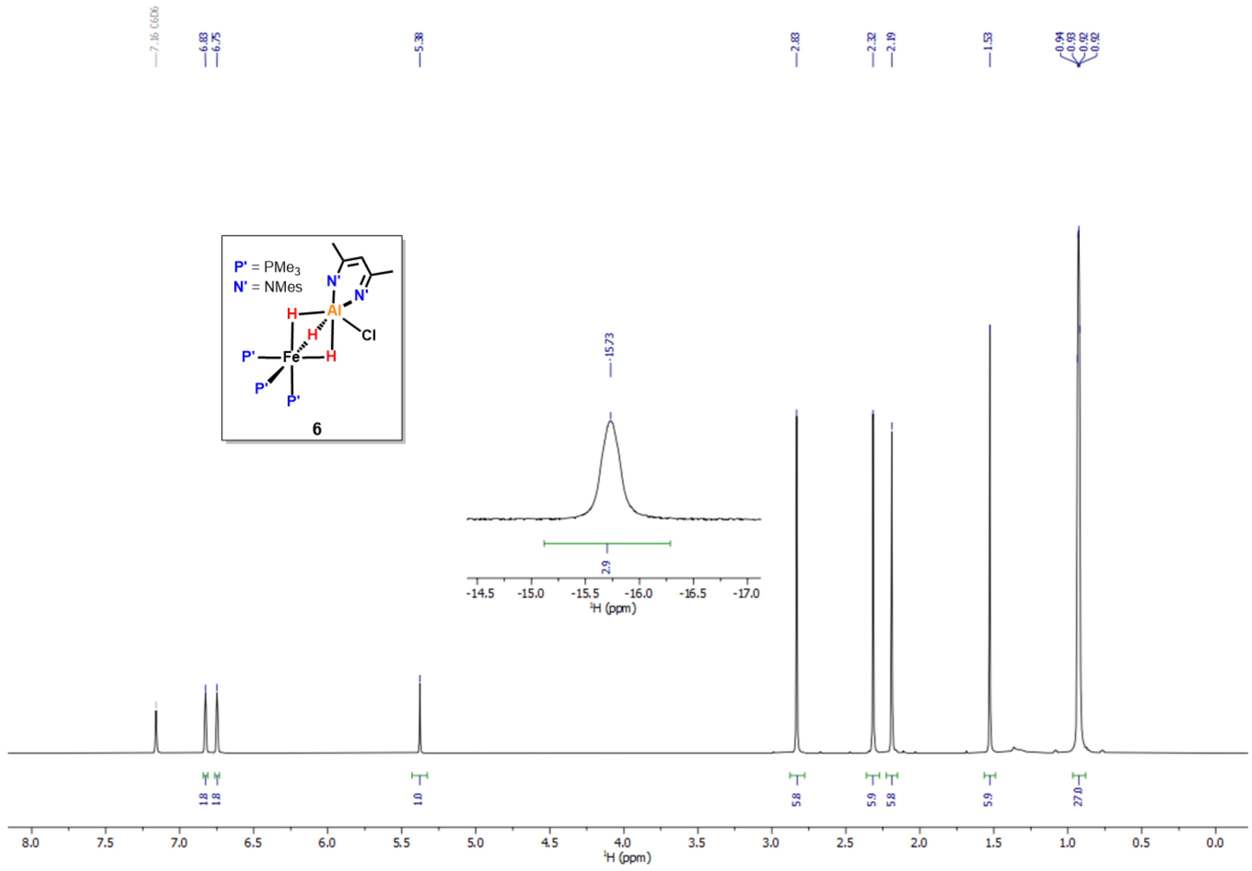

Figure S90. ^{1}H NMR of **6** (400 MHz, C_6D_6, 298 K).

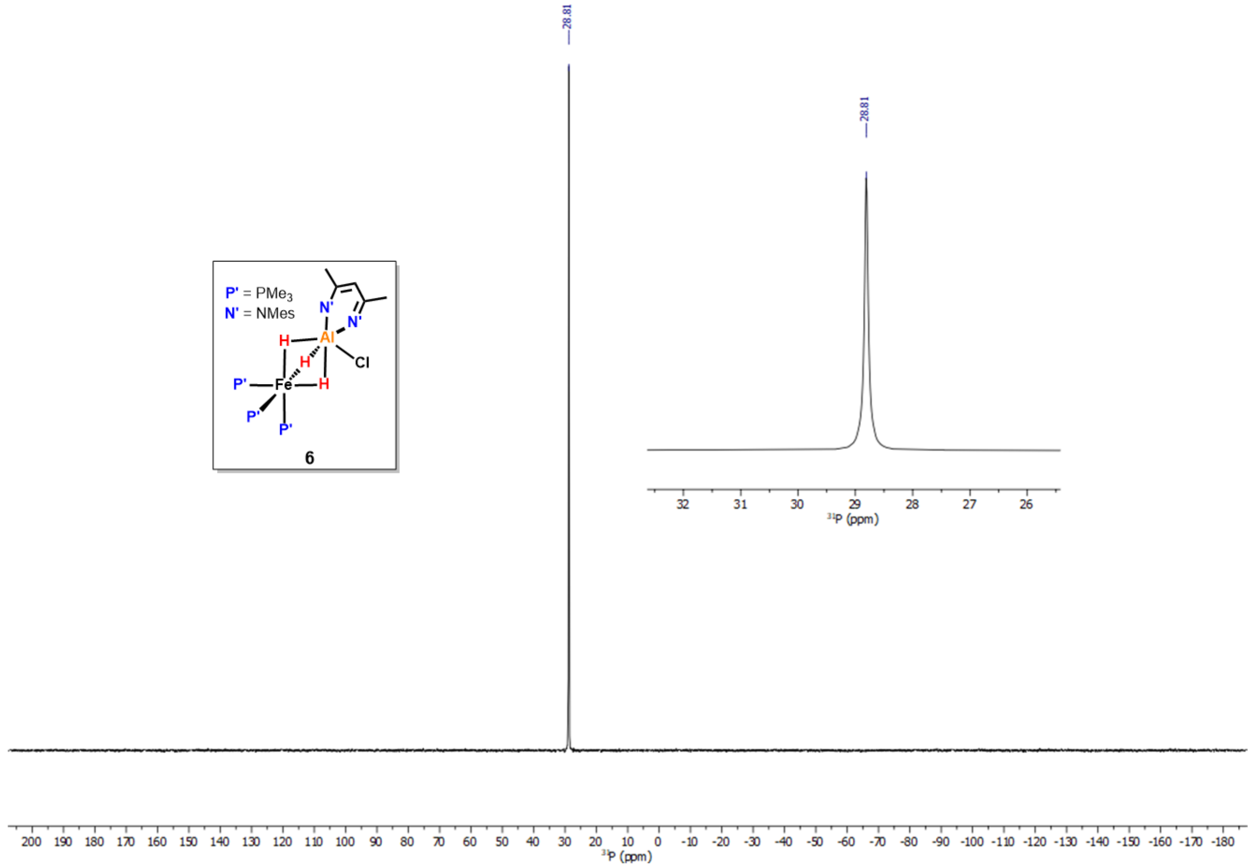

Figure S91. $^{31}P\{^{1}H\}$ NMR of **6** (162 MHz, C_6D_6, 298 K).

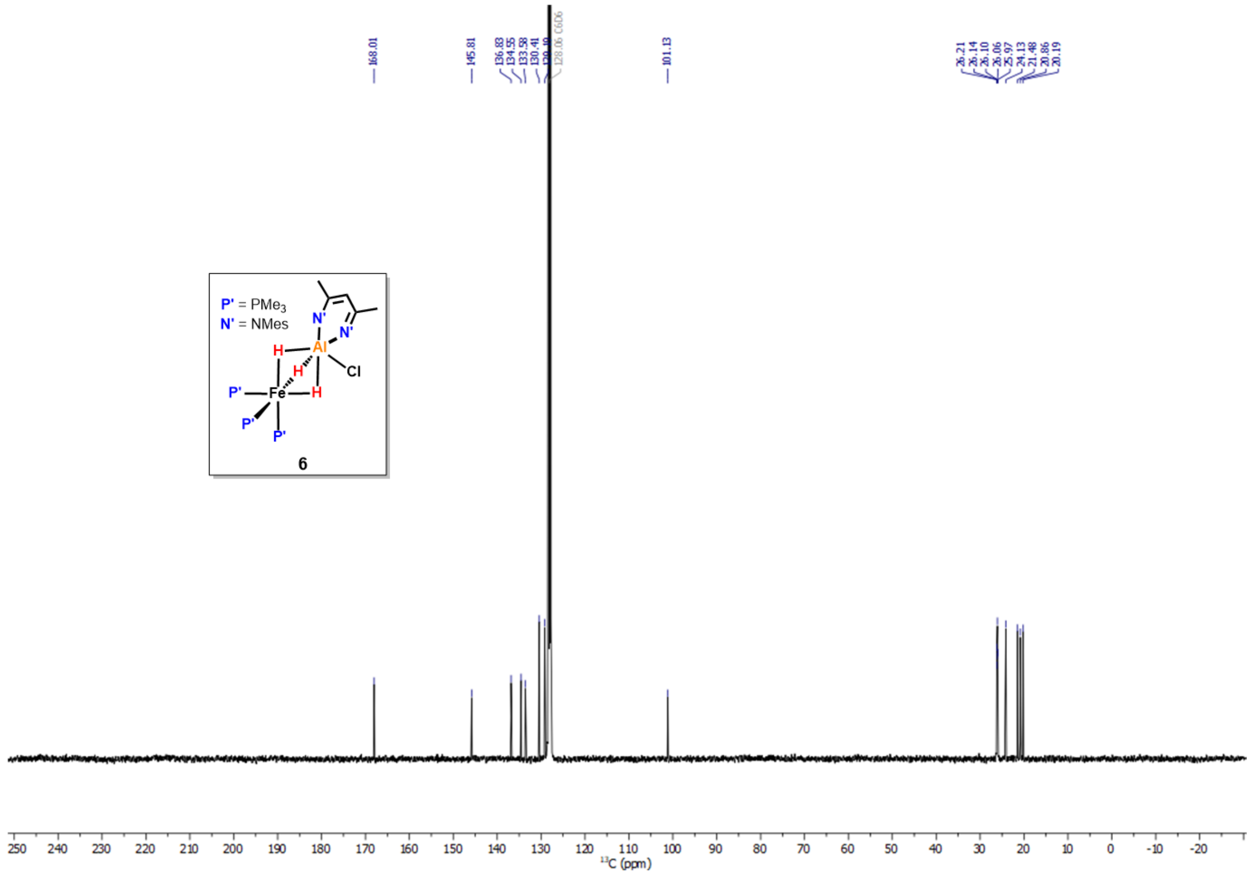

Figure S92. $^{13}C\{^1H\}$ NMR of **6** (101 MHz, C_6D_6, 298 K).

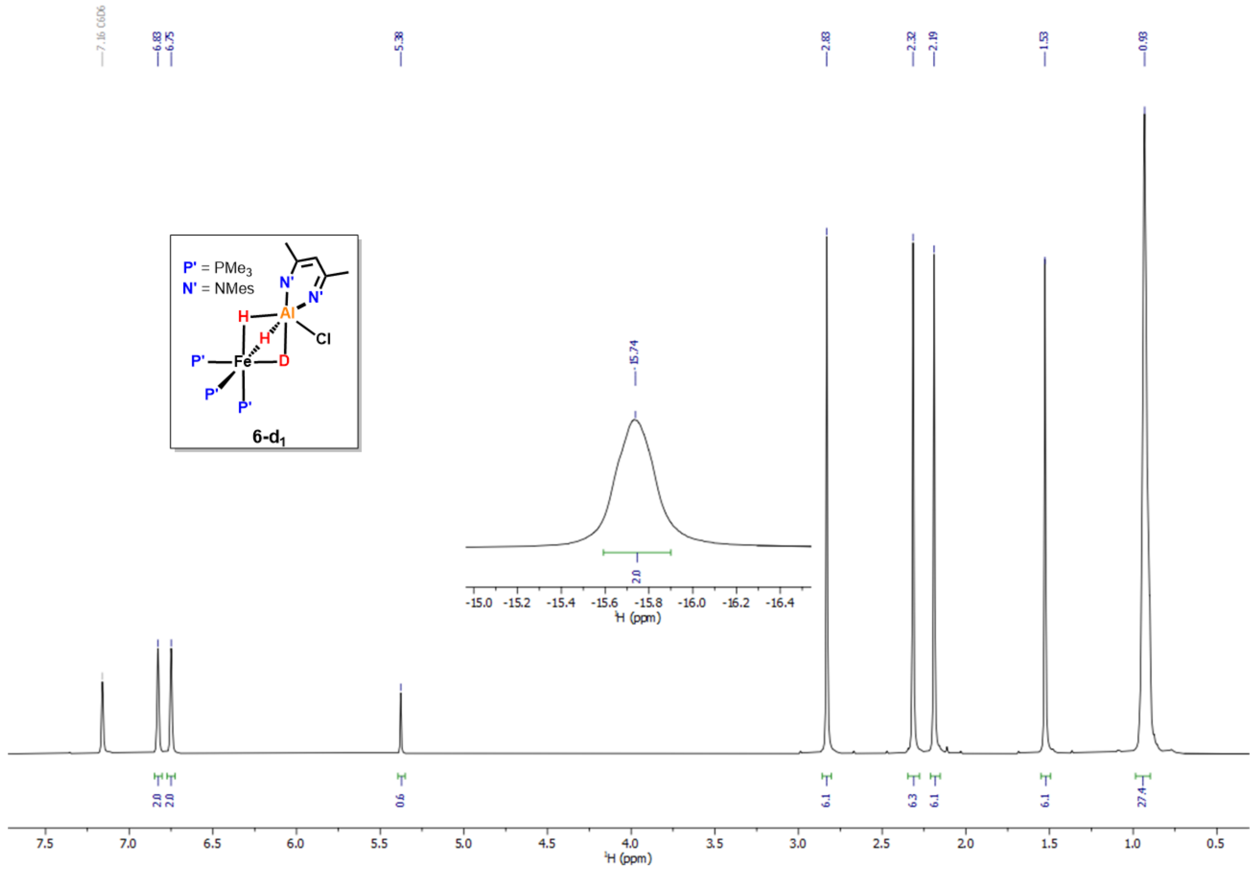

Figure S93. ^{1}H NMR of **6-d$_1$** (400 MHz, C_6D_6, 298 K).

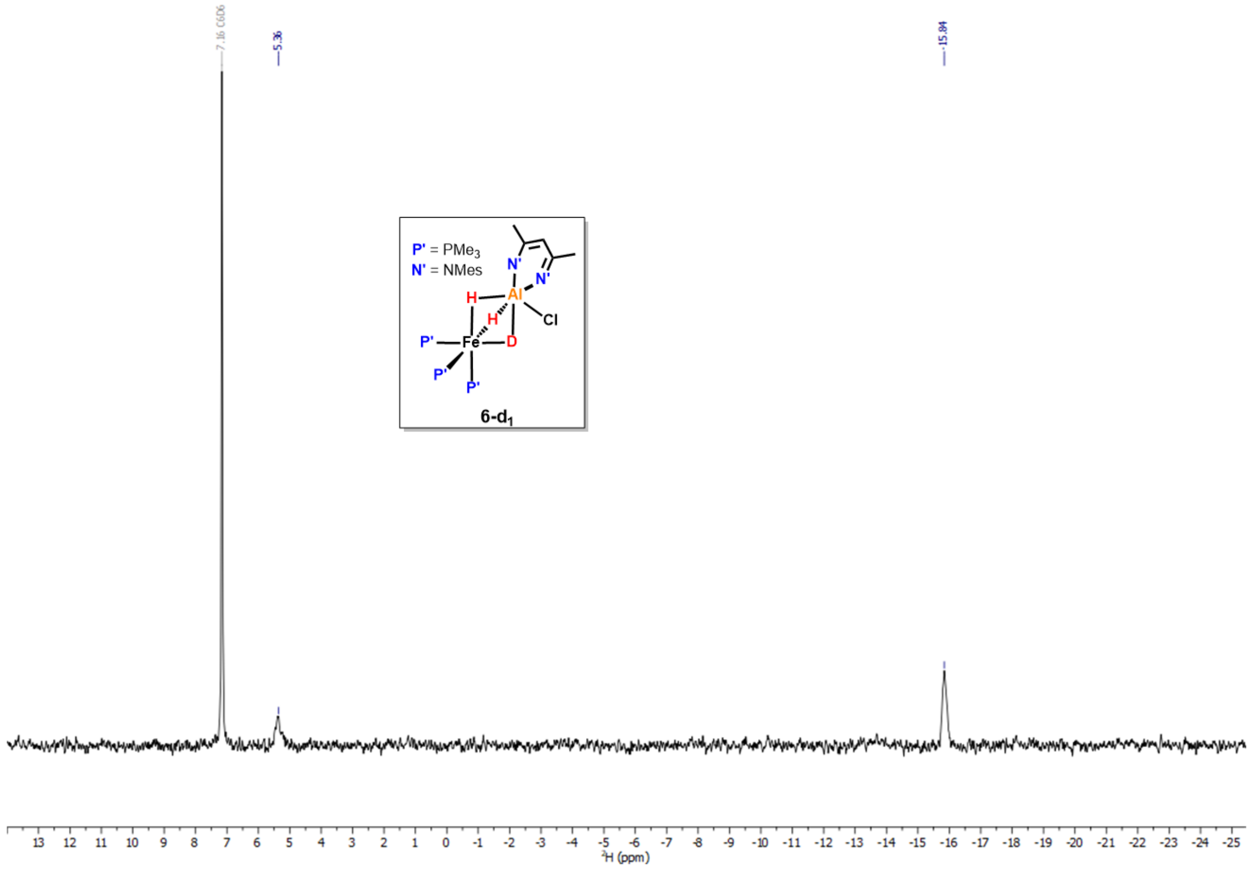

Figure S94. ^{2}H NMR of **6-d$_1$** (400 MHz, C_6H_6, 298 K).

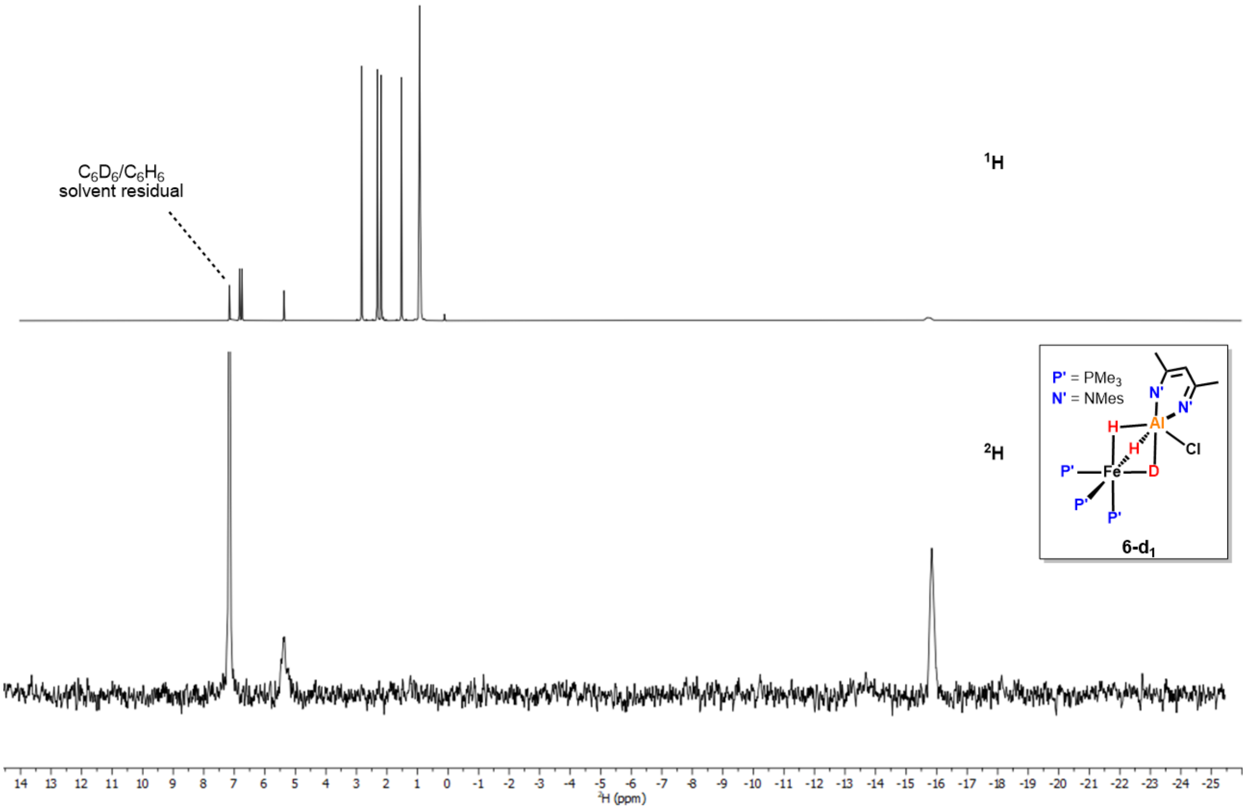

Figure S95.Stacked ^{1}H and ^{2}H NMRs of **6-d₁.** Top: ^{1}H NMR of **6-d₁** (400 MHz, C_6D_6, 298 K). Bottom: ^{2}H NMR of **6-d₁** (400 MHz, C_6H_6, 298 K).

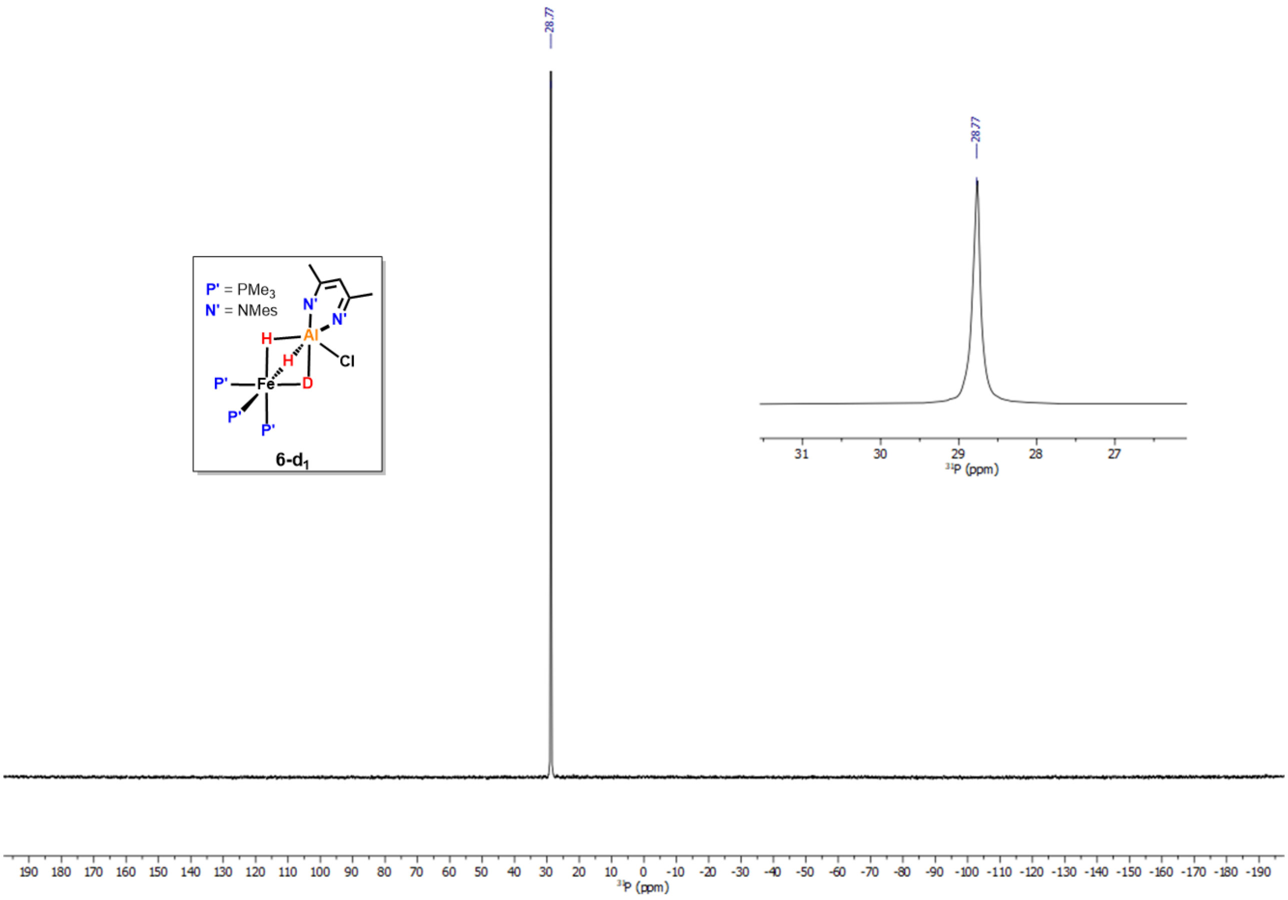

Figure S96. $^{31}P\{^{1}H\}$ NMR of **6-d₁** (162 MHz, C_6D_6, 298 K).

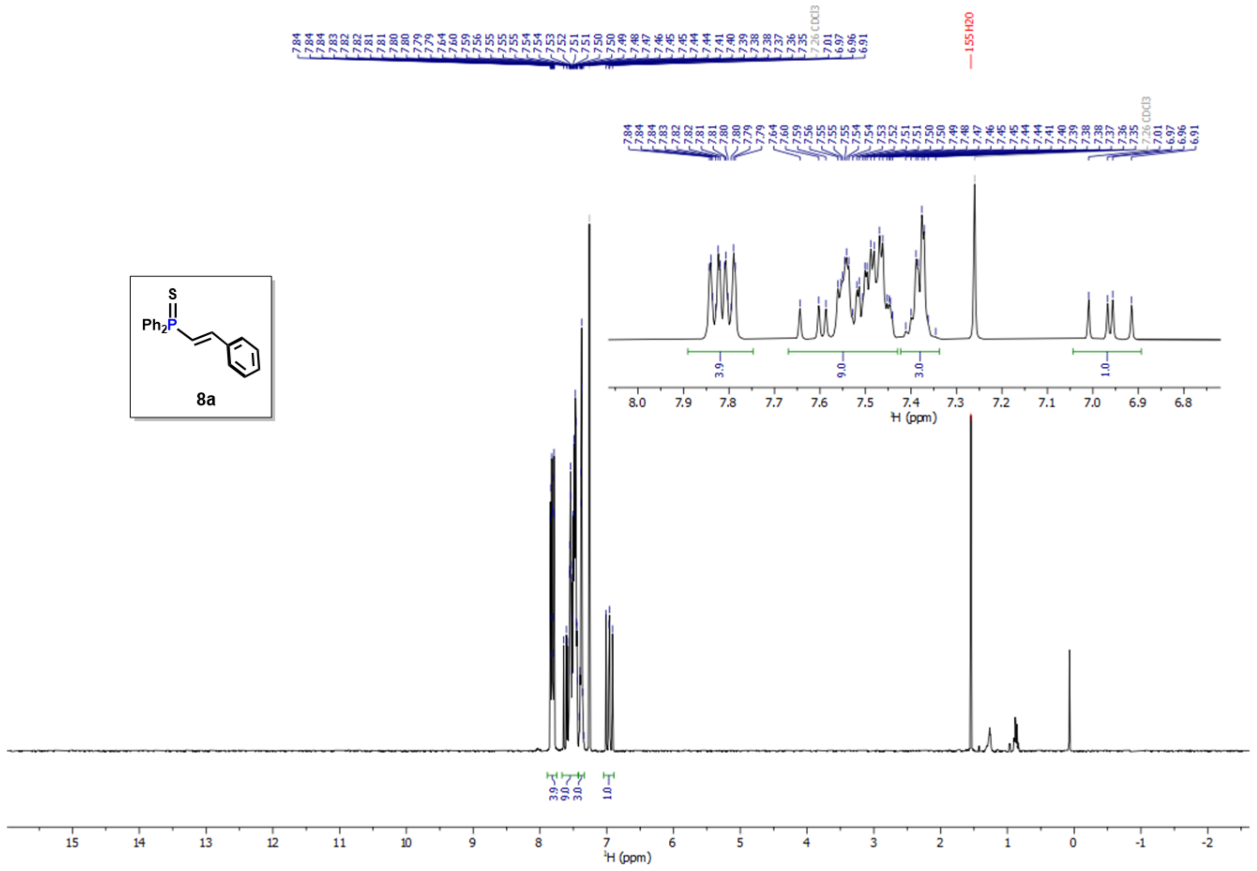

Figure S97. ^{1}H NMR of **8a** (400 MHz, $CDCl_3$, 298 K).

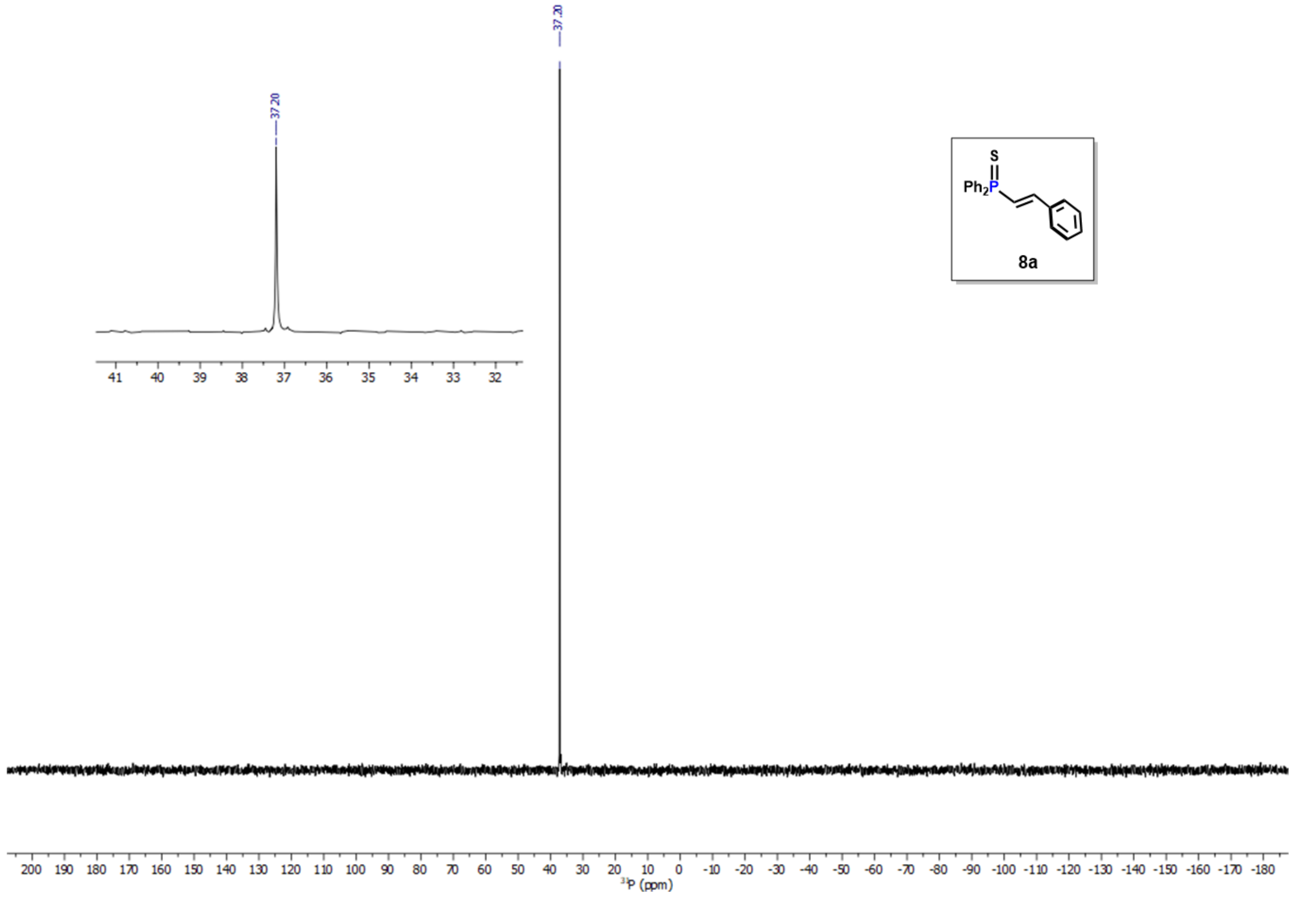

Figure S98. $^{31}P\{^{1}H\}$ NMR of **8a** (162 MHz, $CDCl_3$, 298 K).

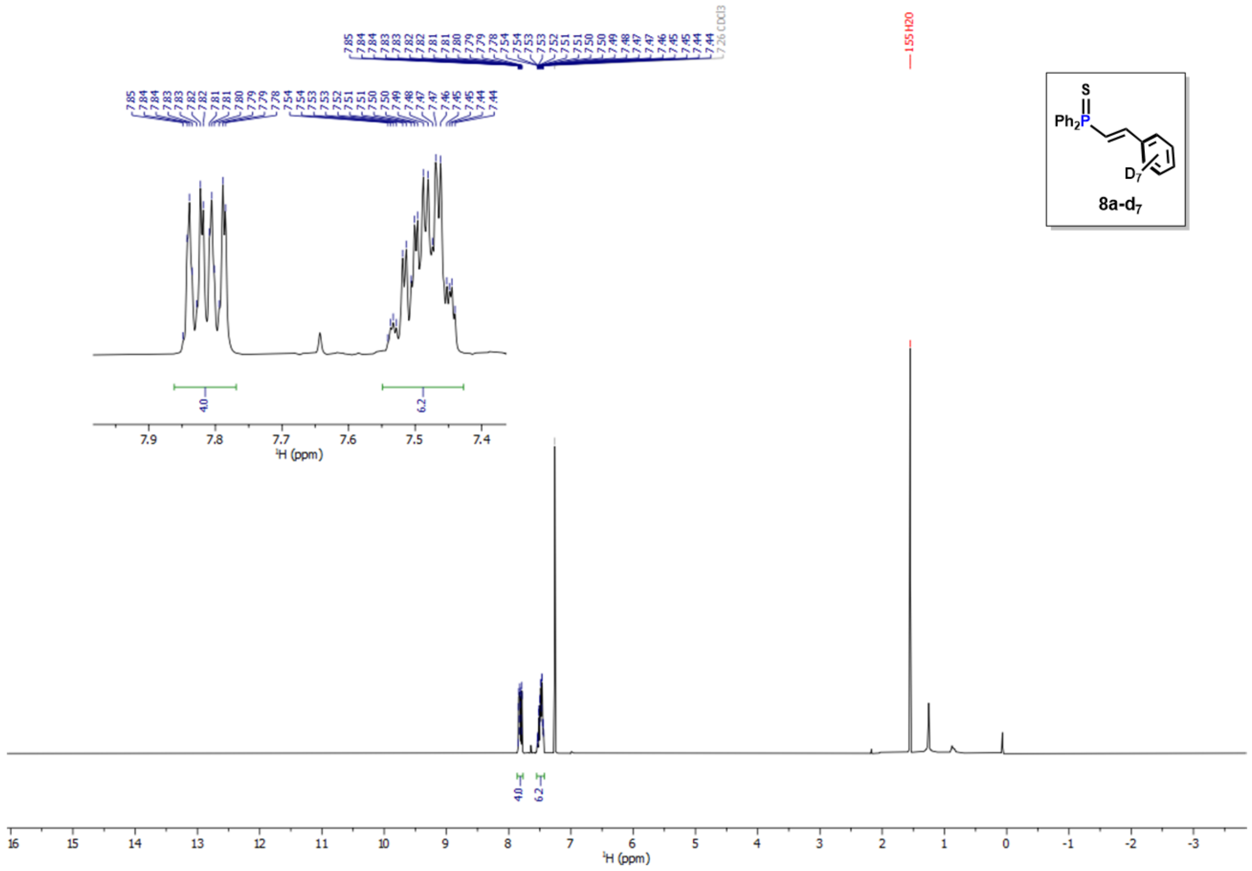

Figure S99. ^{2}H NMR of **8a** (400 MHz, $CDCl_3$, 298 K).

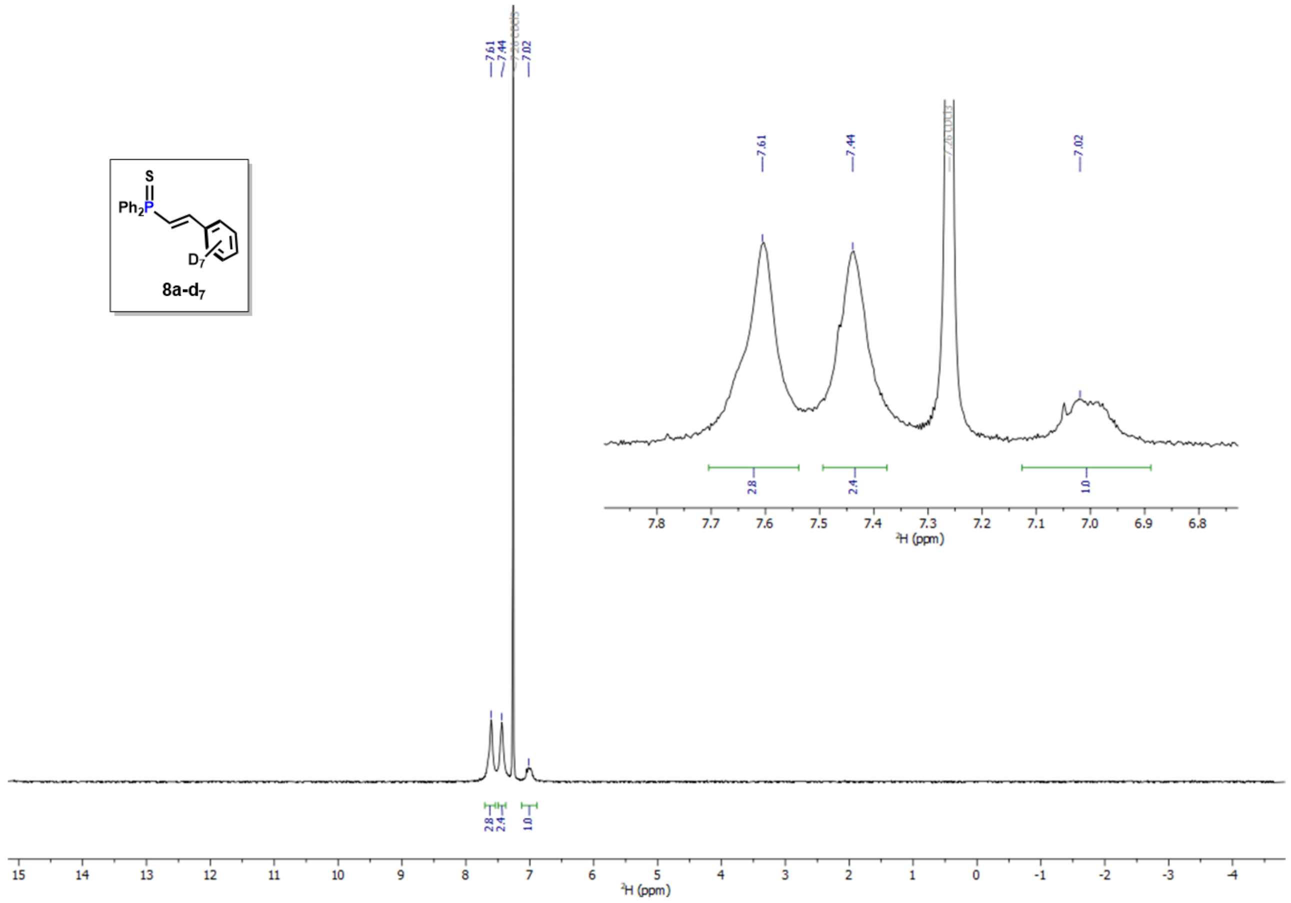

Figure S100. ^{2}H NMR of **8a-d$_7$** (500 MHz, $CHCl_3$, 298 K).

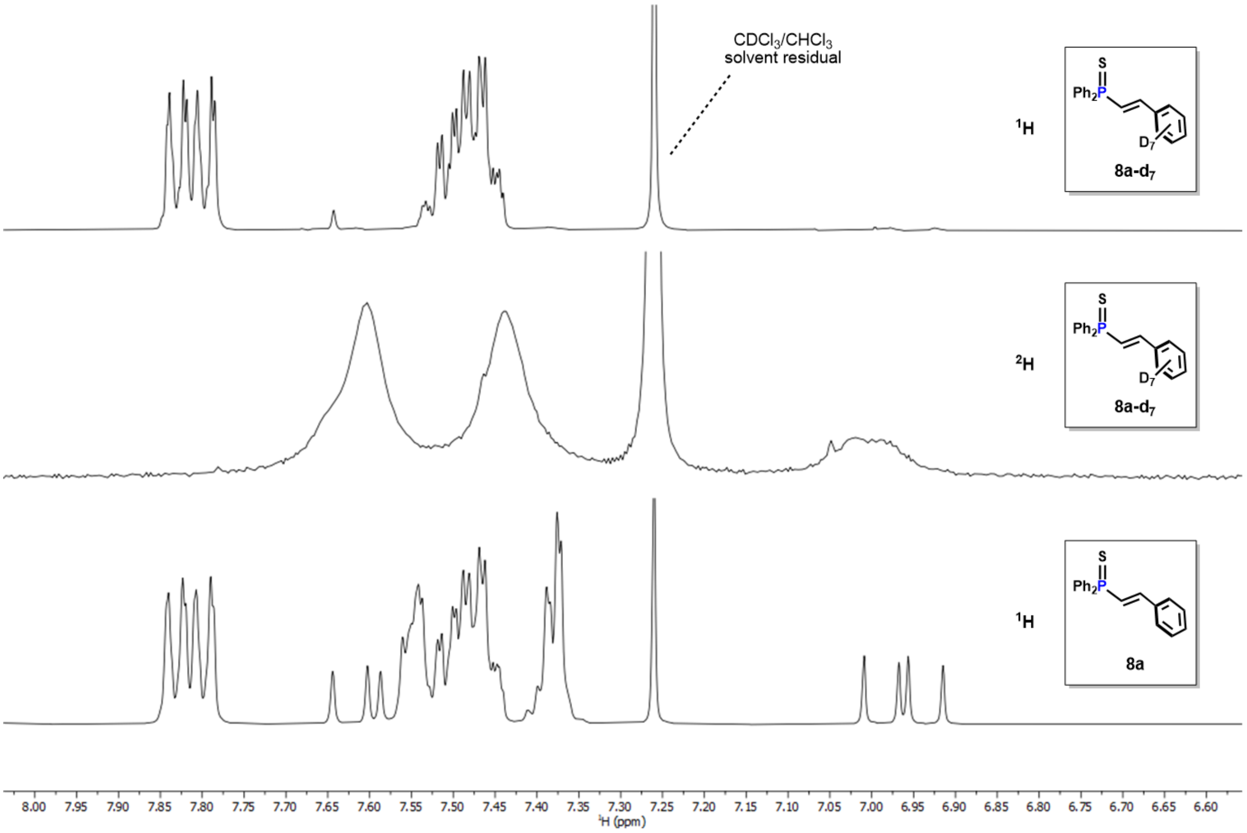

Figure S101. Stacked ^{1}H NMR and ^{2}H NMRs of **8a-d₇** and ^{1}H NMR of **8a**. Top: ^{1}H NMR of **8a-d₇** (400 MHz, $CDCl_3$, 298 K). Middle: ^{2}H NMR of **8a-d₇** (500 MHz, $CHCl_3$, 298 K). Bottom: ^{1}H NMR of **8a** (400 MHz, $CDCl_3$, 298 K).

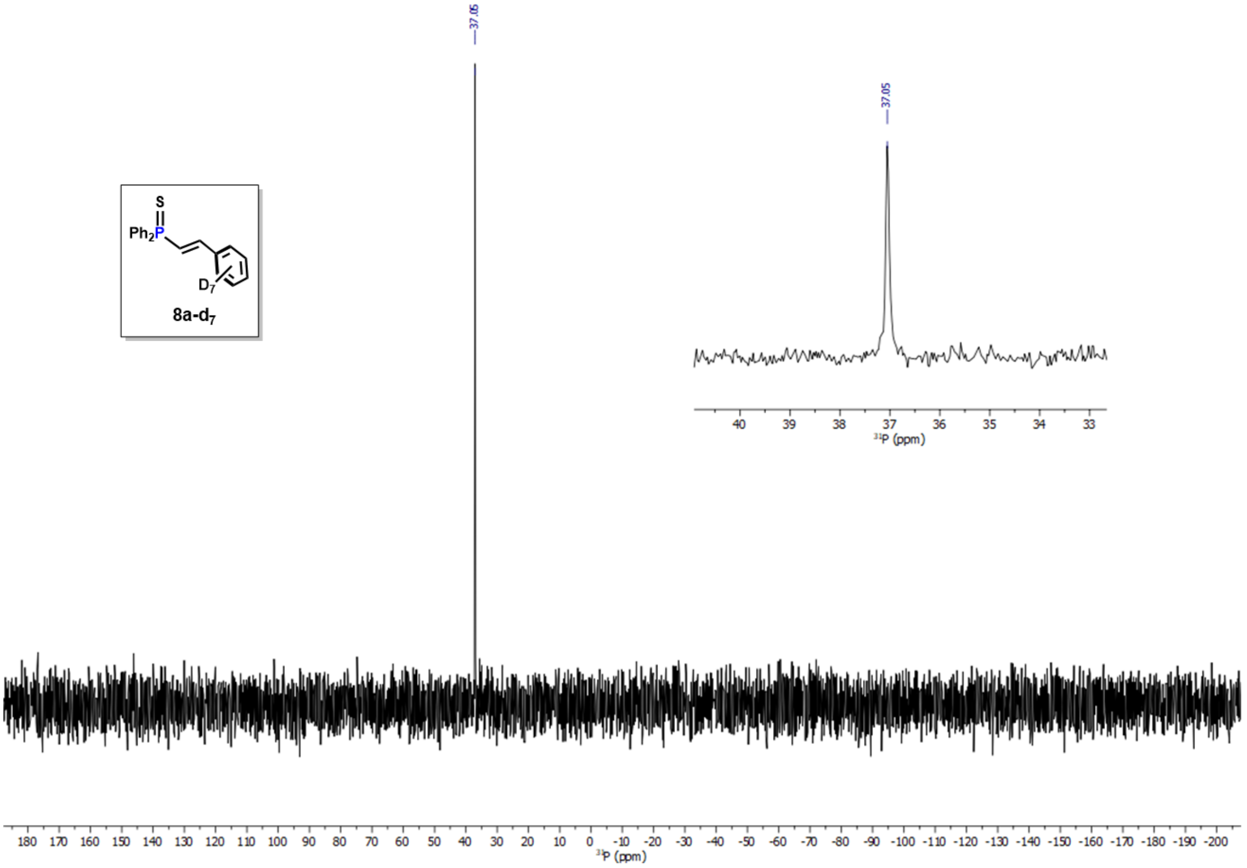

Figure S102. $^{31}P\{^{1}H\}$ NMR of **8a-d₇** (162 MHz, $CDCl_3$, 298 K).

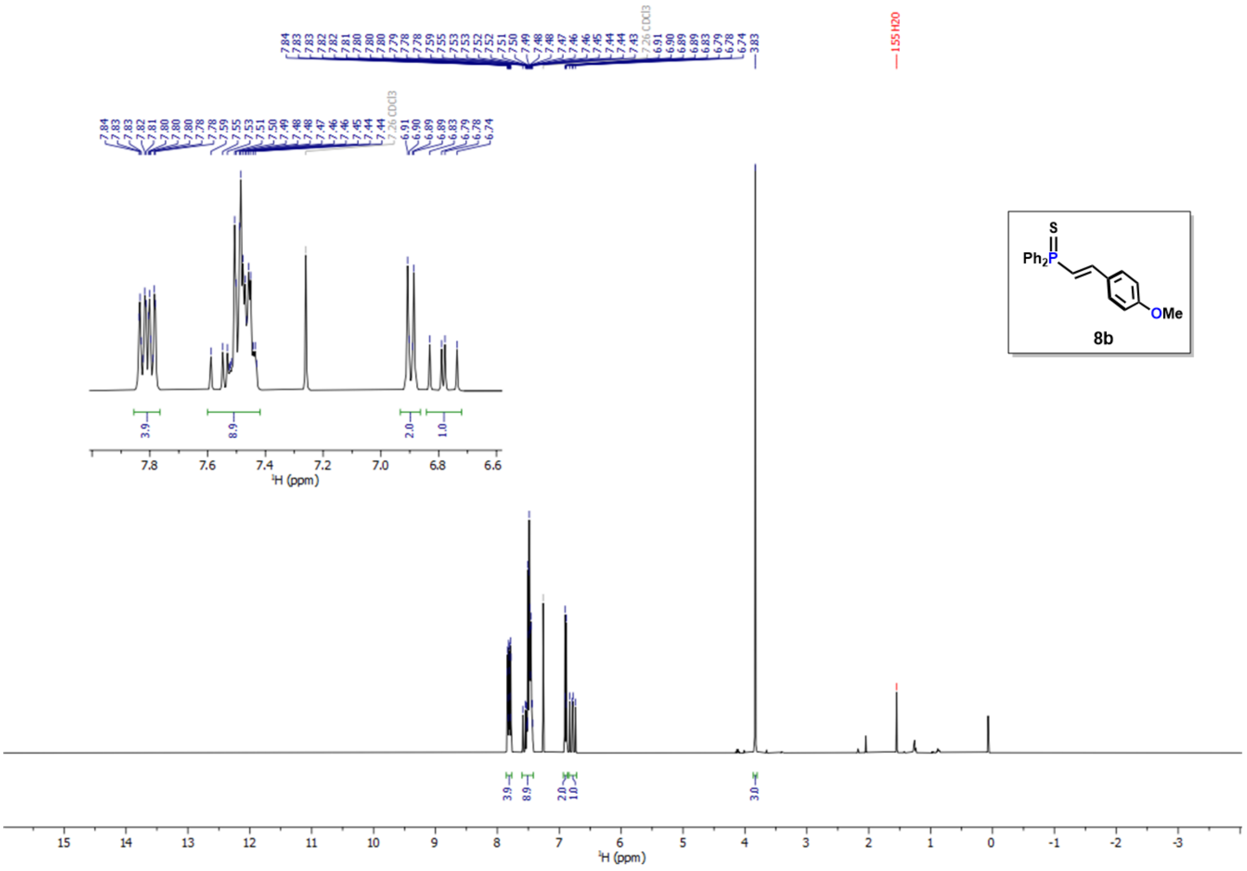

Figure S103. ^{1}H NMR of **8b** (400 MHz, $CDCl_3$, 298 K).

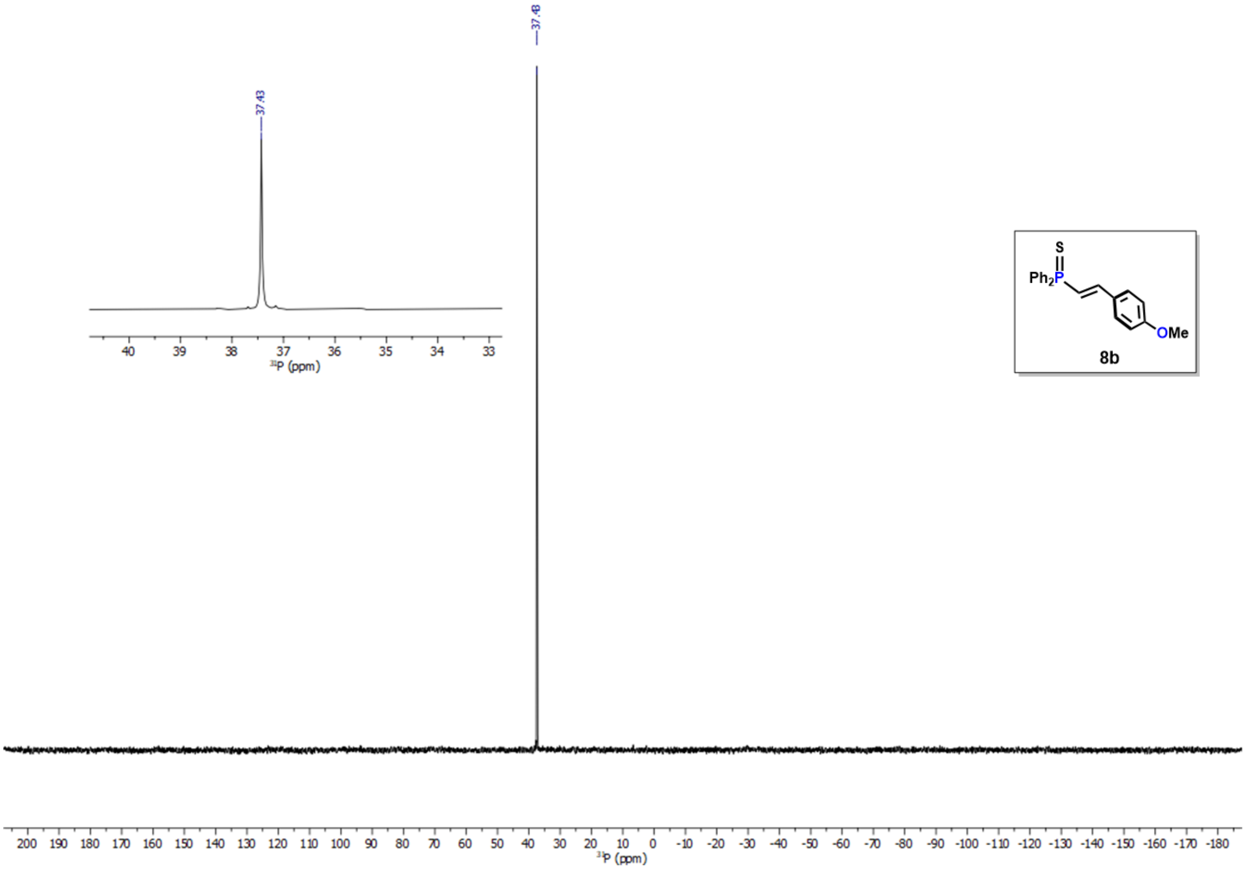

Figure S104. $^{31}P\{^{1}H\}$ NMR of **8b** (162 MHz, $CDCl_3$, 298 K).

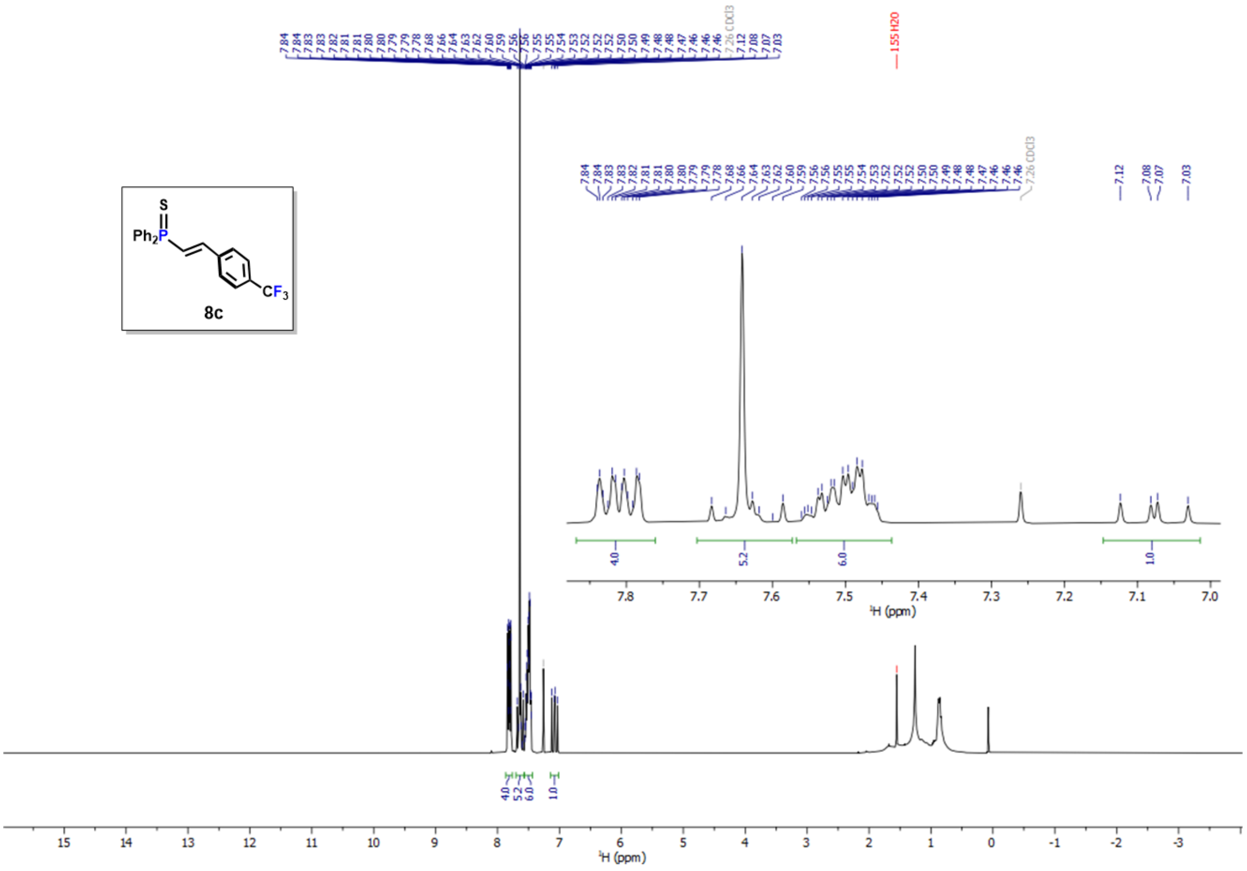

Figure S105. ^{1}H NMR of **8c** (400 MHz, $CDCl_3$, 298 K).

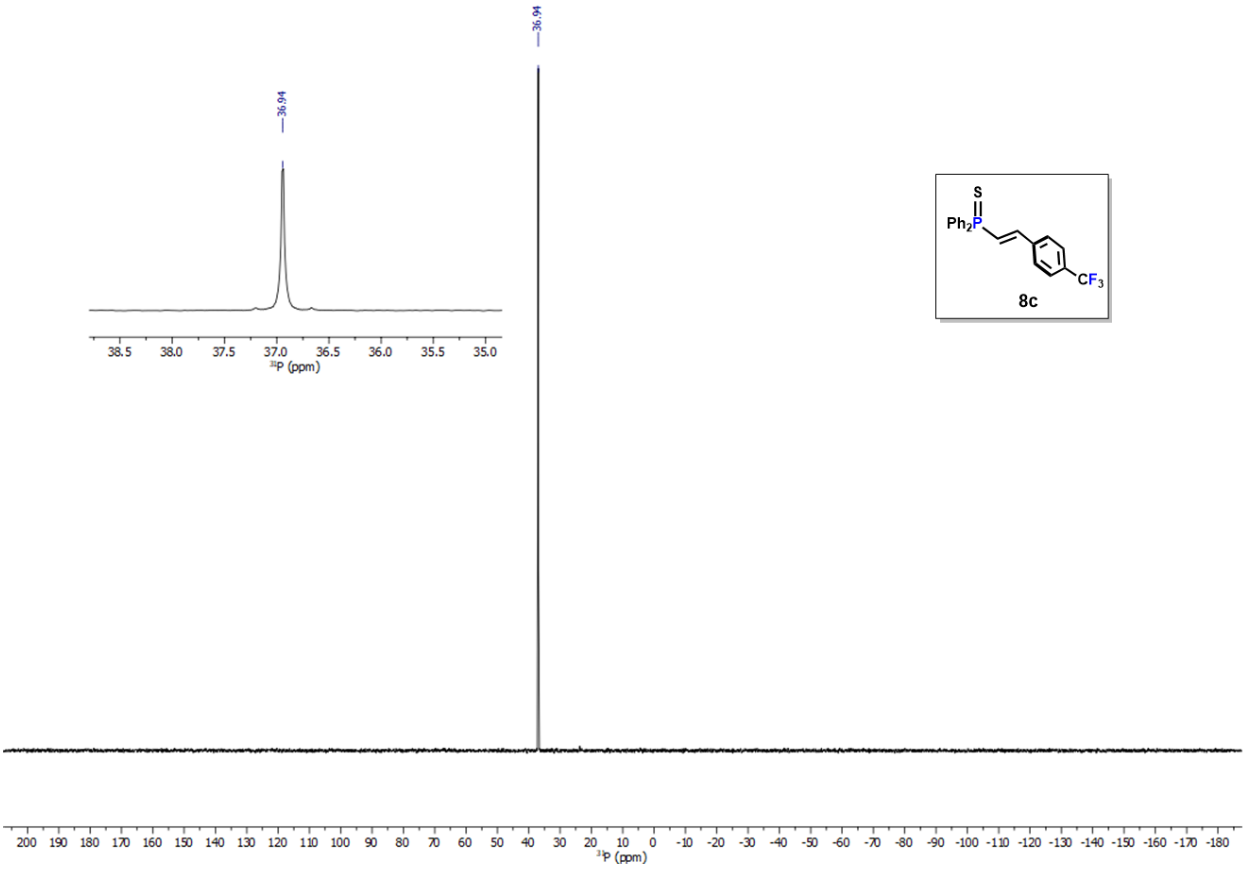

Figure S106. $^{31}P\{^{1}H\}$ NMR of **8c** (162 MHz, $CDCl_3$, 298 K).

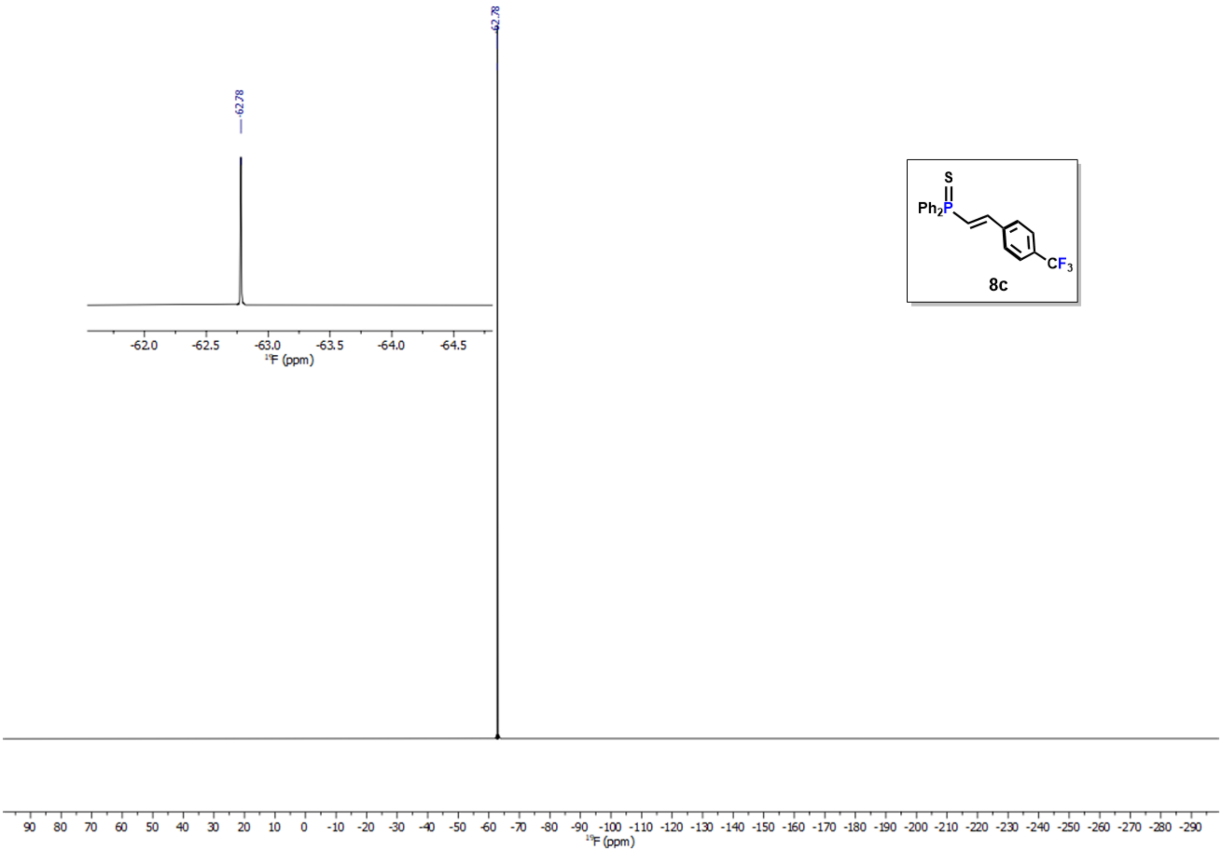

Figure S107. ^{19}F NMR of **8b** (377 MHz, $CDCl_3$, 298 K).

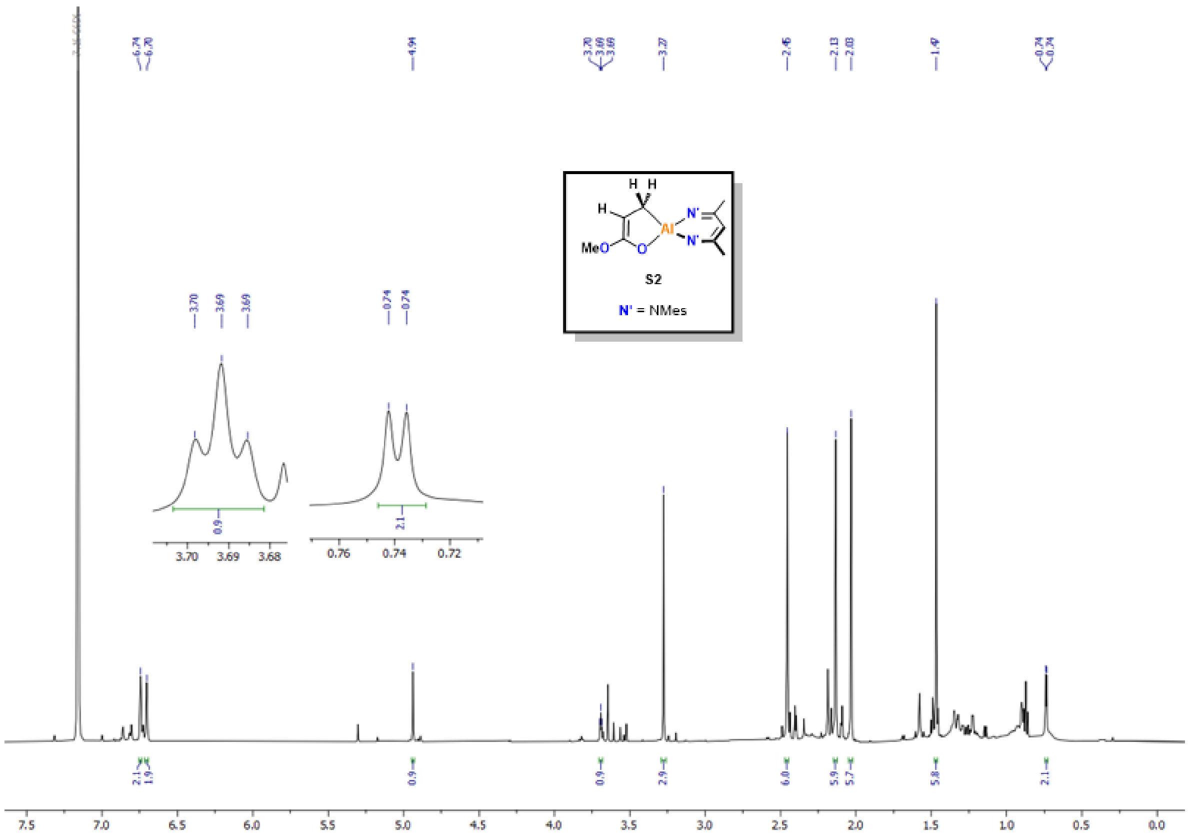

Figure S108. ^{1}H NMR of **S2** (500 MHz, C_6D_6, 298 K).

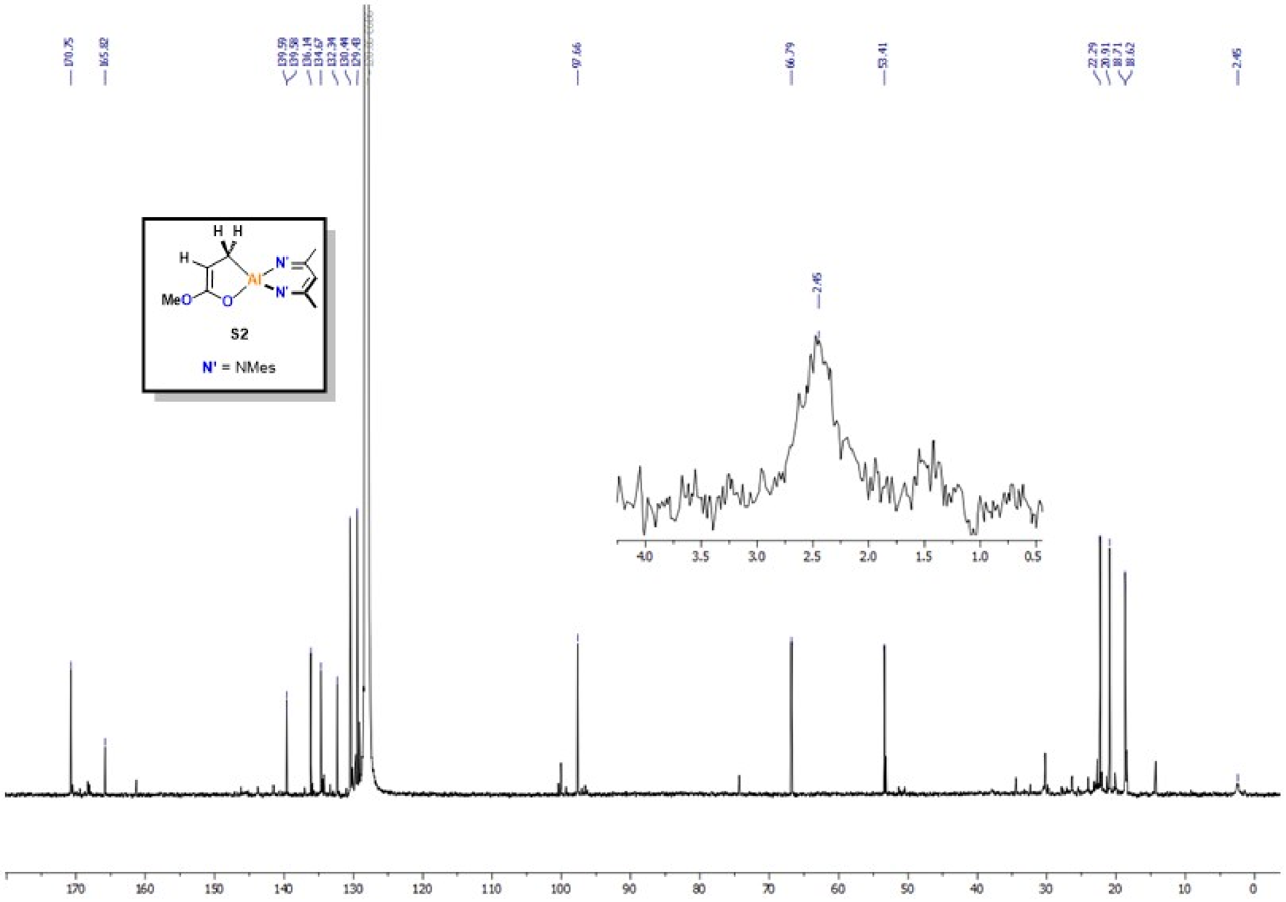

Figure S109. ^{13}C{^{1}H} NMR of **S1** (126 MHz, C_6D_6, 298 K).

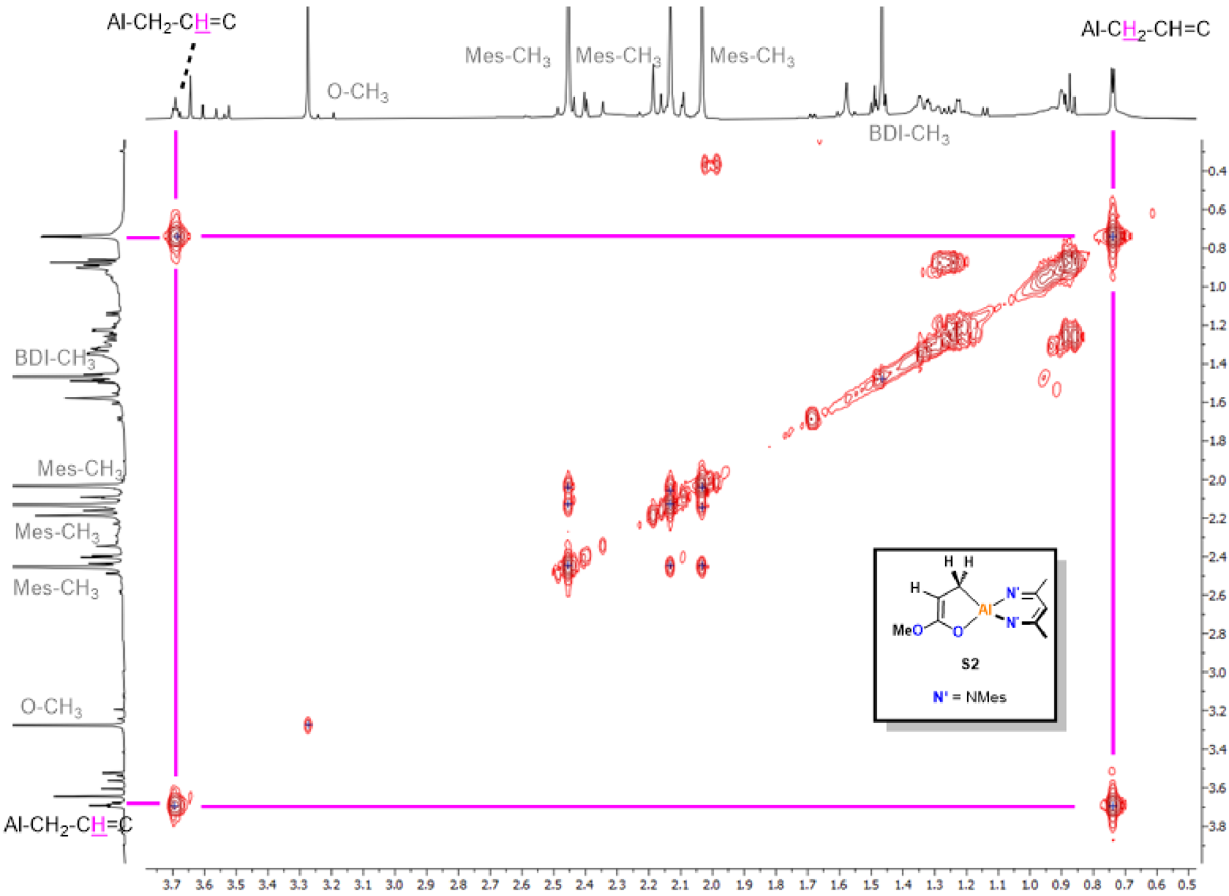

Figure S110. ^{1}H COSY NMR of **S1** (500 MHz, C_6D_6, 298 K).

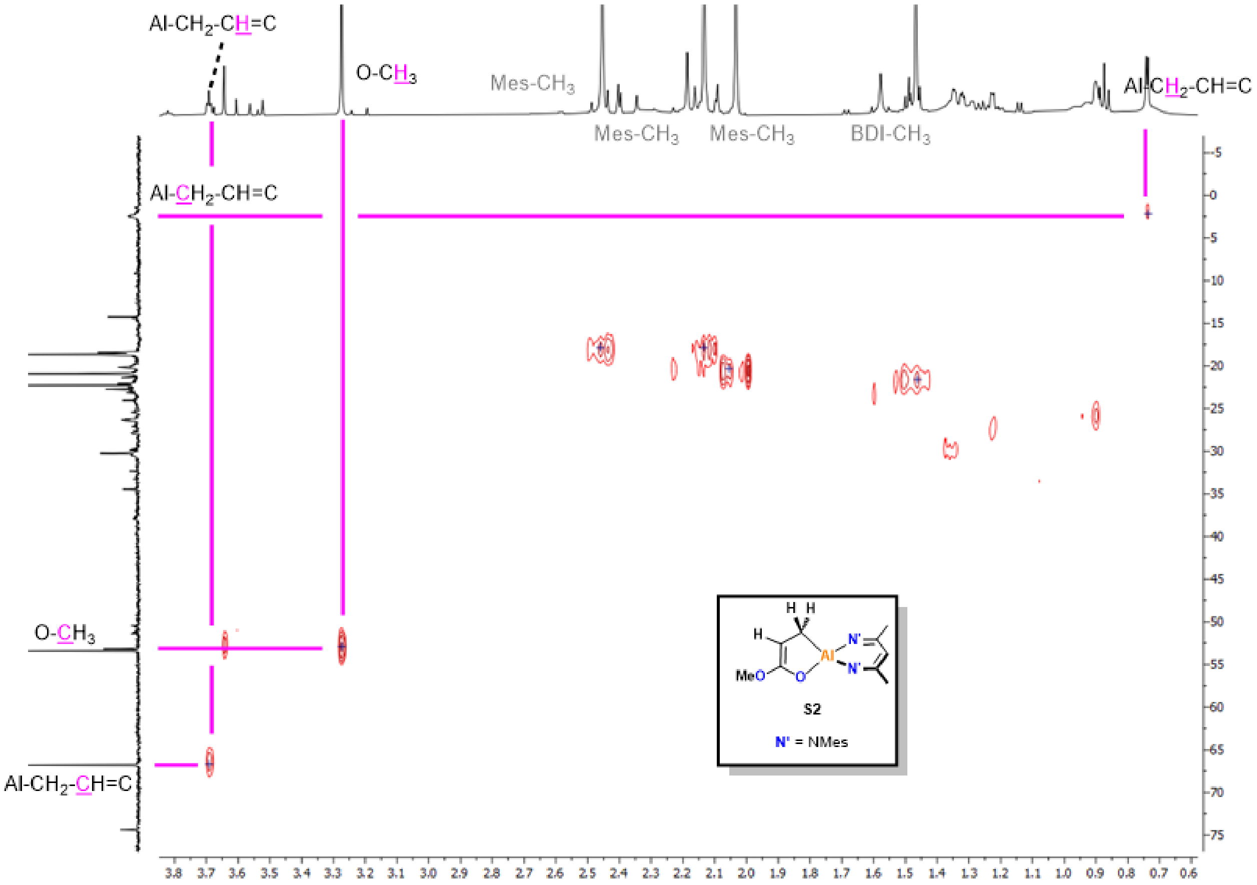

Figure S111. ^{1}H-^{13}C HSQC NMR of **S1** (C_6D_6, 298 K).

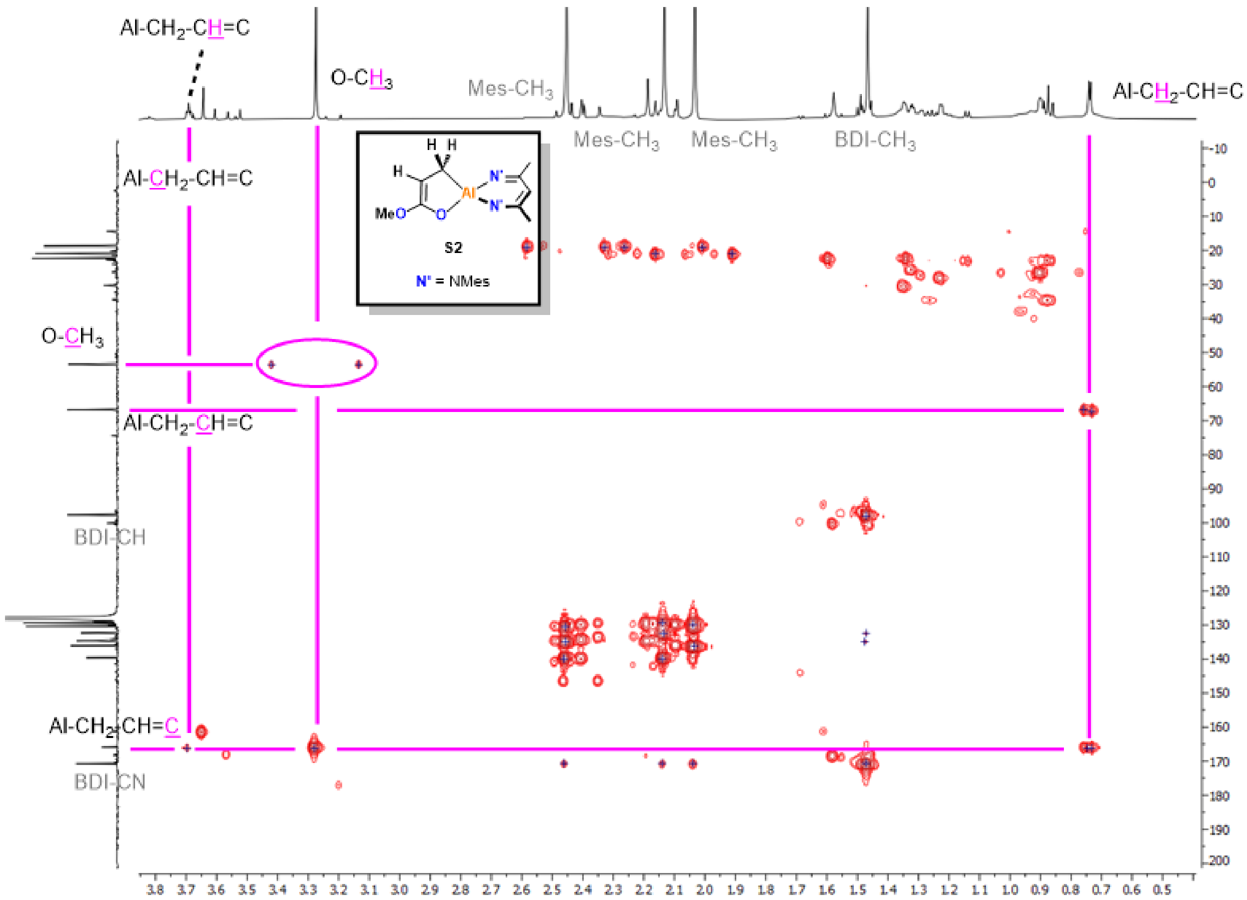

Figure S112. ^{1}H-^{13}C HMBC NMR of **4d** (C_6D_6, 298 K).

8. References

[1] N. Gorgas, A. J. P. White and M. R. Crimmin, *J. Am. Chem. Soc.*, **2022**, *144*, 8770–8777.

[2] (1) Di Giuseppe, A.; De Luca, R.; Castarlenas, R.; Pérez-Torrente, J. J.; Crucianelli, M.; Oro, L. A. Double Hydrophosphination of Alkynes Promoted by Rhodium: The Key Role of an N-Heterocyclic Carbene Ligand. Chem. Commun. **2016**, *52* (32), 5554–5557.

[3] (1) Wang, X.-L.; Chen, J.-X.; Jia, X.-S.; Yin, L. Synthesis of α,β-Unsaturated Phosphine Sulfides. Synthesis (Stuttg). **2020**, *52* (01), 141–149.

[4] Hoops, S.; Sahle, S.; Gauges, R.; Lee, C.; Pahle, J.; Simus, N.; Singhal, M.; Xu, L.; Mendes, P.; Kummer, U. COPASI—a COmplex PAthway SImulator. *Bioinformatics* **2006**, *22*, 3067–3074.

[5] Frisch, M. J.; Trucks, G. W.; Schlegel, H. B.; Scuseria, G. E.; Robb, M. A.; Cheeseman, J. R.; Scalmani, G.; Barone, V.; Mennucci, B.; Petersson, G. A.; Nakatsuji, H.; Caricato, M.; Li, X.; Hratchian, H. P.; Izmaylov, A. F.; Bloino, J.; Zheng, G.; Sonnenberg, J. L.; Hada, M.; Ehara, M.; Toyota, K.; Fukuda, R.; Hasegawa, J.; Ishida, M.; Nakajima, T.; Honda, Y.; Kitao, O.; Nakai, H.; Vreven, T.; Montgomery, J. A., Jr.; Peralta, J. E.; Ogliaro, F.; Bearpark, M.; Heyd, J. J.; Brothers, E.; Kudin, K. N.; Staroverov, V. N.; Kobayashi, R.; Normand, J.; Raghavachari, K.; Rendell, A.; Burant, J. C.; Iyengar, S. S.; Tomasi, J.; Cossi, M.; Rega, N.; Millam, J. M.; Klene, M.; Knox, J. E.; Cross, J. B.; Bakken, V.; Adamo, C.; Jaramillo, J.; Gomperts, R.; Stratmann, R. E.; Yazyev, O.; Austin, A. J.; Cammi, R.; Pomelli, C.; Ochterski, J. W.; Martin, R. L.; Morokuma, K.; Zakrzewski, V. G.; Voth, G. A.; Salvador, P.; Dannenberg, J. J.; Dapprich, S.; Daniels, A. D.; Farkas, Ö.; Foresman, J. B.; Ortiz, J. V.; Cioslowski, J.; Fox, D. J. Gaussian 09, Revision D.01; Gaussian, Inc., Wallingford, CT, **2009**.

[6] NBO 6.0. Glendening, E. D.; Badenhoop, J. K.; Reed, A. E.; Carpenter, J. E.; Bohmann, J. A.; Morales, C. M.; Landis, C. R.; Weinhold, F. Theoretical Chemistry Institute, University of Wisconsin, Madison (2013).

[7] Fukui, K. The path of chemical reactions - the IRC approach. *Acc. Chem. Res.*, **1981**, *14*, 363–368.

[8] Hratchian, H. P.; Schlegel, H. B. Chapter 10 - Finding minima, transition states, and following reaction pathways on ab initio potential energy surfaces. *Theory and Applications of Computational Chemistry – The First Forty Years*, 1; Elsevier: London, 2005, 195–249.

[9] Tomasi, J.; Mennucci, B.; Cammi, R. Quantum Mechanical Continuum Solvation Models. *Chem. Rev.* **2005**, *105*, 2999–3094.

[10] Grimme, S.; Antony, J.; Ehrlich, S.; Krieg, H. A consistent and accurate ab initio parametrization of density functional dispersion correction (DFT-D) for the 94 elements H-Pu. *J. Chem. Phys.* **2010**, *132*, 154104.

[11] Head-Gordon, M.; Chai, J. D. Long-range corrected hybrid density functionals with damped atom–atom dispersion corrections. *Phys. Chem. Chem. Phys.* **2008**, *10*, 6615–6620.

[12] Hehre, W. J.; Ditchfield, R.; Pople, J. A. Self—Consistent Molecular Orbital Methods. XII. Further Extensions of Gaussian—Type Basis Sets for Use in Molecular Orbital Studies of Organic Molecules. *J. Chem. Phys.* **1972**, *56*, 2257–2261.

[13] Hariharan, P. C.; Pople, J. A. The influence of polarization functions on molecular orbital hydrogenation energies. *Theor. Chim. Acta.* **1973**, *28*, 213–222.

[14] Clark, T.; Chandrasekhar, J.; Spitznagel, G. W.; Schleyer, P. V. R. Efficient diffuse function augmented basis sets for anion calculations. III. The 3-21+G basis set for first-row elements, Li–F. *J. Comput. Chem*. **1983**, *4*, 294–301.

[15] C. Lee , W. Yang and R. G. Parr , *Phys. Rev. B: Condens. Matter Mater. Phys.*, **1988**, *37* , 785

[16] A. D. Becke *J. Chem. Phys.*, **1993**, *98* , 5648

[17] Weigend, F.; Ahlrichs, R. Balanced Basis Sets of Split Valence, Triple Zeta Valence and Quadruple Zeta Valence Quality for H to Rn: Design and Assessment of Accuracy. *Phys. Chem. Chem. Phys.* **2005**, *7*, 3297.

[18] Gasevic, T.; Stückrath, J. B.; Grimme, S.; Bursch, M. Optimization of the r^{2}SCAN-3c Composite Electronic-Structure Method for Use with Slater-Type Orbital Basis Sets. *J. Phys. Chem. A* **2022**, *126*, 3826–3838.

[19] Neese, F. Software Update: Software update: The ORCA program system—Version 5.0. *WIREs Comput. Mol. Sci.* **2022**, *12* (5).

[20] Lu, T.; Chen, F. Multiwfn: A Multifunctional Wavefunction Analyzer. *J. Comput. Chem.* **2012**, *33*, 580–592.

[21] Neese, F. Software Update: The ORCA Program System, Version 4.0. *WIREs Comput. Mol. Sci.* **2018**, *8* (1).

[22] Neese, F.; Wennmohs, F.; Becker, U.; Riplinger, C. The ORCA Quantum Chemistry Program Package. *J. Chem. Phys.* **2020**, *152* (22), 224108.

[23] Neese, F.; Wennmohs, F.; Hansen, A.; Becker, U. Efficient, Approximate and Parallel Hartree–Fock and Hybrid DFT Calculations. A 'Chain-of-Spheres' Algorithm for the Hartree–Fock Exchange. *Chem. Phys.* **2009**, *356* (1–3), 98–109.

[24] Pipek, J.; Mezey, P. G. *J. Chem. Phys.* **1989**, *90*, 4916–4926.

[25] Vidossich, P.; Lledós, A. *Dalt. Trans.* **2014**, *43*, 11145.

[26] Hammett, L. P. The Effect of Structure upon the Reactions of Organic Compounds. Benzene Derivatives. *J. Am. Chem. Soc.* **1937**, *59* (1), 96–103.

[27] Jaffé, H. H. A Reëxamination of the Hammett Equation. *Chem. Rev.* **1953**, *53* (2), 191–261.

[28] O. V. Dolomanov, L. J. Bourhis, R. J. Gildea, J. A. K. Howard, H. Puschmann, *J. Appl. Crystallogr.* **2009**, *42*, 339–341.

[29] G. M. Sheldrick, Acta Crystallogr. Sect. *C Struct. Chem.* **2015**, *71*, 3–8.
